# O,S,Se-containing Biginelli products based on cyclic β-ketosulfone and their postfunctionalization

**DOI:** 10.3762/bjoc.20.184

**Published:** 2024-08-27

**Authors:** Kateryna V Dil, Vitalii A Palchykov

**Affiliations:** 1 Research Institute of Chemistry and Geology, Oles Honchar Dnipro National University, Nauky Av. 72, Dnipro, 49045, Ukraine; 2 Enamine Ltd. (www.enamine.net), Winston Churchill Str. 78, Kyiv, 02094, Ukraine

**Keywords:** dihydropyrimidinone/thione/selenone, green chemistry, in silico biological profile, multicomponent reaction (MCR), thiopyrandioxide

## Abstract

A one-pot three-component Biginelli synthesis of dihydropyrimidinones/thiones/selenones via acetic acid or solvent-free Yb(OTf)_3_-catalyzed tandem reaction of β-ketosulfone (dihydro-2*H*-thiopyran-3(4*H*)-one-1,1-dioxide), an appropriate urea, and arylaldehyde has been developed. The reaction proceeds with high chemo- and regioselectivity to give diverse DHPMs in reasonable yields up to 95%. Moreover, an SO_2_-containing analogue of anticancer drug-candidate enastron (SO_2_ vs C=O) was obtained by using the here reported method in gram scale. We also demonstrate the reactivity of the Biginelli product in various directions – synthesis of condensed thiazoles and tetrazoles. In silico assessment of ADMET parameters shows that most compounds meet the lead-likeness requirements. The biological profiles of new compounds demonstrate high probability levels of activity against the following pathogens/diseases: *Candida albicans, Alphis gossypii, Tripomastigote Chagas, Tcruzi amastigota, Tcruzi epimastigota, Leishmania amazonensis,* and *Dengue larvicida.*

## Introduction

**Scheme 1:**
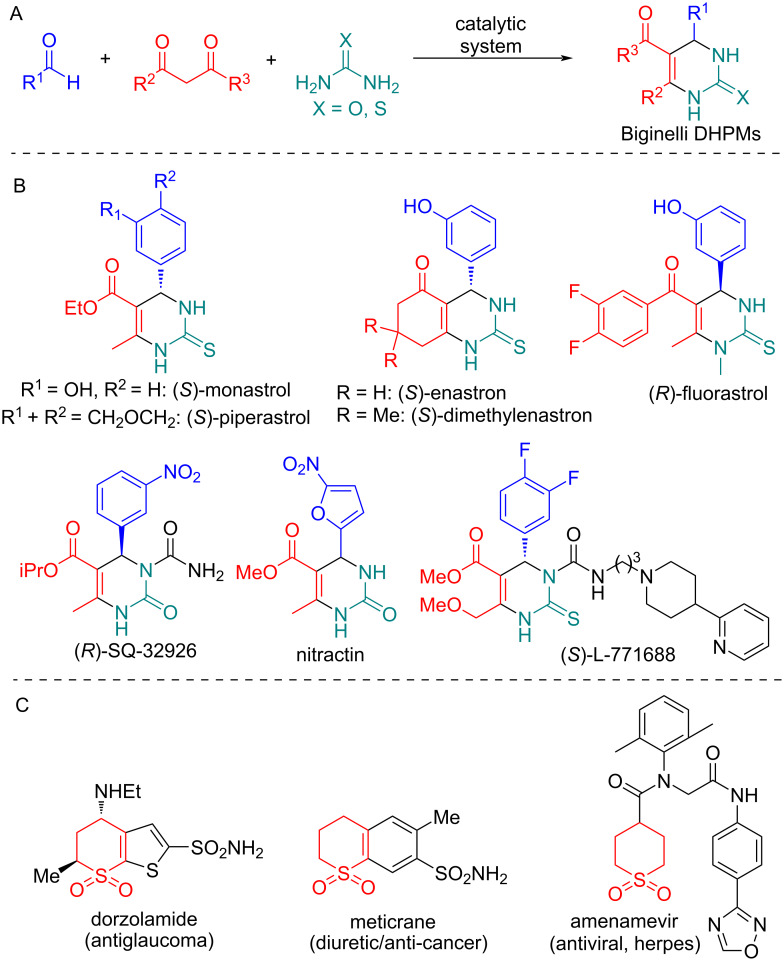
The general Biginelli reaction (A) and examples of DHMP (B) and thiopyran-1,1-dioxide (C) containing drugs.

Multicomponent reactions (MCRs) are the key methodology to access valuable heterocycles for medicinal chemistry projects. The classical Biginelli reaction (1893) is an acid-catalyzed, three-component reaction between an aldehyde, β-ketoester, and urea that produces 3,4-dihydropyrimidin-2(1*H*)-ones, also known as DHPMs (Scheme 1A). This reaction is believed to be one of the most famous MCRs with >2000 papers published in the last 20 years (according to Scopus database). These MCRs allow the direct synthesis of known DHMP drugs such as monastrol, piperastrol, enastron, fluorastrol etc. (Scheme 1B) and dozens of highly bioactive compounds with anticancer, antihypertensive, antiinflammatory, antioxidant, antimicrobial, antifungal, antimalarial, antitubercular, antidiabetic, antifilarial, anti-Alzheimer, antiepileptics and other activities [1–11]. The concept of "privileged structures" in medicinal chemistry highlights derivatives capable of interacting with multiple receptors or enzymes, making them ideal candidates for drug discovery. Dihydropyrimidinones (DHPMs) and their derivatives are particularly noteworthy within this category. Accordingly, their synthesis is of significant interest for organic and medicinal chemists. DHPMs are found in a variety of marine-sourced alkaloids, which are essential for creating biologically active natural products [10]. Some of the DHPM derivatives are also known as functional polymers, adhesives, and fabric dyes [8,12].

**Figure 1:**
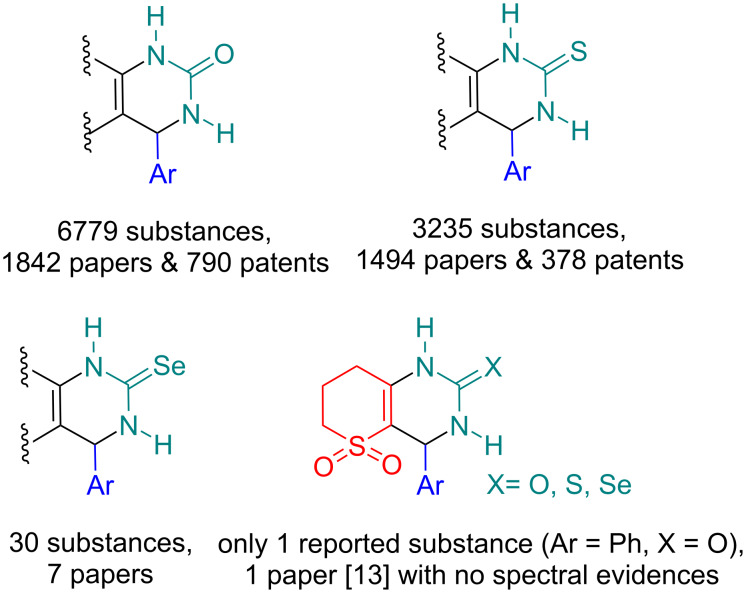
Number of aryl-substituted Biginelli-type products and publications as analyzed by Reaxys database. The search was performed using depicted substructures “on all atoms” (May 2024).

In recent decades, the scope of the original Biginelli reaction shown in Scheme 1A was significantly extended by variation of the 1,3-dicarbonyl-containing building blocks. Many groups have elegantly demonstrated the synthetic versatility of numerous enolizable carbonyl components, including β-keto esters, cyclic/acyclic β-diketones, β-keto amides, coumarins, alicyclic ketones, β-ketophosphonates, α-nitroketones, curcumin, and barbituric acid derivatives [1–2,8–9]. We analyzed a number of Biginelli-type products and publications and concluded that Se-containing DHPMs among the rarest examples and, in addition to this, ketosulfones have never been used as enolizable carbonyl component in this chemistry. To the best of our knowledge only one compound from the target group was published (2003) [13] before this work, however, without any spectral evidence (Figure 1).

Considering our constant interest in the development of methods for the synthesis of new S-heterocyclic systems we tried to combine the broad synthetic potential of Biginelli condensation and high reactivity β-ketosulfone **1** (dihydro-2*H*-thiopyran-3(4*H*)-one-1,1-dioxide) in various condensation reactions [14–20]. It was also worth mentioning that the thiopyran-1,1-dioxide motif is presented in a number of biologically active compounds including important market drugs as antiglaucoma agent dorzolamide [21], diuretic/anticancer meticrane [22] and antiherpesvirus agent amenamevir (ASP-2151) [23] which was recently synthesized by Ugi-4CR [24] (Scheme 1C).

## Results and Discussion

### Reaction optimization

Over the past two decades more than 300 various catalytic systems have been proposed for Biginelli chemistry, e.g., simple inorganic and organic acids, metal salts, metal oxides, ionic liquids, phosphines, nanocatalysts, organocatalysts, ion exchange resins [1,4,9] or even visible light-driven methods [25–28].

We started our study from the optimization of the reaction conditions using β-ketosulfone **1**, benzaldehyde and thiourea as model reaction. According to the literature, the reaction has been shown to work best and most efficiently under acidic conditions since such conditions enhance the selectivity, so various catalysts, mainly acidic, were tested for the model Biginelli reaction and the results are shown in Table 1.

**Table 1:** Optimization of reaction.

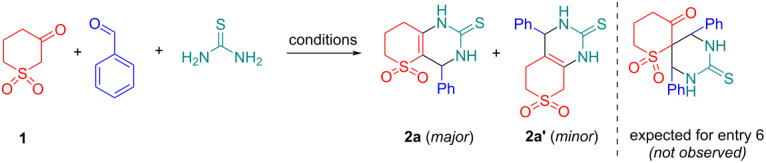			

Entry	Conditions^а^	Isolated yield **2a** + **2a’**, %	Isomer ratio, **2a:2a’**

1	0.8 equiv NaI, 0.8 equiv TMSCl, MeCN, 22 °C, 4 h	traces	n.d.
2	4 equiv TMSCl, DMF, 60 °C, 14 h	traces	n.d.
3	4 equiv TMSCl, DMF, −20 °C, 5 days	23	2:1
4	4 equiv TMSCl, DMF, 22 °C, 3 days	59	2:1.2
5	4 equiv TMSCl, DMF/MeCN (1:1), 22 °C, 2 days	51	3:1
6^b^	3 equiv TMSCl, DMF, 22 °C, 5 days	72	2:1
7	3 equiv HСl, EtOH, 82 °C, 6 h	23	1:0
8	10 mol % *p*-TsOH, MeOH, 82 °C, 36 h	35	1:0
9	10 mol % CAN, EtOH, 82 °C, 18 h	32	1:0
10	10 mol % SrCl_2_·6H_2_O, EtOH, 82 °C, 18 h	8	1:0
11	10 mol % La(NO_2_)_3_·6H_2_O, solvent-free, 82 °С, 18 h	61	1:0
**12** **^c^**	**10 mol % Yb(OTf)** **_3_** **, solvent-free, 140 °C, 6 h**	**65**	**1:0**
13^c^	AcOH, 110 °C, 14 h	68	1:0
14	AcOH, 82 °C, 14 h	40	1:0
15	AcOH, 110 °C, 4 h	71	1:0
**16** **^c^**	**AcOH, 110 °C, 4 h**	**74**	**1:0**
17^d^	TFA, 110 °C, 14 h	45	1:0
18^c^	AcOH, MW (300 W), 120 °С, 20 min	36	1:0

^a^Unless specifically stated the ratio of ketosulfone/benzaldehyde/thiourea 1:1:1 (1 mmol scale); ^b^ketosulfone (1 mmol, 1 equiv), benzaldehyde (2 mmol, 2 equiv), thiourea (1 mmol, 1 equiv); ^c^ketosulfone (1 mmol, 1 equiv), benzaldehyde (1 mmol, 1 equiv), thiourea (1.2 mmol, 1.2 equiv); ^d^ketosulfone (1 mmol, 1 equiv), benzaldehyde (1 mmol, 1 equiv), thiourea (3 mmol, 3 equiv).

One of the most effective promoters for this type of reaction is TMSCl [29–31] and we also tried to involve TMSCl in our study (Table 1, entries 1–6), but in any case, we received a mixture of regioisomeric products **2a**/**2a’** (3:1 to 2:1) confirmed by 2D NMR spectroscopy (see Supporting Information File 1, Figures S1–S6). Classical conditions for Biginelli reaction (reflux in ethanolic HCl) gave only 23% yield (Table 1, entry 7). Reflux in alcoholic media in the presence of *p*-toluenesulfonic acid or CAN (ceric ammonium nitrate) was also not particularly successful (Table 1, entries 8 and 9, yield 32–35%). We subsequently explored a range of reaction conditions to improve overall yield and selectivity. We experimented with Lewis acids (Table 1, entries 10–12) to catalyze the reaction and observe low conversion with SrCl_2_. The reaction was significantly better with both La(NO_2_)_3_ and Yb(OTf)_3_ under solvent-free conditions (Table 1, entries 11 and 12, yield 61–65%). The most promising results were obtained by using simplest heating in acetic acid (Table 1, entries 13–16). After some playing with temperature, reaction time, and ratio of starting reagents we ultimately found conditions (AcOH, 110 ^o^C, 4 h) suitable best for our chemistry and leading to the yield of the target product **2a** 74% (Table 1, entry 16). However, increasing acidity and using trifluoroacetic acid (Table 1, entry 17) did not improve the overall yield. We also tried microwave activation conditions since this is a known technique for reactions of this type [32], but unfortunately, we did not find any improvement in the yield (Table 1, entry 18).

### Reaction scope

**Scheme 2:**
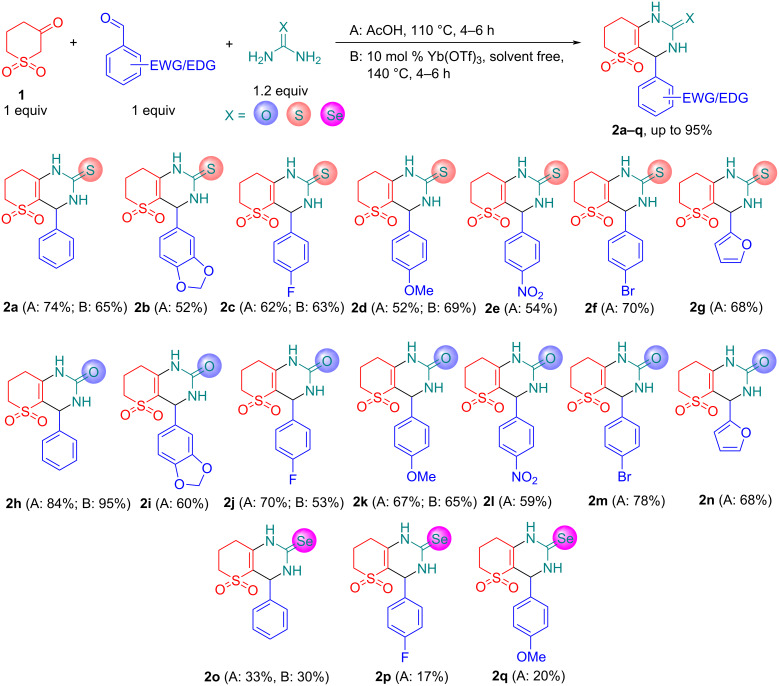
Scope of the obtained Biginelli products **2a**–**q**.

We then used optimized reaction conditions from Table 1, entry 16 (method A) and 12 (method B) to further explore the scope of the reaction (Scheme 2). By employing various EWG/EDG-substituted benzaldehydes and urea/thiourea/selenourea we synthesized novel Biginelli products **2a**–**q** with up to 95% yield. The use of selenourea has been shown to give low yields of products (up to 33%). In fact, we have thus expanded the range of available selenium-containing DHPMs in addition to the work of other authors [33–35].

We also attempted to replace urea/thiourea/selenourea with *N*-alkyl/aryl-substituted analogues, and aldehyde component switch to heteroaromatic (2-pyridinaldehyde) and aliphatic (iPrCHO, cinnamaldehyde). Unfortunately, we failed in both replacements and were unable to obtain any reasonable products. The list of unsuccessful reagents is shown in Figure S7 (see Supporting Information File 1).

**Scheme 3:**
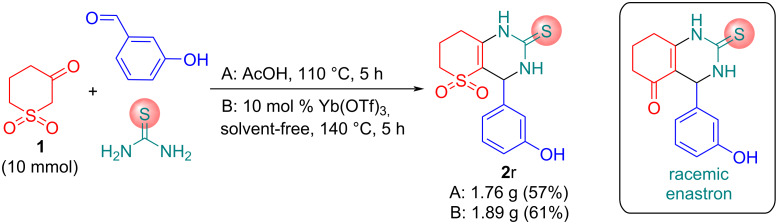
Synthesis of SO_2_-containing enastron analogue **2r**.

Next, we paid attention to testing the conditions we developed for the synthesis of the SO_2_-containing analogue **2r** of potent anticancer drug enastron in gram scale (Scheme 3). Enastron is a novel dihydropyrimidine-based mitotic kinesin spindle protein KSP/Eg5 inhibitor [36]. We hope that compound **2r** and its analogues obtained in this work can be further deeply studied by in silico and in vitro methods to discover the compound most suitable for clinical trials.

The structures of the synthesized compounds **2a**–**r** were confirmed by spectral data. The ^1^H NMR spectra of the obtained products are characterized by the following signals: aromatic ring protons (6.25–8.23 ppm), Ar–CH group proton singlet (5.13–5.44 ppm), broadened NH singlets (7.78–10.89 ppm), as well as the corresponding signals of successive 3 × CH_2_ groups of the sulfone fragment (2.18–3.34 ppm). Such a set of signals clearly corresponds to the heterocycles depicted above. Figure S8 (see Supporting Information File 1) shows ^1^H NMR spectra for Ph-substituted O,S,Se-DHPMs **2a,h,o**. It can be seen from them that when going from oxygen to selenium (**2h**→**2a**→**2o**), the signals of both NH groups shift to down field (7.87/9.26→9.75/10.50→10.26/10.85 ppm) and become more equivalent (Δδ = 1.39→0.75→0.59 ppm accordingly). The key signal in the ^13^C NMR spectra is located in the regions 174.04–174.69 ppm (C=S), 151.44–151.86 ppm (C=O), and 170.15–170.91 ppm (C=Se) accordingly.

### Utilization of reaction products

**Scheme 4:**
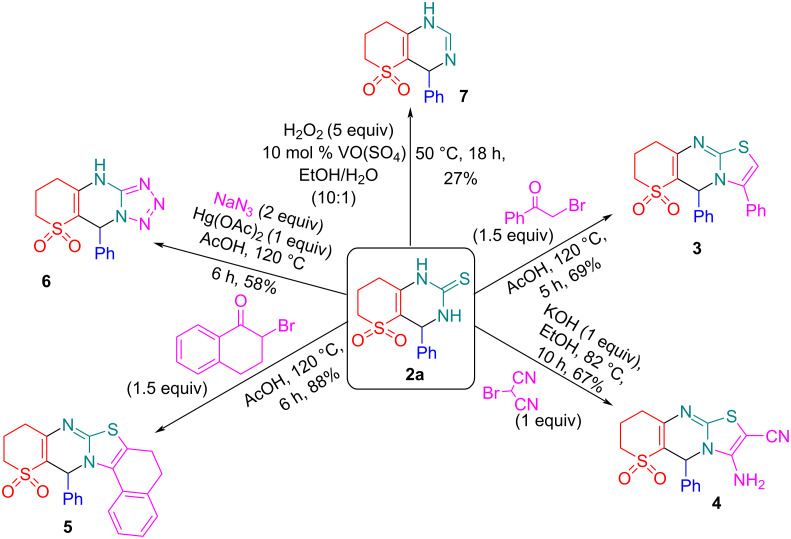
Postmodification of the Biginelli product **2a**.

The Biginelli reaction is the traditional method for synthesizing DHPM scaffolds, but it faces limitations in product diversity. To overcome these challenges, two main strategies have been developed. The first strategy involves modifying the conventional components of the Biginelli chemistry, while the second focuses on the postmodification of the Biginelli products [2]. Both approaches were tested in this work. We used Hantzsch-type thiazole synthesis for postmodification of product **2a**. By employing 2-bromoacetophenone, bromomalononitrile and 2-bromo-1-tetralone we obtained condensed thiazoles **3**–**5** in 67–88% yields using slightly modified methods described [37–39]. We also applied desulfurization to obtain products **6** and **7** [40–41] (Scheme 4).

While the Hantzsch thiazole synthesis is well documented from a synthetic and mechanistic point [42–43] and do not need discussion, more desulfurizations and oxidations of rare Biginelli products are discussed in Supporting Information File 1 using compound **2a** as an example.

### In silico evaluation of ADMET parameters and biological profile

**Figure 2:**
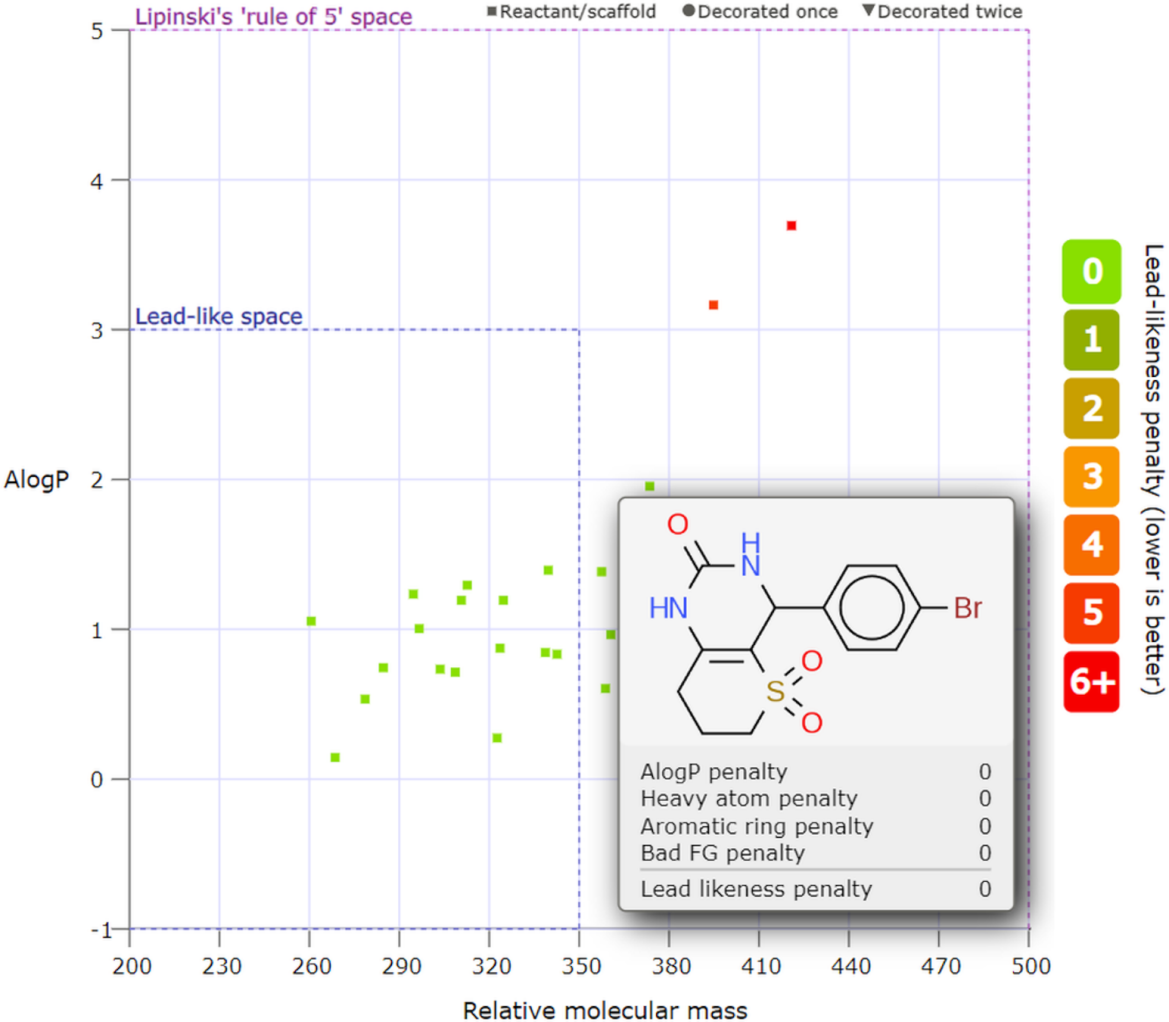
Distribution of compounds **2a**–**r**, **3**–**7** (log *P* (*y*)–MW (*x*)) through LLAMA software. The chemical structure of a representative compound **2m** is shown.

We performed in silico screening of the biological properties of Biginelli products **2a**–**r** and their postfunctionalized derivatives **3**–**7** using SwissADME (http://www.swissadme.ch) [44], ProTox 3.0 (https://tox-new.charite.de) [45], and MolPredictX (https://www.molpredictx.ufpb.br) [46] free online software. Considering ADMET [47] and other crucial properties, we found that all compounds (except for the nitro derivative **2e**) do not violate the Lipinski, Ghose, Veber, Egan, and Muegge rules and PAINS filter [48–52]. The lipophilicity (estimated as log *P*_o/w_) for all compounds was shown to be in a wide range from 0.43 to 4.11. The topological polar surface area (TPSA), which is important for oral bioavailability, was found to be 67–129 Å^2^ for all compounds except products **4** and **2e**. None of the compounds penetrate the BBB (blood–brain barrier) except for seleno-**2p** and desulfurized product **7**. Regarding water solubility, we can conclude that all compounds are either soluble or moderately soluble. For more details, see Supporting Information File 1 (Table S1). To visualize the lead-likeness of compounds **2a**–**r**, **3–7**, we utilized the free online software LLAMA (https://llama.leeds.ac.uk) [53], which showed that 70% of the products (16 of 23) fall within the specific lead-like space (Figure 2).

We then used the free online software ProTox 3.0 for computational toxicity assessment of the products as their LD_50_ values. The tested compounds mainly belong to the 4th class of acute oral toxicity with 300 < LD_50_ ≤ 2000 mg/kg. In addition, we used MolPredictX (https://www.molpredictx.ufpb.br) [46] to evaluate potential biotargets (pathogens, species, diseases) for new synthesized compounds. If we focus on high levels of biological activity probability (80% and above), the following pathogens and diseases may be potential areas of interest: *Alphis gossypii, Tripomastigote Chagas, Candida albicans, Tcruzi amastigota, Leishmania amazonensis, Tcruzi epimastigota, Dengue larvicida,* and for selected cases *Alzheimer* and *Sars-COVID.* For more details, see Table S1 (Supporting Information File 1).

## Conclusion

In summary, we have demonstrated the catalytic regioselective Biginelli synthesis of new S-heterocyclic systems ‒ 4-aryl-4,6,7,8-tetrahydro-1*H*-thiopyrano[3,2-*d*]pyrimidine-2(3*H*)-one/thione/selenone 5,5-dioxides and some of their derivatives. Furthermore, this methodology was successfully applied for the synthesis of the SO_2_-containing analogue of the anticancer drug-candidate enastron (SO_2_ vs C=O), and we believe a multitude of other sulfones of both synthetic and biological importance can be obtained by using the in this work reported efficient, multicomponent and green protocol. We postfunctionalized the typical Biginelli product using Hantzsch-type thiazole chemistry and desulfurization. Assessing drug-likeness, we found that most of the synthesized compounds correspond to the parameters established by the Lipinski, Ghose, Veber, Egan, and Muegge rules. In silico screening of their biological profiles indicated that these new derivatives fall into the 4th class of acute toxicity. Additionally, they exhibit potential high activity against diseases associated with these species: *Alphis gossypii, Tripomastigote Chagas, Candida albicans, Tcruzi amastigota, Leishmania amazonensis, Tcruzi epimastigota, Dengue larvicida,* and for selected cases *Alzheimer* and *Sars-COVID.*

## Supporting Information

File 1 Experimental procedures and characterization data of new compounds.

## Data Availability

The data that supports the findings of this study is available from the corresponding author upon reasonable request.
