# Supplementary material for: O,S,Se-containing Biginelli products based on cyclic β-ketosulfone and their postfunctionalization
Source: Beilstein J Org Chem. 2024 Aug 27;20:2143–51. doi: 10.3762/bjoc.20.184 (PMC11368051; doi:10.3762/bjoc.20.184)
Supplement: File 1 — Experimental procedures and characterization data of new compounds. [file Beilstein_J_Org_Chem-20-2143-s001.pdf]

BEILSTEIN JOURNAL OF ORGANIC CHEMISTRY

Supporting Information

for

O,S,Se-containing Biginelli products based on cyclic β-ketosulfone and their postfunctionalization

Kateryna V. Dil and Vitalii A. Palchykov

Beilstein J. Org. Chem. **2024,** *20,* 2143–2151. doi:10.3762/bjoc.20.184

Experimental procedures and characterization data of new compounds

License and Terms: This is a supporting information file under the terms of the Creative Commons Attribution License (https://creativecommons.org/licenses/by/4.0). Please note that the reuse, redistribution and reproduction in particular requires that the author(s) and source are credited and that individual graphics may be subject to special legal provisions.
The license is subject to the *Beilstein Journal of Organic Chemistry* terms and conditions: (https://www.beilstein-journals.org/bjoc/terms)

CONTENTS:

General experimental……………………………………………………………………………S2

Methods and compounds characterization…………………………………………………S3

NMR spectra……………………………………………………………………………………….S14

In silico evaluation of ADMET parameters and biological profile…………………………S42

Plausible mechanisms of desulfurization for compound 2a……………........................S126

General experimental

All chemicals were supplied by Enamine Ltd. (www.enamine.net). All solvents were purified according to standard methods. Thin-layer chromatography (TLC) was carried out using Merck aluminium backed DC 60 F254 0.2 mm precoated plates. Spots were then visualized by the quenching of ultraviolet light fluorescence (λ_{max} 254 nm) and then stained and heated with potassium permanganate solution. ^{1}H NMR spectra were recorded at 500 or 400 MHz, and ^{13}C NMR spectra were recorded at 126 or 101 MHz using Bruker spectrometers. ^{1}H and ^{13}C NMR chemical shifts are calibrated using residual undeuterated DMSO (δ = 2.50 ppm for ^{1}H, 39.52 ppm for ^{13}C). Coupling constants (*J*) are given in Hz, multiplicities are given as s (singlet), d (doublet), dd (doublet of doublets), t (triplet), m (multiplet) and br (broad). High-resolution mass spectra (HRMS) were recorded on an Agilent 6224 TOF LC/MS mass spectrometer by electrospray ionization time-of-flight reflectron experiments. Starting dihydro-2*H*-thiopyran-3(4*H*)-one **1** was synthesized as described [*Synth. Commun*. **2018**, *48*, 2198-2205 https://doi.org/10.1080/00397911.2018.1486427]. All reactions were performed in 5 mL Wheaton V-vials (Thermo Fisher Scientific Inc.) with PTFE screw cap under argon atmosphere using preheated oil bath where necessary.

General method A for the synthesis of compounds 2a–r. A stirred reaction mixture of dihydro-2*H*-thiopyran-3(4*H*)-one **1** (148 mg, 1 mmol, 1 equiv), an appropriate urea (1.2 mmol, 1.2 equiv), and aromatic aldehyde (1 mmol, 1 equiv) in acetic acid (1 mL) was heated at 110 °C for 4–6 h. The progress of the reaction was monitored by TLC. After completion the reaction, solvent was evaporated in vacuo and the semi-crystalline residue was triturated or recrystallized from isopropyl alcohol.

General method B for the synthesis of compounds 2a,c,d,h,j,k,o,r. A stirred reaction mixture of dihydro-2*H*-thiopyran-3(4*H*)-one **1** (148 mg, 1 mmol), an appropriate urea (1.2 mmol), aromatic aldehyde (1 mmol), and $Yb(OTf)_3$ (62 mg, 10 mol %, 0.1 equiv) was heated

without solvent at 140 °C for 4–6 h. After cooling, the reaction mixture was triturated with water, and then recrystallized from isopropyl alcohol.

4-Phenyl-3,4,7,8-tetrahydro-1*H*-thiopyrano[3,2-*d*]pyrimidine-2(6*H*)-thione 5,5-dioxide (2a)

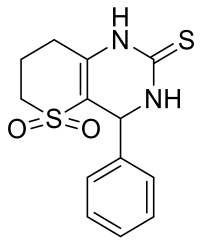

Yield: **A** 218 mg (74%), **B** 191 mg (65%), m.p. 176-177°C, white powder. ^{1}H NMR (500 MHz, DMSO-d_6), δ, ppm (J, Hz): 10.50 (1H, s, NH), 9.74 (1H, s, NH), 7.38 (2H, m, H^{Ar}), 7.30 (3H, m, H^{Ar}), 5.24 (1H, s, CH), 3.28 (2H, m, CH_2), 2.52 (2H, m, CH_2), 2.21 (2H, m, CH_2). ^{13}C NMR (126 MHz, DMSO-d_6), ppm: 174.29 (C=S), 141.77, 140.33, 128.61, 128.08, 126.66, 109.54, 50.18, 50.01, 25.18, 18.15. HRMS (ESI-TOF), *m/z*: found 295.0563, calculated for $C_{13}H_{15}N_2O_2S_2$ $[M+H]^+$ 295.0569.

4-(Benzo[*d*][1,3]dioxol-5-yl)-3,4,7,8-tetrahydro-1*H*-thiopyrano[3,2-*d*]pyrimidine-2(6*H*)-thione 5,5-dioxide (2b)

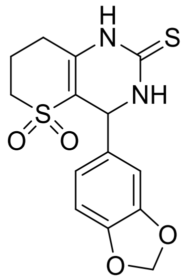

Yield: **A** 177 mg (52%), m.p. 122-125°C, white powder. ^{1}H NMR (500 MHz, DMSO-d_6), δ, ppm (J, Hz): 10.49 (1H, s, NH), 9.69 (1H, s, NH), 6.90 (1H, d, J=7.8 Hz, H^{Ar}), 6.80 (1H, s, H^{Ar}), 6.76 (1H, d, J=7.8 Hz, H^{Ar}), 6.02 (2H, d, J=3.0 Hz, OCH_2O), 5.16 (1H, s, CH), 3.28 (2H, m, CH_2), 2.53 (2H, m, CH_2), 2.20 (2H, m, CH_2). ^{13}C NMR (126 MHz, DMSO-d_6), ppm: 174.04 (C=S), 147.41, 147.08, 140.37, 135.75, 120.25, 109.56, 108.15, 107.00, 101.18, 49.99, 49.86, 25.15, 18.14. HRMS (ESI-TOF), *m/z*: found 339.0460, calculated for $C_{14}H_{15}N_2O_4S_2$ $[M+H]^+$ 339.0468.

4-(4-Fluorophenyl)-3,4,7,8-tetrahydro-1*H*-thiopyrano[3,2-*d*]pyrimidine-2(6*H*)-thione 5,5-dioxide (2c)

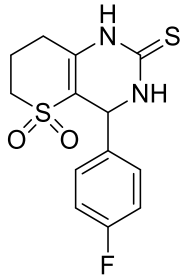

Yield: **A** 193 mg (62%), **B** 197 (63%), m.p. 139-142°C, white powder. ^{1}H NMR (DMSO-d_6, 500 MHz), *δ*, ppm (*J*, Hz): 10.54 (1H, s, NH), 9.76 (1H, s, NH), 7.33 (2H, dd, *J*=8.4 Hz, *J*=5.6 Hz, H^{Ar}), 7.21 (2H, t, *J*=8.4 Hz, H^{Ar}), 5.25 (1H, s, CH), 3.29 (2H, m, CH_2), 2.55 (2H, m, CH_2), 2.21 (2H, m, CH_2). ^{13}C NMR (DMSO-d_6, 126 MHz), *δ*, ppm: 174.20 (C=S), 161.82 (d, $^{1}J_{C\text{-}F}$=243.1 Hz), 140.45, 138.05, 128.86 (d, $^{3}J_{C\text{-}F}$=8.5 Hz), 115.43 (d, $^{2}J_{C\text{-}F}$=21.8 Hz), 109.42, 49.98, 49.54, 25.16, 18.15. HRMS (ESI-TOF), *m/z*: found 313.0470, calculated for $C_{13}H_{14}FN_2O_2S_2$ $[M+H]^+$ 313.0475.

4-(4-Methoxyphenyl)-4,6,7,8-tetrahydro-1*H*-thiopyrano[3,2-d]pyrimidine-2(3*H*)-thione 5,5-dioxide (2d)

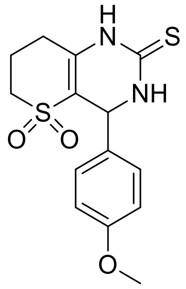

Yield: **A** 168 mg (52%), **B** 224 mg (69%), m.p. 108-111°C, white powder. ^{1}H NMR (DMSO-d_6, 500 MHz), *δ*, ppm (*J*, Hz): 10.45 (1H, s, NH), 9.68 (1H, s, NH), 7.20 (2H, d, *J*=8.2 Hz, H^{Ar}), 6.92 (2H, d, *J*=8.2 Hz, H^{Ar}), 5.19 (1H, s, CH), 3.74 (3H, s, CH_3), 3.26 (2H, m, CH_2), 2.50 (2H, m, CH_2), 2.20 (2H, m, CH_2). ^{13}C NMR (DMSO-d_6, 126 MHz), *δ*, ppm: 174.04 (C=S), 159.05, 140.04, 133.99, 131.81, 127.97, 114.52, 113.92, 109.80, 55.13, 50.02, 49.64, 25.15, 18.16. HRMS (ESI-TOF), *m/z*: found 325.0669, calculated for $C_{14}H_{17}N_2O_3S_2$ $[M+H]^+$ 325.0675.

4-(4-Nitrophenyl)-3,4,7,8-tetrahydro-1*H*-thiopyrano[3,2-*d*]pyrimidine-2(6*H*)-thione 5,5-dioxide (2e)

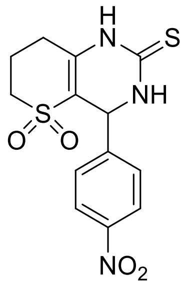

Yield: **A** 183 mg (54%), m.p. 158-160°C, yellow powder. ^{1}H NMR (DMSO-d_6, 500 MHz), *δ*, ppm (*J*, Hz): 10.67 (1H, s, NH), 9.87 (1H, s, NH), 8.19 (1H, d, *J*=8.3 Hz, H^{Ar}), 7.73 (2H, d, *J*=8.3 Hz, H^{Ar}), 5.44 (1H, s, CH), 3.32 (2H, m, CH_2), 2.54 (2H, m, CH_2), 2.22 (2H, m, CH_2). ^{13}C NMR (DMSO-d_6, 126 MHz), *δ*, ppm: 174.53 (C=S), 147.89, 143.71, 141.21, 133.50, 130.45, 123.21, 121.54, 108.66, 49.97, 49.62, 25.20, 18.19. HRMS (ESI-TOF), *m/z*: found 340.0425, calculated for $C_{13}H_{14}N_3O_4S_2$ $[M+H]^+$ 340.0420.

4-(4-Bromophenyl)-4,6,7,8-tetrahydro-1*H*-thiopyrano[3,2-*d*]pyrimidine-2(3*H*)-thione 5,5-dioxide (2f)

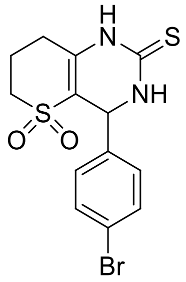

Yield: 260 mg (70%), m.p. 109-112°C, light brown powder. ^{1}H NMR (DMSO-d_6, 500 MHz), δ, ppm (*J*, Hz): 10.55 (1H, s, NH), 9.76 (1H, s, NH), 7.59 (2H, d, *J*=8.4 Hz, H^{Ar}), 7.24 (2H, d, *J*=8.4 Hz, H^{Ar}), 5.23 (1H, s, CH), 3.29 (2H, m, CH_2), 2.50 (2H, m, CH_2), 2.20 (2H, m, CH_2). ^{13}C NMR (DMSO-d_6, 126 MHz), *δ*, ppm: 174.26 (C=S), 141.13, 140.58, 131.60, 128.99, 121.40, 109.11, 49.96, 49.72, 25.17, 18.15. HRMS (ESI-TOF), *m/z*: found 372.9665, calculated for $C_{13}H_{14}BrN_2O_2S_2$ $[M+H]^+$ 372.9675.

4-(Furan-2-yl)-4,6,7,8-tetrahydro-1*H*-thiopyrano[3,2-*d*]pyrimidin-2(3*H*)-thione 5,5-dioxide (2g)

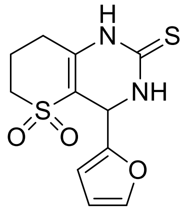

Yield: 193 mg (68%), m.p. 159-163°C, white powder. ^{1}H NMR (DMSO-d_6, 500 MHz), *δ*, ppm (*J*, Hz): 10.56 (1H, s, NH), 9.71 (1H, s, NH), 7.64 (1H, s, H^{Ar}), 6.41 (1H, s, H^{Ar}), 6.30 (1H, s, H^{Ar}), 5.27 (1H, s, CH), 3.28 (2H, m, CH_2), 2.50 (2H, m, CH_2), 2.20 (2H, m, CH_2). ^{13}C NMR (DMSO-d_6, 126 MHz), *δ*, ppm: 174.69 (C=S), 152.99, 143.28, 140.92, 110.56, 107.51, 107.46, 49.96, 43.64, 25.15, 18.18. HRMS (ESI-TOF), *m/z*: found 285.0360, calculated for $C_{11}H_{13}N_2O_3S_2$ [M+H]$^+$ 285.0362.

4-Phenyl-4,6,7,8-tetrahydro-1*H*-thiopyrano[3,2-*d*]pyrimidin-2(3*H*)-one-5,5-dioxide (2h)

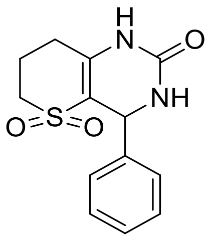

Yield: **A** 234 mg (84%), **B** 264 mg (95%), m.p. 176-178°C, white powder. ^{1}H NMR (DMSO-d_6, 500 MHz), δ, ppm (*J*, Hz): 9.26 (1H, s, NH), 7.87 (1H, s, NH), 7.26-7.37 (5H, m, H^{Ar}), 5.20 (1H, s, CH), 3.22 (2H, m, CH_2), 2.44 (2H, m, CH_2), 2.19 (2H, m, CH_2). ^{13}C NMR (DMSO-d_6, 126 MHz), *δ*, ppm: 151.82 (C=O), 143.26, 143.08, 128.45, 127.69, 126.55, 107.94, 50.36, 50.14, 25.50, 18.34. HRMS (ESI-TOF), *m/z*: found 279.0791, calculated for $C_{13}H_{15}N_2O_3S$ [M+H]$^+$ 279.0798.

4-(Benzo[*d*][1,3]dioxol-5-yl)-4,6,7,8-tetrahydro-1*H*-thiopyrano[3,2-*d*]pyrimidin-2(3*H*)-one 5,5-dioxide (2i)

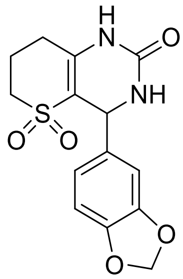

Yield: 193 mg (60%), m.p. 138-141°C, white powder. ^{1}H NMR (DMSO-d_6, 500 MHz), *δ*, ppm (*J*, Hz): 9.23 (1H, s, NH), 7.79 (1H, s, NH), 6.87 (1H, d, *J*=8.0 Hz, H^{Ar}), 6.82 (1H, s, H^{Ar}), 6.77 (1H, d, *J*=8.0 Hz, H^{Ar}), 6.00 (2H, s, CH_2), 5.13 (1H, s, CH), 3.22 (2H, m, CH_2), 2.44 (2H, s, CH_2), 2.19 (2H, m, CH_2). ^{13}C NMR (DMSO-d_6, 126 MHz), *δ*, ppm: 151.68 (C=O), 147.27, 146.74, 143.26, 137.09, 119.96, 108.02, 107.96, 106.97, 101.04, 50.14, 50.10, 25.49, 18.35. HRMS (ESI-TOF), *m/z*: found 323.0687, calculated for $C_{14}H_{15}N_2O_5S$ [M+H]$^+$ 323.0696.

4-(4-Fluorophenyl)-4,6,7,8-tetrahydro-1*H*-thiopyrano[3,2-*d*]pyrimidin-2(3*H*)-one 5,5-dioxide (2j).

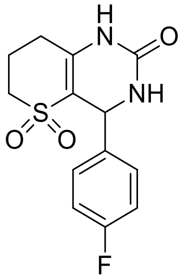

Yield: **A** 207 mg (70%), **B** 157 mg (53%), m.p.162-165°C, white powder. ^{1}H NMR (DMSO-d_6, 500 MHz), δ, ppm (*J*, Hz): 9.28 (1H, s, NH), 7.87 (1H, s, NH), 7.34 (2H, dd, *J*=8.5 Hz, *J*=5.6 Hz, H^{Ar}), 7.18 (2H, t, *J*=8.8 Hz, H^{Ar}), 5.23 (1H, s, CH), 3.23 (2H, m, CH_2), 2.44 (2H, m, CH_2), 2.19 (2H, m, CH_2). ^{13}C NMR (DMSO-d_6, 126 MHz), *δ*, ppm: 161.65 (d, $^1J_{C\text{-}F}$=243.1 Hz), 151.66 (C=O), 143.38, 139.38, 128.71 (d, $^3J_{C\text{-}F}$=8.4 Hz), 115.22 (d, $^2J_{C\text{-}F}$=21.6 Hz), 107.81, 50.13, 49.78, 25.49, 18.35. HRMS (ESI-TOF), *m/z*: found 297.0698, calculated for $C_{13}H_{14}FN_2O_3S$ $[M+H]^+$ 297.0704.

4-(4-Methoxyphenyl)-4,6,7,8-tetrahydro-1*H*-thiopyrano[3,2-*d*]pyrimidin-2(3*H*)-one 5,5-dioxide (2k)

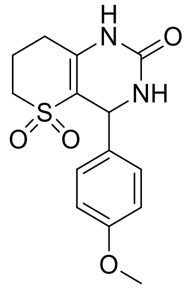

Yield: **A** 206 mg (67%), **B** 200 mg (65%), m.p. 134-136°C, white powder. ^{1}H NMR (DMSO-d_6, 500 MHz), *δ*, ppm (*J*, Hz): 9.21 (1H, s, NH), 7.78 (1H, s, NH), 7.22 (2H, d, *J*=8.5 Hz, H^{Ar}), 6.90 (2H, d, *J*=8.5 Hz, H^{Ar}), 5.17 (1H, s, CH), 3.74 (3H, s, CH_3), 3.21 (2H, m, CH_2), 2.43 (2H, m, CH_2), 2.18 (2H, m, CH_2). ^{13}C NMR (DMSO-d_6, 126 MHz), *δ*, ppm: 158.79, 151.80 (C=O), 142.92, 135.29, 127.81, 113.77, 108.23, 55.11, 50.18, 49.84, 25.50, 18.37. HRMS (ESI-TOF), *m/z*: found 309.0894, calculated for $C_{14}H_{17}N_2O_4S$ $[M+H]^+$ 309.0904.

4-(4-Nitrophenyl)-4,6,7,8-tetrahydro-1*H*-thiopyrano[3,2-*d*]pyrimidin-2(3*H*)-one 5,5-dioxide (2l).

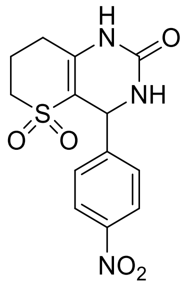

Yield: **A** 191 mg (59%), m.p. 154-159°C, yellow powder. ^{1}H NMR (DMSO-d_6, 500 MHz), δ, ppm (J, Hz): 9.40 (1H, s, NH), 8.23 (1H, d, J=8.3 Hz, H^{Ar}), 8.00 (1H, s, NH), 7.59 (2H, d, J=8.3 Hz, H^{Ar}), 5.37 (1H, s, CH), 3.25 (2H, m, CH_2), 2.46 (2H, m, CH_2), 2.20 (2H, m, CH_2). ^{13}C NMR (DMSO-d_6, 126 MHz), δ, ppm: 151.44 (C=O), 150.18, 147.07, 144.07, 128.10, 123.85, 107.01, 50.09, 25.54, 21.07, 18.35. HRMS (ESI-TOF), *m/z*: found 324.0640, calculated for $C_{13}H_{14}N_3O_5S$ $[M+H]^+$ 324.0649.

4-(4-Bromophenyl)-4,6,7,8-tetrahydro-1*H*-thiopyrano[3,2-*d*]pyrimidin-2(3*H*)-one 5,5-dioxide (2m)

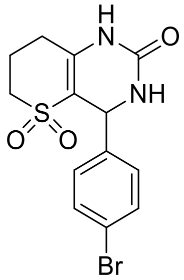

Yield: **A** 278 mg (78%), m.p. 135-138°C, light brown powder. ^{1}H NMR (DMSO-d_6, 500 MHz), δ, ppm (J, Hz): 9.30 (1H, s, NH), 7.89 (1H, s, NH), 7.56 (2H, d, J=8.1 Hz, H^{Ar}), 7.26 (2H, d, J=8.1 Hz, H^{Ar}), 5.20 (1H, s, CH), 3.23 (2H, m, CH_2), 2.44 (2H, m, CH_2), 2.19 (2H, m, CH_2). ^{13}C NMR (DMSO-d_6, 126 MHz), δ, ppm: 151.59 (C=O), 143.51, 142.48, 131.41, 128.92, 120.91, 107.49, 50.11, 49.98, 25.51, 18.35. HRMS (ESI-TOF), *m/z*: found 356.9896, calculated for $C_{13}H_{14}BrN_2O_3S$ $[M+H]^+$ 356.9903.

4-(Furan-2-yl)-4,6,7,8-tetrahydro-1*H*-thiopyrano[3,2-*d*]pyrimidin-2(3*H*)-one 5,5-dioxide (2n)

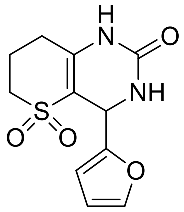

Yield: **A** 182 mg (68%), m.p. 126-129°C, white powder. ^{1}H NMR (DMSO-d_6, 500 MHz), *δ*, ppm (*J*, Hz): 9.30 (1H, s, NH), 7.90 (1H, s, NH), 7.60 (1H, s, H^{Ar}), 6.38 (1H, s, H^{Ar}), 6.25 (1H, s, H^{Ar}), 5.24 (1H, s, CH), 3.23 (2H, m, CH_2), 2.41 (2H, m, CH_2), 2.18 (2H, m, CH_2). ^{13}C NMR (DMSO-d_6, 126 MHz), *δ*, ppm: 154.24, 151.86 (C=O), 143.95, 142.80, 110.46, 106.67, 105.78, 50.09, 43.95, 25.51, 18.38. HRMS (ESI-TOF), *m/z*: found 269.0591, calculated for $C_{11}H_{13}N_2O_4S$ $[M+H]^+$ 269.0591.

4-Phenyl-4,6,7,8-tetrahydro-1*H*-thiopyrano[3,2-*d*]pyrimidine-2(3*H*)-selenone-5,5-dioxide (2o)

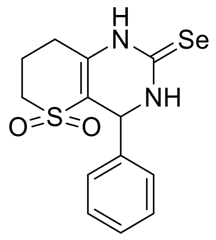

Yield: **A** 113 mg (33%), **B** 103 mg (30%), m.p. 174-177°C, pink powder. ^{1}H NMR (DMSO-d_6, 500 MHz), *δ*, ppm (*J*, Hz): 10.85 (1H, s, NH), 10.28 (1H, s, NH), 7.39 (2H, m, H^{Ar}), 7.30 (3H, m, H^{Ar}), 5.26 (1H, s, CH), 3.29 (2H, m, CH_2), 2.56 (2H, m, CH_2), 2.21 (2H, m, CH_2). ^{13}C NMR (DMSO-d_6, 126 MHz), *δ*, ppm: 170.54 (C=Se), 141.35, 139.50, 128.70, 128.24, 126.73, 110.11, 50.28, 49.93, 25.10, 18.07. HRMS (ESI-TOF), *m/z*: found 343.0013, calculated for $C_{13}H_{15}N_2O_2SSe$ $[M+H]^+$ 343.0014.

4-(4-Fluorophenyl)-4,6,7,8-tetrahydro-1*H*-thiopyrano[3,2-*d*]pyrimidine-2(3*H*)-selenone 5,5-dioxide (2p)

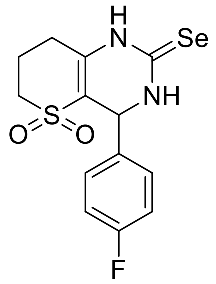

Yield: **A** 61 mg (17%), m.p. 145-148°C, white powder. ^{1}H NMR (DMSO-d_6, 500 MHz), *δ*, ppm (*J*, Hz): 10.89 (1H, s, NH), 10.28 (1H, s, NH), 7.33 (2H, dd, *J*=8.3 Hz, *J*=5.4 Hz, H^{Ar}), 7.22 (2H, t, *J*=8.8 Hz, H^{Ar}), 5.27 (1H, s, CH), 3.30 (2H, m, CH_2), 2.55 (2H, m, CH_2), 2.21 (2H, m, CH_2). ^{13}C NMR (DMSO-d_6, 126 MHz), *δ*, ppm: 170.91 (C=Se), 161.46 (d, $^{1}J_{C\text{-}F}$=250 Hz), 143.49, 140.04, 129.37 (d, $^{3}J_{C\text{-}F}$=8.6 Hz), 115.94 (d, $^{2}J_{C\text{-}F}$=21.6 Hz), 110.39, 50.31, 50.02, 25.51, 18.50. HRMS (ESI-TOF), *m/z*: found 360.9913, calculated for $C_{13}H_{14}FN_2O_2SSe$ $[M+H]^+$ 360.9920.

4-(4-Methoxyphenyl)-4,6,7,8-tetrahydro-1*H*-thiopyrano[3,2-*d*]pyrimidine-2(3*H*)-selenone 5,5-dioxide (2q)

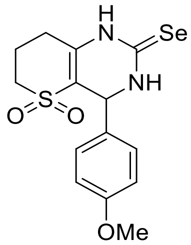

Yield: **A** 74 mg (20%), m.p. 158-161°C, white powder. ^{1}H NMR (DMSO-*d*$_6$, 500 MHz), *δ*, ppm (*J*, Hz): 10.80 (1H, s, NH), 10.22 (1H, s, NH), 7.20 (2H, d, *J*=8.5 Hz, H^{Ar}), 6.93 (2H, d, *J*=8.5 Hz, H^{Ar}), 5.20 (1H, s, CH), 3.75 (3H, s, CH_3), 3.28 (2H, m, CH_2), 2.54 (2H, m, CH_2), 2.21 (2H, m, CH_2). ^{13}C NMR (DMSO-*d*$_6$, 126 MHz), *δ*, ppm: 170.15 (C=Se), 159.14, 139.21, 133.57, 128.04, 113.99, 110.35, 55.15, 49.94, 49.71, 25.05, 18.08. HRMS (ESI-TOF), *m/z*: found 373.0109, calculated for $C_{14}H_{17}N_2O_3SSe$ $[M+H]^+$ 373.0120.

4-(3-Hydroxyphenyl)-3,4,7,8-tetrahydro-1*H*-thiopyrano[3,2-*d*]pyrimidine-2(6*H*)-thione 5,5-dioxide (2r)

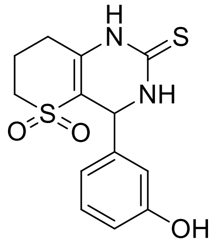

Yield: **A** 1.767 g (57%), **B** 1.891 g (61%) (starting from 10 mmol ketosulfone **1**), m.p. 143-145°C, white powder. ^{1}H NMR (DMSO-*d*$_6$, 500 MHz), *δ*, ppm (*J*, Hz): 10.47 (1H, s, NH), 9.69 (1H, s, NH), 9.50 (1H, s, OH), 7.15 (1H, s, H^{Ar}), 6.69 (3H, m, H^{Ar}), 5.14 (1H, s, CH), 3.26 (2H, m, CH_2), 2.50 (2H, m, CH_2), 2.20 (2H, m, CH_2). ^{13}C NMR (DMSO-*d*$_6$, 126 MHz), *δ*, ppm: 174.23 (C=S), 157.48, 143.29, 140.09, 129.62, 117.29, 115.09, 113.53, 109.75, 50.22, 50.05, 25.21, 18.14. HRMS (ESI-TOF), *m/z*: found 311.0511, calculated for $C_{13}H_{15}N_2O_3S_2$ $[M+H]^+$ 311.0519.

3,5-Diphenyl-5,7,8,9-tetrahydrothiazolo[3,2-*a*]thiopyrano[3,2-*d*]pyrimidine 6,6-dioxide (3)

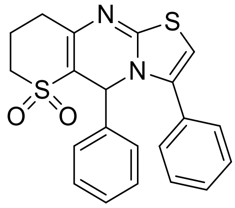

A stirred reaction mixture of 4-phenyl-3,4,7,8-tetrahydro-1*H*-thiopyrano[3,2-*d*]pyrimidine-2(6*H*)-thione 5,5-dioxide (**2a**, 200 mg, 0.68 mmol, 1 equiv) and 2-bromoacetophenone (203 mg, 1.02 mmol, 1.5 equiv) in acetic acid (2 mL) was heated at 120 °C for 5 h. After completion the reaction, solvent was evaporated in vacuo and the semi-crystalline residue was triturated with 5% aq. $NaHCO_3$, water and then recrystallized from isopropyl alcohol. Yield: 185 mg (69%), m.p. 164-167°C, white powder. ^{1}H NMR (500 MHz, DMSO-*d6*), *δ*, ppm (*J*, Hz): 7.56 (1H, t, *J*=7.3 Hz, H^{Ar}), 7.48 (2H, t, *J*=7.3 Hz, H^{Ar}), 7.40 (1H, s, CH), 7.14-7.26 (5H, m, H^{Ar}), 6.67 (2H, d, *J*=7.3 Hz, H^{Ar}), 6.37 (1H, s, CH), 3.42 (1H, d, *J*=12.6 Hz, CH), 3.23 (1H, t, *J*=12.6 Hz, CH), 2.77 (1H, d, *J*=18.3 Hz, CH), 2.66 (1H, m, CH), 2.31 (1H, m, CH), 2.21 (1H, m, CH). ^{13}C NMR (DMSO-*d*6, 126 MHz), *δ*, ppm: 139.48, 138.30, 130.51, 129.34, 129.23, 128.90, 128.75, 127.29, 126.39, 111.22, 55.16, 49.88, 25.99, 18.04. HRMS (ESI-TOF), *m/z*: found 395.0876, calculated for $C_{21}H_{19}N_2O_2S_2$ $[M+H]^+$ 395.0882.

3-Amino-5-phenyl-5,7,8,9-tetrahydrothiazolo[3,2-*a*]thiopyrano[3,2-*d*]pyrimidine-2-carbonitrile 6,6-dioxide (4)

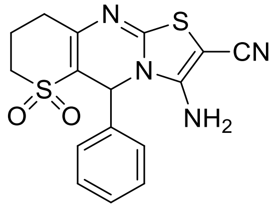

To a stirred reaction mixture of 4-phenyl-3,4,7,8-tetrahydro-1*H*-thiopyrano[3,2-d]pyrimidine-2(6*H*)-thione 5,5-dioxide (**2a**, 200 mg, 0.68 mmol, 1 equiv) and potassium hydroxide (38 mg, 0.68 mmol, 1 equiv) in ethanol (3 mL) bromomalononitrile (99 mg, 0.68 mmol, 1 equiv) was added in portions. The reaction mixture was heated at 82 °C for 10 h. After completion the reaction, the inorganic solid was filtrated, the filtrate was evaporated in vacuo and the semi-crystalline residue was recrystallized with isopropyl alcohol. Yield: 163 mg (67%), m.p. 155-159°C, yellow powder. ^{1}H NMR (500 MHz, DMSO-*d*6), *δ*, ppm (*J*, Hz): 7.84 (2H, br.s, NH_2), 7.38-7.30 (5H, m, H^{Ar}), 4.49 (1H, s, CH), 3.24 (2H, m, CH_2), 2.54 (2H, m, CH_2), 2.17 (2H, m, CH_2). ^{13}C NMR (DMSO-*d*6, 126 MHz), *δ*, ppm: 143.07, 129.10, 128.89, 126.59, 126.56, 116.47, 62.05, 51.56, 50.15, 25.49, 18.37. HRMS (ESI-TOF), *m/z*: found 359.0631, calculated for $C_{16}H_{15}N_4O_2S_2$ $[M+H]^+$ 359.0631.

13-Phenyl-5,6,9,10,11,13-hexahydronaphtho[1',2':4,5]thiazolo[3,2-*a*]thiopyrano[3,2-*d*]pyrimidine 12,12-dioxide (5)

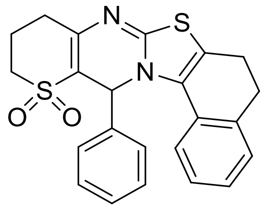

A stirred reaction mixture of 4-phenyl-3,4,7,8-tetrahydro-1*H*-thiopyrano[3,2-d]pyrimidine-2(6*H*)-thione 5,5-dioxide **2a** (200 mg, 0.68 mmol, 1 equiv) and 2-bromo-1-tetralone (230 mg, 1.02 mmol, 1.5 equiv) in acetic acid (2 mL) was heated at 120 ^{o}C for 6 h. After completion the reaction, solvent was evaporated *in vacuo* and the semi-crystalline residue was triturated with 5% aq. $NaHCO_3$, water and then recrystallized from isopropyl alcohol. Yield: 251 mg (88%), m.p. 158-162°C, light-yellow powder. ^{1}H NMR (500 MHz, DMSO-d_6), *δ*, ppm (*J*, Hz): 7.03-7.49 (9H, m, H^{Ar}), 4.72 (3H, m, CH+CH_2), 3.49 (1H, m, CH), 3.31 (1H, m, CH), 2.89 (1H, m, CH), 2.68 (3H, m, CH+CH_2), 2.32 (2H, m, CH_2). ^{13}C NMR (DMSO-d_6, 126 MHz), *δ*, ppm: 137.40, 136.56, 131.97, 129.39, 129.13, 129.07, 128.95, 128.91, 127.48, 126.66, 125.80, 124.61, 122.24, 110.96, 62.00, 54.04, 49.94, 28.63, 26.59, 21.82, 18.15. HRMS (ESI-TOF), *m/z*: found 421.1027, calculated for $C_{23}H_{21}N_2O_2S_2$ $[M+H]^+$ 421.1039.

5-Phenyl-5,8,9,10-tetrahydro-7*H*-tetrazolo[1,5-*a*]thiopyrano[3,2-*d*]pyrimidine 6,6-dioxide (6)

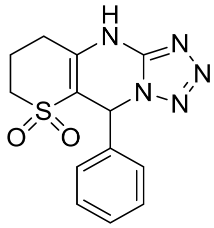

A stirred reaction mixture of 4-phenyl-3,4,7,8-tetrahydro-1*H*-thiopyrano[3,2-*d*]pyrimidine-2(6*H*)-thione 5,5-dioxide (**2a**, 200 mg, 0.68 mmol, 1 equiv), mercury(II) acetate (217 mg, 0.68 mmol, 1 equiv), and sodium azide (88 mg, 1.36 mmol, 2 equiv) in acetic acid (3 mL) was heated at 120 ^{o}C for 6 h. After completion the reaction, solvent was evaporated in vacuo and the semi-crystalline residue was triturated with 5% aq. $NaHCO_3$, water and then recrystallized from isopropyl alcohol. Yield: 120 mg (58%), m.p. 219-222°C, light-yellow powder. ^{1}H NMR (500 MHz, DMSO-d_6), δ, ppm (*J*, Hz): 11.37 (1H, br.s, NH), 7.36 (5H, m, H^{Ar}), 6.78 (1H, s, CH), 3.21-3.30 (2H, m, CH_2), 2.63-2.76 (2H, m, CH_2), 2.28 (2H, m, CH_2). ^{13}C NMR (126 MHz, DMSO-d_6), ppm: 148.30, 141.84, 138.66, 128.96, 128.64, 127.53, 106.42, 55.77, 49.88, 25.99, 17.94. The spectroscopic data are in agreement with those reported in the literature [Dil, K. V.; Okovytyy,

S. I.; Palchykov, V. A. *J. Chem. Technol.* **2023**, *31,* 411–418. https://doi.org/10.15421/jchemtech.v31i2.126304]

4-Phenyl-4,6,7,8-tetrahydro-1*H*-thiopyrano[3,2-*d*]pyrimidine 5,5-dioxide (7)

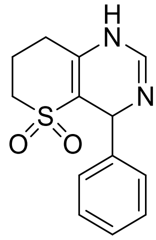

To a stirred reaction mixture of 4-phenyl-3,4,7,8-tetrahydro-1*H*-thiopyrano[3,2-*d*]pyrimidine-2(6*H*)-thione 5,5-dioxide (**2a**, 200 mg, 0.68 mmol, 1 equiv) and vanadyl(IV) sulfate $VO(SO_4)\cdot 2H_2O$ (13.5 mg, 0.068 mmol, 0.1 equiv) in ethanol-water (8 mL-0.8 mL, 10:1) hydrogen peroxide solution (50% aq., 231 mg, 193 µL 3.4 mmol, 5 equiv) was added. The reaction mixture was heated at 50 ^{o}C for 18 h. After completion the reaction was evaporated in vacuo and the semi-crystalline residue was purified on silica using ethyl acetate as eluent. Yield: 48 mg (27%), m.p. 128-131°C, R_f (ethyl acetate) 0.15, white powder. ^{1}H NMR (500 MHz, DMSO-d_6), δ, ppm (*J*, Hz): 9.25 (1H, s, NH), 7.86 (1H, s, N=CH), 7.37-7.26 (5H, m, H^{Ar}), 5.21 (1H, d, *J*=2.9 Hz, CH), 3.22 (2H, m, CH_2), 2.46 (2H, m, CH_2), 2.19 (2H, m, CH_2). ^{13}C NMR (126 MHz, DMSO-d_6), ppm: 151.83, 143.28, 128.46, 127.71, 126.57, 121.11, 107.94, 50.36, 50.15, 25.50, 18.34. HRMS (ESI-TOF), *m/z*: found 263.0842, calculated for $C_{13}H_{15}N_2O_2S$ [M+H]$^+$ 263.0849.

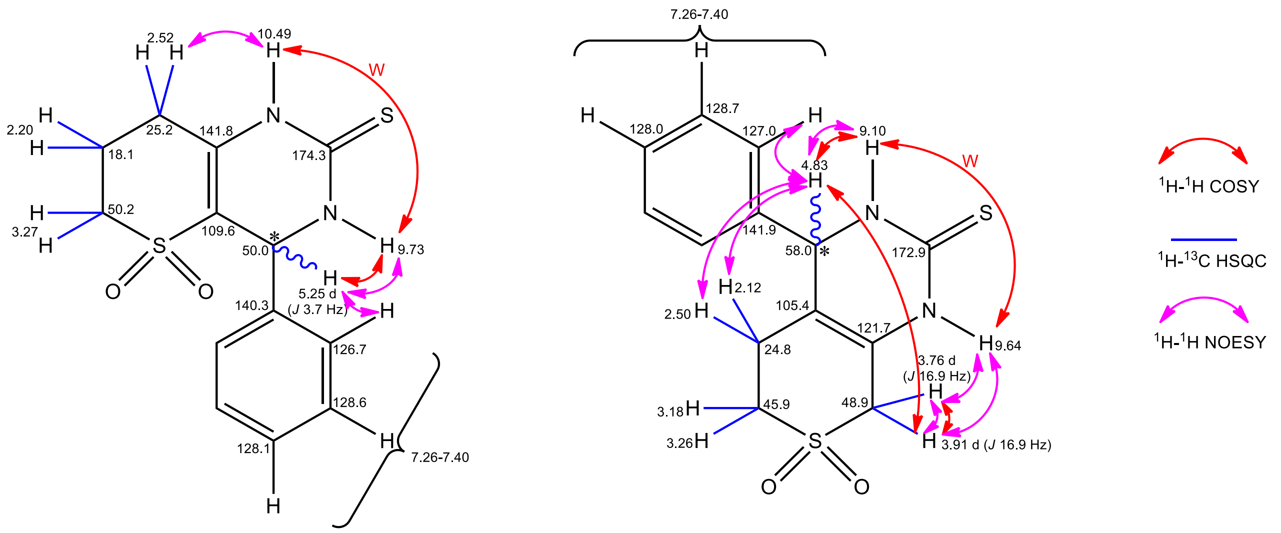

2a (major isomer) **2a' (minor isomer)**

Figure S1: NMR chemical shifts and important 2D correlations for the corresponding regioisomers **2a:2a'** (ratio 2:1) (400 MHz, DMSO-d_6, ppm). This sample was obtained from the reaction mixture entry 6 (Table 1, see article).

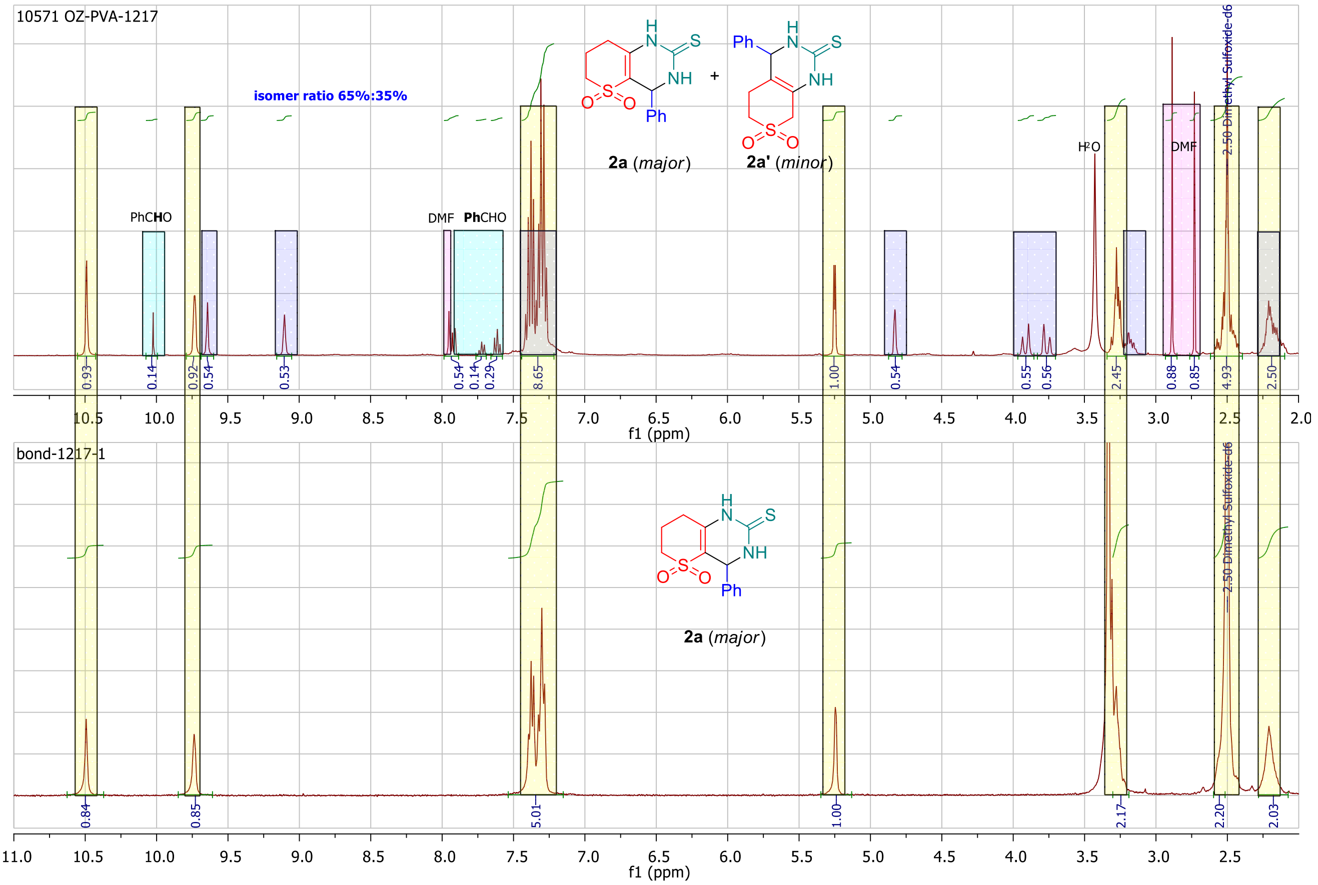

Figure S2: ^{1}H NMR spectrum for regioisomers **2a:2a'** (*up*) and for major isomer **2a** (*bottom*) (400 MHz, DMSO-d_6, ppm).

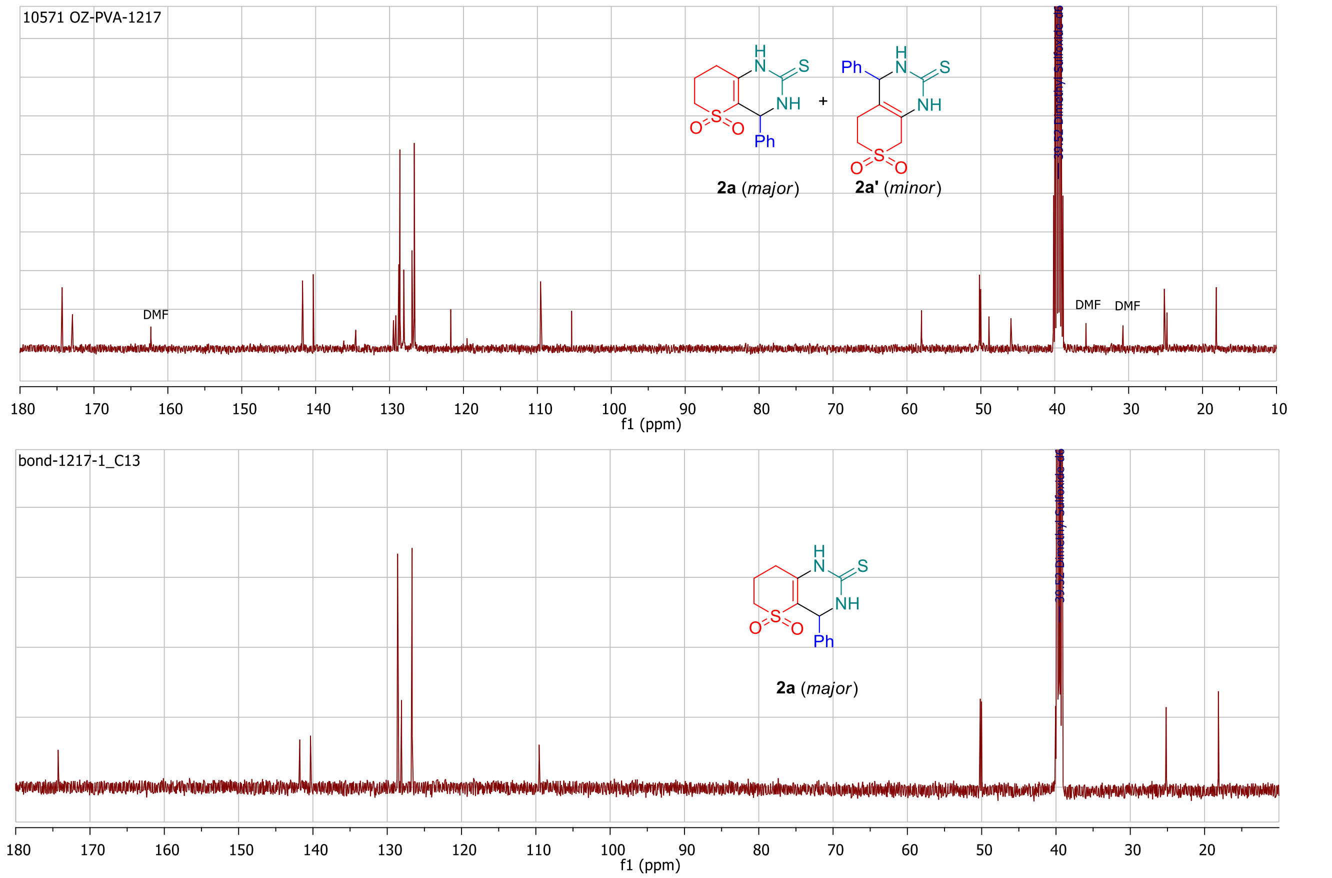

Figure S3: ^{13}C NMR spectrum for regioisomers **2a:2a'** (*up*) and for major isomer **2a** (*bottom*) (101 MHz, DMSO-*d*6, ppm).

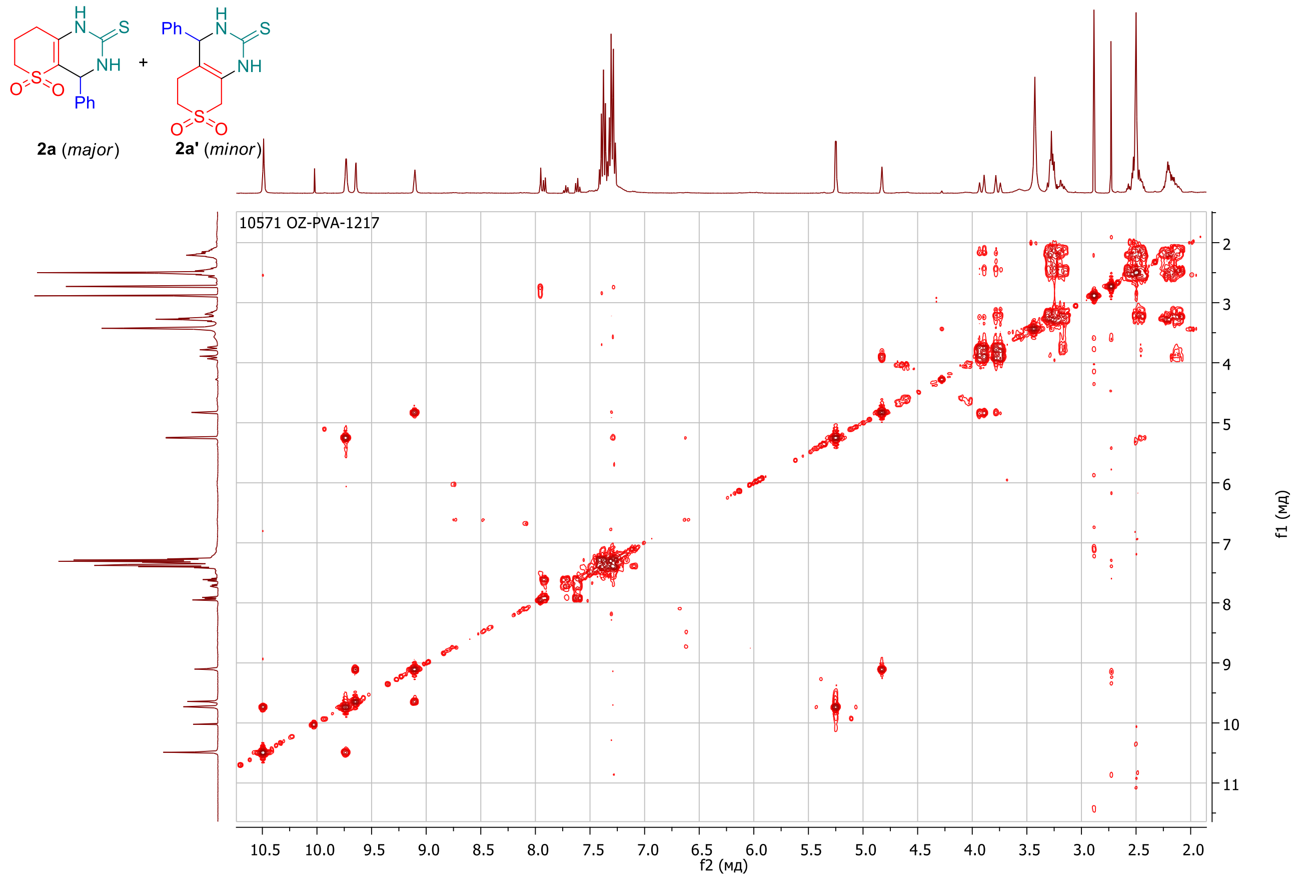

Figure S4: COSY spectrum for regioisomers **2a:2a'** (400 MHz, DMSO-*d*6, ppm).

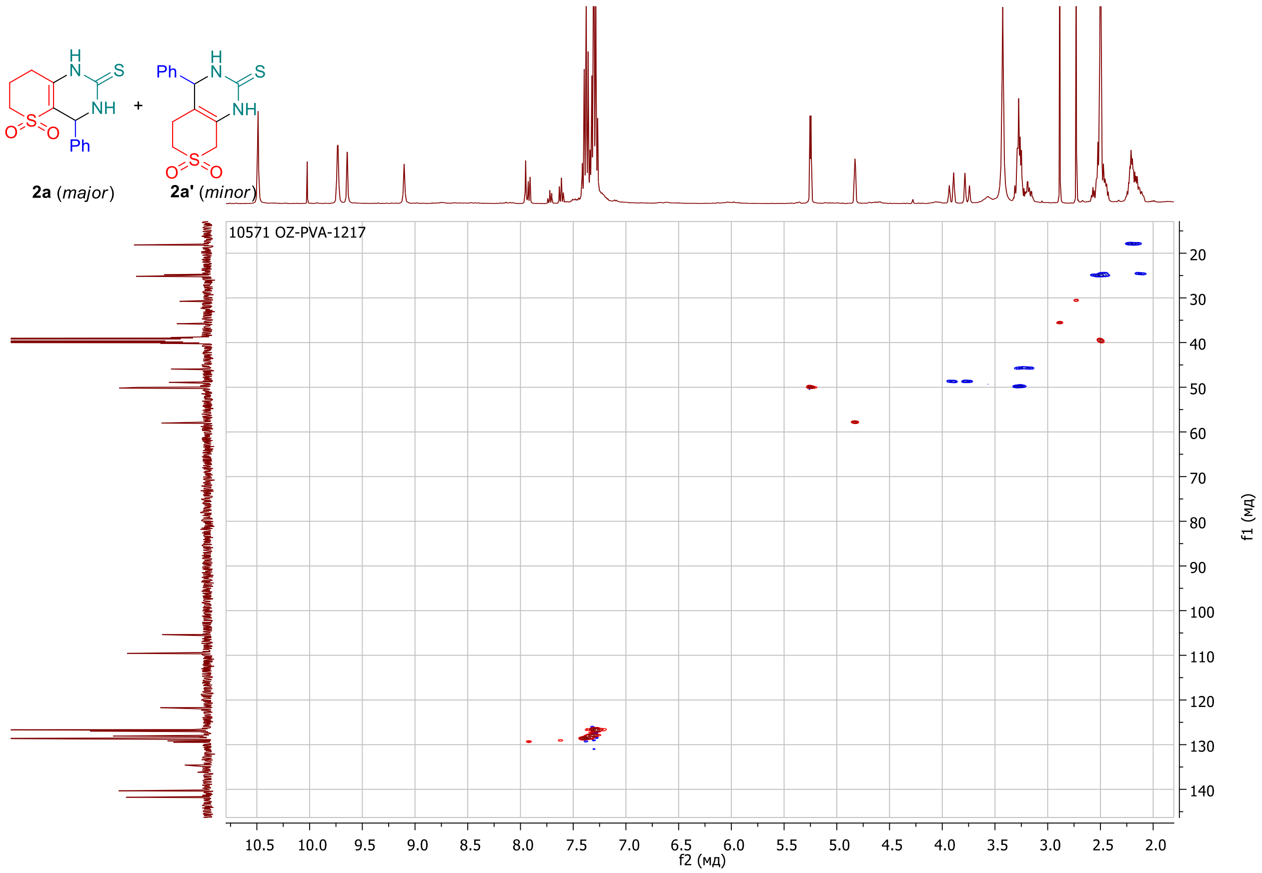

Figure S5: HSQC spectrum for regioisomers **2a:2a'** (400/101 MHz, DMSO-d_6, ppm).

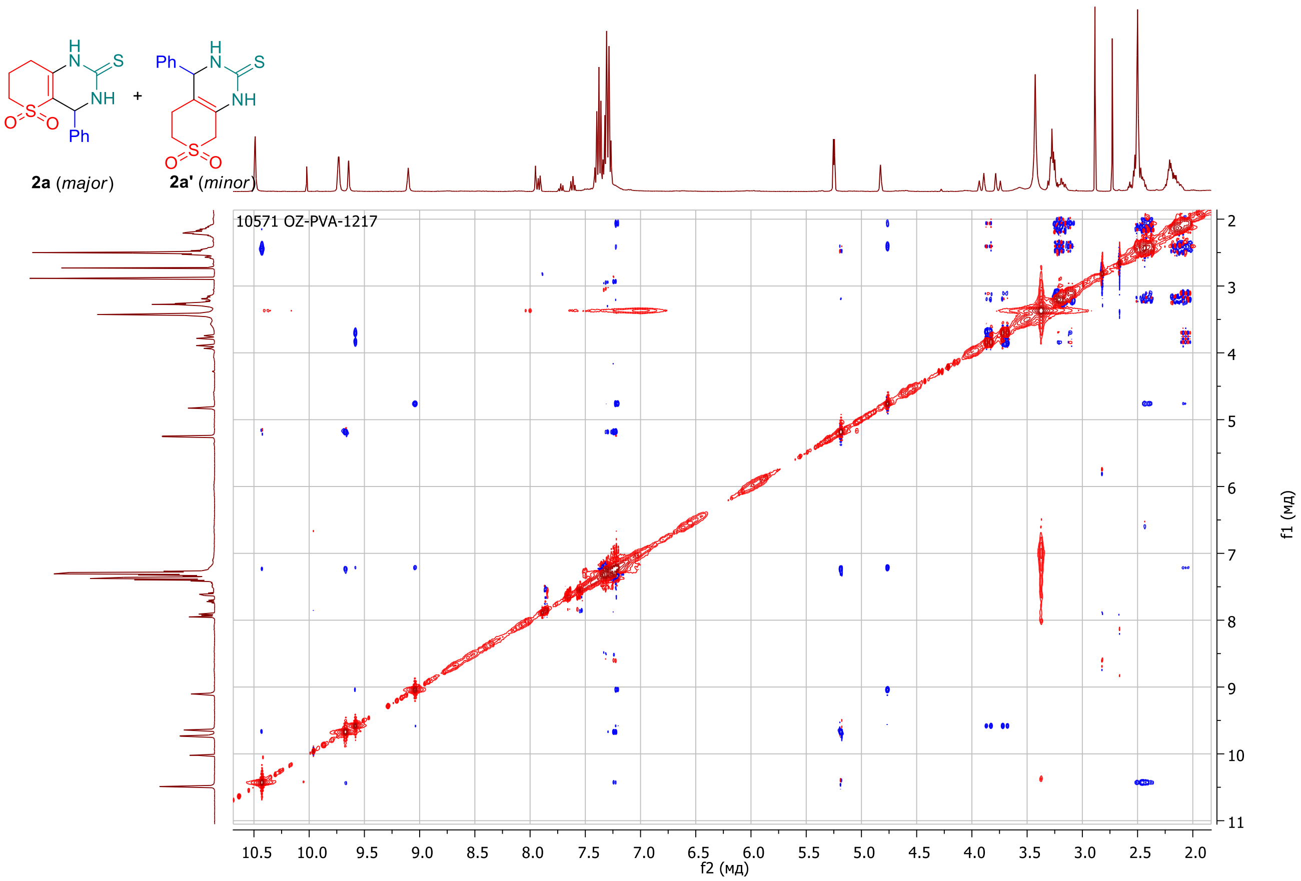

Figure S6: NOESY spectrum for regioisomers **2a:2a'** (400 MHz, DMSO-d_6, ppm).

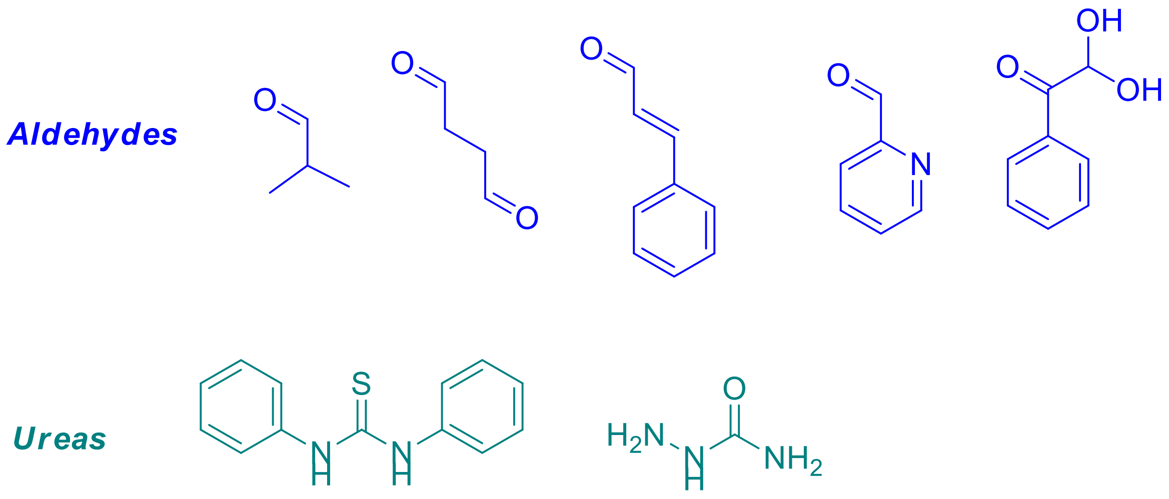

Figure S7: The list of unsuccessful starting reagents.

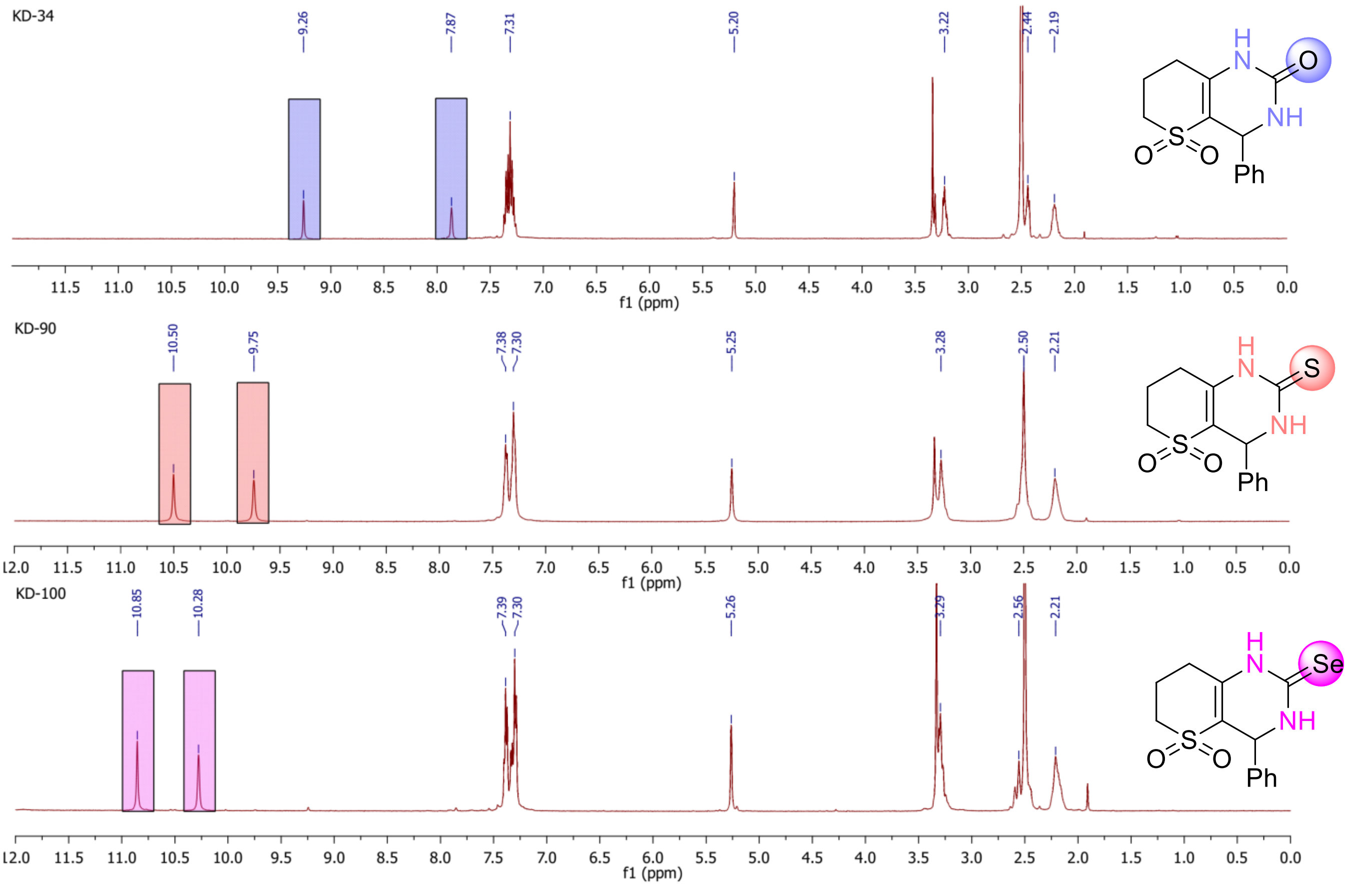

Figure S8: Comparison of ^{1}H NMR spectra (500 MHz, DMSO-d_6) for Ph-substituted O,S,Se-DHPMs **2a,h,o**

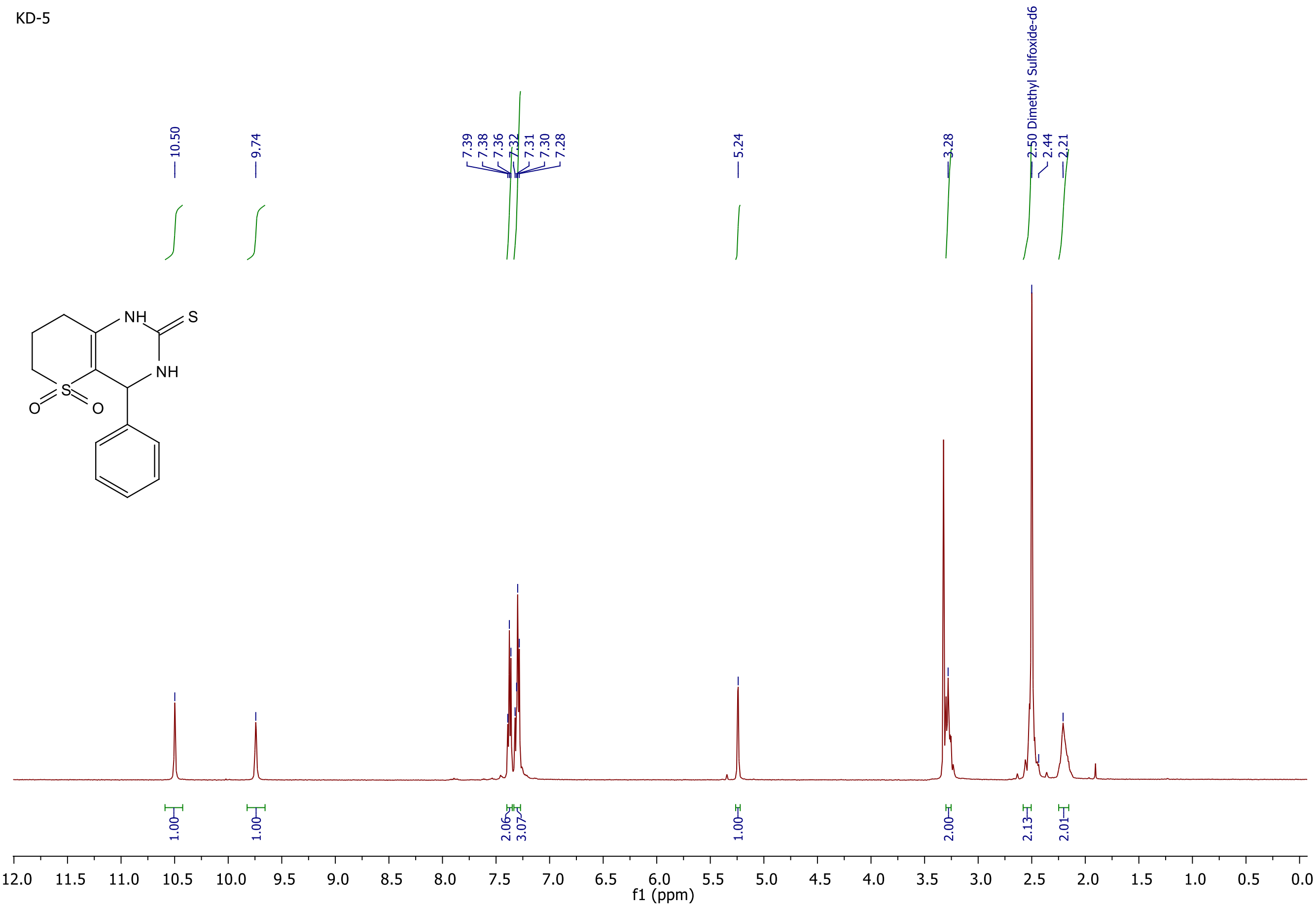

Figure S9: ^{1}H NMR spectrum of **2a** (500 MHz, DMSO-d_6)

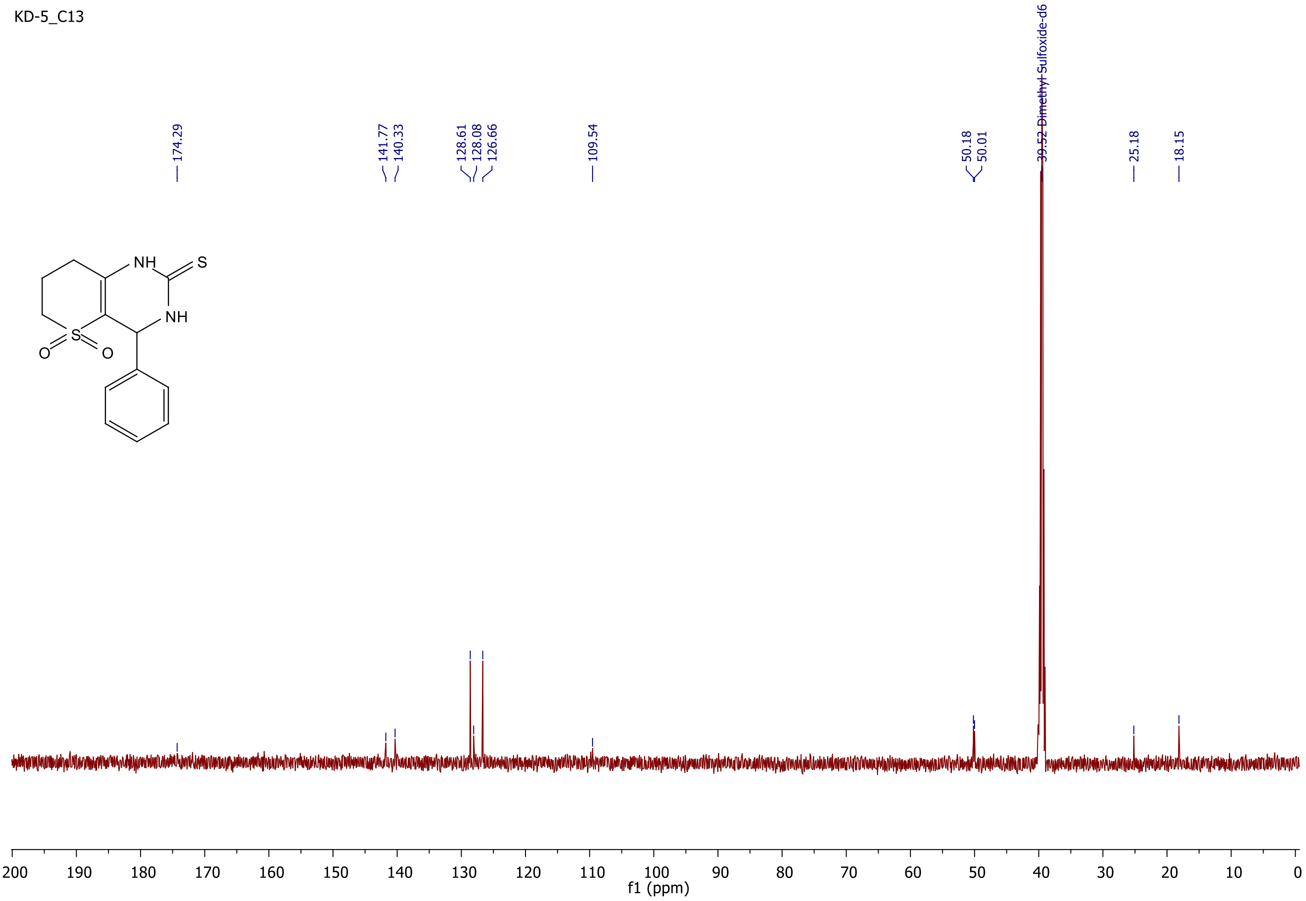

Figure S10: ^{13}C NMR spectrum of **2a** (126 MHz, DMSO-d_6)

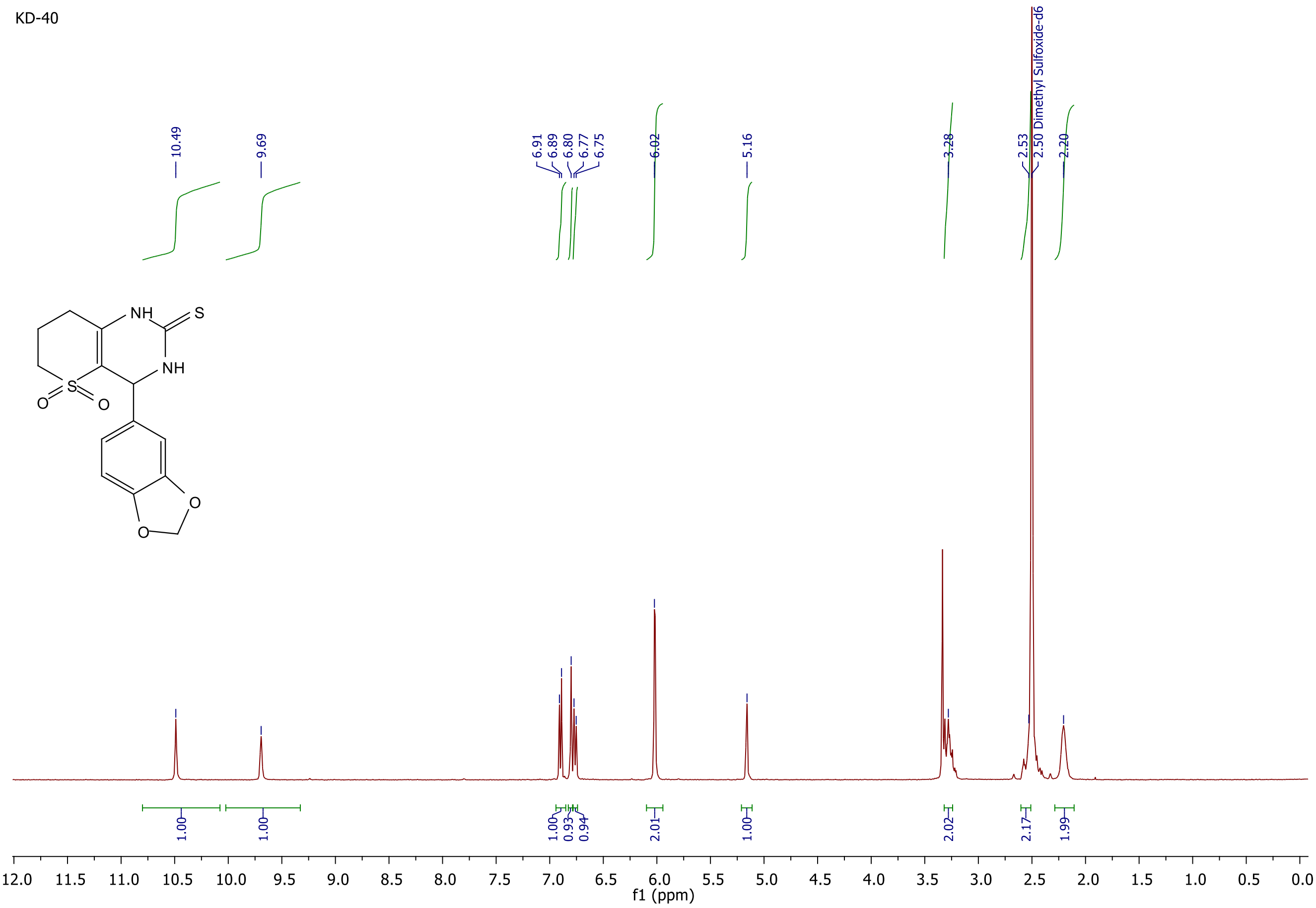

Figure S11: ^{1}H NMR spectrum of **2b** (500 MHz, DMSO-d_6)

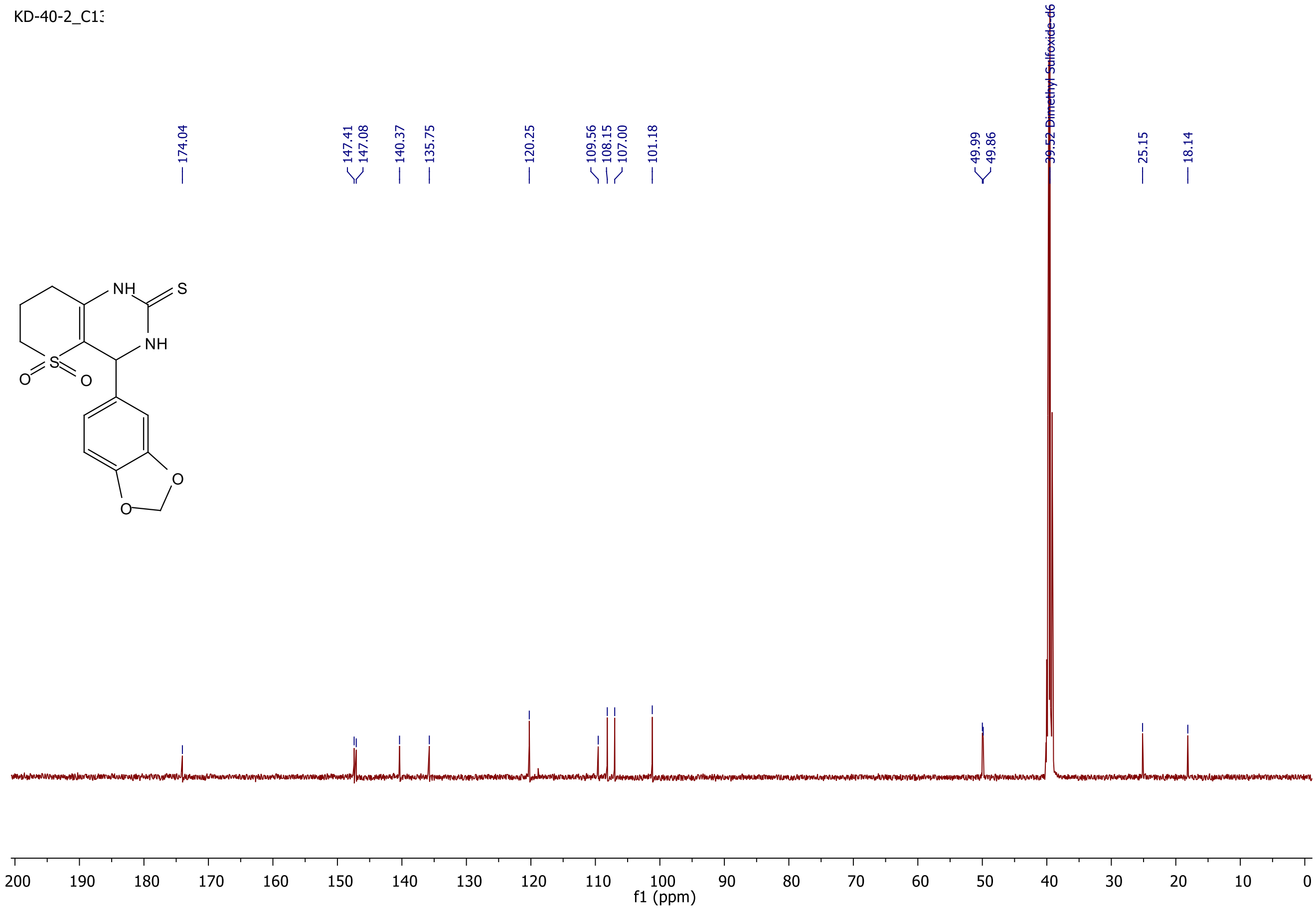

Figure S12: ^{13}C NMR spectrum of **2b** (126 MHz, DMSO-d_6)

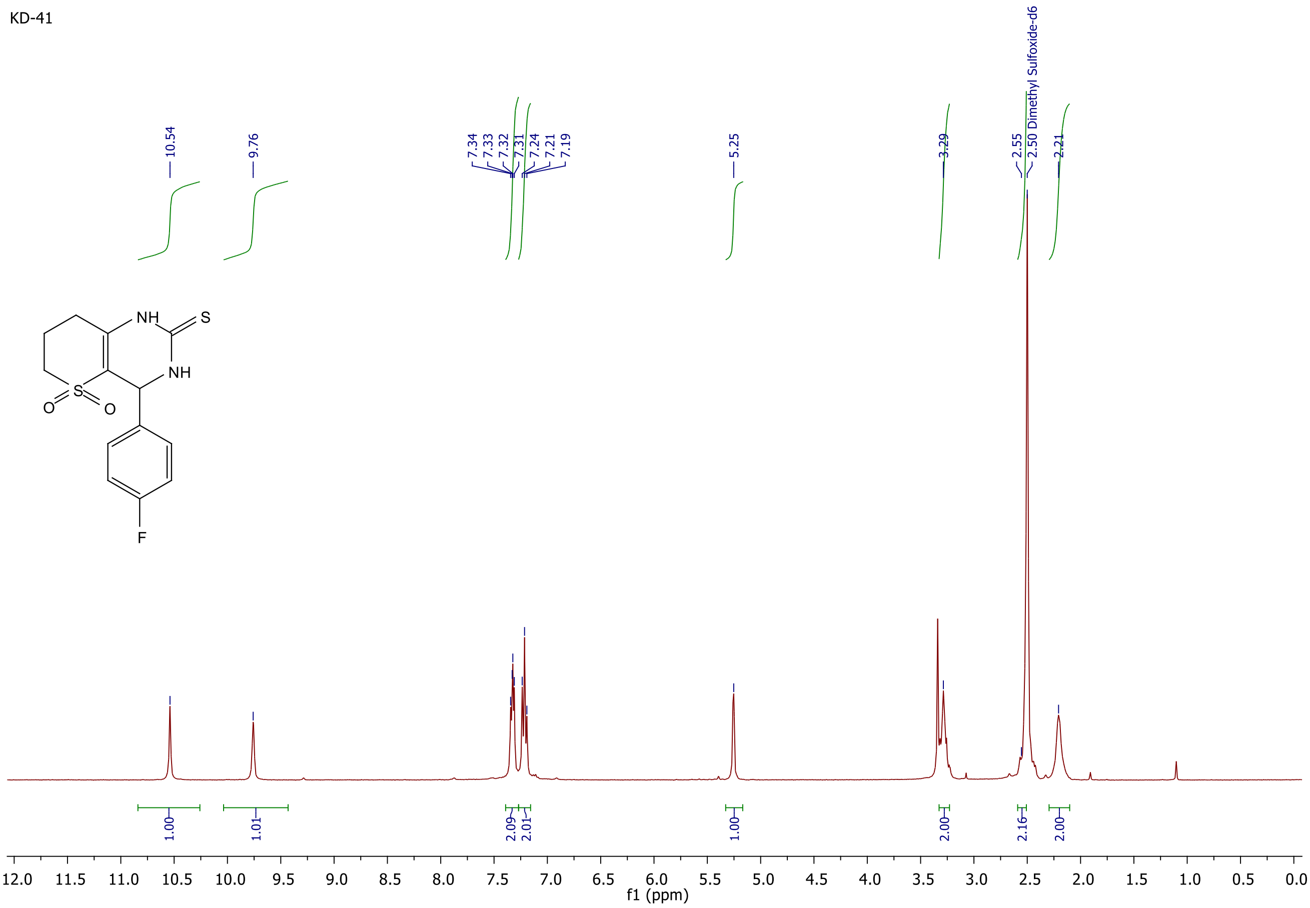

Figure S13: ^{1}H NMR spectrum of **2c** (500 MHz, DMSO-d_6)

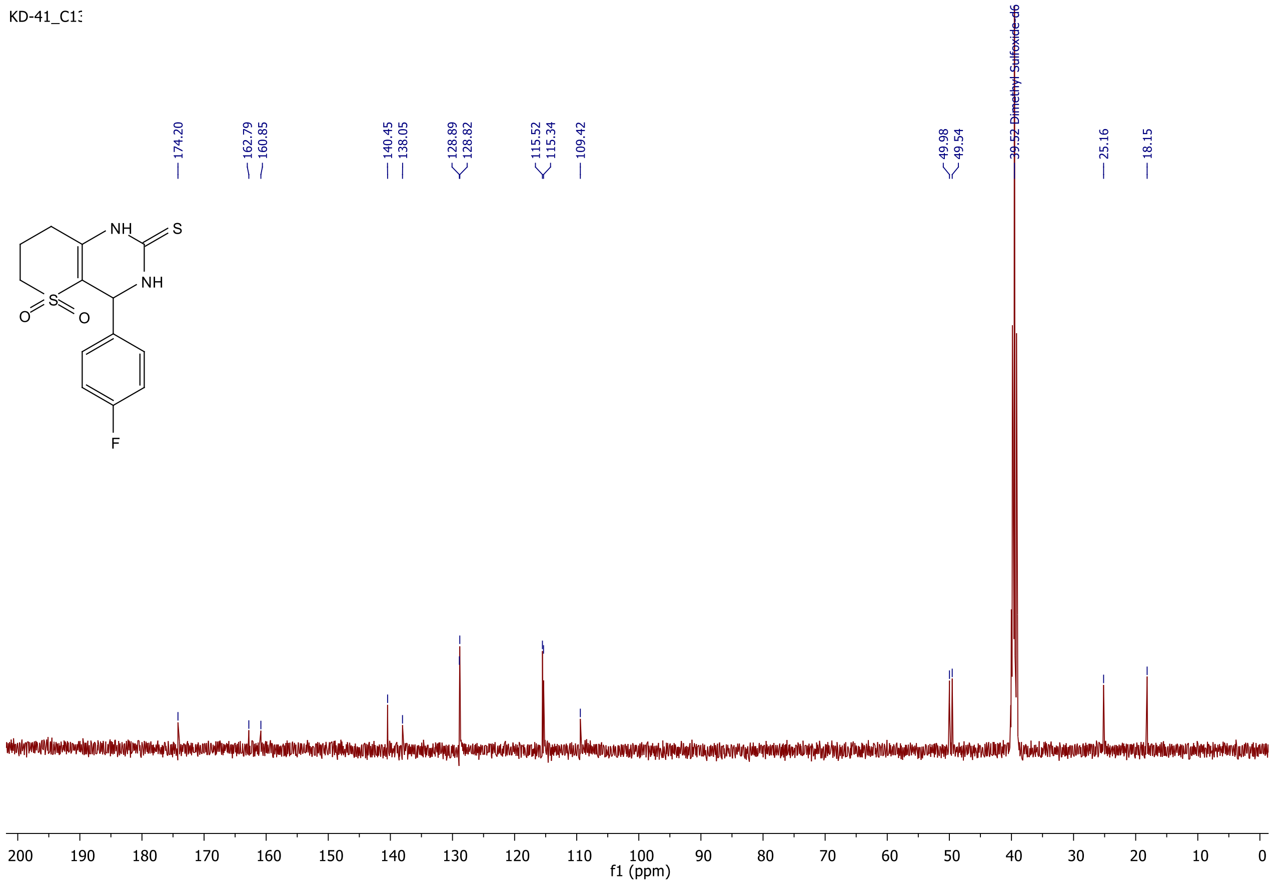

Figure S14: ^{13}C NMR spectrum of **2c** (126 MHz, DMSO-d_6)

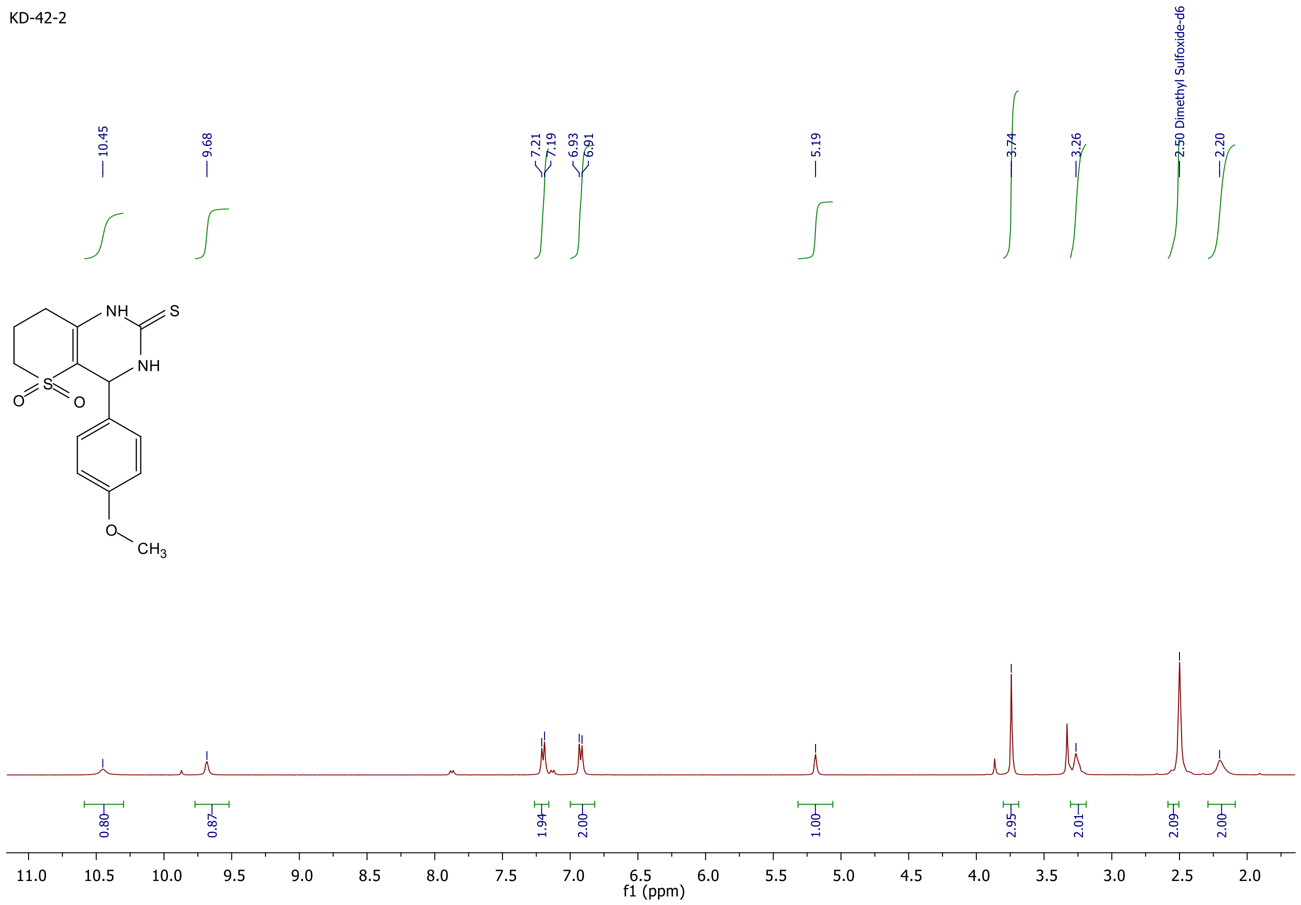

Figure S15: ^{1}H NMR spectrum of **2d** (500 MHz, DMSO-d_6)

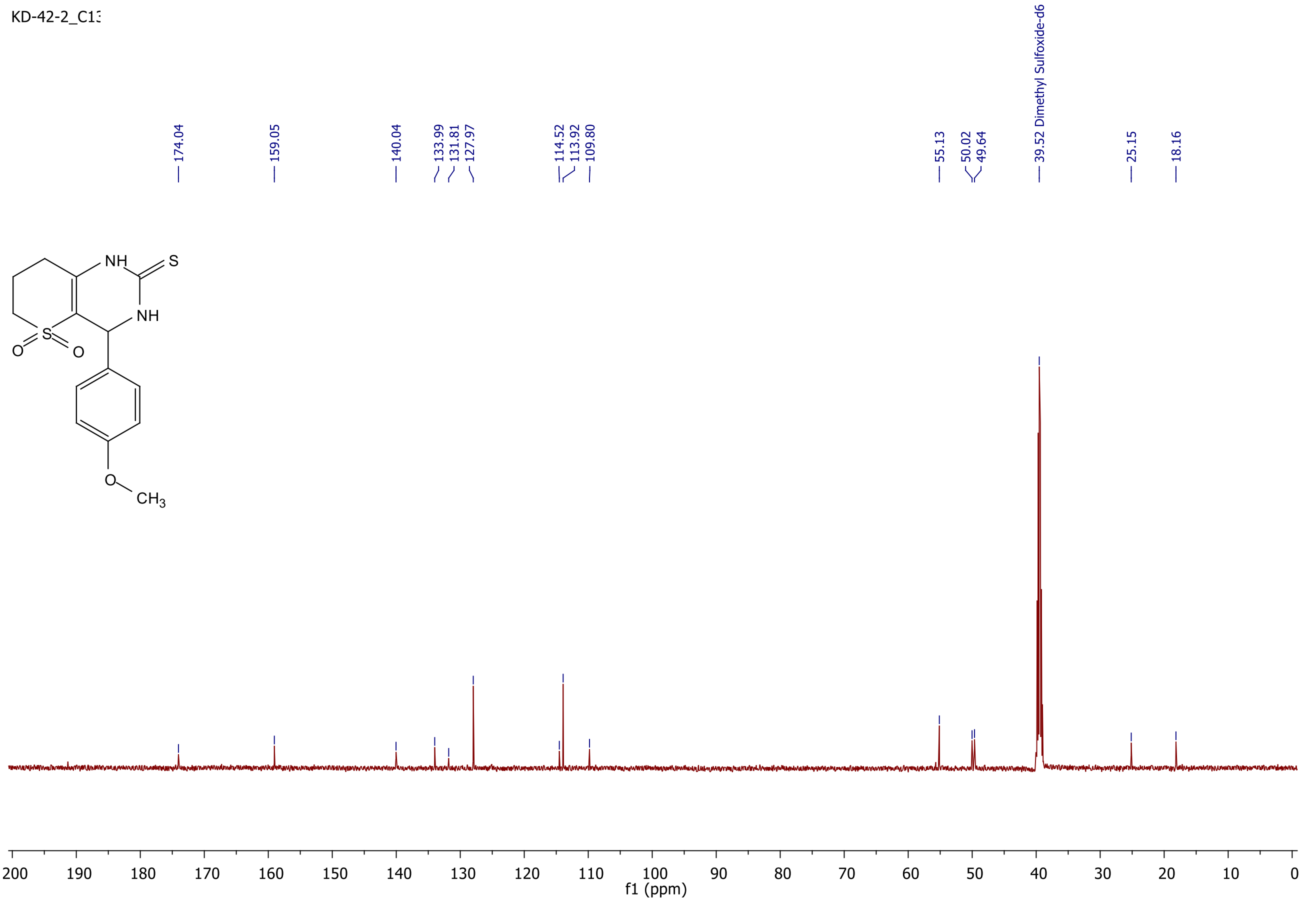

Figure S16: ^{13}C NMR spectrum of **2d** (126 MHz, DMSO-d_6)

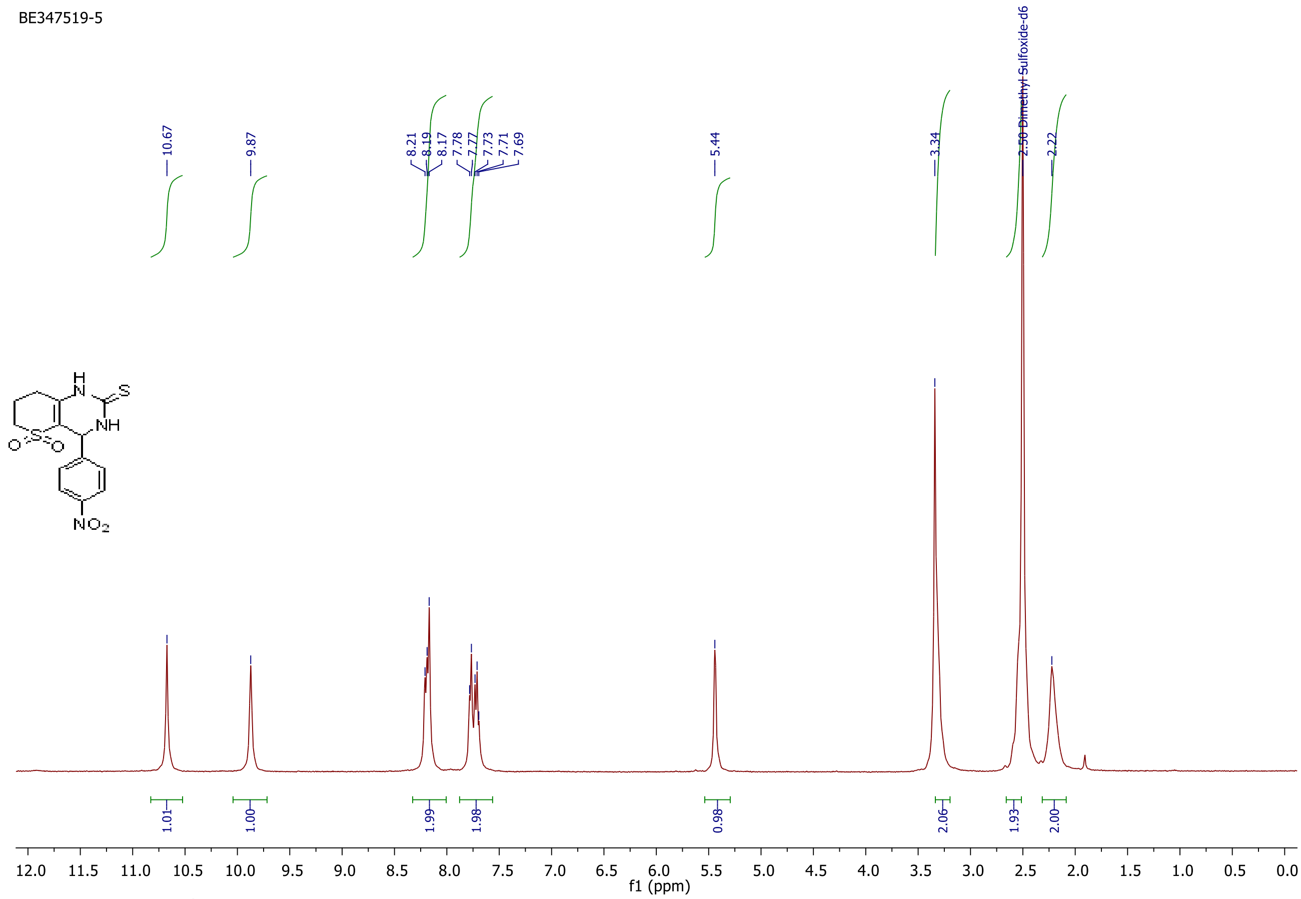

Figure S17: ^{1}H NMR spectrum of **2e** (500 MHz, DMSO-d_6)

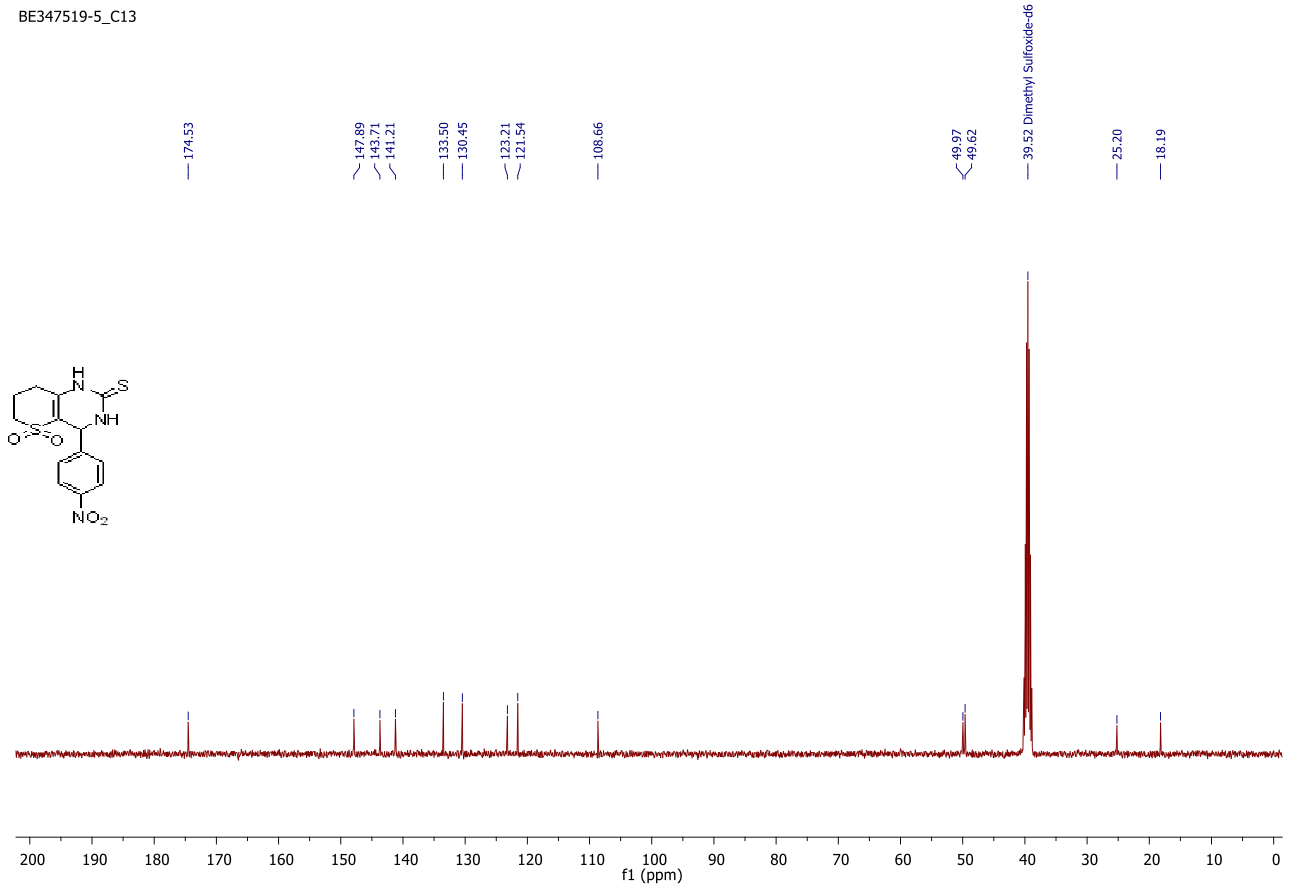

Figure S18: ^{13}C NMR spectrum of **2e** (126 MHz, DMSO-d_6)

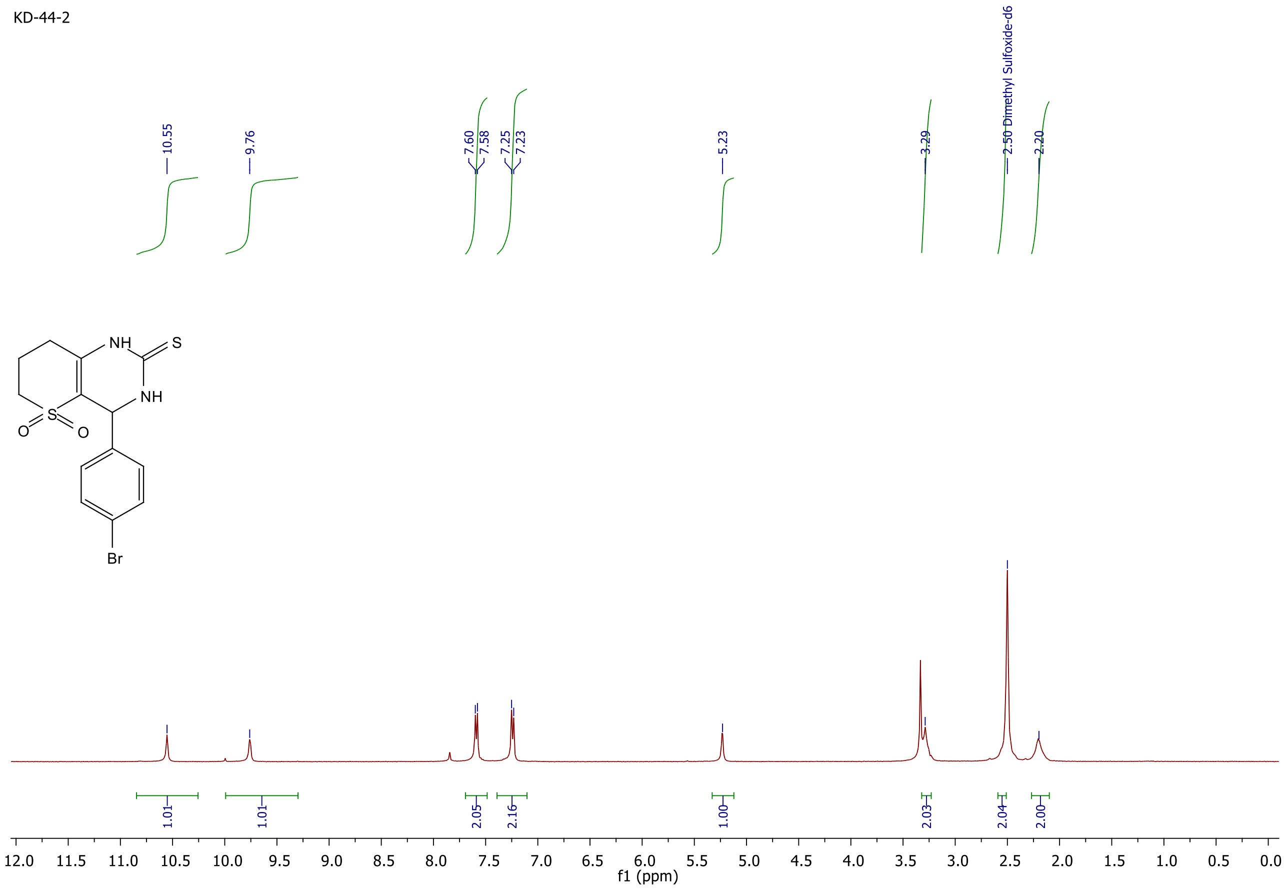

Figure S19: ^{1}H NMR spectrum of **2f** (500 MHz, DMSO-d_6)

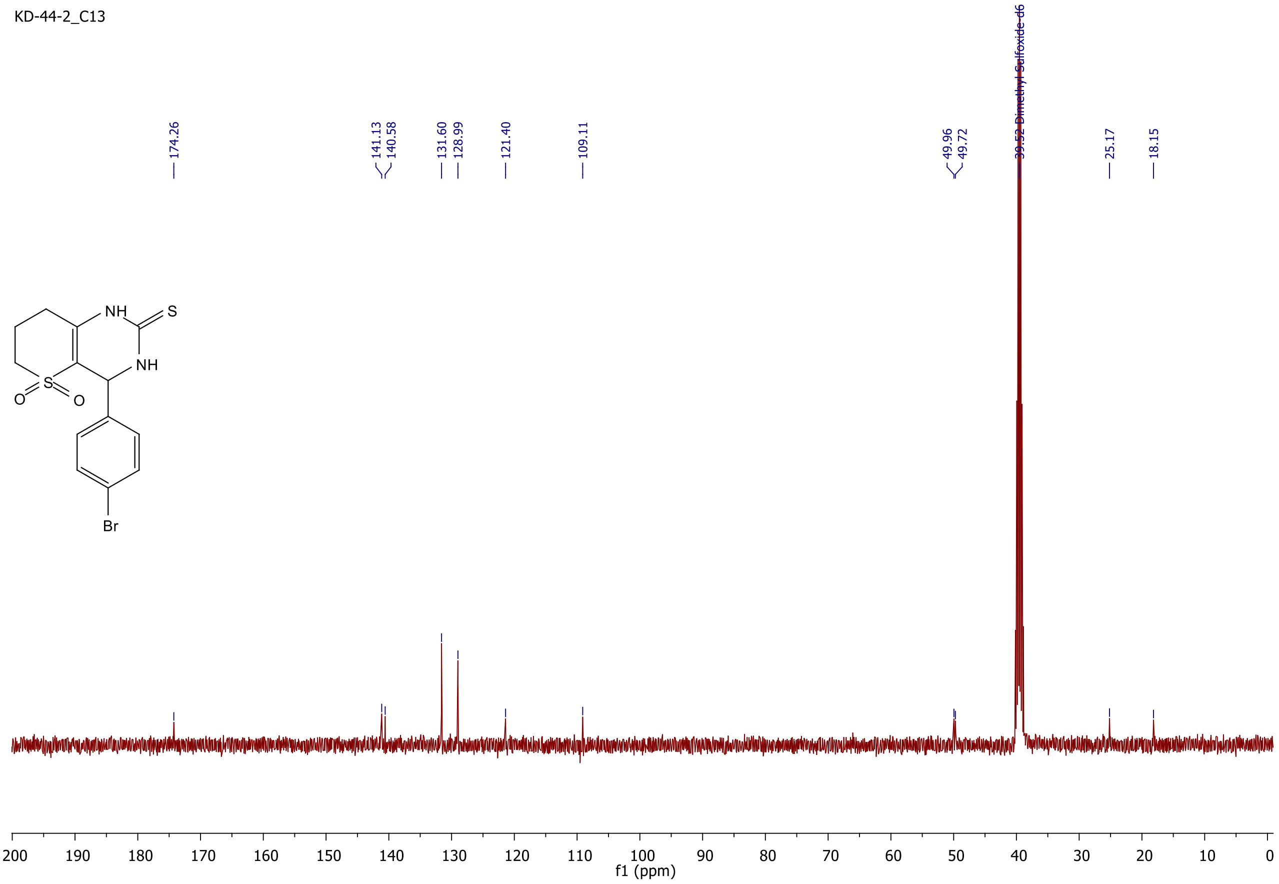

Figure S20: ^{13}C NMR spectrum of **2f** (126 MHz, DMSO-d_6)

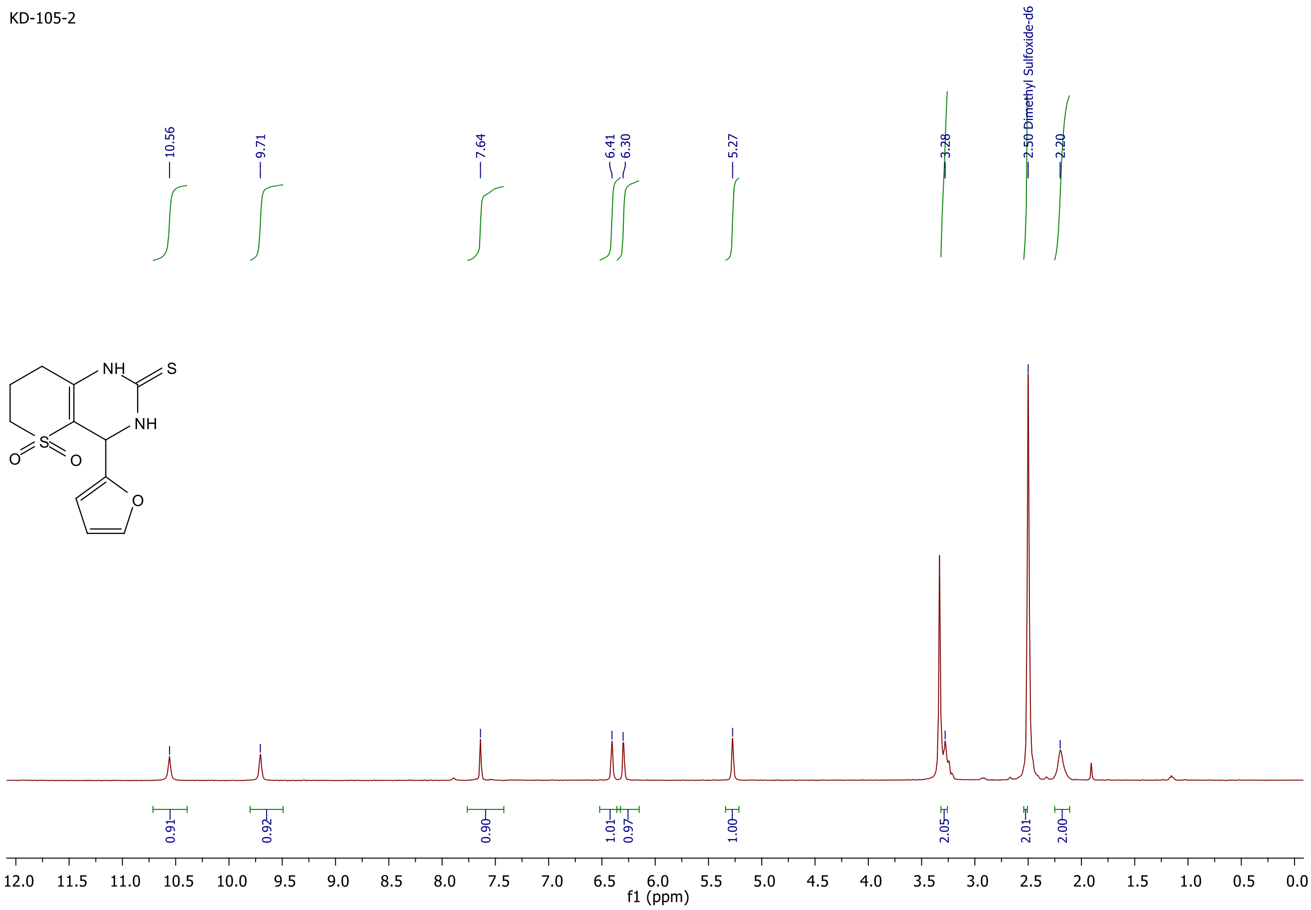

Figure S21: ^{1}H NMR spectrum of **2g** (500 MHz, DMSO-d_6)

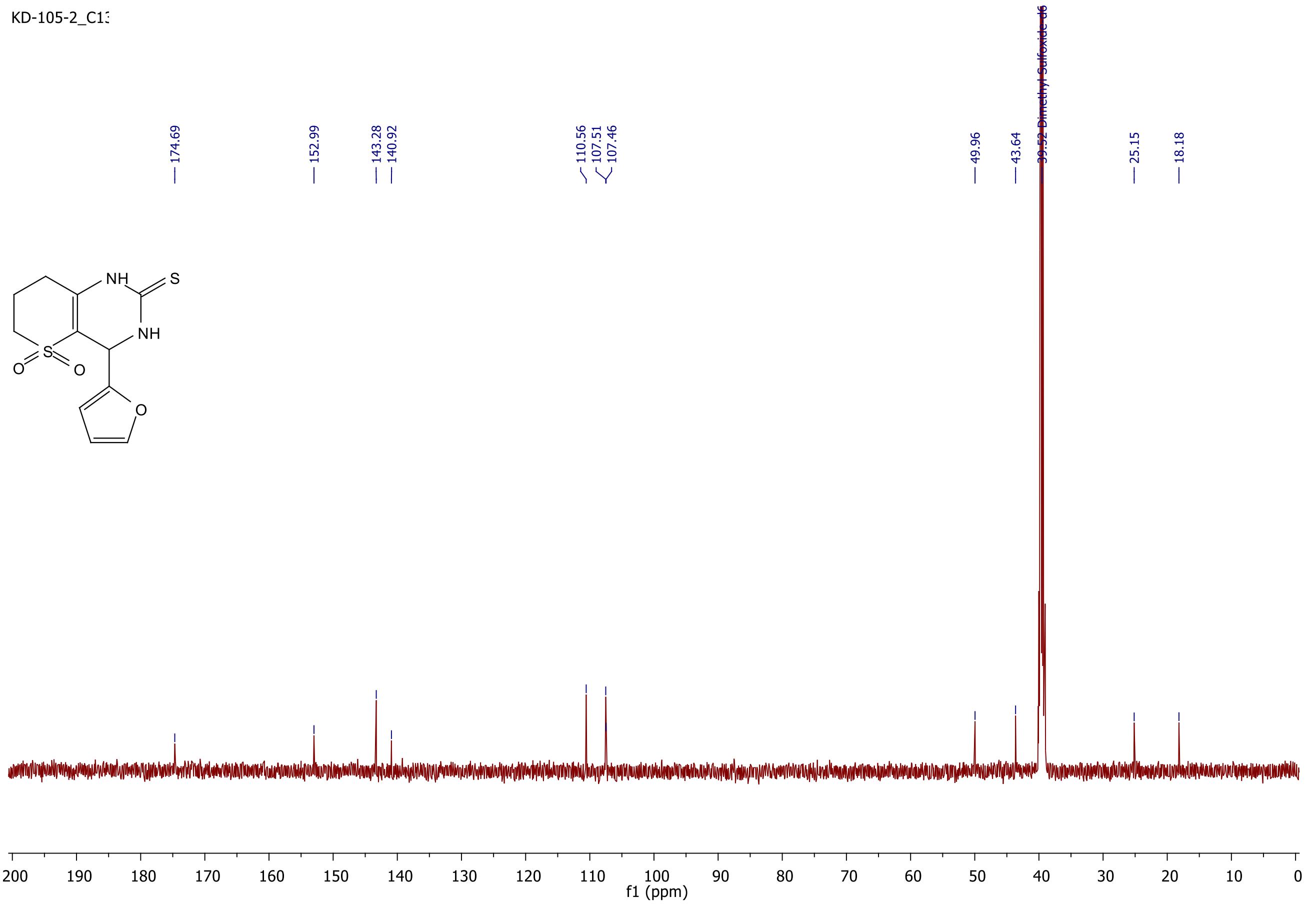

Figure S22: ^{13}C NMR spectrum of **2g** (126 MHz, DMSO-d_6)

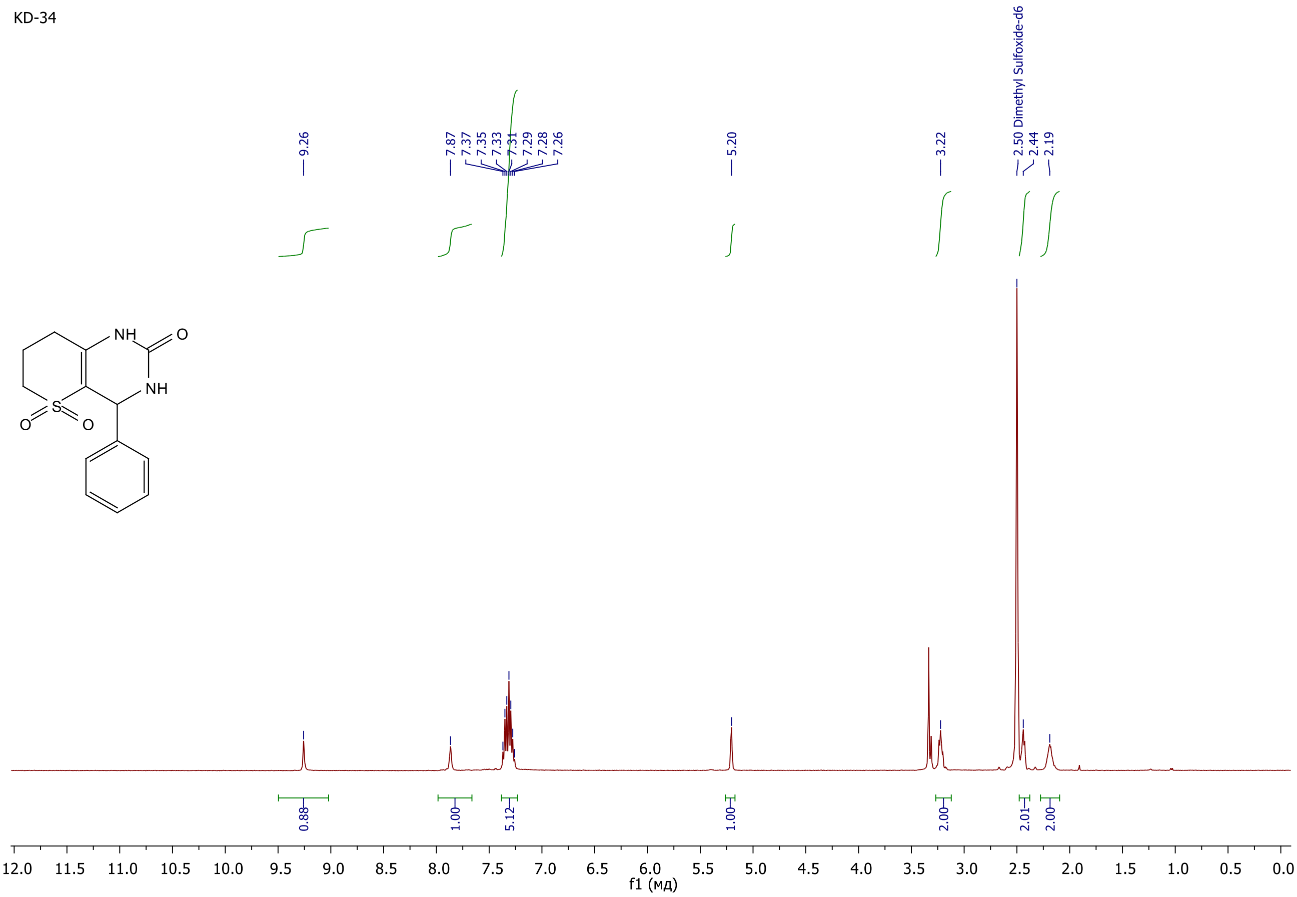

Figure S23: ^{1}H NMR spectrum of **2h** (500 MHz, DMSO-d_6)

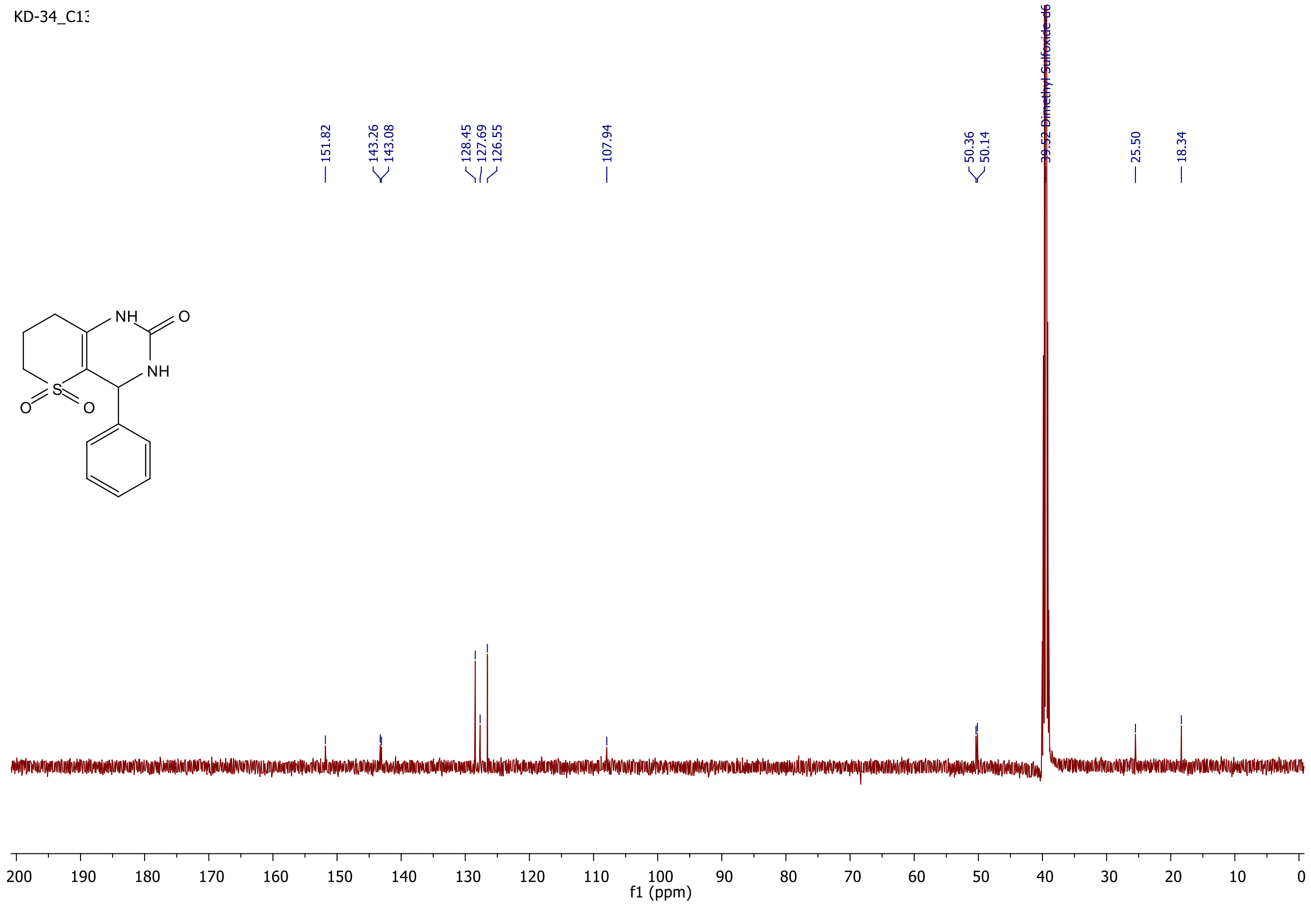

Figure S24: ^{13}C NMR spectrum of **2h** (126 MHz, DMSO-d_6)

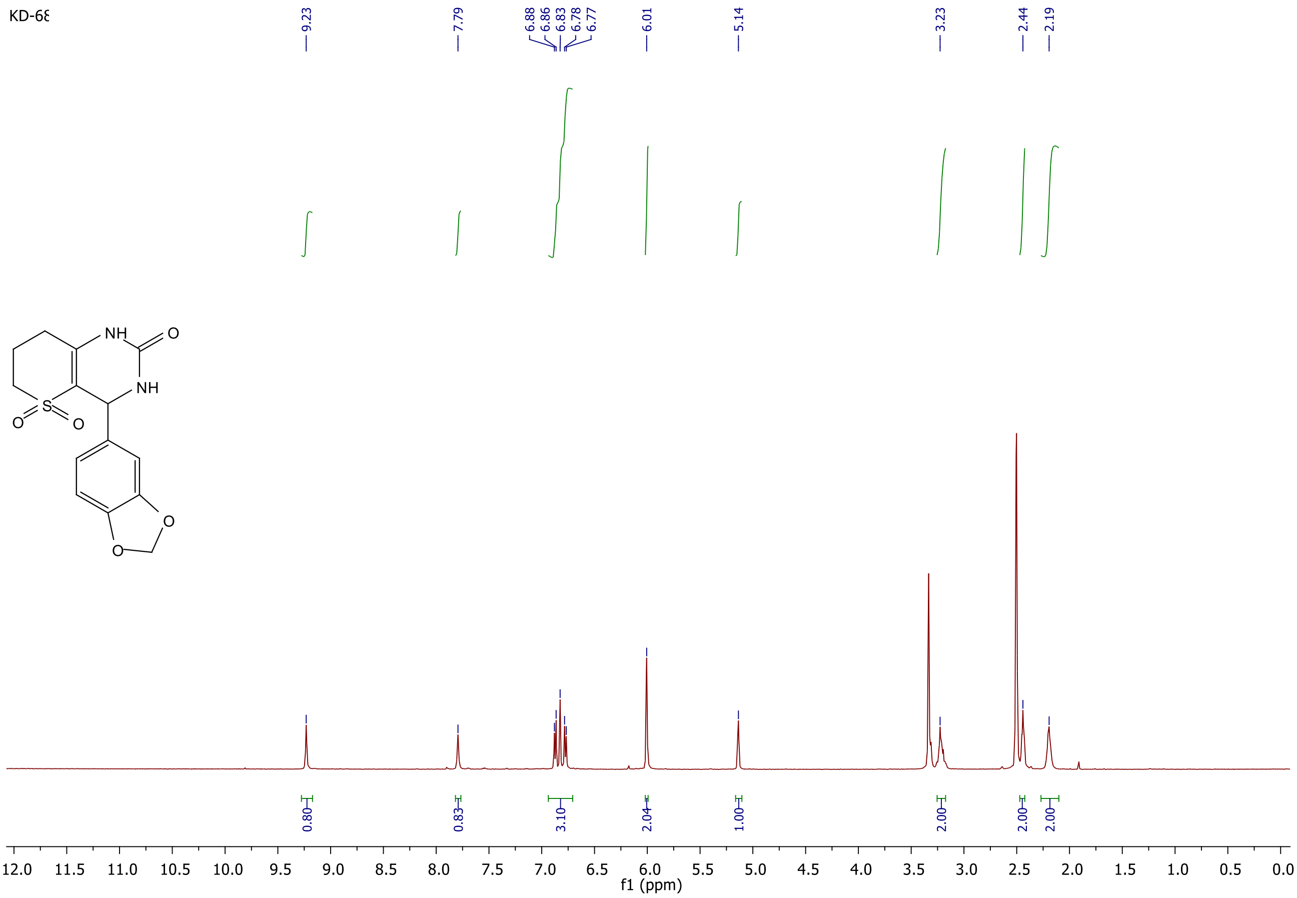

Figure S25: ^{1}H NMR spectrum of **2i** (500 MHz, DMSO-d_6)

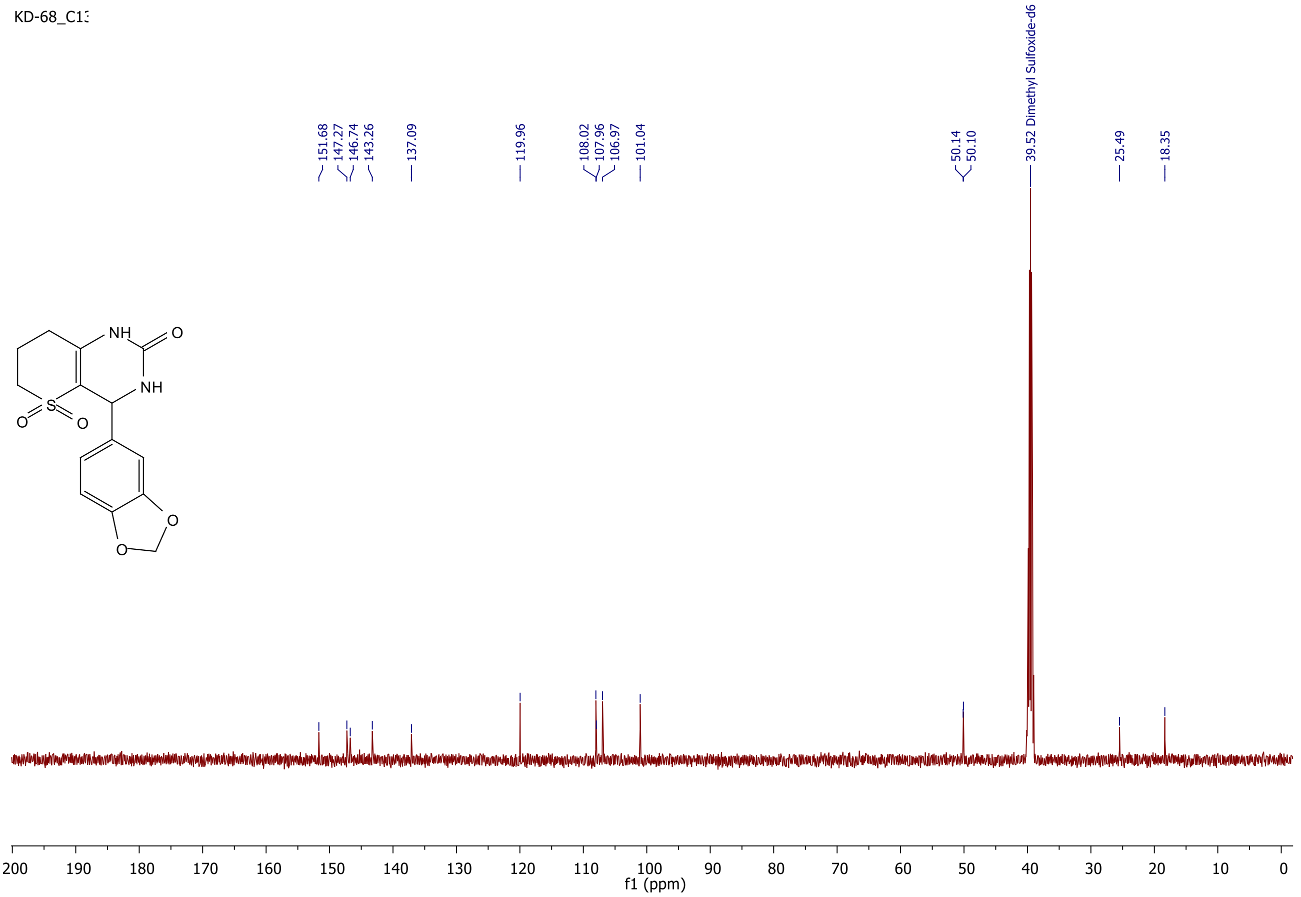

Figure S26: ^{13}C NMR spectrum of **2i** (126 MHz, DMSO-d_6)

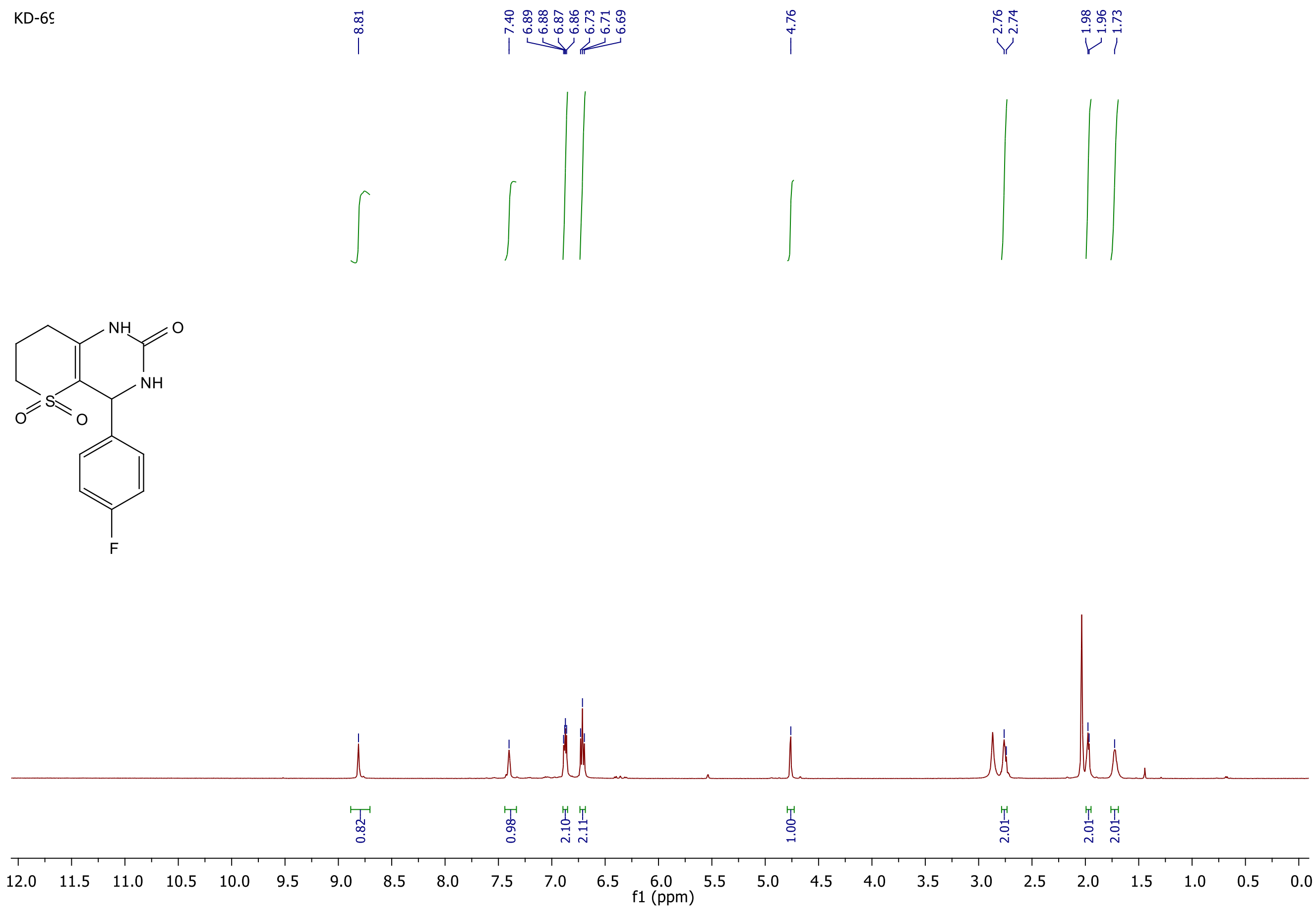

Figure S27: ^{1}H NMR spectrum of **2j** (500 MHz, DMSO-d_6)

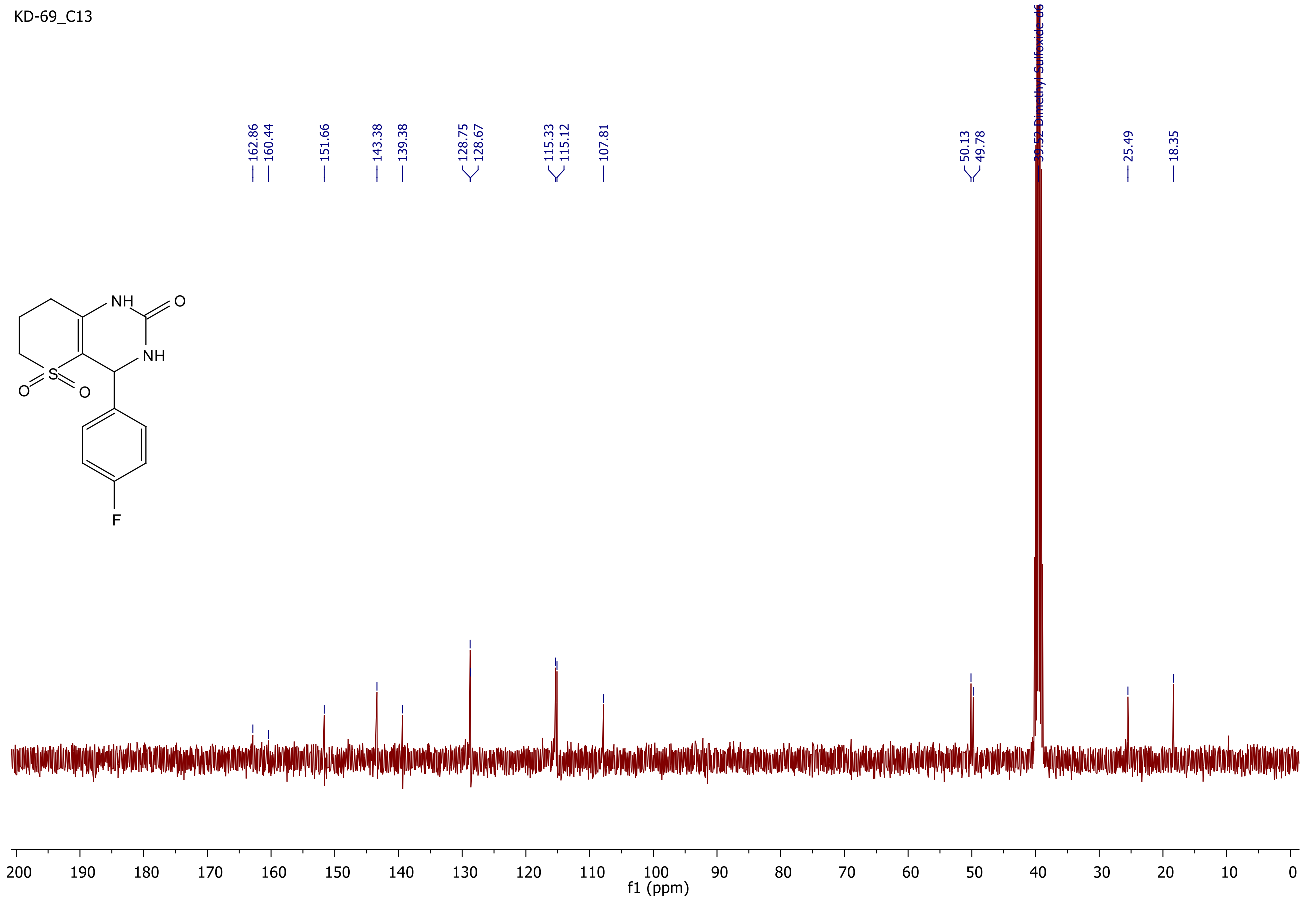

Figure S28: ^{13}C NMR spectrum of **2j** (126 MHz, DMSO-d_6)

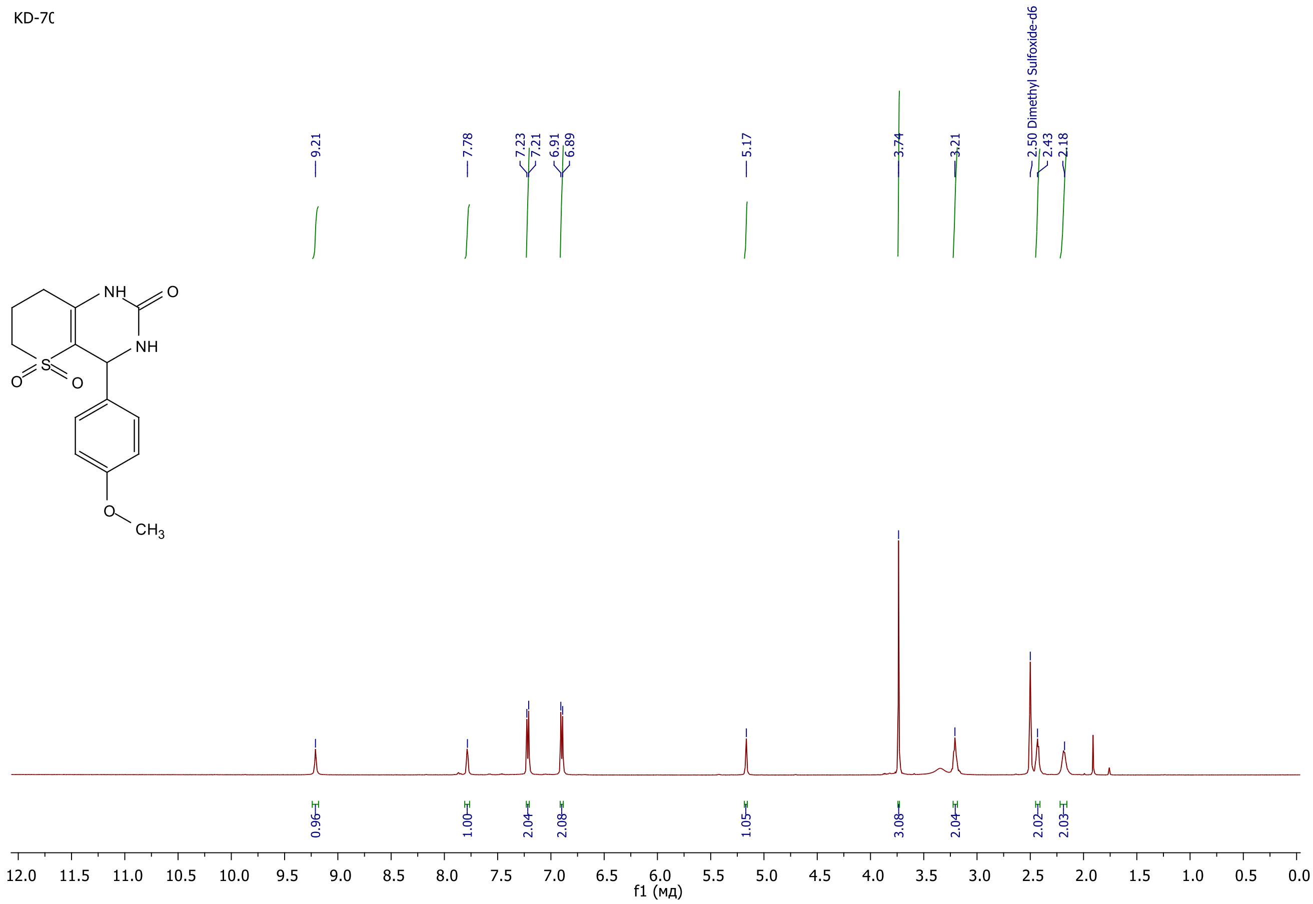

Figure S29: ^{1}H NMR spectrum of **2k** (500 MHz, DMSO-d_6)

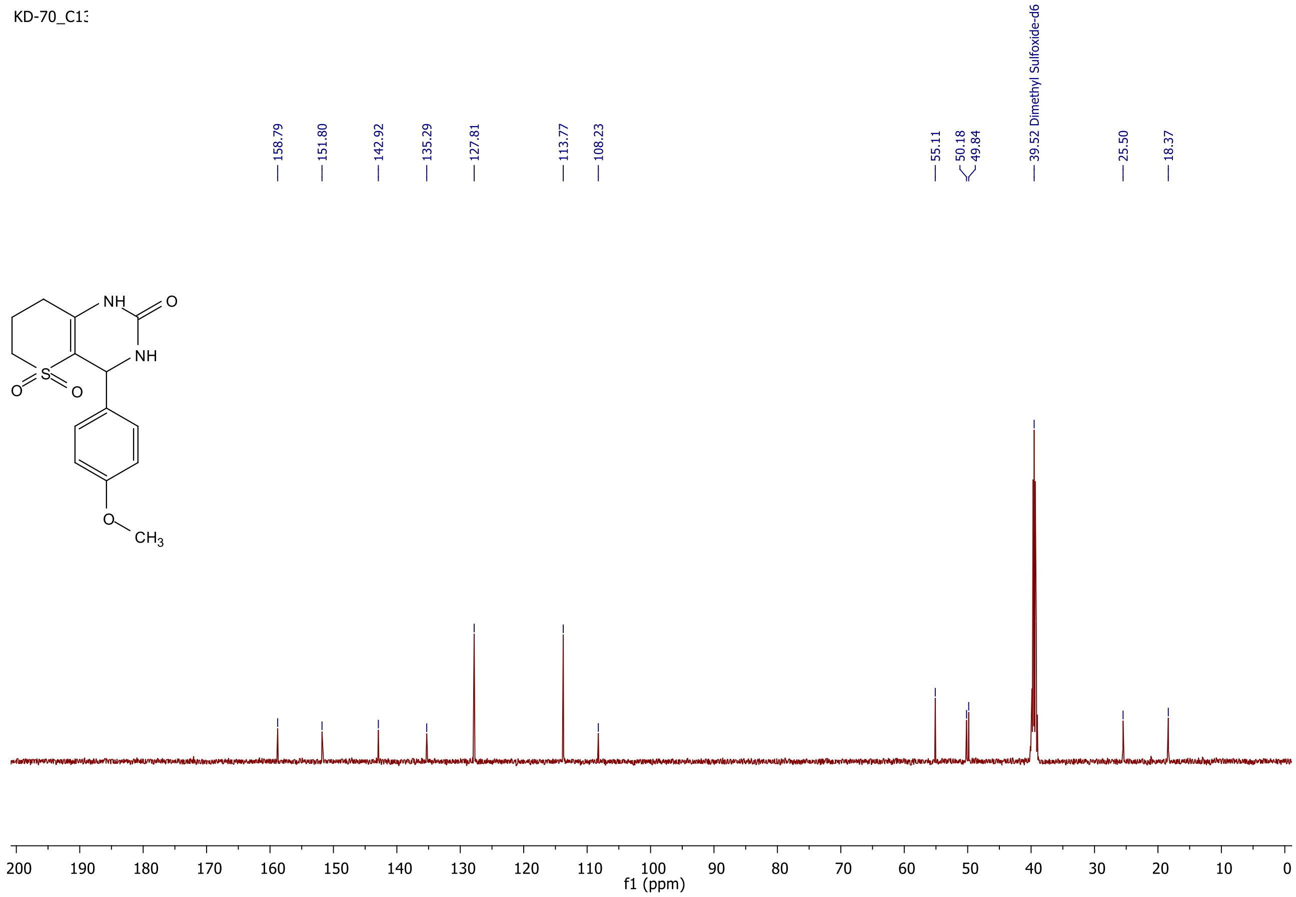

Figure S30: ^{13}C NMR spectrum of **2k** (126 MHz, DMSO-d_6)

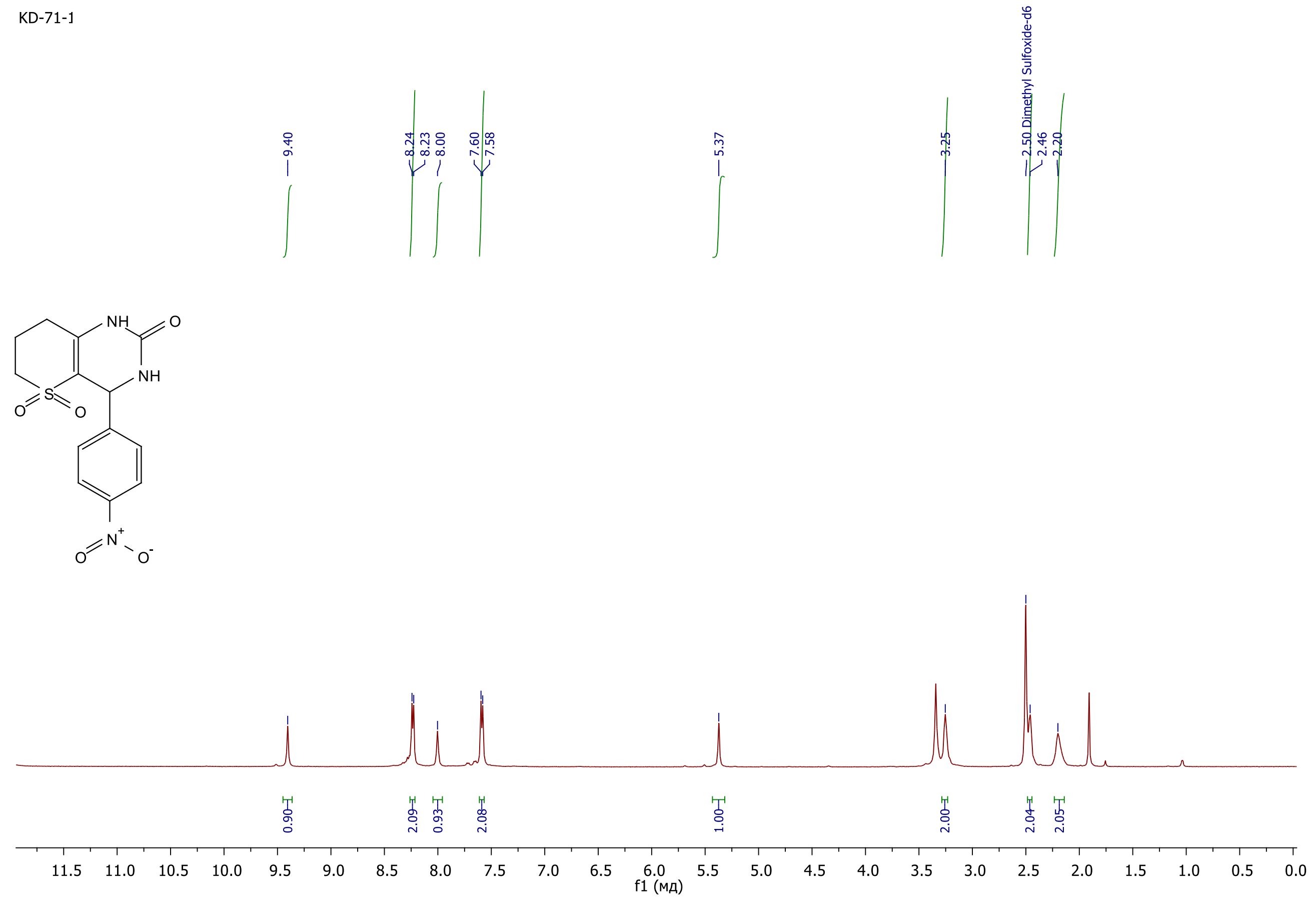

Figure S31: ^{1}H NMR spectrum of **2l** (500 MHz, DMSO-d_6)

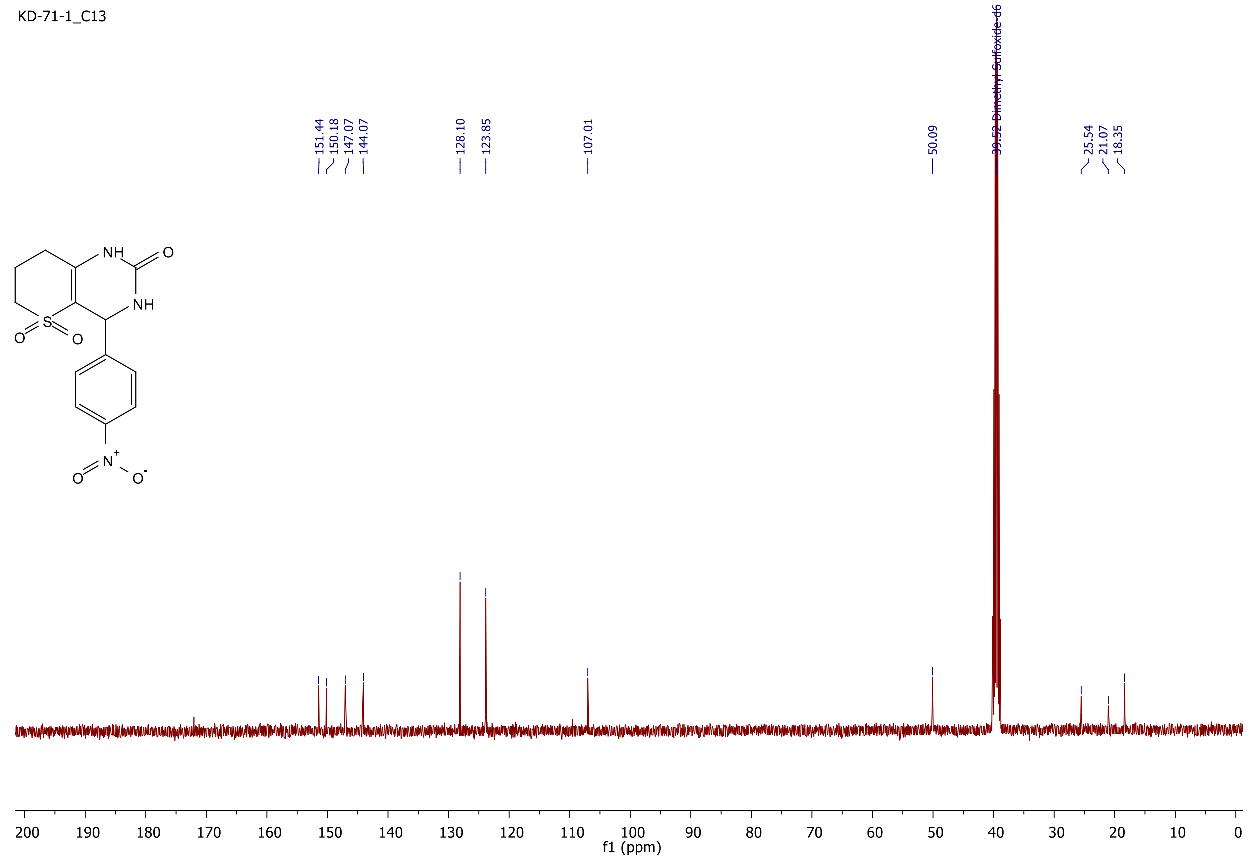

Figure S32: ^{13}C NMR spectrum of **2l** (126 MHz, DMSO-d_6)

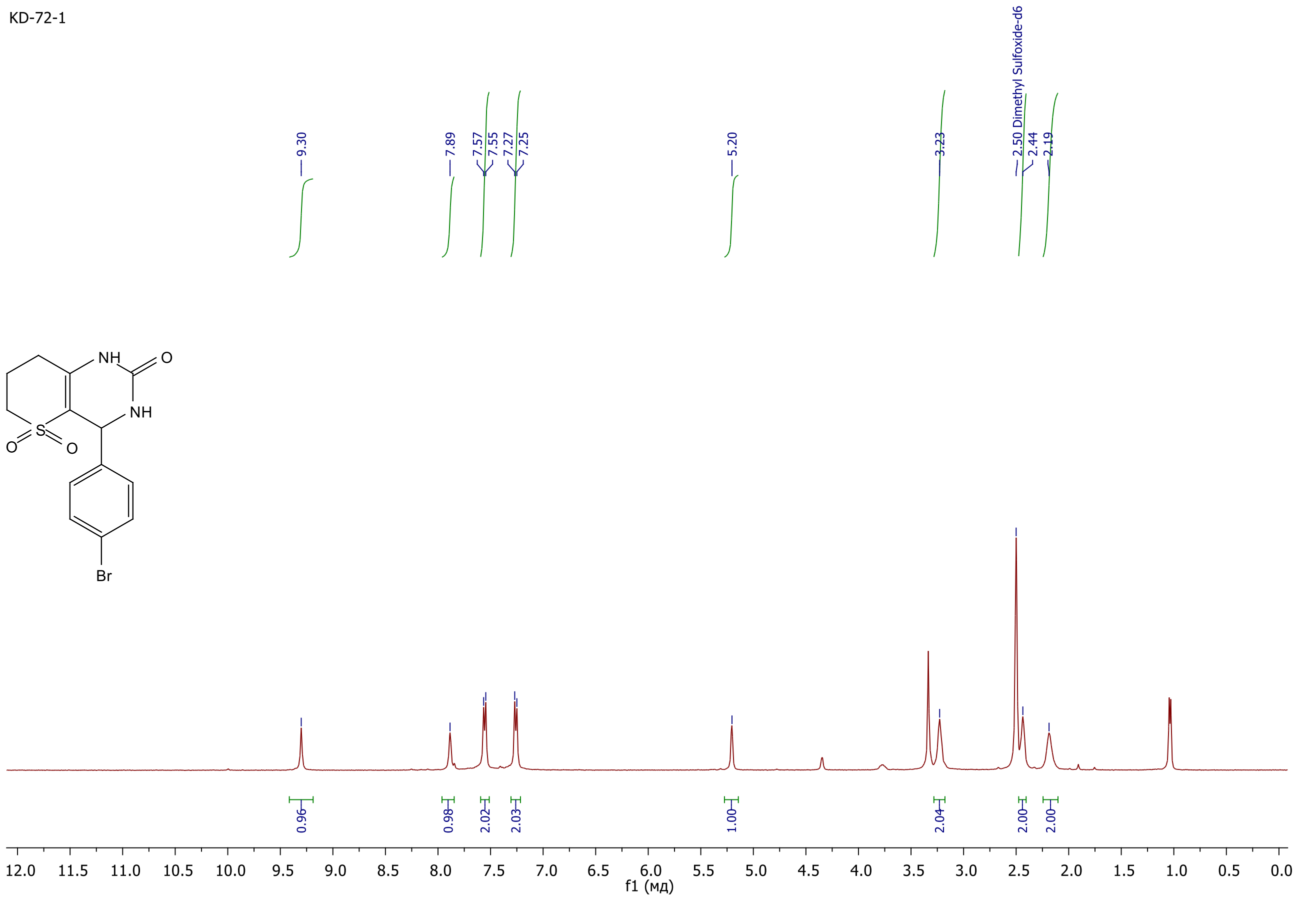

Figure S33: ^{1}H NMR spectrum of **2m** (500 MHz, DMSO-*d*6)

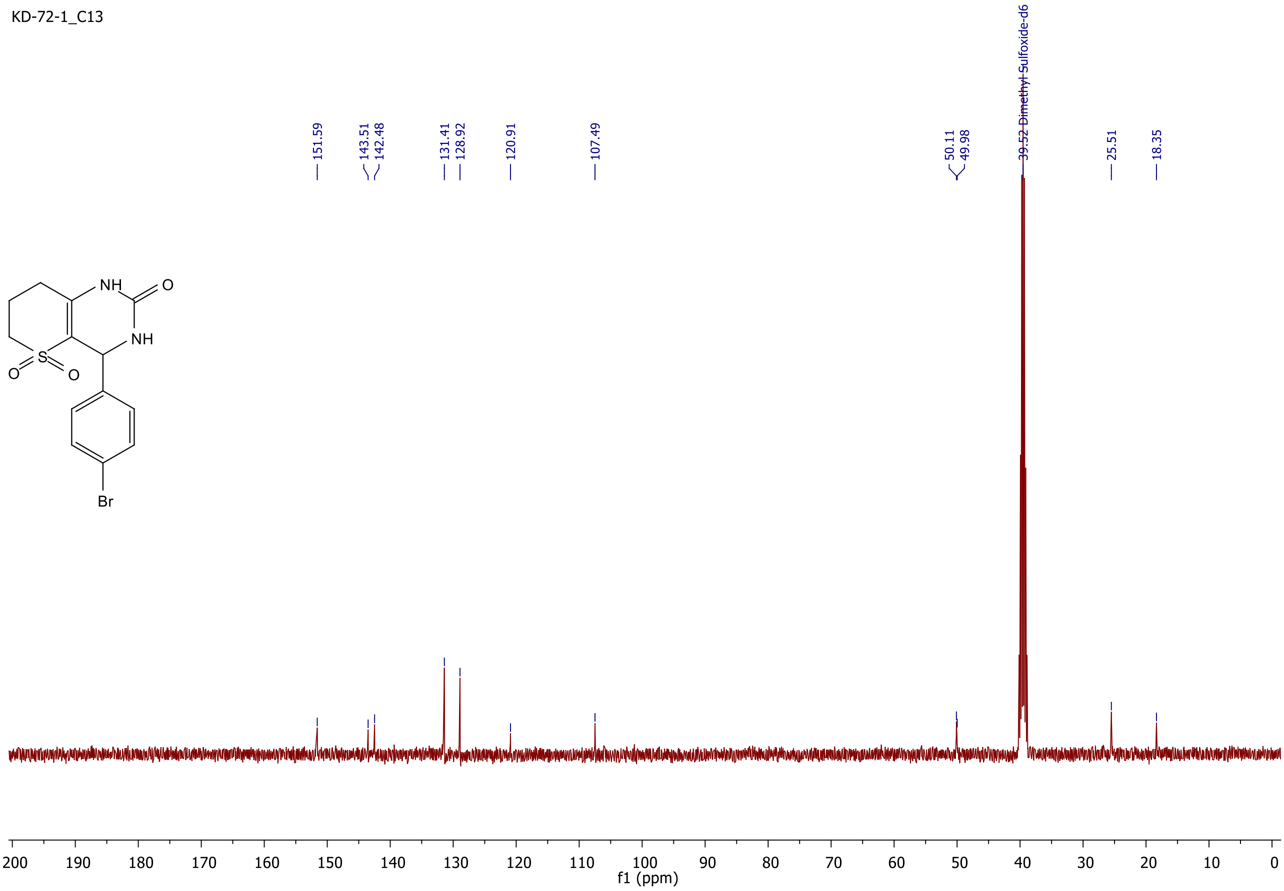

Figure S34: ^{13}C NMR spectrum of **2m** (126 MHz, DMSO-*d*6)

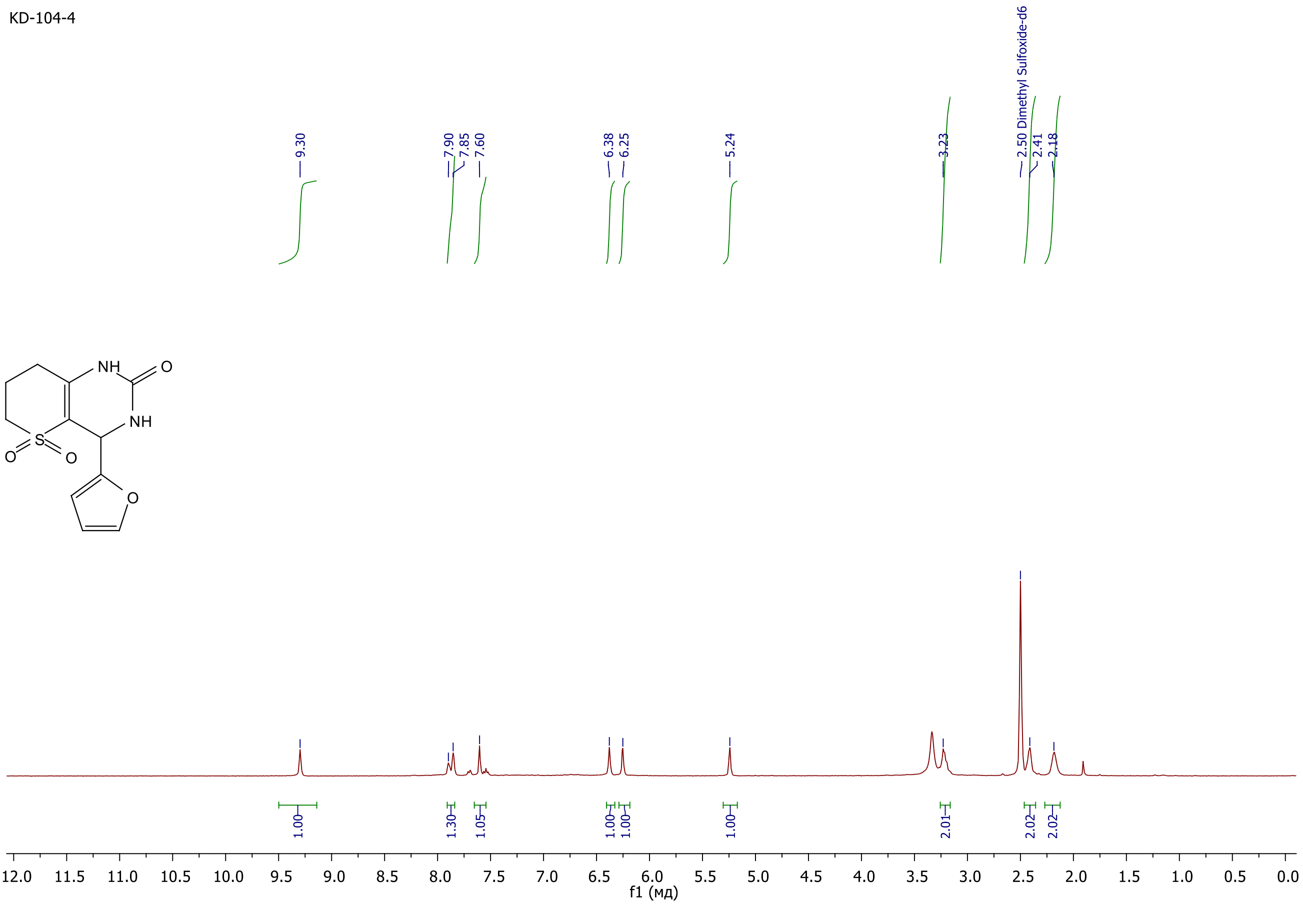

Figure S35: ^{1}H NMR spectrum of **2n** (500 MHz, DMSO-d_6)

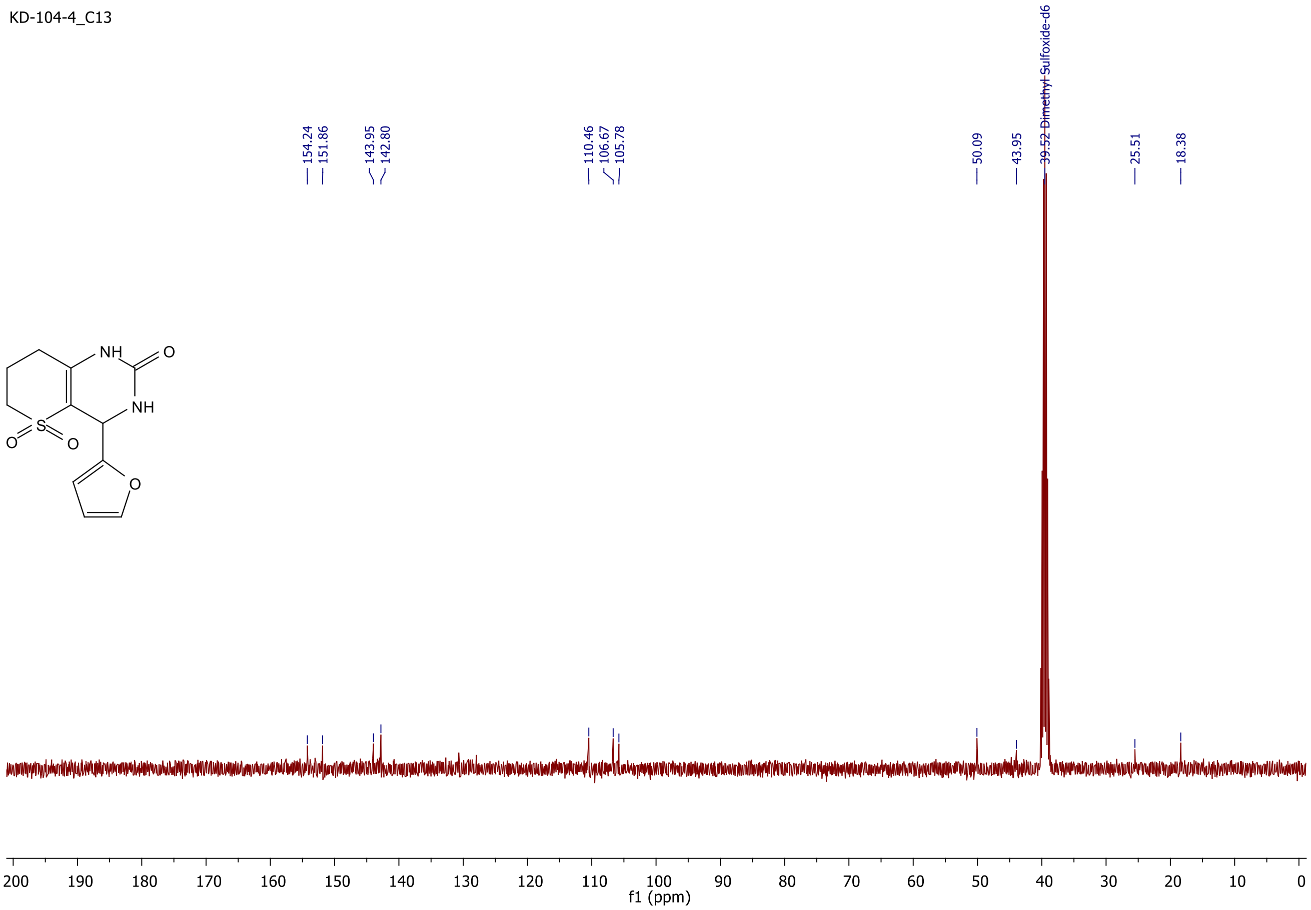

Figure S36: ^{13}C NMR spectrum of **2n** (126 MHz, DMSO-d_6)

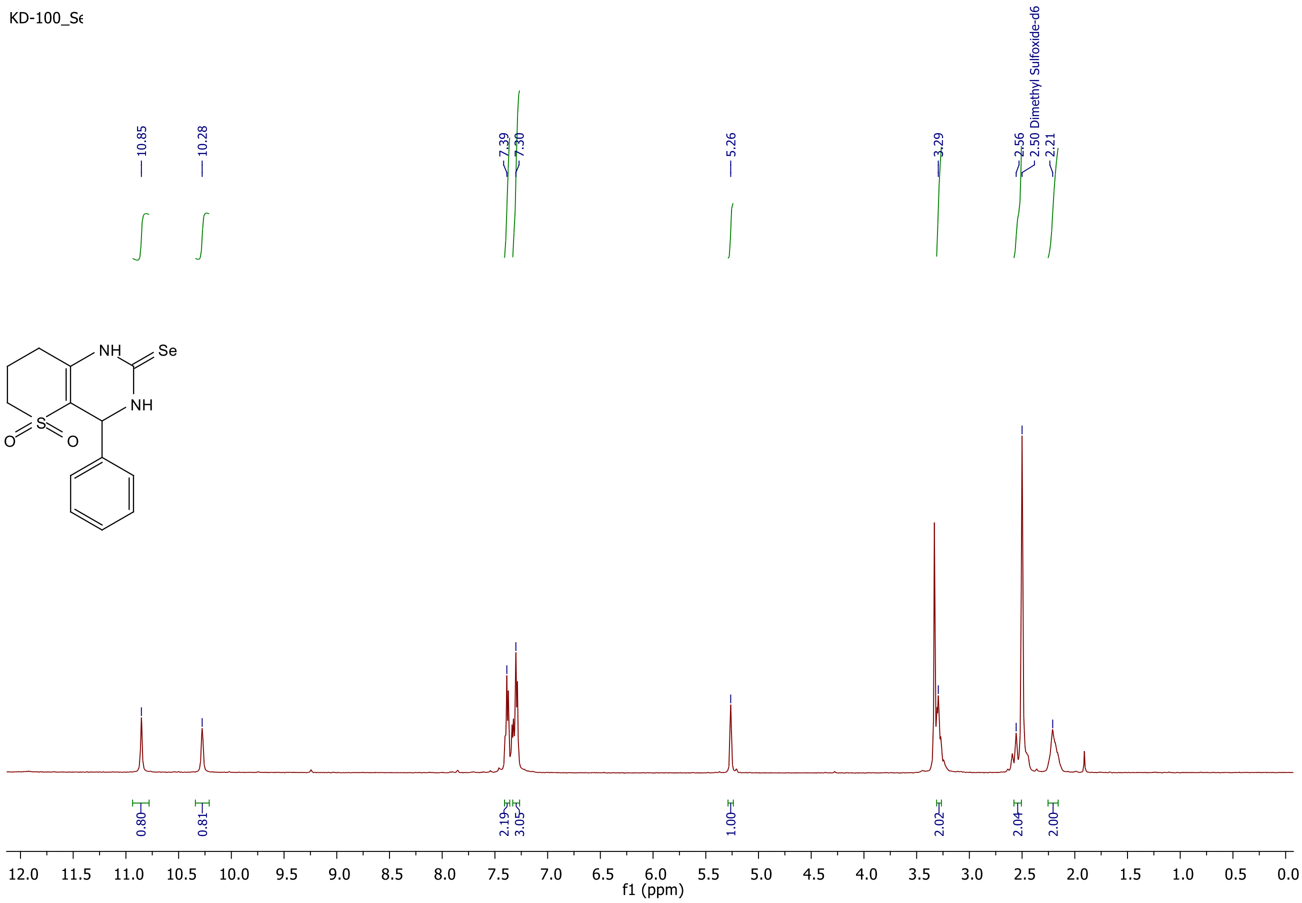

Figure S37: ^{1}H NMR spectrum of **2o** (500 MHz, DMSO-d_6)

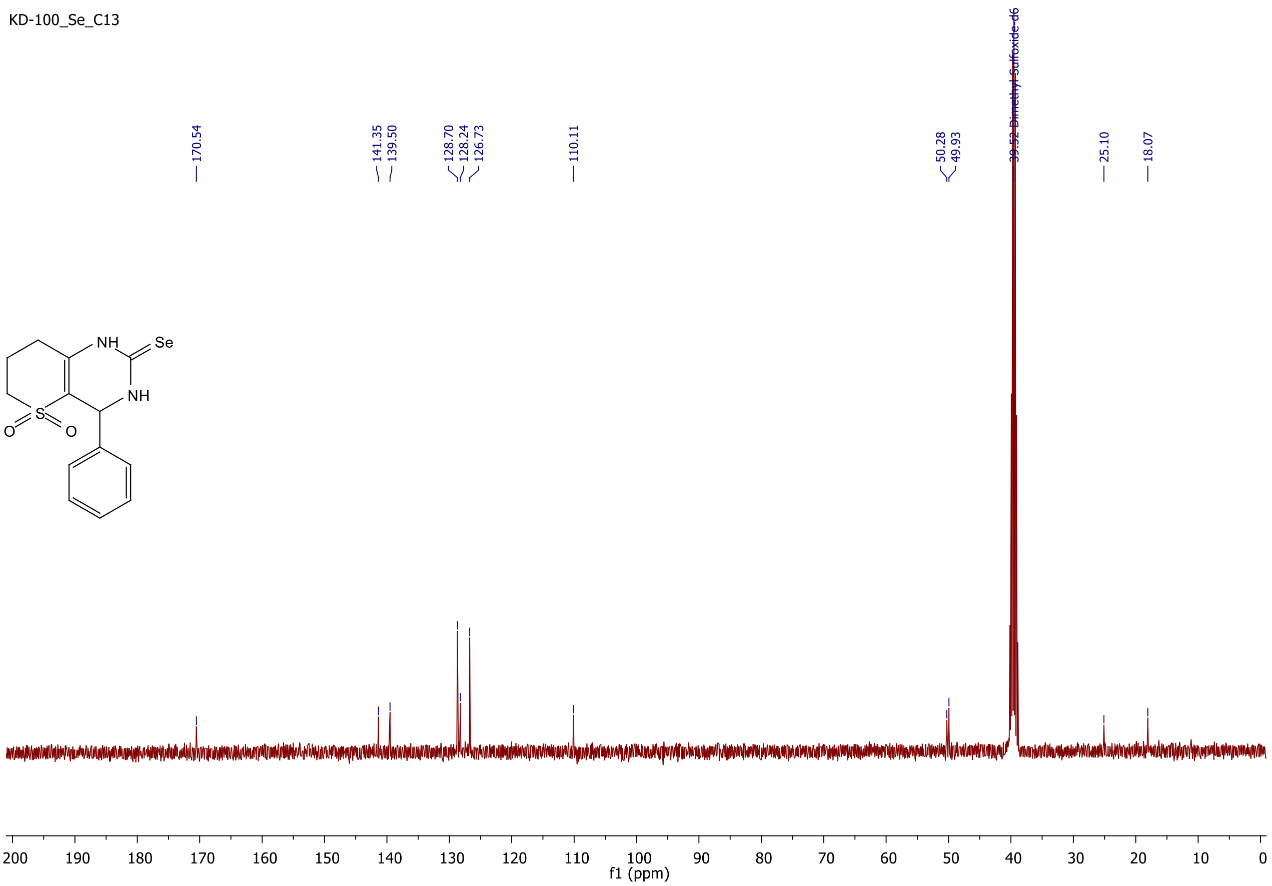

Figure S38: ^{13}C NMR spectrum of **2o** (126 MHz, DMSO-d_6)

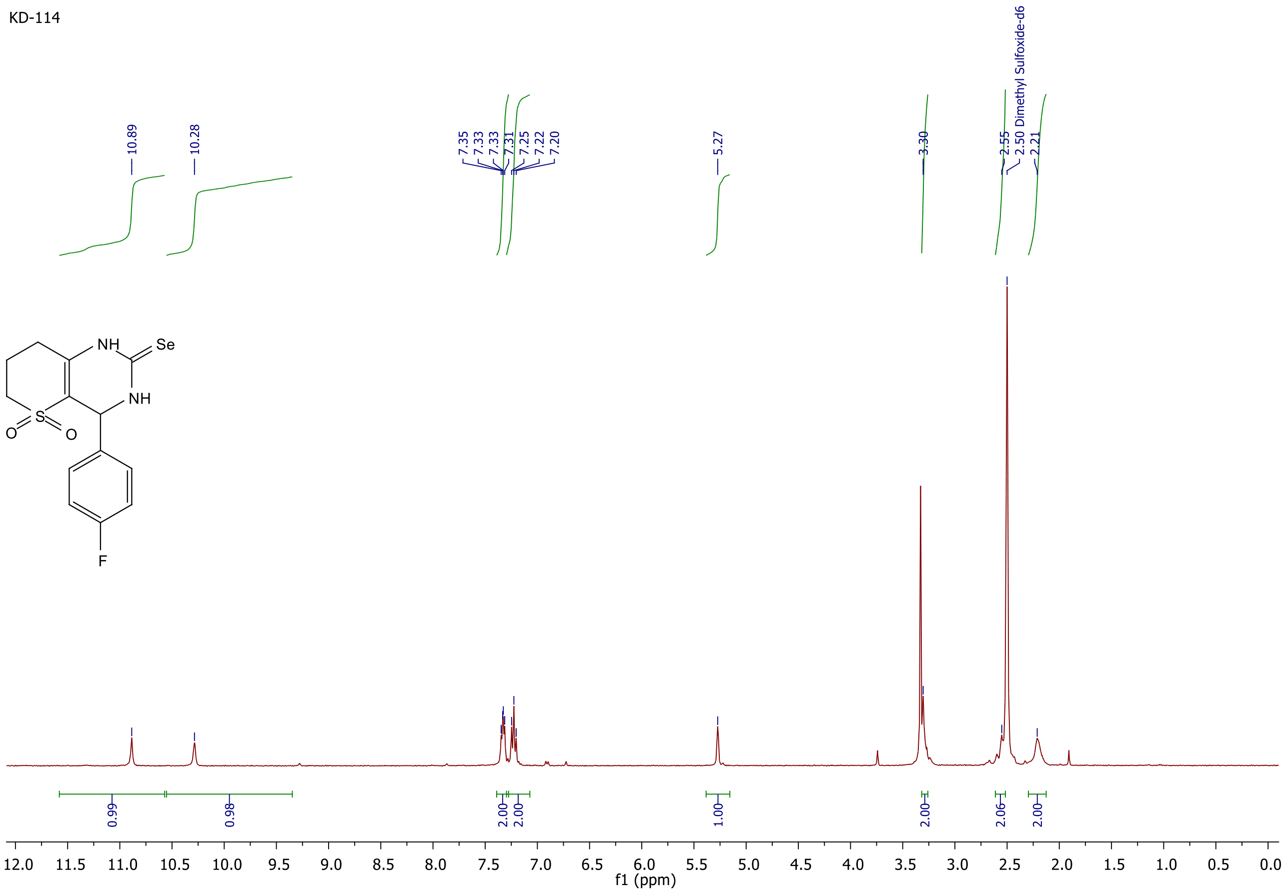

Figure S39: ^{1}H NMR spectrum of **2p** (500 MHz, DMSO-d_6)

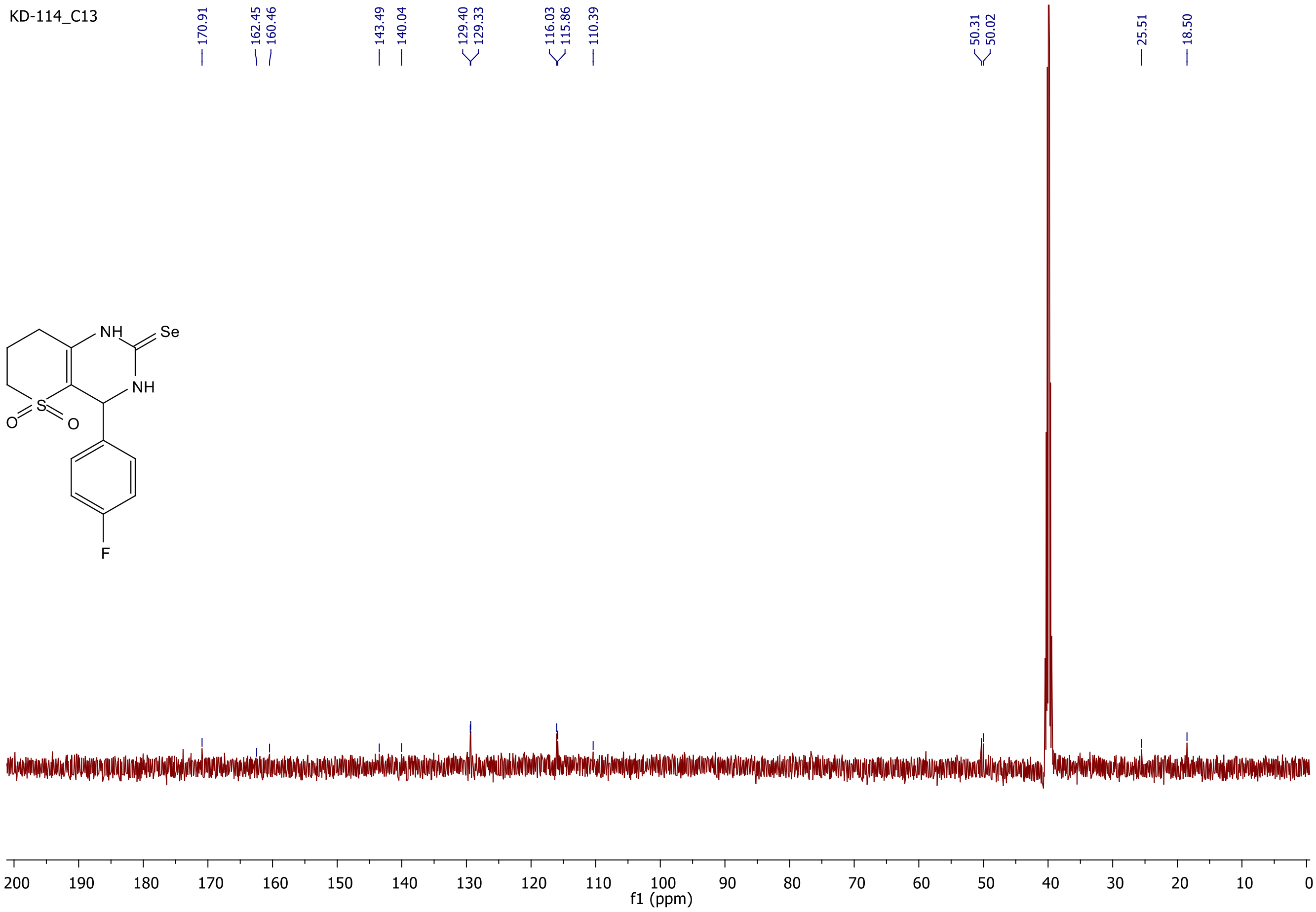

Figure S40: ^{13}C NMR spectrum of **2p** (126 MHz, DMSO-d_6)

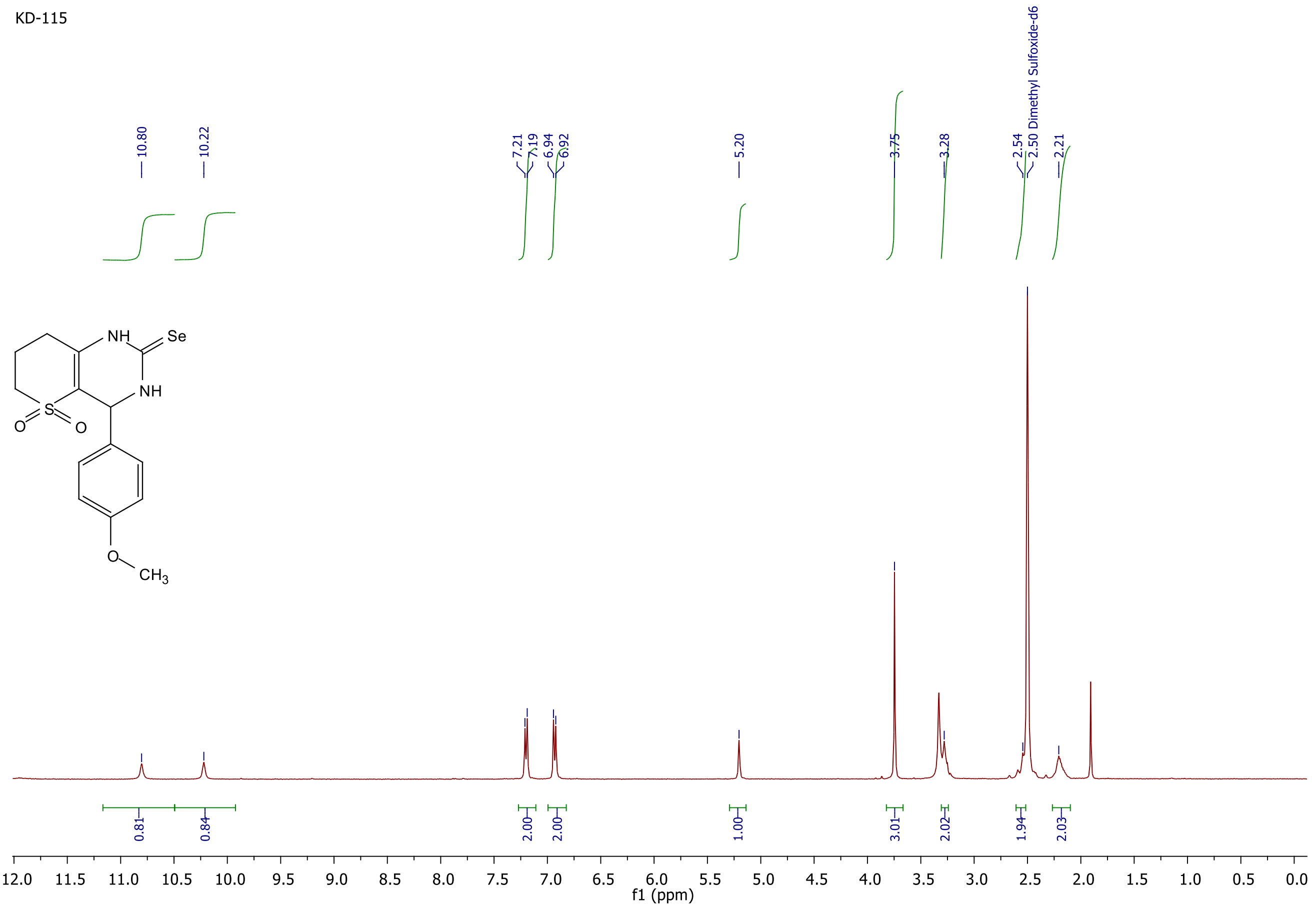

Figure S41: ^{1}H NMR spectrum of **2q** (500 MHz, DMSO-d_6)

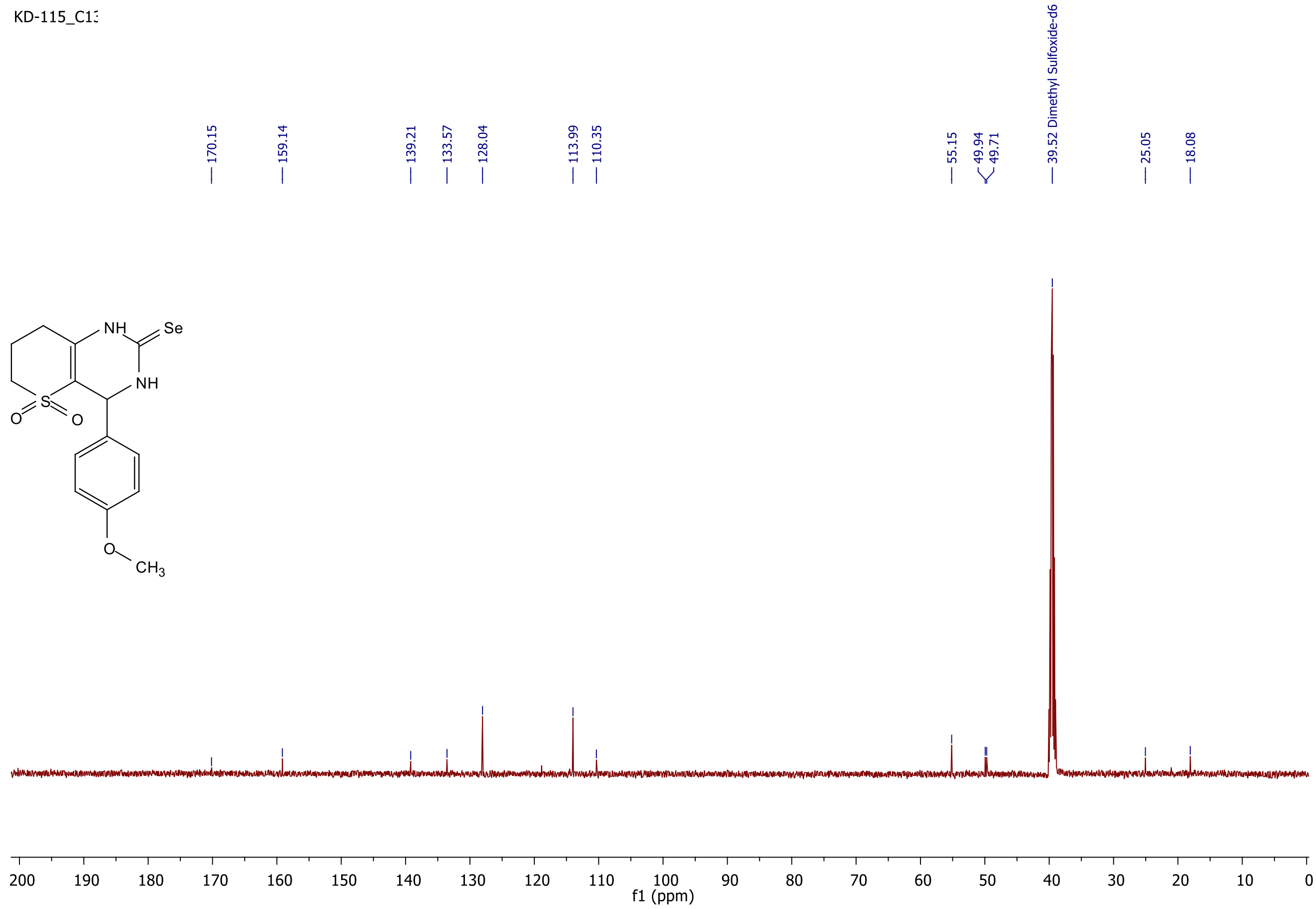

Figure S42: ^{13}C NMR spectrum of **2q** (126 MHz, DMSO-d_6)

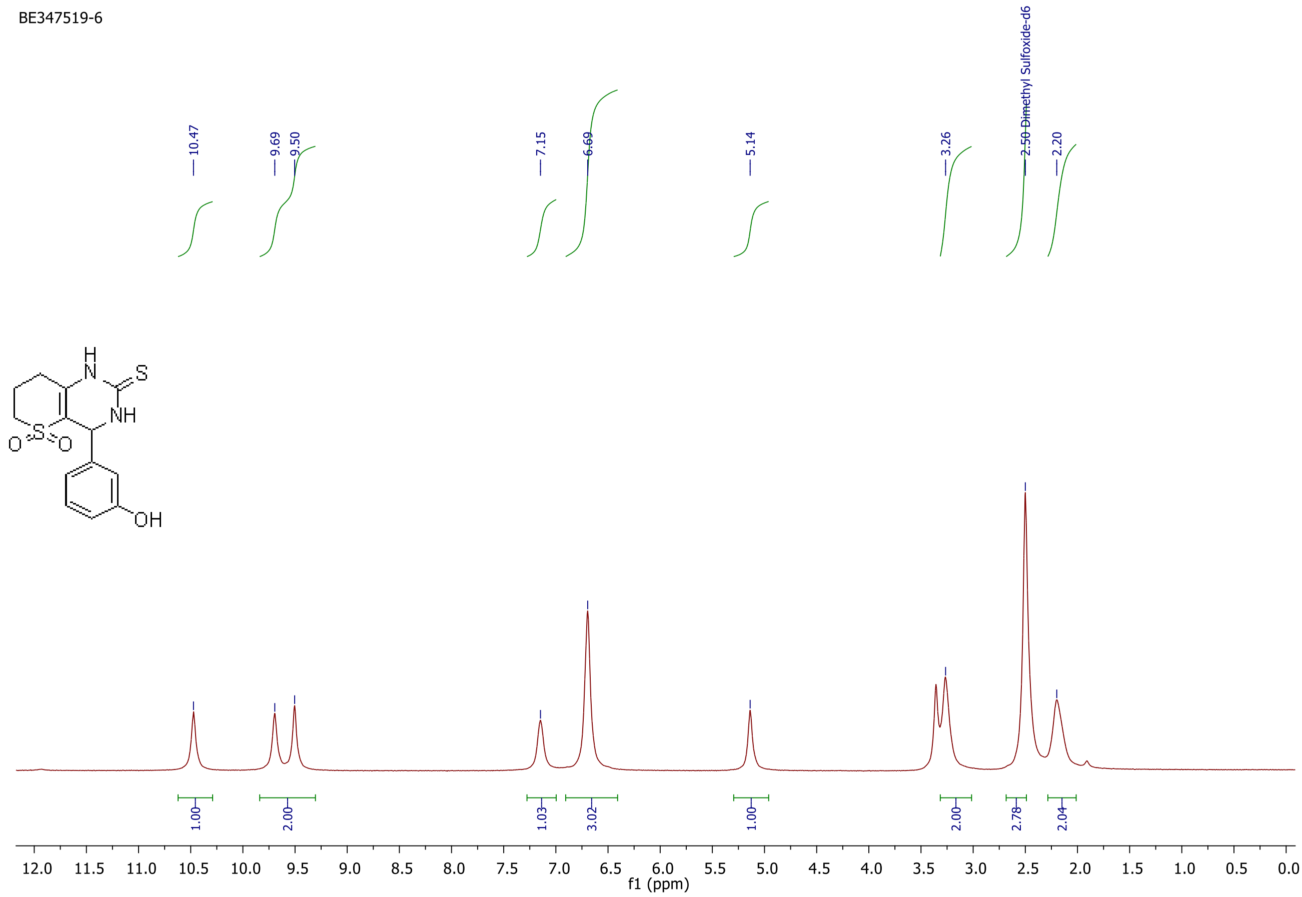

Figure S43: ^{1}H NMR spectrum of **2r** (500 MHz, DMSO-d_6)

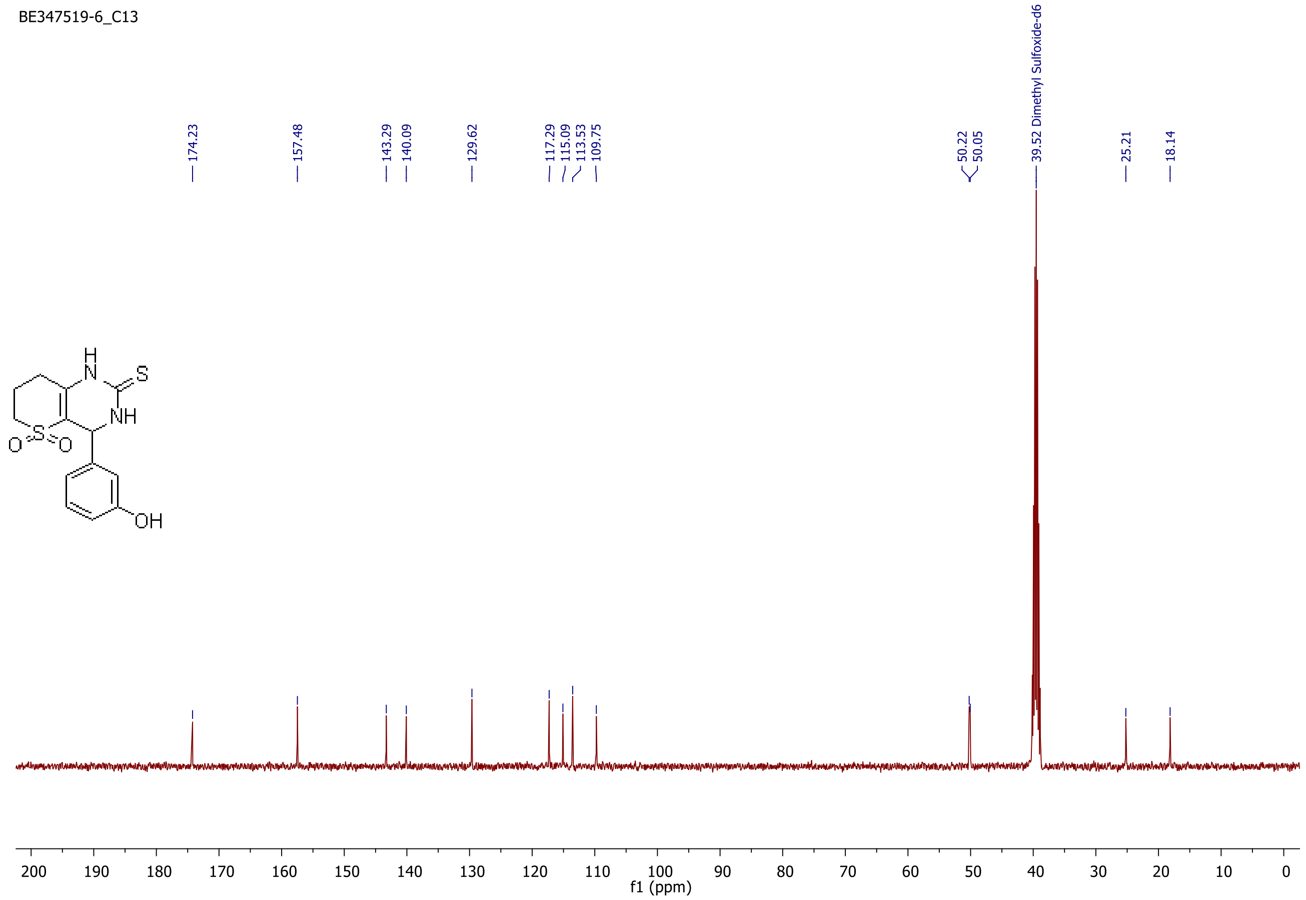

Figure S44: ^{13}C NMR spectrum of **2r** (126 MHz, DMSO-d_6)

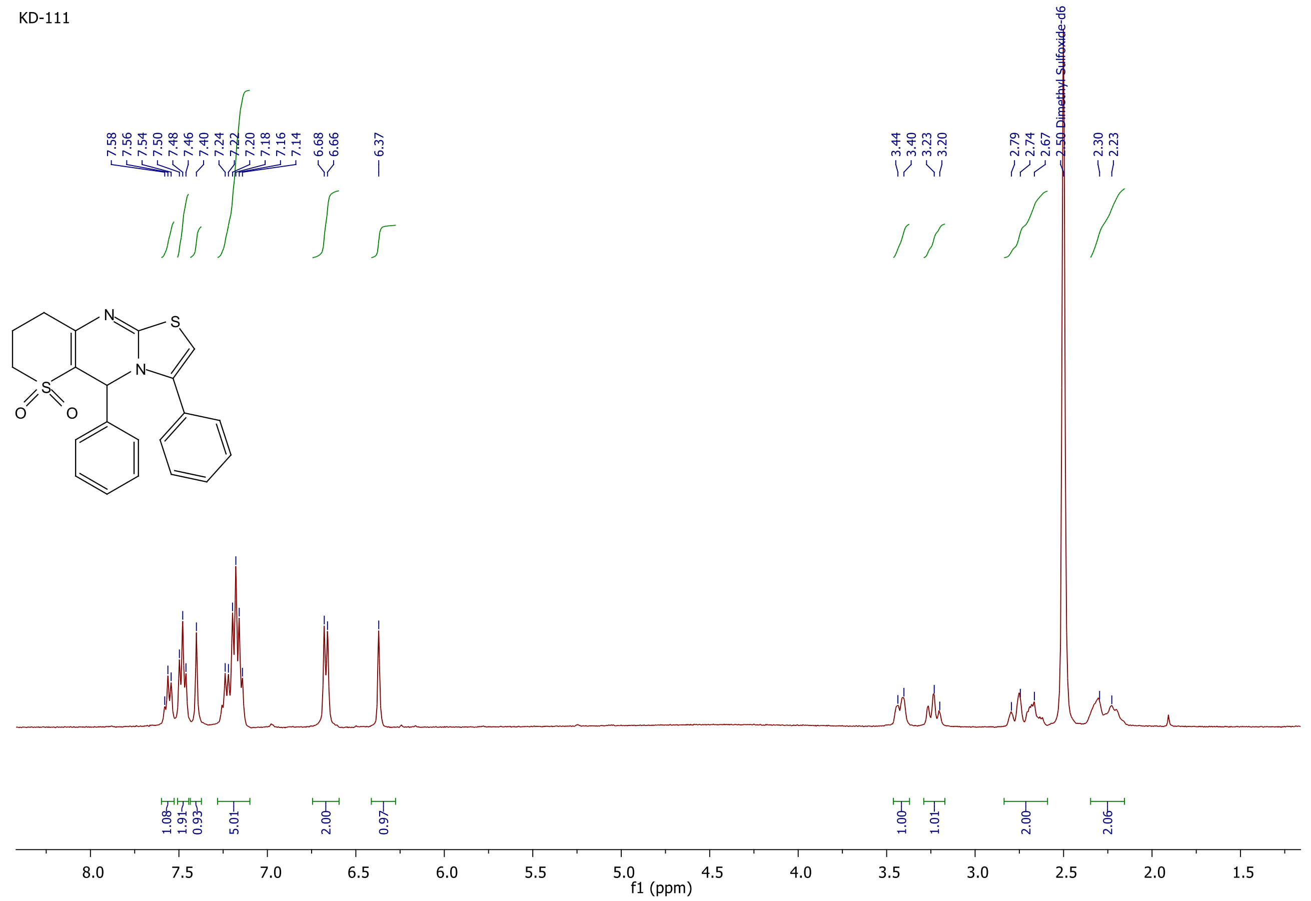

Figure S45: ^{1}H NMR spectrum of **3** (500 MHz, DMSO-d_6)

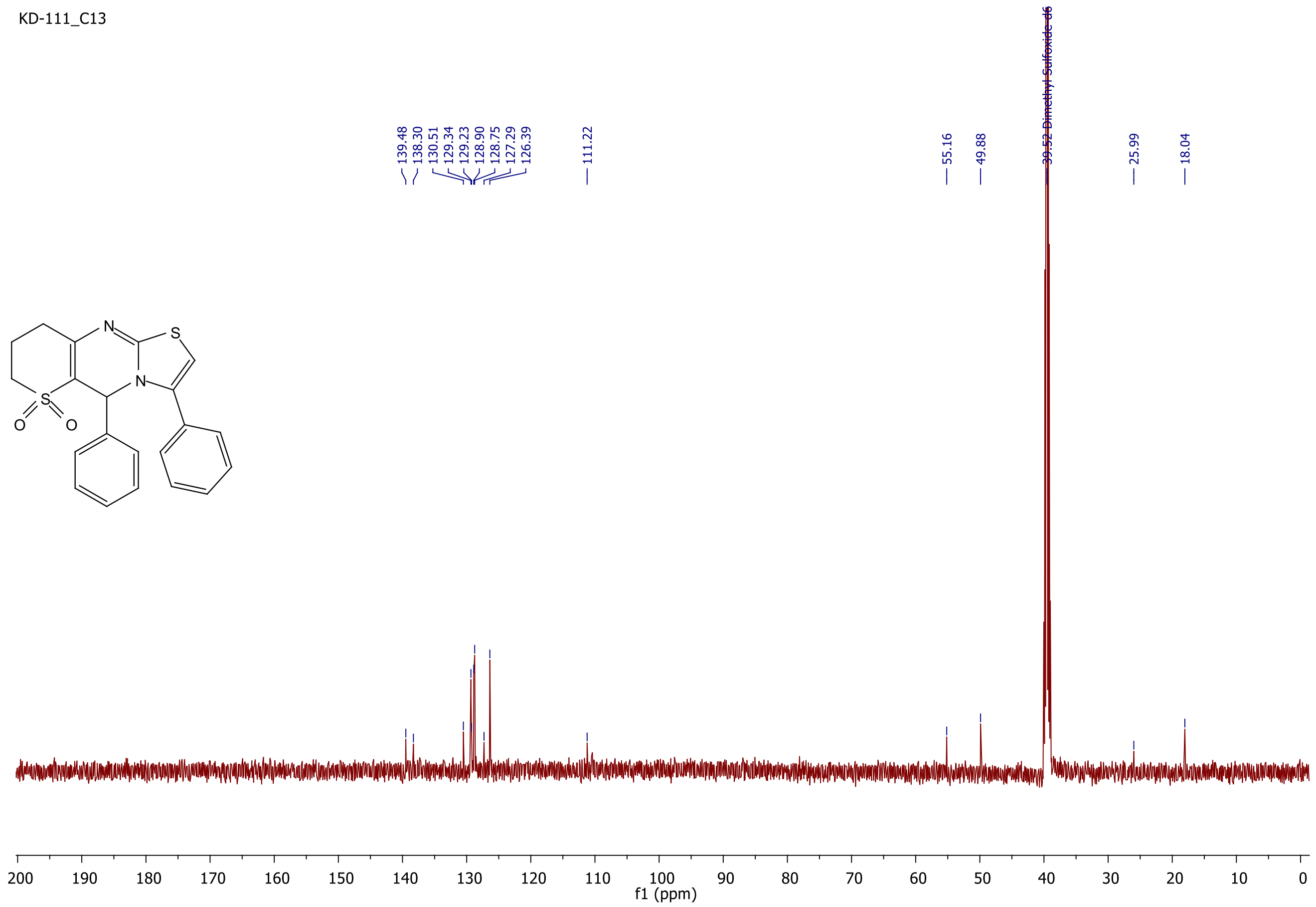

Figure S46: ^{13}C NMR spectrum of **3** (126 MHz, DMSO-d_6)

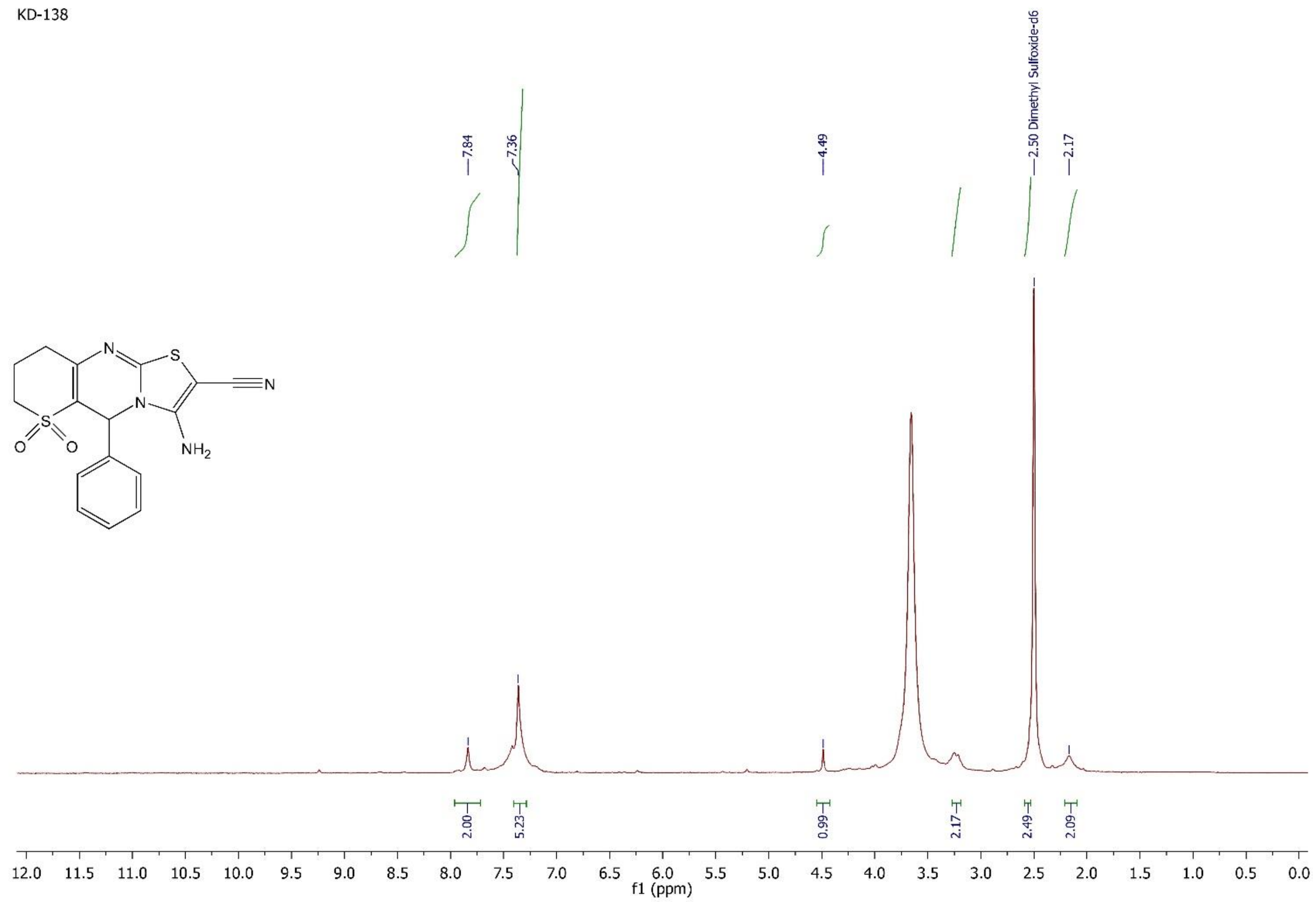

Figure S47: ^{1}H NMR spectrum of **4** (500 MHz, DMSO-d_6)

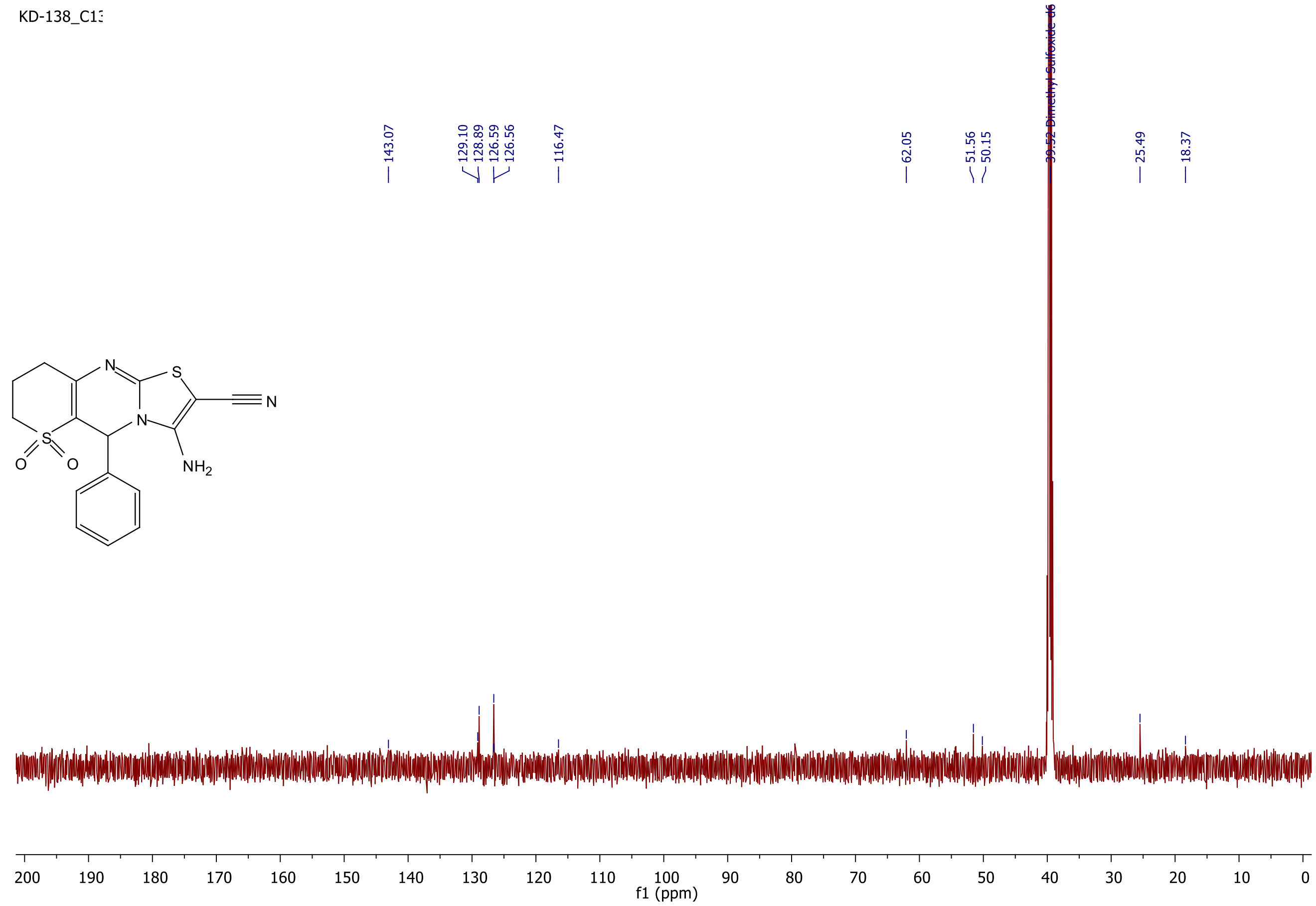

Figure S48: ^{13}C NMR spectrum of **4** (126 MHz, DMSO-d_6)

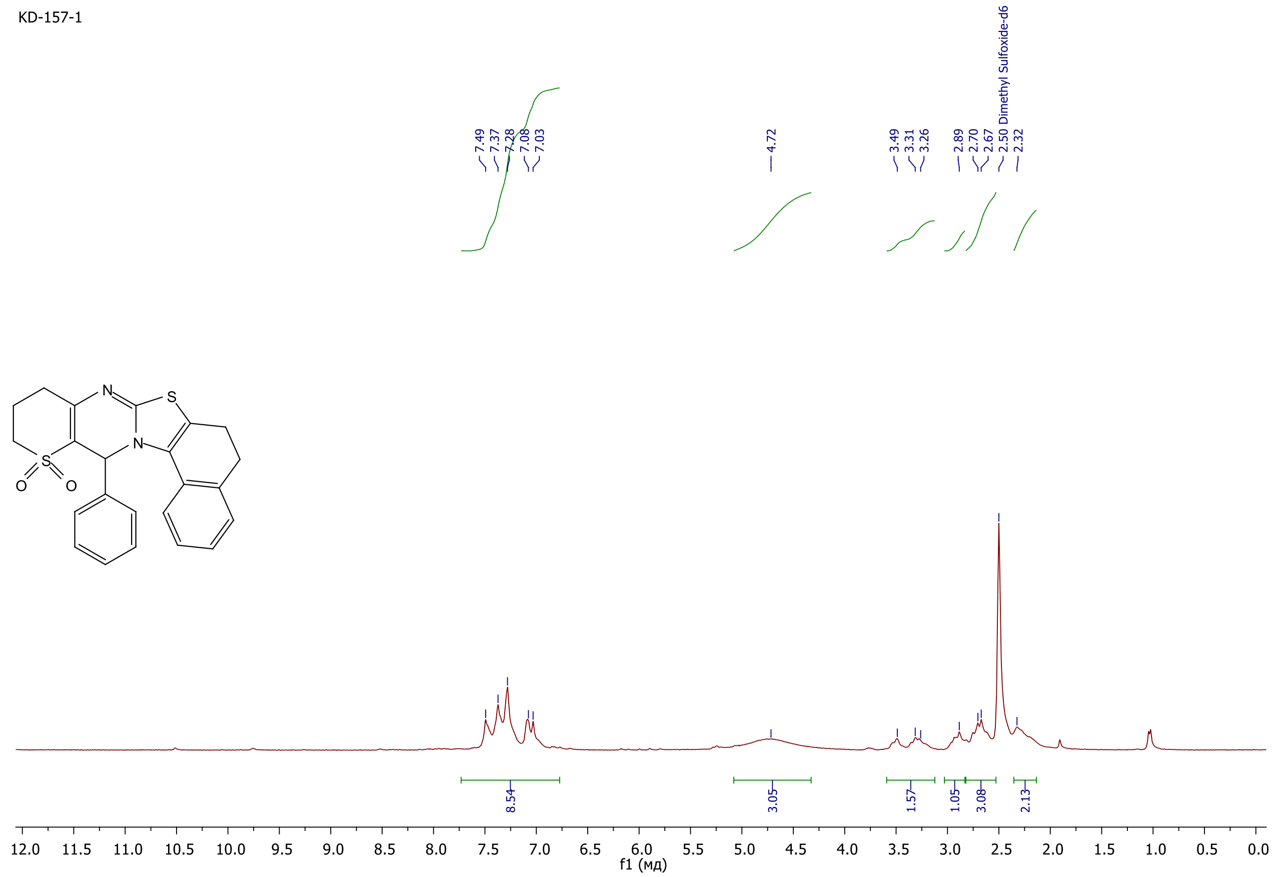

Figure S49: ^{1}H NMR spectrum of **5** (500 MHz, DMSO-d_6)

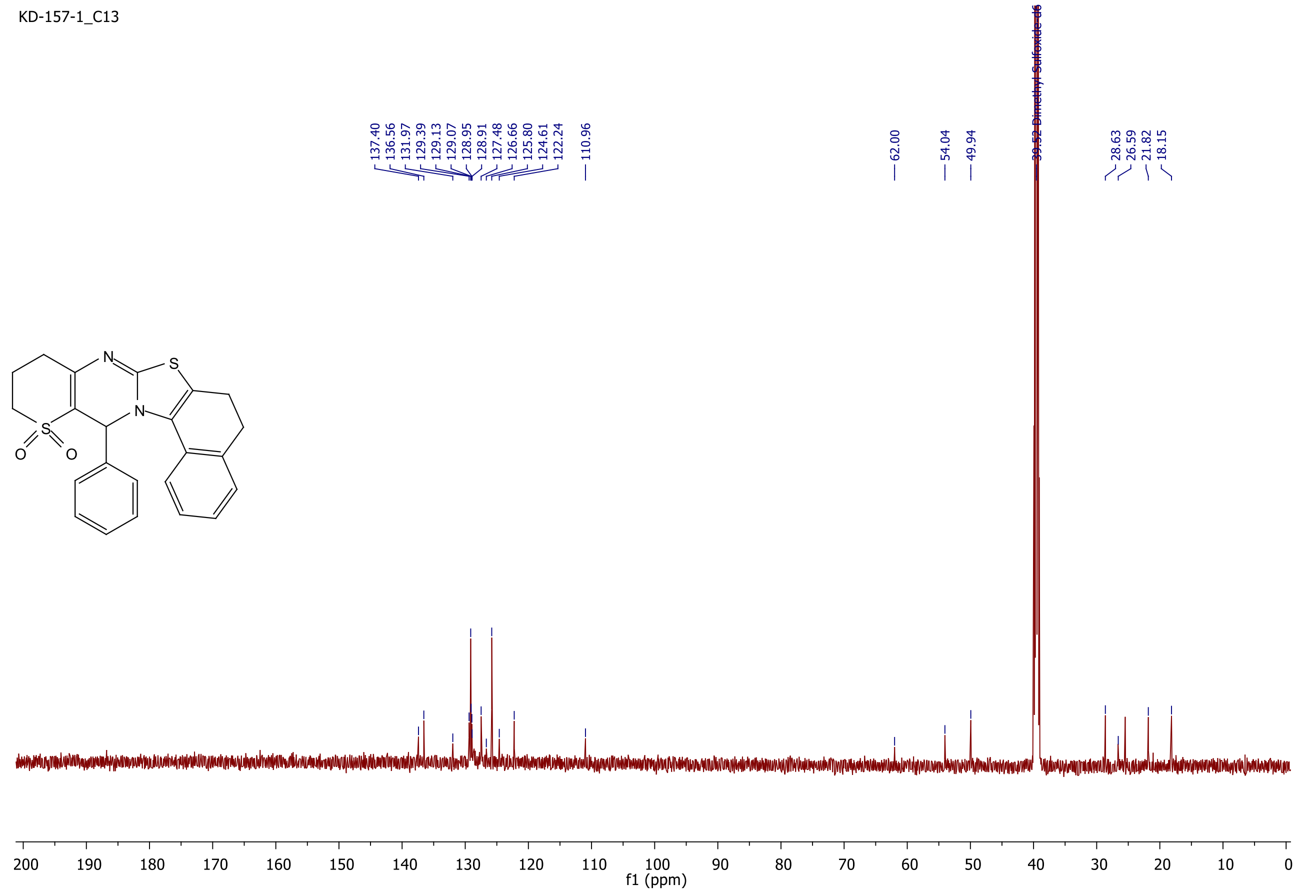

Figure S50: ^{13}C NMR spectrum of **5** (126 MHz, DMSO-d_6)

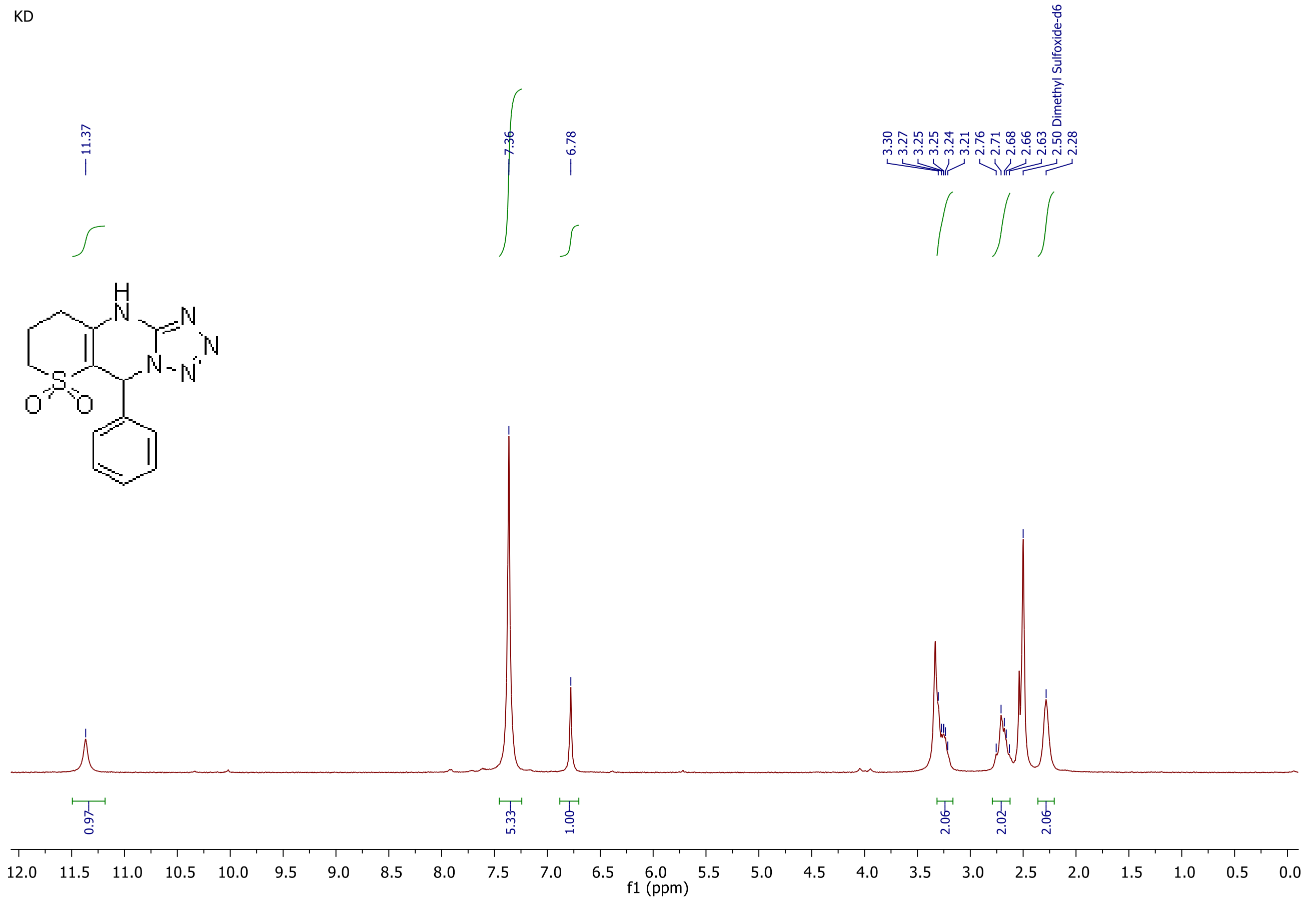

Figure S51: ^{1}H NMR spectrum of **6** (500 MHz, DMSO-d_6)

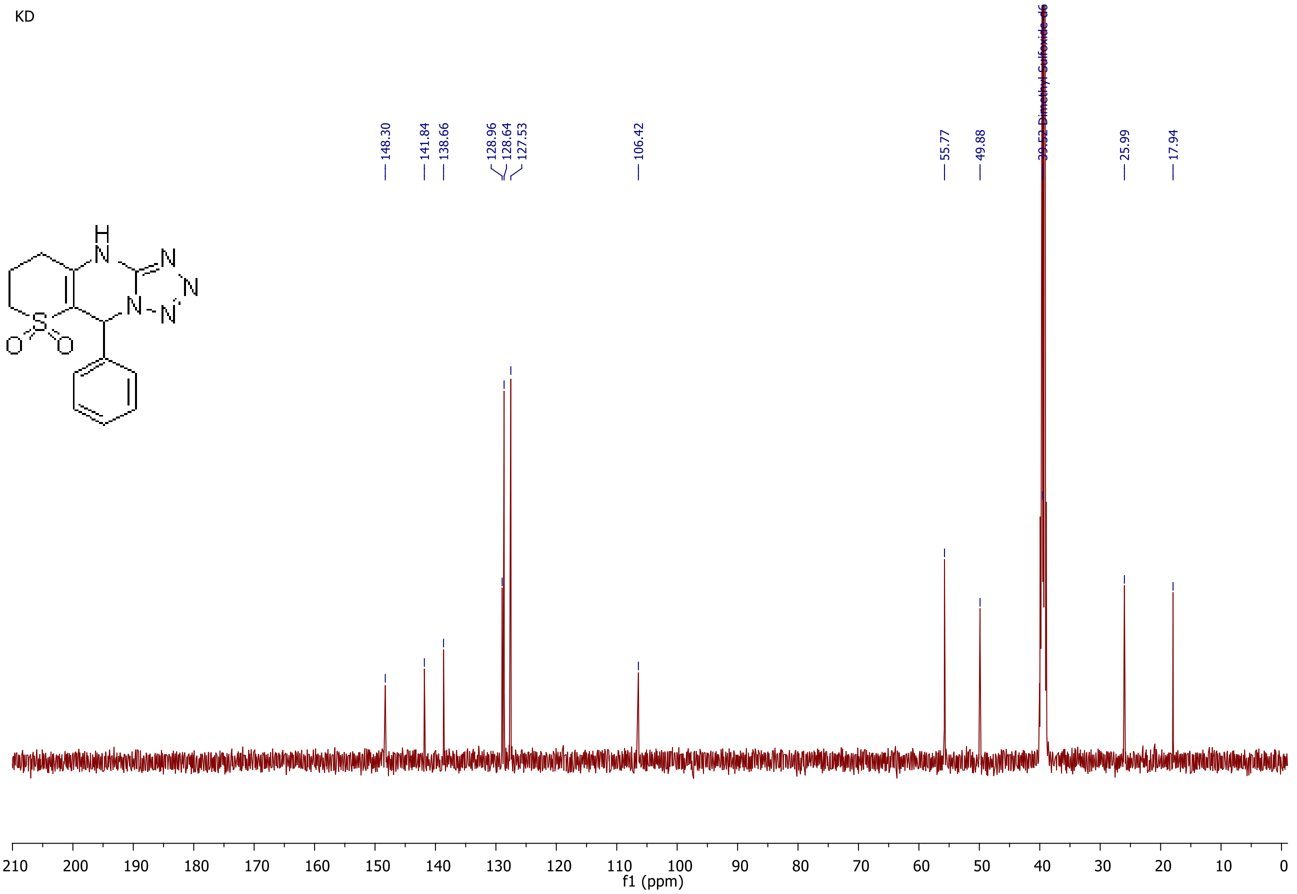

Figure S52: ^{13}C NMR spectrum of **6** (126 MHz, DMSO-d_6)

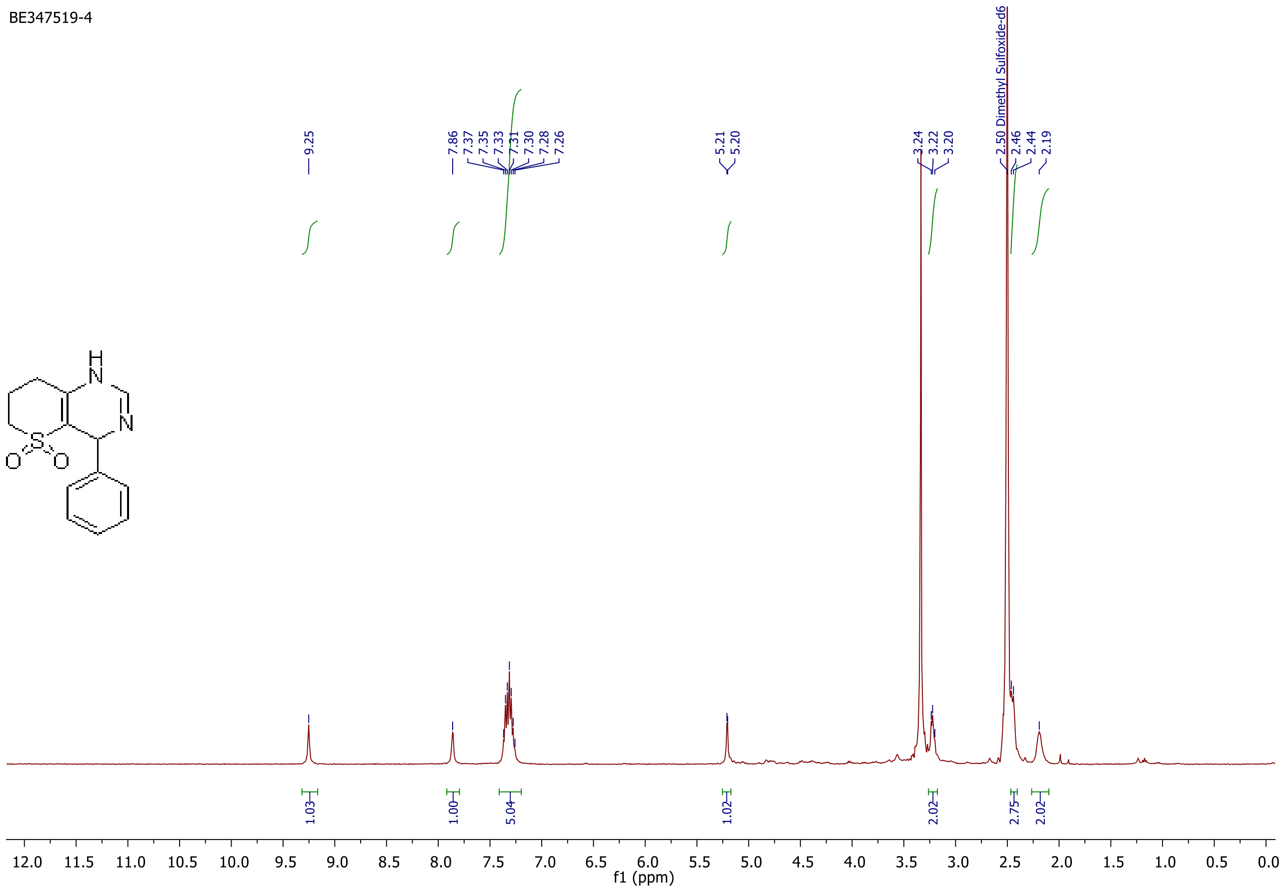

Figure S53: ^{1}H NMR spectrum of **7** (500 MHz, DMSO-*d*6)

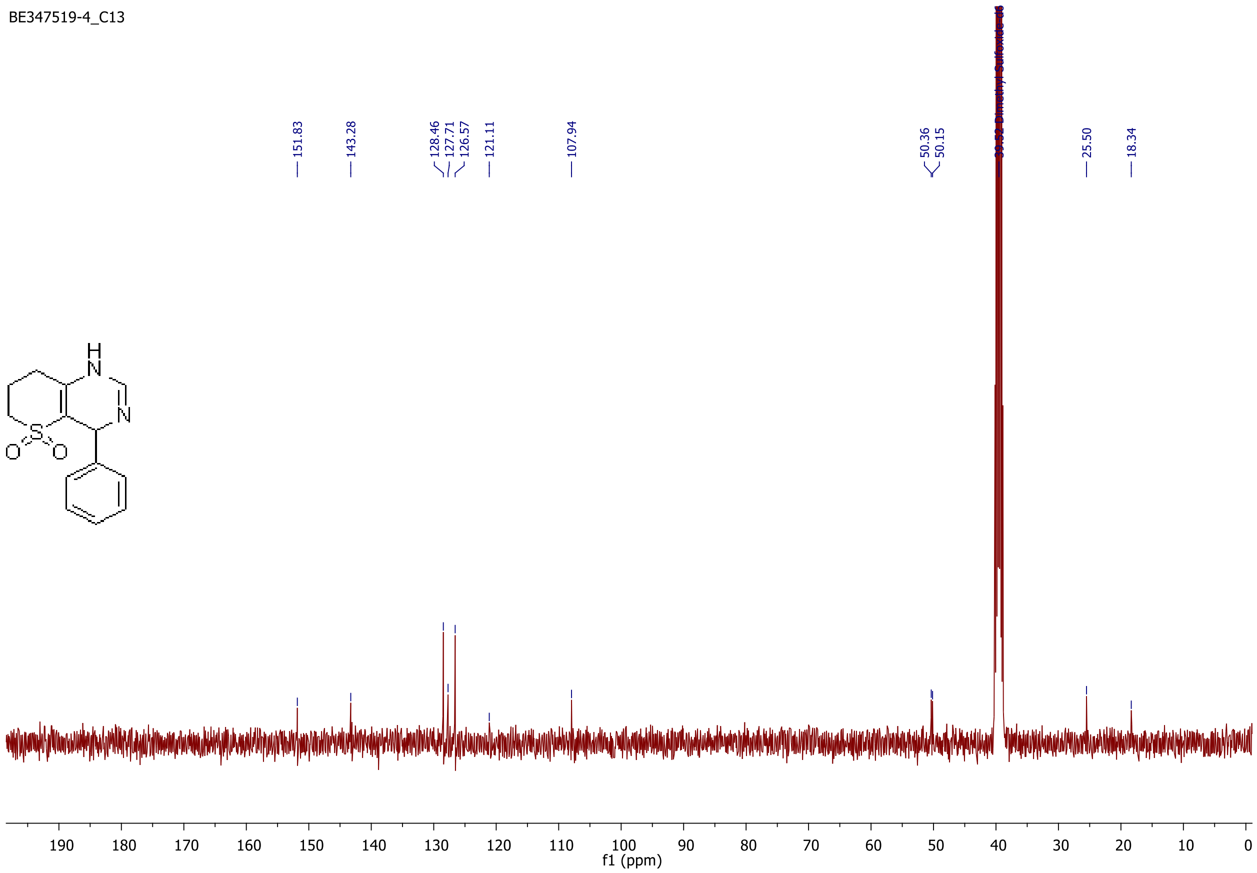

Figure S54: ^{13}C NMR spectrum of **7** (126 MHz, DMSO-*d*6)

Table S1: In silico evaluation of ADMET parameters and biological profile

№	Structure	Log $P_{o/w}$	LD_{50} mg/kg	TSA, Å	Predicted biological profile
**2a**	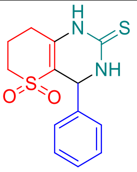	1.60	927 IV class	98.67	*Dengue larvicida (60%), C_albicans (100%), Leishmania braziliensis (60%), Alphis gossypii (100%), Alzheimer – iNOS (80%), Tcruzi_amastigota (60%), Tcruzi_epimastigota (80%)*
**2b**	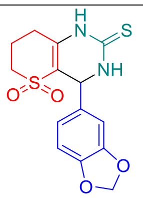	1.45	2000 IV class	117.13	*Dengue larvicida (80%), Sars-Cov (80%), C_albicans (100%), Leishmania amazonensis – Promastigota (80%), Leishmania infantum – Promastigota (100%), Leishmania braziliensis (60%), Alphis gossypii (100%), Alzheimer – iNOS (60%), Lamazonensis_promastigota (60%), Tcruzi_amastigota (80%), Tcruzi_epimastigota (80%)*
**2c**	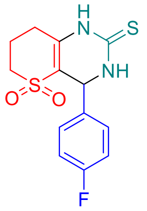	1.92	927 IV class	98.67	*Dengue larvicida (60%), Salmonella (60%), C_albicans (100%), Leishmania braziliensis (80%), Alphis gossypii (100%), Promastigote Ldonovani (80%), Lamazonensis_amastigota (60%), Tripomastigote Chagas (60 %), Tcruzi_amastigota (80%), Tcruzi_trypomastigota (80%), Tcruzi_epimastigota (60%)*
**2d**	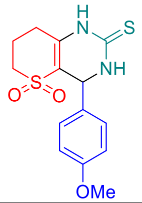	1.60	155 III class	107.90	*Dengue larvicida (80%), C_albicans (100%), Leishmania braziliensis (60%), Alphis gossypii (100%), Tcruzi_amastigota (80%), Tcruzi_epimastigota (80%)*
**2e**	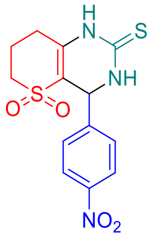	1.20	150 III class	144.49	*Dengue larvicida (80%), C_albicans (100%), Leishmania braziliensis (60%), Leishmania infantum – Promastigota (60%), Leishmania amazonensis – Promastigota (80%), Alphis gossypii (100%), Alzheimer – iNOS (60%), Tripomastigote Chagas (60%), Tcruzi_amastigota (80%), Tcruzi_epimastigota (60%)*
**2f**	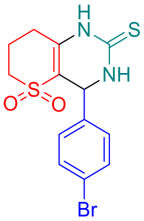	2.23	1000 IV class	98.67	*Dengue larvicida (80%), C_albicans (100%), Leishmania braziliensis (60%), Alphis gossypii (100%), Tcruzi_epimastigota (80%)*
**2g**	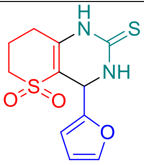	0.92	950 IV class	111.81	*Dengue larvicida (80%), C_albicans (100%), Leishmania braziliensis (60%), Alphis gossypii (100%), Alzheimer – iNOS (80%), Promastigote Ldonovani (60%), Tripomastigote Chagas (60%), Tcruzi_amastigota (100%)*
**2h**	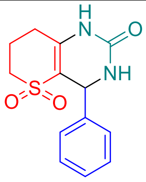	1.08	1000 IV class	83.65	*Dengue larvicida (100%), Alphis gossypii (100%), Tcruzi_epimastigota (100%)*

**2i**	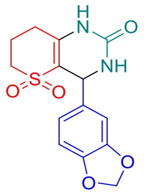	0.94	2000<br><br>IV class	102.11	*Dengue larvicida (100%), Sars-Cov (80%), C_albicans (100%), Leishmania amazonensis – Promastigota (60%), Leishmania infantum – Promastigota (80%), Leishmania major (100%), Alphis gossypii (100%), PTR L major (100%)*
**2j**	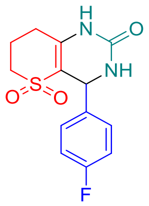	1.41	1000<br><br>IV class	83.65	*Dengue larvicida (80%), Salmonella (60%), C_albicans (100%), Leishmania braziliensis (60%), Alphis gossypii (100%), Promastigote Ldonovani (60%), Epimastigote Chagas (100%), PTR L major (80%), Lamazonensis_amastigota (80%), Tripomastigote Chagas (100%), Tcruzi_epimastigota (80%)*
**2k**	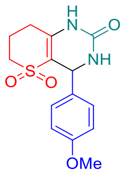	1.09	1000<br><br>IV class	92.88	*Dengue larvicida (100%), C_albicans (100%), Alphis gossypii (100%), Tripomastigote Chagas (100%), Tcruzi_amastigota (60%), Tcruzi_epimastigota (100%)*
**2l**	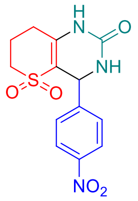	0.57	1000<br><br>IV class	129.47	*Dengue larvicida (100%), C_albicans (100%), Leishmania amazonensis – Promastigota (80%), Hepatite C - RNA dependent (100%), Alphis gossypii (100%), Tripomastigote Chagas (100%), Tcruzi_amastigota (60%), Tcruzi_epimastigota (60%)*
**2m**	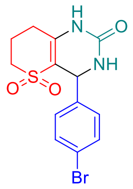	1.72	1000<br><br>IV class	83.65	*Dengue larvicida (100%), C_albicans (100%), Alphis gossypii (100%), Tripomastigote Chagas (80%), Tcruzi_epimastigota (100%)*
**2n**	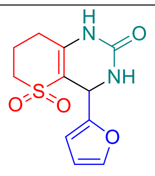	0.43	927<br><br>IV class	96.79	*Dengue larvicida (100%), E_coli (80%), C_albicans (100%), Leishmania braziliensis (80%), Alphis gossypii (100%), Alzheimer - COX2 (60%), Promastigote Ldonovani (60%), Tripomastigote Chagas (80%), Tcruzi_amastigota (100%)*
**2o**	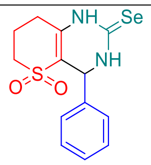	0.60	1000<br><br>IV class	66.58	*Dengue larvicida (80%), C_albicans (100%), Leishmania braziliensis (80%), Alphis gossypii (60%), Tripomastigote Chagas (100%), Tcruzi_epimastigota (80%),*
**2p**	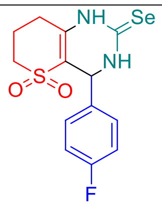	0.90	1000<br><br>IV class	66.58	*Dengue larvicida (60%), C_albicans (100%), Salmonella (60%), Leishmania braziliensis (80%), Alphis gossypii (100%), Promastigote Ldonovani (60%), Tripomastigote Chagas (100%)*

**2q**	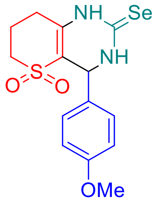	0.54	581<br><br>IV class	75.81	*Dengue larvicida (80%), C_albicans (100%), Leishmania braziliensis (80%), Alphis gossypii (100%), Tripomastigote Chagas (100%)*
**2r**	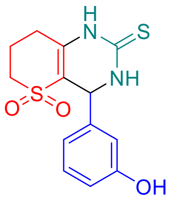	1.19	927<br><br>IV class	118.90	*Dengue larvicida (80%), C_albicans (100%), Leishmania infantum – Promastigota (60%), Alphis gossypii (100%), Alzheimer – iNOS (60%), Tcruzi_epimastigota (80%)*
**3**	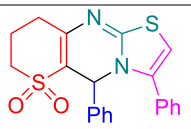	3.76	1000<br><br>IV class	88.05	*Leishmania amazonensis – Promastigota (60%), Alphis gossypii (100%), C_albicans (80%), Leishmania braziliensis (60%), Tcruzi_epimastigota (100%), Tripomastigote Chagas (60%)*
**4**	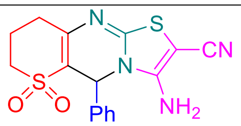	1.98	1000<br><br>IV class	137.88	*Salmonella (60%), C_albicans (80%), Leishmania braziliensis (60%), Alphis gossypii (100%), Alzheimer – iNOS (60%), Tcruzi_trypomastigota (100%), Tcruzi_epimastigota (100%)*
**5**	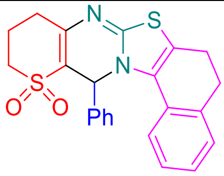	4.11	1000<br><br>IV class	88.05	*Dengue larvicida (60%), Hepatite C - Type1 (60%), C_albicans (80%), Leishmania braziliensis (60%), Alphis gossypii (100%), PTR L major (60%), Tripomastigote Chagas (80%), Tcruzi_epimastigota (100%)*
**6**	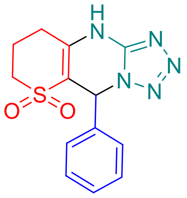	1.17	2025<br><br>V class	98.15	*C_albicans (100%), Alphis gossypii (100%), Tripomastigote Chagas (60%), Tcruzi_epimastigota (60%)*
**7**	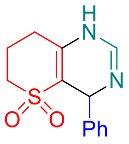	1.46	1000<br><br>IV class	66.91	*Sars-Cov (60%), C_albicans (100%), E_coli (80%), Alphis gossypii (100%), Tripomastigote Chagas (100%), Tcruzi_epimastigota (60%)*

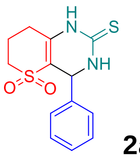

2a O=S1(=O)CCCC2=C1C(NC(=S)N2)C1=CC=CC=C1

Molecule 1

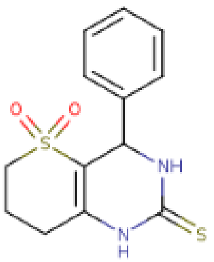

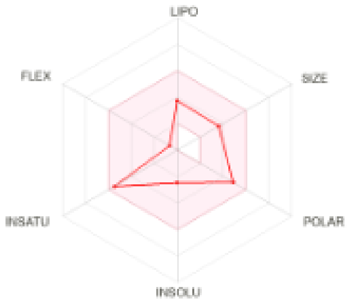

SMILES S=C1NC2=C(C(N1)c1ccccc1)S(=O)(=O)CCC2

Physicochemical Properties	
Formula	C13H14N2O2S2
Molecular weight	294.39 g/mol
Num. heavy atoms	19
Num. arom. heavy atoms	6
Fraction Csp3	0.31
Num. rotatable bonds	1
Num. H-bond acceptors	2
Num. H-bond donors	2
Molar Refractivity	85.74
TPSA	98.67 Å²

Lipophilicity	
Log $P_{o/w}$ (iLOGP)	1.72
Log $P_{o/w}$ (XLOGP3)	1.02
Log $P_{o/w}$ (WLOGP)	1.62
Log $P_{o/w}$ (MLOGP)	1.16
Log $P_{o/w}$ (SILICOS-IT)	2.45
Consensus Log $P_{o/w}$	1.60

Water Solubility	
Log *S* (ESOL)	-2.48
Solubility	9.85e-01 mg/ml ; 3.35e-03 mol/l
Class	Soluble
Log *S* (Ali)	-2.68
Solubility	6.13e-01 mg/ml ; 2.08e-03 mol/l
Class	Soluble
Log *S* (SILICOS-IT)	-4.19
Solubility	1.91e-02 mg/ml ; 6.49e-05 mol/l
Class	Moderately soluble

Pharmacokinetics	
GI absorption	High
BBB permeant	No
P-gp substrate	No
CYP1A2 inhibitor	No
CYP2C19 inhibitor	Yes
CYP2C9 inhibitor	No
CYP2D6 inhibitor	No
CYP3A4 inhibitor	No
Log K_p (skin permeation)	-7.37 cm/s

Druglikeness	
Lipinski	Yes; 0 violation
Ghose	Yes
Veber	Yes
Egan	Yes
Muegge	Yes
Bioavailability Score	0.55

Medicinal Chemistry	
PAINS	0 alert
Brenk	1 alert: thiocarbonyl_group
Leadlikeness	Yes
Synthetic accessibility	3.89

Oral toxicity prediction results for input compound

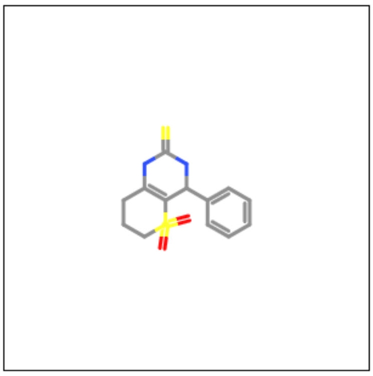

Predicted LD50: 927mg/kg

Predicted Toxicity Class: 4

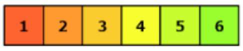

Average similarity: 33.64%

Prediction accuracy: 23%

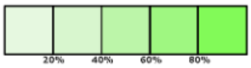

Print Toxicity Report

Name	
Molweight	294.39
Number of hydrogen bond acceptors	5
Number of hydrogen bond donors	2
Number of atoms	19
Number of bonds	21
Number of rotatable bonds	1
Molecular refractivity	85.74
Topological Polar Surface Area	98.67
octanol/water partition coefficient(logP)	3.36

1	Dengue larvicida				
Molecule	Predicted Outcome	Probability	Probability Active	Probability Inactive	Predicted Reliability
O=S1(=O)CCCC2=C1C(NC(=S)N2)C1=CC=CC=C1	Active	0.6	0.6	0.4	reliable

2	Sars-Cov				
Molecule	Predicted Outcome	Probability	Probability Active	Probability Inactive	Predicted Reliability
O=S1(=O)CCCC2=C1C(NC(=S)N2)C1=CC=CC=C1	Inactive	0.6	0.4	0.6	reliable

3	Acetylcholinesterase				
Molecule	Predicted Outcome	Probability	Probability Active	Probability Inactive	Predicted Reliability
O=S1(=O)CCCC2=C1C(NC(=S)N2)C1=CC=CC=C1	Inactive	1.0	0.0	1.0	reliable

4	C_albicans				
Molecule	Predicted Outcome	Probability	Probability Active	Probability Inactive	Predicted Reliability
O=S1(=O)CCCC2=C1C(NC(=S)N2)C1=CC=CC=C1	Active	1.0	1.0	0.0	reliable

5	Salmonella				
Molecule	Predicted Outcome	Probability	Probability Active	Probability Inactive	Predicted Reliability
O=S1(=O)CCCC2=C1C(NC(=S)N2)C1=CC=CC=C1	Inactive	1.0	0.0	1.0	reliable

6	E_coli				
Molecule	Predicted Outcome	Probability	Probability Active	Probability Inactive	Predicted Reliability
O=S1(=O)CCCC2=C1C(NC(=S)N2)C1=CC=CC=C1	Inactive	1.0	0.0	1.0	reliable

7	Hepatite C - NS3-protease helicase				
Molecule	Predicted Outcome	Probability	Probability Active	Probability Inactive	Predicted Reliability
O=S1(=O)CCCC2=C1C(NC(=S)N2)C1=CC=CC=C1	Inactive	1.0	0.0	1.0	reliable

8	Hepatite C - Type1				
Molecule	Predicted Outcome	Probability	Probability Active	Probability Inactive	Predicted Reliability
O=S1(=O)CCCC2=C1C(NC(=S)N2)C1=CC=CC=C1	Inactive	0.8	0.2	0.8	reliable

9	Hepatite C - Serine protease				
Molecule	Predicted Outcome	Probability	Probability Active	Probability Inactive	Predicted Reliability
O=S1(=O)CCCC2=C1C(NC(=S)N2)C1=CC=CC=C1	Inactive	0.8	0.2	0.8	unreliable

10	Hepatite C - RNA dependent				
Molecule	Predicted Outcome	Probability	Probability Active	Probability Inactive	Predicted Reliability
O=S1(=O)CCCC2=C1C(NC(=S)N2)C1=CC=CC=C1	Inactive	1.0	0.0	1.0	reliable

11	Leishmania amazonensis - Promastigota				
Molecule	Predicted Outcome	Probability	Probability Active	Probability Inactive	Predicted Reliability
O=S1(=O)CCCC2=C1C(NC(=S)N2)C1=CC=CC=C1	Inactive	0.8	0.2	0.8	reliable

12	Leishmania infantum - Promastigota				
Molecule	Predicted Outcome	Probability	Probability Active	Probability Inactive	Predicted Reliability
O=S1(=O)CCCC2=C1C(NC(=S)N2)C1=CC=CC=C1	Inactive	0.8	0.2	0.8	reliable

13	Drosophila melanogaster				
Molecule	Predicted Outcome	Probability	Probability Active	Probability Inactive	Predicted Reliability
O=S1(=O)CCCC2=C1C(NC(=S)N2)C1=CC=CC=C1	Inactive	1.0	0.0	1.0	reliable

14	Leishmania braziliensis				
Molecule	Predicted Outcome	Probability	Probability Active	Probability Inactive	Predicted Reliability
O=S1(=O)CCCC2=C1C(NC(=S)N2)C1=CC=CC=C1	Active	0.6	0.6	0.4	reliable

15	Leishmania major				
Molecule	Predicted Outcome	Probability	Probability Active	Probability Inactive	Predicted Reliability
O=S1(=O)CCCC2=C1C(NC(=S)N2)C1=CC=CC=C1	Inactive	1.0	0.0	1.0	reliable

16	Alphis gossypii				
Molecule	Predicted Outcome	Probability	Probability Active	Probability Inactive	Predicted Reliability
O=S1(=O)CCCC2=C1C(NC(=S)N2)C1=CC=CC=C1	Active	1.0	1.0	0.0	reliable

17	Alzheimer - iNOS				
Molecule	Predicted Outcome	Probability	Probability Active	Probability Inactive	Predicted Reliability
O=S1(=O)CCCC2=C1C(NC(=S)N2)C1=CC=CC=C1	Active	0.8	0.8	0.2	reliable

18	Alzheimer - COX2				
Molecule	Predicted Outcome	Probability	Probability Active	Probability Inactive	Predicted Reliability
O=S1(=O)CCCC2=C1C(NC(=S)N2)C1=CC=CC=C1	Inactive	1.0	0.0	1.0	reliable

19	Alzheimer - JNK-3				
Molecule	Predicted Outcome	Probability	Probability Active	Probability Inactive	Predicted Reliability
O=S1(=O)CCCC2=C1C(NC(=S)N2)C1=CC=CC=C1	Inactive	1.0	0.0	1.0	reliable

20	Alzheimer - NADPH				
Molecule	Predicted Outcome	Probability	Probability Active	Probability Inactive	Predicted Reliability
O=S1(=O)CCCC2=C1C(NC(=S)N2)C1=CC=CC=C1	Inactive	1.0	0.0	1.0	reliable

21	Amastigote Chagas				
Molecule	Predicted Outcome	Probability	Probability Active	Probability Inactive	Predicted Reliability
O=S1(=O)CCCC2=C1C(NC(=S)N2)C1=CC=CC=C1	Inactive	1.0	0.0	1.0	reliable

22	Alzheimer - PDE5				
Molecule	Predicted Outcome	Probability	Probability Active	Probability Inactive	Predicted Reliability
O=S1(=O)CCCC2=C1C(NC(=S)N2)C1=CC=CC=C1	Inactive	1.0	0.0	1.0	reliable

23	Epimastigote Chagas				
Molecule	Predicted Outcome	Probability	Probability Active	Probability Inactive	Predicted Reliability
O=S1(=O)CCCC2=C1C(NC(=S)N2)C1=CC=CC=C1	Inactive	1.0	0.0	1.0	reliable

24	Amastigote Ldonovani				
Molecule	Predicted Outcome	Probability	Probability Active	Probability Inactive	Predicted Reliability
O=S1(=O)CCCC2=C1C(NC(=S)N2)C1=CC=CC=C1	Inactive	1.0	0.0	1.0	reliable

25	Promastigote Ldonovani				
Molecule	Predicted Outcome	Probability	Probability Active	Probability Inactive	Predicted Reliability
O=S1(=O)CCCC2=C1C(NC(=S)N2)C1=CC=CC=C1	Inactive	0.6	0.4	0.6	reliable

26	PTR L major				
Molecule	Predicted Outcome	Probability	Probability Active	Probability Inactive	Predicted Reliability
O=S1(=O)CCCC2=C1C(NC(=S)N2)C1=CC=CC=C1	Inactive	1.0	0.0	1.0	reliable

27	Lamazonensis_amastigota				
Molecule	Predicted Outcome	Probability	Probability Active	Probability Inactive	Predicted Reliability
O=S1(=O)CCCC2=C1C(NC(=S)N2)C1=CC=CC=C1	Inactive	1.0	0.0	1.0	reliable

28	Tripomastigote Chagas				
Molecule	Predicted Outcome	Probability	Probability Active	Probability Inactive	Predicted Reliability
O=S1(=O)CCCC2=C1C(NC(=S)N2)C1=CC=CC=C1	Inactive	0.6	0.4	0.6	reliable

29	Lamazonensis_promastigota				
Molecule	Predicted Outcome	Probability	Probability Active	Probability Inactive	Predicted Reliability
O=S1(=O)CCCC2=C1C(NC(=S)N2)C1=CC=CC=C1	Inactive	1.0	0.0	1.0	reliable

30	Tcruzi_amastigota				
Molecule	Predicted Outcome	Probability	Probability Active	Probability Inactive	Predicted Reliability
O=S1(=O)CCCC2=C1C(NC(=S)N2)C1=CC=CC=C1	Active	0.6	0.6	0.4	reliable

31	Tcruzi_epimastigota				
Molecule	Predicted Outcome	Probability	Probability Active	Probability Inactive	Predicted Reliability
O=S1(=O)CCCC2=C1C(NC(=S)N2)C1=CC=CC=C1	Active	0.8	0.8	0.2	reliable

32	Tcruzi_trypomastigota				
Molecule	Predicted Outcome	Probability	Probability Active	Probability Inactive	Predicted Reliability
O=S1(=O)CCCC2=C1C(NC(=S)N2)C1=CC=CC=C1	Inactive	1.0	0.0	1.0	reliable

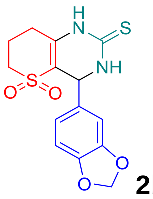

2b O=S1(=O)CCCC2=C1C(NC(=S)N2)C1=CC=C2OCOC2=C1

Molecule 1

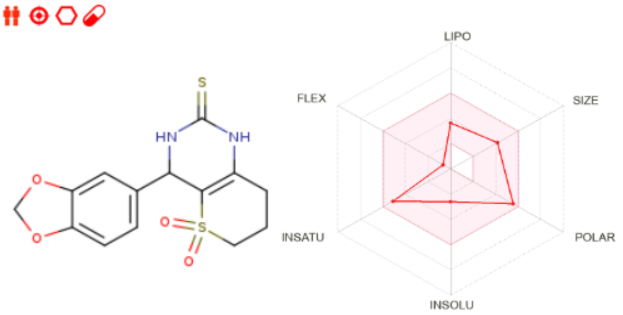

SMILES S=C1NC2=C(C(N1)c1ccc3c(c1)OCO3)S(=O)(=O)CCC2

Physicochemical Properties	
Formula	C14H14N2O4S2
Molecular weight	338.40 g/mol
Num. heavy atoms	22
Num. arom. heavy atoms	6
Fraction Csp3	0.36
Num. rotatable bonds	1
Num. H-bond acceptors	4
Num. H-bond donors	2
Molar Refractivity	91.80
TPSA	117.13 Å²

Lipophilicity	
Log $P_{o/w}$ (iLOGP)	2.00
Log $P_{o/w}$ (XLOGP3)	0.84
Log $P_{o/w}$ (WLOGP)	1.35
Log $P_{o/w}$ (MLOGP)	0.73
Log $P_{o/w}$ (SILICOS-IT)	2.31
Consensus Log $P_{o/w}$	1.45

Water Solubility	
Log *S* (ESOL)	-2.60
Solubility	8.44e-01 mg/ml ; 2.49e-03 mol/l
Class	Soluble
Log *S* (Ali)	-2.88
Solubility	4.43e-01 mg/ml ; 1.31e-03 mol/l
Class	Soluble
Log *S* (SILICOS-IT)	-3.92
Solubility	4.09e-02 mg/ml ; 1.21e-04 mol/l
Class	Soluble

Pharmacokinetics	
GI absorption	High
BBB permeant	No
P-gp substrate	Yes
CYP1A2 inhibitor	No
CYP2C19 inhibitor	Yes
CYP2C9 inhibitor	No
CYP2D6 inhibitor	No
CYP3A4 inhibitor	No
Log K_p (skin permeation)	-7.77 cm/s

Druglikeness	
Lipinski	Yes; 0 violation
Ghose	Yes
Veber	Yes
Egan	Yes
Muegge	Yes
Bioavailability Score	0.55

Medicinal Chemistry	
PAINS	0 alert
Brenk	1 alert: thiocarbonyl_group
Leadlikeness	Yes
Synthetic accessibility	3.95

Oral toxicity prediction results for input compound

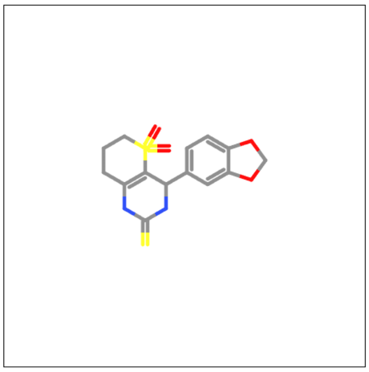

Predicted LD50: 2000mg/kg

Predicted Toxicity Class: 4

1	2	3	4	5	6

Average similarity: 37.23%

Prediction accuracy: 23%

20% 40% 60% 80%

Print Toxicity Report

Name	O=S1(=O)CCCC2=C1C(l
Molweight	338.4
Number of hydrogen bond acceptors	7
Number of hydrogen bond donors	2
Number of atoms	22
Number of bonds	25
Number of rotable bonds	1
Molecular refractivity	91.8
Topological Polar Surface Area	117.13
octanol/water partition coefficient(logP)	3.09

1	Dengue larvicida				
**Molecule**	**Predicted Outcome**	**Probability**	**Probability Active**	**Probability Inactive**	**Predicted Reliability**
O=S1(=O)CCCC2=C1C(NC(=S)N2)C1=CC=C2OCOC2=C1	Active	0.8	0.8	0.2	reliable

2	Sars-Cov				
**Molecule**	**Predicted Outcome**	**Probability**	**Probability Active**	**Probability Inactive**	**Predicted Reliability**
O=S1(=O)CCCC2=C1C(NC(=S)N2)C1=CC=C2OCOC2=C1	Active	0.8	0.8	0.2	reliable

3	C_albicans				
**Molecule**	**Predicted Outcome**	**Probability**	**Probability Active**	**Probability Inactive**	**Predicted Reliability**
O=S1(=O)CCCC2=C1C(NC(=S)N2)C1=CC=C2OCOC2=C1	Active	1.0	1.0	0.0	reliable

4	Acetylcholinesterase				
**Molecule**	**Predicted Outcome**	**Probability**	**Probability Active**	**Probability Inactive**	**Predicted Reliability**
O=S1(=O)CCCC2=C1C(NC(=S)N2)C1=CC=C2OCOC2=C1	Inactive	1.0	0.0	1.0	reliable

5	Salmonella				
**Molecule**	**Predicted Outcome**	**Probability**	**Probability Active**	**Probability Inactive**	**Predicted Reliability**
O=S1(=O)CCCC2=C1C(NC(=S)N2)C1=CC=C2OCOC2=C1	Inactive	1.0	0.0	1.0	unreliable

6	E_coli				
**Molecule**	**Predicted Outcome**	**Probability**	**Probability Active**	**Probability Inactive**	**Predicted Reliability**
O=S1(=O)CCCC2=C1C(NC(=S)N2)C1=CC=C2OCOC2=C1	Inactive	1.0	0.0	1.0	reliable

7	Hepatite C - NS3-protease helicase				
**Molecule**	**Predicted Outcome**	**Probability**	**Probability Active**	**Probability Inactive**	**Predicted Reliability**
O=S1(=O)CCCC2=C1C(NC(=S)N2)C1=CC=C2OCOC2=C1	Inactive	1.0	0.0	1.0	unreliable

8	Hepatite C - Type1				
**Molecule**	**Predicted Outcome**	**Probability**	**Probability Active**	**Probability Inactive**	**Predicted Reliability**
O=S1(=O)CCCC2=C1C(NC(=S)N2)C1=CC=C2OCOC2=C1	Inactive	0.8	0.2	0.8	unreliable

9	Hepatite C - Serine protease				
**Molecule**	**Predicted Outcome**	**Probability**	**Probability Active**	**Probability Inactive**	**Predicted Reliability**
O=S1(=O)CCCC2=C1C(NC(=S)N2)C1=CC=C2OCOC2=C1	Inactive	1.0	0.0	1.0	unreliable

10	Hepatite C - RNA dependent				
**Molecule**	**Predicted Outcome**	**Probability**	**Probability Active**	**Probability Inactive**	**Predicted Reliability**
O=S1(=O)CCCC2=C1C(NC(=S)N2)C1=CC=C2OCOC2=C1	Inactive	1.0	0.0	1.0	unreliable

11	Leishmania amazonensis - Promastigota				
**Molecule**	**Predicted Outcome**	**Probability**	**Probability Active**	**Probability Inactive**	**Predicted Reliability**
O=S1(=O)CCCC2=C1C(NC(=S)N2)C1=CC=C2OCOC2=C1	Active	0.6	0.6	0.4	reliable

12	Leishmania infantum - Promastigota				
**Molecule**	**Predicted Outcome**	**Probability**	**Probability Active**	**Probability Inactive**	**Predicted Reliability**
O=S1(=O)CCCC2=C1C(NC(=S)N2)C1=CC=C2OCOC2=C1	Active	0.8	0.8	0.2	reliable

13	Drosophila melanogaster				
**Molecule**	**Predicted Outcome**	**Probability**	**Probability Active**	**Probability Inactive**	**Predicted Reliability**
O=S1(=O)CCCC2=C1C(NC(=S)N2)C1=CC=C2OCOC2=C1	Inactive	1.0	0.0	1.0	unreliable

14	Leishmania braziliensis				
**Molecule**	**Predicted Outcome**	**Probability**	**Probability Active**	**Probability Inactive**	**Predicted Reliability**
O=S1(=O)CCCC2=C1C(NC(=S)N2)C1=CC=C2OCOC2=C1	Active	0.6	0.6	0.4	reliable

15	Leishmania major				
**Molecule**	**Predicted Outcome**	**Probability**	**Probability Active**	**Probability Inactive**	**Predicted Reliability**
O=S1(=O)CCCC2=C1C(NC(=S)N2)C1=CC=C2OCOC2=C1	Inactive	0.8	0.2	0.8	reliable

16	Alphis gossypii				
Molecule	Predicted Outcome	Probability	Probability Active	Probability Inactive	Predicted Reliability
O=S1(=O)CCCC2=C1C(NC(=S)N2)C1=CC=C2OCOC2=C1	Active	1.0	1.0	0.0	unreliable

17	Alzheimer - iNOS				
Molecule	Predicted Outcome	Probability	Probability Active	Probability Inactive	Predicted Reliability
O=S1(=O)CCCC2=C1C(NC(=S)N2)C1=CC=C2OCOC2=C1	Active	0.6	0.6	0.4	reliable

18	Alzheimer - JNK-3				
Molecule	Predicted Outcome	Probability	Probability Active	Probability Inactive	Predicted Reliability
O=S1(=O)CCCC2=C1C(NC(=S)N2)C1=CC=C2OCOC2=C1	Inactive	1.0	0.0	1.0	reliable

19	Alzheimer - COX2				
Molecule	Predicted Outcome	Probability	Probability Active	Probability Inactive	Predicted Reliability
O=S1(=O)CCCC2=C1C(NC(=S)N2)C1=CC=C2OCOC2=C1	Inactive	1.0	0.0	1.0	reliable

20	Alzheimer - NADPH				
Molecule	Predicted Outcome	Probability	Probability Active	Probability Inactive	Predicted Reliability
O=S1(=O)CCCC2=C1C(NC(=S)N2)C1=CC=C2OCOC2=C1	Inactive	1.0	0.0	1.0	reliable

21	Alzheimer - PDE5				
Molecule	Predicted Outcome	Probability	Probability Active	Probability Inactive	Predicted Reliability
O=S1(=O)CCCC2=C1C(NC(=S)N2)C1=CC=C2OCOC2=C1	Inactive	1.0	0.0	1.0	reliable

22	Amastigote Ldonovani				
Molecule	Predicted Outcome	Probability	Probability Active	Probability Inactive	Predicted Reliability
O=S1(=O)CCCC2=C1C(NC(=S)N2)C1=CC=C2OCOC2=C1	Inactive	1.0	0.0	1.0	reliable

23	Amastigote Chagas				
Molecule	Predicted Outcome	Probability	Probability Active	Probability Inactive	Predicted Reliability
O=S1(=O)CCCC2=C1C(NC(=S)N2)C1=CC=C2OCOC2=C1	Inactive	1.0	0.0	1.0	reliable

24	Epimastigote Chagas				
Molecule	Predicted Outcome	Probability	Probability Active	Probability Inactive	Predicted Reliability
O=S1(=O)CCCC2=C1C(NC(=S)N2)C1=CC=C2OCOC2=C1	Inactive	1.0	0.0	1.0	reliable

25	PTR L major				
Molecule	Predicted Outcome	Probability	Probability Active	Probability Inactive	Predicted Reliability
O=S1(=O)CCCC2=C1C(NC(=S)N2)C1=CC=C2OCOC2=C1	Inactive	0.6	0.4	0.6	reliable

26	Promastigote Ldonovani				
Molecule	Predicted Outcome	Probability	Probability Active	Probability Inactive	Predicted Reliability
O=S1(=O)CCCC2=C1C(NC(=S)N2)C1=CC=C2OCOC2=C1	Inactive	0.8	0.2	0.8	reliable

27	Lamazonensis_amastigota				
Molecule	Predicted Outcome	Probability	Probability Active	Probability Inactive	Predicted Reliability
O=S1(=O)CCCC2=C1C(NC(=S)N2)C1=CC=C2OCOC2=C1	Inactive	1.0	0.0	1.0	reliable

28	Tripomastigote Chagas				
Molecule	Predicted Outcome	Probability	Probability Active	Probability Inactive	Predicted Reliability
O=S1(=O)CCCC2=C1C(NC(=S)N2)C1=CC=C2OCOC2=C1	Inactive	1.0	0.0	1.0	reliable

29	Lamazonensis_promastigota				
Molecule	Predicted Outcome	Probability	Probability Active	Probability Inactive	Predicted Reliability
O=S1(=O)CCCC2=C1C(NC(=S)N2)C1=CC=C2OCOC2=C1	Active	0.6	0.6	0.4	reliable

30	Tcruzi_amastigota				
Molecule	Predicted Outcome	Probability	Probability Active	Probability Inactive	Predicted Reliability
O=S1(=O)CCCC2=C1C(NC(=S)N2)C1=CC=C2OCOC2=C1	Active	0.8	0.8	0.2	reliable

31	Tcruzi_epimastigota				
Molecule	Predicted Outcome	Probability	Probability Active	Probability Inactive	Predicted Reliability
O=S1(=O)CCCC2=C1C(NC(=S)N2)C1=CC=C2OCOC2=C1	Active	0.8	0.8	0.2	reliable

32	Tcruzi_trypomastigota				
Molecule	Predicted Outcome	Probability	Probability Active	Probability Inactive	Predicted Reliability
O=S1(=O)CCCC2=C1C(NC(=S)N2)C1=CC=C2OCOC2=C1	Inactive	0.6	0.4	0.6	reliable

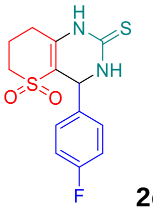

2c FC1=CC=C(C=C1)C1NC(=S)NC2=C1S(=O)(=O)CCC2

Molecule 1

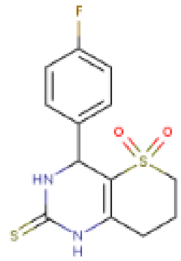

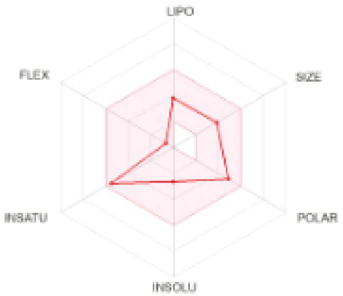

SMILES S=C1NC2=C(C(N1)c1ccc(cc1)F)S(=O)(=O)CCC2

Physicochemical Properties	
Formula	C13H13FN2O2S2
Molecular weight	312.38 g/mol
Num. heavy atoms	20
Num. arom. heavy atoms	6
Fraction Csp3	0.31
Num. rotatable bonds	1
Num. H-bond acceptors	3
Num. H-bond donors	2
Molar Refractivity	85.69
TPSA	98.67 Å²
**Lipophilicity**	
Log $P_{o/w}$ (iLOGP)	1.86
Log $P_{o/w}$ (XLOGP3)	1.12
Log $P_{o/w}$ (WLOGP)	2.18
Log $P_{o/w}$ (MLOGP)	1.57
Log $P_{o/w}$ (SILICOS-IT)	2.86
Consensus Log $P_{o/w}$	1.92

Water Solubility	
Log *S* (ESOL)	-2.64
Solubility	7.18e-01 mg/ml ; 2.30e-03 mol/l
Class	Soluble
Log *S* (Ali)	-2.79
Solubility	5.12e-01 mg/ml ; 1.64e-03 mol/l
Class	Soluble
Log *S* (SILICOS-IT)	-4.46
Solubility	1.09e-02 mg/ml ; 3.48e-05 mol/l
Class	Moderately soluble
**Pharmacokinetics**	
GI absorption	High
BBB permeant	No
P-gp substrate	No
CYP1A2 inhibitor	No
CYP2C19 inhibitor	Yes
CYP2C9 inhibitor	No
CYP2D6 inhibitor	No
CYP3A4 inhibitor	No
Log K_p (skin permeation)	-7.41 cm/s
**Druglikeness**	
Lipinski	Yes; 0 violation
Ghose	Yes
Veber	Yes
Egan	Yes
Muegge	Yes
Bioavailability Score	0.55
**Medicinal Chemistry**	
PAINS	0 alert
Brenk	1 alert: thiocarbonyl_group
Leadlikeness	Yes
Synthetic accessibility	3.88

Oral toxicity prediction results for input compound

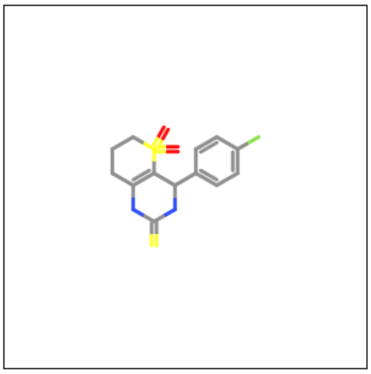

Predicted LD50: 927mg/kg

Predicted Toxicity Class: 4

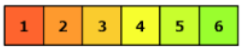

Average similarity: 32.75%

Prediction accuracy: 23%

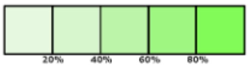

Print Toxicity Report

Name	
Molweight	312.38
Number of hydrogen bond acceptors	5
Number of hydrogen bond donors	2
Number of atoms	20
Number of bonds	22
Number of rotatable bonds	1
Molecular refractivity	85.69
Topological Polar Surface Area	98.67
octanol/water partition coefficient(logP)	3.5

1	Dengue larvicida				
**Molecule**	**Predicted Outcome**	**Probability**	**Probability Active**	**Probability Inactive**	**Predicted Reliability**
FC1=CC=C(C=C1)C1NC(=S)NC2=C1S(=O)(=O)CCC2	Active	0.6	0.6	0.4	reliable

2	Sars-Cov				
**Molecule**	**Predicted Outcome**	**Probability**	**Probability Active**	**Probability Inactive**	**Predicted Reliability**
FC1=CC=C(C=C1)C1NC(=S)NC2=C1S(=O)(=O)CCC2	Inactive	0.8	0.2	0.8	reliable

3	Salmonella				
**Molecule**	**Predicted Outcome**	**Probability**	**Probability Active**	**Probability Inactive**	**Predicted Reliability**
FC1=CC=C(C=C1)C1NC(=S)NC2=C1S(=O)(=O)CCC2	Active	0.6	0.6	0.4	reliable

4	Acetylcholinesterase				
**Molecule**	**Predicted Outcome**	**Probability**	**Probability Active**	**Probability Inactive**	**Predicted Reliability**
FC1=CC=C(C=C1)C1NC(=S)NC2=C1S(=O)(=O)CCC2	Inactive	1.0	0.0	1.0	reliable

5	C_albicans				
**Molecule**	**Predicted Outcome**	**Probability**	**Probability Active**	**Probability Inactive**	**Predicted Reliability**
FC1=CC=C(C=C1)C1NC(=S)NC2=C1S(=O)(=O)CCC2	Active	1.0	1.0	0.0	reliable

6	E_coli				
**Molecule**	**Predicted Outcome**	**Probability**	**Probability Active**	**Probability Inactive**	**Predicted Reliability**
FC1=CC=C(C=C1)C1NC(=S)NC2=C1S(=O)(=O)CCC2	Inactive	1.0	0.0	1.0	reliable

7	Hepatite C - Type1				
**Molecule**	**Predicted Outcome**	**Probability**	**Probability Active**	**Probability Inactive**	**Predicted Reliability**
FC1=CC=C(C=C1)C1NC(=S)NC2=C1S(=O)(=O)CCC2	Inactive	0.8	0.2	0.8	reliable

8	Hepatite C - NS3-protease helicase				
**Molecule**	**Predicted Outcome**	**Probability**	**Probability Active**	**Probability Inactive**	**Predicted Reliability**
FC1=CC=C(C=C1)C1NC(=S)NC2=C1S(=O)(=O)CCC2	Inactive	1.0	0.0	1.0	reliable

9	Hepatite C - Serine protease				
**Molecule**	**Predicted Outcome**	**Probability**	**Probability Active**	**Probability Inactive**	**Predicted Reliability**
FC1=CC=C(C=C1)C1NC(=S)NC2=C1S(=O)(=O)CCC2	Inactive	1.0	0.0	1.0	unreliable

10	Hepatite C - RNA dependent				
**Molecule**	**Predicted Outcome**	**Probability**	**Probability Active**	**Probability Inactive**	**Predicted Reliability**
FC1=CC=C(C=C1)C1NC(=S)NC2=C1S(=O)(=O)CCC2	Inactive	1.0	0.0	1.0	reliable

11	Leishmania amazonensis - Promastigota				
**Molecule**	**Predicted Outcome**	**Probability**	**Probability Active**	**Probability Inactive**	**Predicted Reliability**
FC1=CC=C(C=C1)C1NC(=S)NC2=C1S(=O)(=O)CCC2	Inactive	0.6	0.4	0.6	reliable

12	Leishmania infantum - Promastigota				
**Molecule**	**Predicted Outcome**	**Probability**	**Probability Active**	**Probability Inactive**	**Predicted Reliability**
FC1=CC=C(C=C1)C1NC(=S)NC2=C1S(=O)(=O)CCC2	Inactive	0.8	0.2	0.8	reliable

13	Leishmania braziliensis				
Molecule	Predicted Outcome	Probability	Probability Active	Probability Inactive	Predicted Reliability
FC1=CC=C(C=C1)C1NC(=S)NC2=C1S(=O)(=O)CCC2	Active	0.8	0.8	0.2	reliable

14	Drosophila melanogaster				
Molecule	Predicted Outcome	Probability	Probability Active	Probability Inactive	Predicted Reliability
FC1=CC=C(C=C1)C1NC(=S)NC2=C1S(=O)(=O)CCC2	Inactive	1.0	0.0	1.0	unreliable

15	Leishmania major				
Molecule	Predicted Outcome	Probability	Probability Active	Probability Inactive	Predicted Reliability
FC1=CC=C(C=C1)C1NC(=S)NC2=C1S(=O)(=O)CCC2	Inactive	1.0	0.0	1.0	reliable

16	Alphis gossypii				
Molecule	Predicted Outcome	Probability	Probability Active	Probability Inactive	Predicted Reliability
FC1=CC=C(C=C1)C1NC(=S)NC2=C1S(=O)(=O)CCC2	Active	1.0	1.0	0.0	reliable

17	Alzheimer - iNOS				
Molecule	Predicted Outcome	Probability	Probability Active	Probability Inactive	Predicted Reliability
FC1=CC=C(C=C1)C1NC(=S)NC2=C1S(=O)(=O)CCC2	Inactive	0.8	0.2	0.8	reliable

18	Alzheimer - COX2				
Molecule	Predicted Outcome	Probability	Probability Active	Probability Inactive	Predicted Reliability
FC1=CC=C(C=C1)C1NC(=S)NC2=C1S(=O)(=O)CCC2	Inactive	1.0	0.0	1.0	reliable

19	Alzheimer - NADPH				
Molecule	Predicted Outcome	Probability	Probability Active	Probability Inactive	Predicted Reliability
FC1=CC=C(C=C1)C1NC(=S)NC2=C1S(=O)(=O)CCC2	Inactive	1.0	0.0	1.0	reliable

20	Alzheimer - JNK-3				
Molecule	Predicted Outcome	Probability	Probability Active	Probability Inactive	Predicted Reliability
FC1=CC=C(C=C1)C1NC(=S)NC2=C1S(=O)(=O)CCC2	Inactive	1.0	0.0	1.0	reliable

21	Alzheimer - PDE5				
Molecule	Predicted Outcome	Probability	Probability Active	Probability Inactive	Predicted Reliability
FC1=CC=C(C=C1)C1NC(=S)NC2=C1S(=O)(=O)CCC2	Inactive	0.8	0.2	0.8	reliable

22	Amastigote Ldonovani				
Molecule	Predicted Outcome	Probability	Probability Active	Probability Inactive	Predicted Reliability
FC1=CC=C(C=C1)C1NC(=S)NC2=C1S(=O)(=O)CCC2	Inactive	1.0	0.0	1.0	reliable

23	Amastigote Chagas				
Molecule	Predicted Outcome	Probability	Probability Active	Probability Inactive	Predicted Reliability
FC1=CC=C(C=C1)C1NC(=S)NC2=C1S(=O)(=O)CCC2	Inactive	1.0	0.0	1.0	reliable

24	Promastigote Ldonovani				
Molecule	Predicted Outcome	Probability	Probability Active	Probability Inactive	Predicted Reliability
FC1=CC=C(C=C1)C1NC(=S)NC2=C1S(=O)(=O)CCC2	Active	0.8	0.8	0.2	reliable

25	Epimastigote Chagas				
Molecule	Predicted Outcome	Probability	Probability Active	Probability Inactive	Predicted Reliability
FC1=CC=C(C=C1)C1NC(=S)NC2=C1S(=O)(=O)CCC2	Inactive	1.0	0.0	1.0	reliable

26	Lamazonensis_amastigota				
Molecule	Predicted Outcome	Probability	Probability Active	Probability Inactive	Predicted Reliability
FC1=CC=C(C=C1)C1NC(=S)NC2=C1S(=O)(=O)CCC2	Active	0.6	0.6	0.4	reliable

27	PTR L major				
Molecule	Predicted Outcome	Probability	Probability Active	Probability Inactive	Predicted Reliability
FC1=CC=C(C=C1)C1NC(=S)NC2=C1S(=O)(=O)CCC2	Inactive	1.0	0.0	1.0	reliable

28	Tripomastigote Chagas				
Molecule	Predicted Outcome	Probability	Probability Active	Probability Inactive	Predicted Reliability
FC1=CC=C(C=C1)C1NC(=S)NC2=C1S(=O)(=O)CCC2	Active	0.6	0.6	0.4	reliable

29	Lamazonensis_promastigota				
**Molecule**	**Predicted Outcome**	**Probability**	**Probability Active**	**Probability Inactive**	**Predicted Reliability**
FC1=CC=C(C=C1)C1NC(=S)NC2=C1S(=O)(=O)CCC2	Inactive	1.0	0.0	1.0	reliable

30	Tcruzi_amastigota				
**Molecule**	**Predicted Outcome**	**Probability**	**Probability Active**	**Probability Inactive**	**Predicted Reliability**
FC1=CC=C(C=C1)C1NC(=S)NC2=C1S(=O)(=O)CCC2	Active	0.8	0.8	0.2	reliable

31	Tcruzi_trypomastigota				
**Molecule**	**Predicted Outcome**	**Probability**	**Probability Active**	**Probability Inactive**	**Predicted Reliability**
FC1=CC=C(C=C1)C1NC(=S)NC2=C1S(=O)(=O)CCC2	Active	0.6	0.6	0.4	reliable

32	Tcruzi_epimastigota				
**Molecule**	**Predicted Outcome**	**Probability**	**Probability Active**	**Probability Inactive**	**Predicted Reliability**
FC1=CC=C(C=C1)C1NC(=S)NC2=C1S(=O)(=O)CCC2	Active	0.6	0.6	0.4	reliable

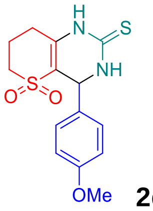

2d COC1=CC=C(C=C1)C1NC(=S)NC2=C1S(=O)(=O)CCC2

Molecule 1

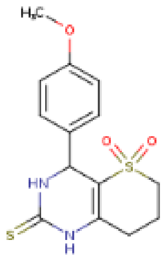

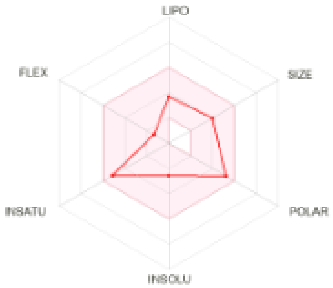

SMILES COc1ccc(cc1)C1NC(=S)NC2=C1S(=O)(=O)CCC2

Physicochemical Properties	
Formula	C14H16N2O3S2
Molecular weight	324.42 g/mol
Num. heavy atoms	21
Num. arom. heavy atoms	6
Fraction Csp3	0.36
Num. rotatable bonds	2
Num. H-bond acceptors	3
Num. H-bond donors	2
Molar Refractivity	92.23
TPSA	107.90 Å²

Lipophilicity	
Log $P_{o/w}$ (iLOGP)	2.03
Log $P_{o/w}$ (XLOGP3)	1.00
Log $P_{o/w}$ (WLOGP)	1.63
Log $P_{o/w}$ (MLOGP)	0.87
Log $P_{o/w}$ (SILICOS-IT)	2.46
Consensus Log $P_{o/w}$	1.60

Water Solubility	
Log *S* (ESOL)	-2.56
Solubility	8.92e-01 mg/ml ; 2.75e-03 mol/l
Class	Soluble
Log *S* (Ali)	-2.85
Solubility	4.53e-01 mg/ml ; 1.40e-03 mol/l
Class	Soluble
Log *S* (SILICOS-IT)	-4.30
Solubility	1.62e-02 mg/ml ; 5.00e-05 mol/l
Class	Moderately soluble

Pharmacokinetics	
GI absorption	High
BBB permeant	No
P-gp substrate	Yes
CYP1A2 inhibitor	No
CYP2C19 inhibitor	Yes
CYP2C9 inhibitor	No
CYP2D6 inhibitor	No
CYP3A4 inhibitor	No
Log K_p (skin permeation)	-7.57 cm/s

Druglikeness	
Lipinski	Yes; 0 violation
Ghose	Yes
Veber	Yes
Egan	Yes
Muegge	Yes
Bioavailability Score	0.55

Medicinal Chemistry	
PAINS	0 alert
Brenk	1 alert: thiocarbonyl_group
Leadlikeness	Yes
Synthetic accessibility	3.92

Oral toxicity prediction results for input compound

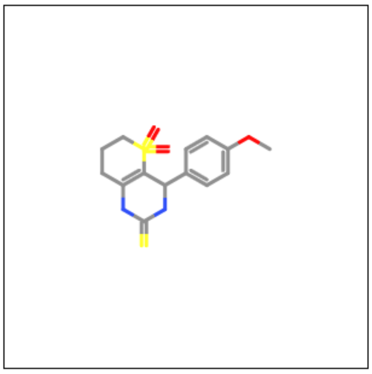

Predicted LD50: 155mg/kg

Predicted Toxicity Class: 3

1	2	3	4	5	6

Average similarity: 34.18%

Prediction accuracy: 23%

20% 40% 60% 80%

Print Toxicity Report

Name	COC1=CC=C(C=C1)C1N (=O)CCC2
Molweight	324.42
Number of hydrogen bond acceptors	6
Number of hydrogen bond donors	2
Number of atoms	21
Number of bonds	23
Number of rotable bonds	2
Molecular refractivity	92.23
Topological Polar Surface Area	107.9
octanol/water partition coefficient(logP)	3.37

1	Dengue larvicida				
**Molecule**	**Predicted Outcome**	**Probability**	**Probability Active**	**Probability Inactive**	**Predicted Reliability**
COC1=CC=C(C=C1)C1NC(=S)NC2=C1S(=O)(=O)CCC2	Active	0.8	0.8	0.2	reliable

2	Sars-Cov				
**Molecule**	**Predicted Outcome**	**Probability**	**Probability Active**	**Probability Inactive**	**Predicted Reliability**
COC1=CC=C(C=C1)C1NC(=S)NC2=C1S(=O)(=O)CCC2	Inactive	0.8	0.2	0.8	reliable

3	Salmonella				
**Molecule**	**Predicted Outcome**	**Probability**	**Probability Active**	**Probability Inactive**	**Predicted Reliability**
COC1=CC=C(C=C1)C1NC(=S)NC2=C1S(=O)(=O)CCC2	Inactive	1.0	0.0	1.0	reliable

4	Acetylcholinesterase				
**Molecule**	**Predicted Outcome**	**Probability**	**Probability Active**	**Probability Inactive**	**Predicted Reliability**
COC1=CC=C(C=C1)C1NC(=S)NC2=C1S(=O)(=O)CCC2	Inactive	1.0	0.0	1.0	reliable

5	Hepatite C - Type1				
**Molecule**	**Predicted Outcome**	**Probability**	**Probability Active**	**Probability Inactive**	**Predicted Reliability**
COC1=CC=C(C=C1)C1NC(=S)NC2=C1S(=O)(=O)CCC2	Inactive	0.8	0.2	0.8	unreliable

6	E_coli				
**Molecule**	**Predicted Outcome**	**Probability**	**Probability Active**	**Probability Inactive**	**Predicted Reliability**
COC1=CC=C(C=C1)C1NC(=S)NC2=C1S(=O)(=O)CCC2	Inactive	0.8	0.2	0.8	reliable

7	Hepatite C - NS3-protease helicase				
**Molecule**	**Predicted Outcome**	**Probability**	**Probability Active**	**Probability Inactive**	**Predicted Reliability**
COC1=CC=C(C=C1)C1NC(=S)NC2=C1S(=O)(=O)CCC2	Inactive	1.0	0.0	1.0	reliable

8	C_albicans				
**Molecule**	**Predicted Outcome**	**Probability**	**Probability Active**	**Probability Inactive**	**Predicted Reliability**
COC1=CC=C(C=C1)C1NC(=S)NC2=C1S(=O)(=O)CCC2	Active	1.0	1.0	0.0	reliable

9	Leishmania amazonensis - Promastigota				
**Molecule**	**Predicted Outcome**	**Probability**	**Probability Active**	**Probability Inactive**	**Predicted Reliability**
COC1=CC=C(C=C1)C1NC(=S)NC2=C1S(=O)(=O)CCC2	Inactive	0.6	0.4	0.6	reliable

10	Hepatite C - RNA dependent				
**Molecule**	**Predicted Outcome**	**Probability**	**Probability Active**	**Probability Inactive**	**Predicted Reliability**
COC1=CC=C(C=C1)C1NC(=S)NC2=C1S(=O)(=O)CCC2	Inactive	1.0	0.0	1.0	reliable

11	Hepatite C - Serine protease				
**Molecule**	**Predicted Outcome**	**Probability**	**Probability Active**	**Probability Inactive**	**Predicted Reliability**
COC1=CC=C(C=C1)C1NC(=S)NC2=C1S(=O)(=O)CCC2	Inactive	1.0	0.0	1.0	unreliable

12	Leishmania infantum - Promastigota				
Molecule	Predicted Outcome	Probability	Probability Active	Probability Inactive	Predicted Reliability
COC1=CC=C(C=C1)C1NC(=S)NC2=C1S(=O)(=O)CCC2	Inactive	0.8	0.2	0.8	reliable

13	Leishmania braziliensis				
Molecule	Predicted Outcome	Probability	Probability Active	Probability Inactive	Predicted Reliability
COC1=CC=C(C=C1)C1NC(=S)NC2=C1S(=O)(=O)CCC2	Active	0.6	0.6	0.4	reliable

14	Drosophila melanogaster				
Molecule	Predicted Outcome	Probability	Probability Active	Probability Inactive	Predicted Reliability
COC1=CC=C(C=C1)C1NC(=S)NC2=C1S(=O)(=O)CCC2	Inactive	1.0	0.0	1.0	unreliable

15	Leishmania major				
Molecule	Predicted Outcome	Probability	Probability Active	Probability Inactive	Predicted Reliability
COC1=CC=C(C=C1)C1NC(=S)NC2=C1S(=O)(=O)CCC2	Inactive	1.0	0.0	1.0	reliable

16	Alphis gossypii				
Molecule	Predicted Outcome	Probability	Probability Active	Probability Inactive	Predicted Reliability
COC1=CC=C(C=C1)C1NC(=S)NC2=C1S(=O)(=O)CCC2	Active	1.0	1.0	0.0	reliable

17	Alzheimer - COX2				
Molecule	Predicted Outcome	Probability	Probability Active	Probability Inactive	Predicted Reliability
COC1=CC=C(C=C1)C1NC(=S)NC2=C1S(=O)(=O)CCC2	Inactive	1.0	0.0	1.0	reliable

18	Alzheimer - iNOS				
Molecule	Predicted Outcome	Probability	Probability Active	Probability Inactive	Predicted Reliability
COC1=CC=C(C=C1)C1NC(=S)NC2=C1S(=O)(=O)CCC2	Inactive	0.8	0.2	0.8	reliable

19	Alzheimer - NADPH				
Molecule	Predicted Outcome	Probability	Probability Active	Probability Inactive	Predicted Reliability
COC1=CC=C(C=C1)C1NC(=S)NC2=C1S(=O)(=O)CCC2	Inactive	1.0	0.0	1.0	reliable

20	Alzheimer - JNK-3				
Molecule	Predicted Outcome	Probability	Probability Active	Probability Inactive	Predicted Reliability
COC1=CC=C(C=C1)C1NC(=S)NC2=C1S(=O)(=O)CCC2	Inactive	1.0	0.0	1.0	reliable

21	Alzheimer - PDE5				
Molecule	Predicted Outcome	Probability	Probability Active	Probability Inactive	Predicted Reliability
COC1=CC=C(C=C1)C1NC(=S)NC2=C1S(=O)(=O)CCC2	Inactive	0.8	0.2	0.8	reliable

22	Epimastigote Chagas				
Molecule	Predicted Outcome	Probability	Probability Active	Probability Inactive	Predicted Reliability
COC1=CC=C(C=C1)C1NC(=S)NC2=C1S(=O)(=O)CCC2	Inactive	1.0	0.0	1.0	reliable

23	Amastigote Ldonovani				
Molecule	Predicted Outcome	Probability	Probability Active	Probability Inactive	Predicted Reliability
COC1=CC=C(C=C1)C1NC(=S)NC2=C1S(=O)(=O)CCC2	Inactive	1.0	0.0	1.0	reliable

24	Amastigote Chagas				
Molecule	Predicted Outcome	Probability	Probability Active	Probability Inactive	Predicted Reliability
COC1=CC=C(C=C1)C1NC(=S)NC2=C1S(=O)(=O)CCC2	Inactive	1.0	0.0	1.0	reliable

25	Promastigote Ldonovani				
Molecule	Predicted Outcome	Probability	Probability Active	Probability Inactive	Predicted Reliability
COC1=CC=C(C=C1)C1NC(=S)NC2=C1S(=O)(=O)CCC2	Inactive	0.8	0.2	0.8	reliable

26	PTR L major				
Molecule	Predicted Outcome	Probability	Probability Active	Probability Inactive	Predicted Reliability
COC1=CC=C(C=C1)C1NC(=S)NC2=C1S(=O)(=O)CCC2	Inactive	1.0	0.0	1.0	reliable

27	Lamazonensis_amastigota				
Molecule	Predicted Outcome	Probability	Probability Active	Probability Inactive	Predicted Reliability
COC1=CC=C(C=C1)C1NC(=S)NC2=C1S(=O)(=O)CCC2	Inactive	1.0	0.0	1.0	reliable

28	Tripomastigote Chagas				
Molecule	Predicted Outcome	Probability	Probability Active	Probability Inactive	Predicted Reliability
COC1=CC=C(C=C1)C1NC(=S)NC2=C1S(=O)(=O)CCC2	Inactive	1.0	0.0	1.0	reliable

29	Lamazonensis_promastigota				
Molecule	Predicted Outcome	Probability	Probability Active	Probability Inactive	Predicted Reliability
COC1=CC=C(C=C1)C1NC(=S)NC2=C1S(=O)(=O)CCC2	Inactive	1.0	0.0	1.0	reliable

30	Tcruzi_amastigota				
Molecule	Predicted Outcome	Probability	Probability Active	Probability Inactive	Predicted Reliability
COC1=CC=C(C=C1)C1NC(=S)NC2=C1S(=O)(=O)CCC2	Active	0.8	0.8	0.2	reliable

31	Tcruzi_trypomastigota				
Molecule	Predicted Outcome	Probability	Probability Active	Probability Inactive	Predicted Reliability
COC1=CC=C(C=C1)C1NC(=S)NC2=C1S(=O)(=O)CCC2	Inactive	0.8	0.2	0.8	reliable

32	Tcruzi_epimastigota				
Molecule	Predicted Outcome	Probability	Probability Active	Probability Inactive	Predicted Reliability
COC1=CC=C(C=C1)C1NC(=S)NC2=C1S(=O)(=O)CCC2	Active	0.8	0.8	0.2	reliable

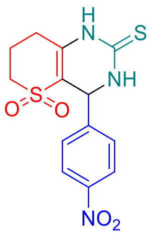

2e O=N(=O)C1=CC=C(C=C1)C1NC(=S)NC2=C1S(=O)(=O)CCC2

Molecule 1

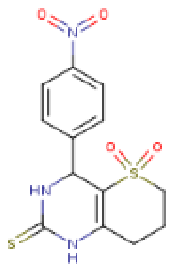

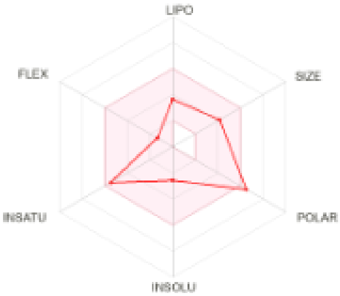

SMILES S=C1NC2=C(C(N1)c1ccc(cc1)N(=O)=O)S(=O)(=O)CCC2

Physicochemical Properties	
Formula	C13H13N3O4S2
Molecular weight	339.39 g/mol
Num. heavy atoms	22
Num. arom. heavy atoms	6
Fraction Csp3	0.31
Num. rotatable bonds	2
Num. H-bond acceptors	4
Num. H-bond donors	2
Molar Refractivity	94.56
TPSA	144.49 Å²

Lipophilicity	
Log $P_{o/w}$ (iLOGP)	1.52
Log $P_{o/w}$ (XLOGP3)	0.85
Log $P_{o/w}$ (WLOGP)	2.05
Log $P_{o/w}$ (MLOGP)	0.94
Log $P_{o/w}$ (SILICOS-IT)	0.67
Consensus Log $P_{o/w}$	1.20

Water Solubility	
Log *S* (ESOL)	-2.55
Solubility	9.58e-01 mg/ml ; 2.82e-03 mol/l
Class	Soluble
Log *S* (Ali)	-3.47
Solubility	1.16e-01 mg/ml ; 3.41e-04 mol/l
Class	Soluble
Log *S* (SILICOS-IT)	-4.02
Solubility	3.21e-02 mg/ml ; 9.46e-05 mol/l
Class	Moderately soluble

Pharmacokinetics	
GI absorption	Low
BBB permeant	No
P-gp substrate	No
CYP1A2 inhibitor	No
CYP2C19 inhibitor	No
CYP2C9 inhibitor	No
CYP2D6 inhibitor	No
CYP3A4 inhibitor	No
Log K_p (skin permeation)	-7.77 cm/s

Druglikeness	
Lipinski	Yes; 0 violation
Ghose	Yes
Veber	No; 1 violation: TPSA>140
Egan	No; 1 violation: TPSA>131.6
Muegge	Yes
Bioavailability Score	0.55

Medicinal Chemistry	
PAINS	0 alert
Brenk	2 alerts: nitro_group, thiocarbonyl_group
Leadlikeness	Yes
Synthetic accessibility	3.90

Oral toxicity prediction results for input compound

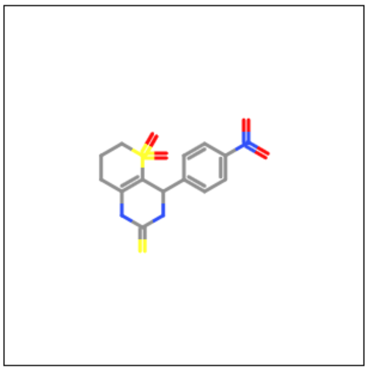

Predicted LD50: 150mg/kg

Predicted Toxicity Class: 3

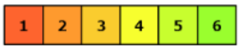

Average similarity: 35.44%

Prediction accuracy: 23%

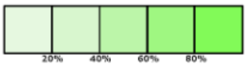

Print Toxicity Report

Name	
Molweight	339.39
Number of hydrogen bond acceptors	7
Number of hydrogen bond donors	2
Number of atoms	22
Number of bonds	24
Number of rotatable bonds	2
Molecular refractivity	94.56
Topological Polar Surface Area	144.49
octanol/water partition coefficient(logP)	3.8

1	Sars-Cov				
Molecule	Predicted Outcome	Probability	Probability Active	Probability Inactive	Predicted Reliability
O=N(=O)C1=CC=C(C=C1)C1NC(=S)NC2=C1S(=O)(=O)CCC2	Inactive	1.0	0.0	1.0	reliable

2	Dengue larvicida				
Molecule	Predicted Outcome	Probability	Probability Active	Probability Inactive	Predicted Reliability
O=N(=O)C1=CC=C(C=C1)C1NC(=S)NC2=C1S(=O)(=O)CCC2	Active	0.8	0.8	0.2	reliable

3	Acetylcholinesterase				
Molecule	Predicted Outcome	Probability	Probability Active	Probability Inactive	Predicted Reliability
O=N(=O)C1=CC=C(C=C1)C1NC(=S)NC2=C1S(=O)(=O)CCC2	Inactive	1.0	0.0	1.0	reliable

4	C_albicans				
Molecule	Predicted Outcome	Probability	Probability Active	Probability Inactive	Predicted Reliability
O=N(=O)C1=CC=C(C=C1)C1NC(=S)NC2=C1S(=O)(=O)CCC2	Active	1.0	1.0	0.0	reliable

5	Salmonella				
Molecule	Predicted Outcome	Probability	Probability Active	Probability Inactive	Predicted Reliability
O=N(=O)C1=CC=C(C=C1)C1NC(=S)NC2=C1S(=O)(=O)CCC2	Inactive	0.6	0.4	0.6	reliable

6	E_coli				
Molecule	Predicted Outcome	Probability	Probability Active	Probability Inactive	Predicted Reliability
O=N(=O)C1=CC=C(C=C1)C1NC(=S)NC2=C1S(=O)(=O)CCC2	Inactive	1.0	0.0	1.0	reliable

7	Hepatite C - Type1				
Molecule	Predicted Outcome	Probability	Probability Active	Probability Inactive	Predicted Reliability
O=N(=O)C1=CC=C(C=C1)C1NC(=S)NC2=C1S(=O)(=O)CCC2	Inactive	0.6	0.4	0.6	unreliable

8	Hepatite C - NS3-protease helicase				
Molecule	Predicted Outcome	Probability	Probability Active	Probability Inactive	Predicted Reliability
O=N(=O)C1=CC=C(C=C1)C1NC(=S)NC2=C1S(=O)(=O)CCC2	Inactive	1.0	0.0	1.0	reliable

9	Hepatite C - Serine protease				
Molecule	Predicted Outcome	Probability	Probability Active	Probability Inactive	Predicted Reliability
O=N(=O)C1=CC=C(C=C1)C1NC(=S)NC2=C1S(=O)(=O)CCC2	Inactive	0.8	0.2	0.8	unreliable

10	Hepatite C - RNA dependent				
Molecule	Predicted Outcome	Probability	Probability Active	Probability Inactive	Predicted Reliability
O=N(=O)C1=CC=C(C=C1)C1NC(=S)NC2=C1S(=O)(=O)CCC2	Inactive	1.0	0.0	1.0	reliable

11	Leishmania braziliensis				
Molecule	Predicted Outcome	Probability	Probability Active	Probability Inactive	Predicted Reliability
O=N(=O)C1=CC=C(C=C1)C1NC(=S)NC2=C1S(=O)(=O)CCC2	Active	0.6	0.6	0.4	reliable

12	Leishmania infantum - Promastigota				
Molecule	Predicted Outcome	Probability	Probability Active	Probability Inactive	Predicted Reliability
O=N(=O)C1=CC=C(C=C1)C1NC(=S)NC2=C1S(=O)(=O)CCC2	Active	0.6	0.6	0.4	reliable

13	Leishmania amazonensis - Promastigota				
Molecule	Predicted Outcome	Probability	Probability Active	Probability Inactive	Predicted Reliability
O=N(=O)C1=CC=C(C=C1)C1NC(=S)NC2=C1S(=O)(=O)CCC2	Active	0.8	0.8	0.2	reliable

14	Drosophila melanogaster				
Molecule	Predicted Outcome	Probability	Probability Active	Probability Inactive	Predicted Reliability
O=N(=O)C1=CC=C(C=C1)C1NC(=S)NC2=C1S(=O)(=O)CCC2	Inactive	1.0	0.0	1.0	unreliable

15	Leishmania major				
Molecule	Predicted Outcome	Probability	Probability Active	Probability Inactive	Predicted Reliability
O=N(=O)C1=CC=C(C=C1)C1NC(=S)NC2=C1S(=O)(=O)CCC2	Inactive	1.0	0.0	1.0	reliable

16	Alphis gossypii				
Molecule	Predicted Outcome	Probability	Probability Active	Probability Inactive	Predicted Reliability
O=N(=O)C1=CC=C(C=C1)C1NC(=S)NC2=C1S(=O)(=O)CCC2	Active	1.0	1.0	0.0	unreliable

17	Alzheimer - iNOS				
Molecule	Predicted Outcome	Probability	Probability Active	Probability Inactive	Predicted Reliability
O=N(=O)C1=CC=C(C=C1)C1NC(=S)NC2=C1S(=O)(=O)CCC2	Active	0.6	0.6	0.4	reliable

18	Alzheimer - NADPH				
Molecule	Predicted Outcome	Probability	Probability Active	Probability Inactive	Predicted Reliability
O=N(=O)C1=CC=C(C=C1)C1NC(=S)NC2=C1S(=O)(=O)CCC2	Inactive	1.0	0.0	1.0	reliable

19	Alzheimer - COX2				
Molecule	Predicted Outcome	Probability	Probability Active	Probability Inactive	Predicted Reliability
O=N(=O)C1=CC=C(C=C1)C1NC(=S)NC2=C1S(=O)(=O)CCC2	Inactive	1.0	0.0	1.0	reliable

20	Alzheimer - JNK-3				
Molecule	Predicted Outcome	Probability	Probability Active	Probability Inactive	Predicted Reliability
O=N(=O)C1=CC=C(C=C1)C1NC(=S)NC2=C1S(=O)(=O)CCC2	Inactive	1.0	0.0	1.0	reliable

21	Alzheimer - PDE5				
Molecule	Predicted Outcome	Probability	Probability Active	Probability Inactive	Predicted Reliability
O=N(=O)C1=CC=C(C=C1)C1NC(=S)NC2=C1S(=O)(=O)CCC2	Inactive	1.0	0.0	1.0	reliable

22	Amastigote Chagas				
Molecule	Predicted Outcome	Probability	Probability Active	Probability Inactive	Predicted Reliability
O=N(=O)C1=CC=C(C=C1)C1NC(=S)NC2=C1S(=O)(=O)CCC2	Inactive	1.0	0.0	1.0	reliable

23	Epimastigote Chagas				
Molecule	Predicted Outcome	Probability	Probability Active	Probability Inactive	Predicted Reliability
O=N(=O)C1=CC=C(C=C1)C1NC(=S)NC2=C1S(=O)(=O)CCC2	Inactive	1.0	0.0	1.0	reliable

24	Amastigote Ldonovani				
Molecule	Predicted Outcome	Probability	Probability Active	Probability Inactive	Predicted Reliability
O=N(=O)C1=CC=C(C=C1)C1NC(=S)NC2=C1S(=O)(=O)CCC2	Inactive	0.8	0.2	0.8	reliable

25	Promastigote Ldonovani				
Molecule	Predicted Outcome	Probability	Probability Active	Probability Inactive	Predicted Reliability
O=N(=O)C1=CC=C(C=C1)C1NC(=S)NC2=C1S(=O)(=O)CCC2	Inactive	0.6	0.4	0.6	reliable

26	PTR L major				
Molecule	Predicted Outcome	Probability	Probability Active	Probability Inactive	Predicted Reliability
O=N(=O)C1=CC=C(C=C1)C1NC(=S)NC2=C1S(=O)(=O)CCC2	Inactive	1.0	0.0	1.0	reliable

27	Lamazonensis_promastigota				
Molecule	Predicted Outcome	Probability	Probability Active	Probability Inactive	Predicted Reliability
O=N(=O)C1=CC=C(C=C1)C1NC(=S)NC2=C1S(=O)(=O)CCC2	Inactive	1.0	0.0	1.0	reliable

28	Lamazonensis_amastigota				
Molecule	Predicted Outcome	Probability	Probability Active	Probability Inactive	Predicted Reliability
O=N(=O)C1=CC=C(C=C1)C1NC(=S)NC2=C1S(=O)(=O)CCC2	Inactive	0.8	0.2	0.8	reliable

29	Tripomastigote Chagas				
**Molecule**	**Predicted Outcome**	**Probability**	**Probability Active**	**Probability Inactive**	**Predicted Reliability**
O=N(=O)C1=CC=C(C=C1)C1NC(=S)NC2=C1S(=O)(=O)CCC2	Active	0.6	0.6	0.4	reliable

30	Tcruzi_amastigota				
**Molecule**	**Predicted Outcome**	**Probability**	**Probability Active**	**Probability Inactive**	**Predicted Reliability**
O=N(=O)C1=CC=C(C=C1)C1NC(=S)NC2=C1S(=O)(=O)CCC2	Active	0.8	0.8	0.2	reliable

31	Tcruzi_epimastigota				
**Molecule**	**Predicted Outcome**	**Probability**	**Probability Active**	**Probability Inactive**	**Predicted Reliability**
O=N(=O)C1=CC=C(C=C1)C1NC(=S)NC2=C1S(=O)(=O)CCC2	Active	0.6	0.6	0.4	reliable

32	Tcruzi_trypomastigota				
**Molecule**	**Predicted Outcome**	**Probability**	**Probability Active**	**Probability Inactive**	**Predicted Reliability**
O=N(=O)C1=CC=C(C=C1)C1NC(=S)NC2=C1S(=O)(=O)CCC2	Inactive	0.8	0.2	0.8	reliable

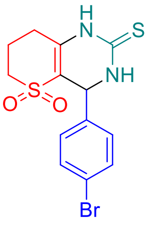

2f BrC1=CC=C(C=C1)C1NC(=S)NC2=C1S(=O)(=O)CCC2

Molecule 1

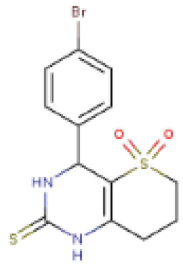

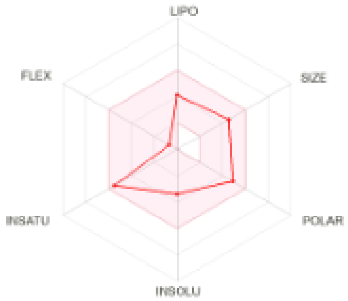

SMILES S=C1NC2=C(C(N1)c1ccc(cc1)Br)S(=O)(=O)CCC2

Physicochemical Properties	
Formula	C13H13BrN2O2S2
Molecular weight	373.29 g/mol
Num. heavy atoms	20
Num. arom. heavy atoms	6
Fraction Csp3	0.31
Num. rotatable bonds	1
Num. H-bond acceptors	2
Num. H-bond donors	2
Molar Refractivity	93.44
TPSA	98.67 Å²

Lipophilicity	
Log $P_{o/w}$ (iLOGP)	2.12
Log $P_{o/w}$ (XLOGP3)	1.72
Log $P_{o/w}$ (WLOGP)	2.38
Log $P_{o/w}$ (MLOGP)	1.83
Log $P_{o/w}$ (SILICOS-IT)	3.12
Consensus Log $P_{o/w}$	2.23

Water Solubility	
Log *S* (ESOL)	-3.39
Solubility	1.51e-01 mg/ml ; 4.04e-04 mol/l
Class	Soluble
Log *S* (Ali)	-3.41
Solubility	1.46e-01 mg/ml ; 3.91e-04 mol/l
Class	Soluble
Log *S* (SILICOS-IT)	-4.99
Solubility	3.80e-03 mg/ml ; 1.02e-05 mol/l
Class	Moderately soluble

Pharmacokinetics	
GI absorption	High
BBB permeant	No
P-gp substrate	Yes
CYP1A2 inhibitor	No
CYP2C19 inhibitor	Yes
CYP2C9 inhibitor	No
CYP2D6 inhibitor	No
CYP3A4 inhibitor	Yes
Log K_p (skin permeation)	-7.38 cm/s

Druglikeness	
Lipinski	Yes; 0 violation
Ghose	Yes
Veber	Yes
Egan	Yes
Muegge	Yes
Bioavailability Score	0.55

Medicinal Chemistry	
PAINS	0 alert
Brenk	1 alert: thiocarbonyl_group
Leadlikeness	No; 1 violation: MW>350
Synthetic accessibility	3.89

Oral toxicity prediction results for input compound

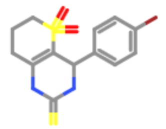

Predicted LD50: 1000mg/kg

Predicted Toxicity Class: 4

1	2	3	4	5	6

Average similarity: 32.53%

Prediction accuracy: 23%

20% 40% 60% 80%

Print Toxicity Report

Name	
Molweight	373.29
Number of hydrogen bond acceptors	5
Number of hydrogen bond donors	2
Number of atoms	20
Number of bonds	22
Number of rotable bonds	1
Molecular refractivity	93.44
Topological Polar Surface Area	98.67
octanol/water partition coefficient(logP)	4.13

1	Sars-Cov				
Molecule	Predicted Outcome	Probability	Probability Active	Probability Inactive	Predicted Reliability
BrC1=CC=C(C=C1)C1NC(=S)NC2=C1S(=O)(=O)CCC2	Inactive	0.6	0.4	0.6	reliable

2	Dengue larvicida				
Molecule	Predicted Outcome	Probability	Probability Active	Probability Inactive	Predicted Reliability
BrC1=CC=C(C=C1)C1NC(=S)NC2=C1S(=O)(=O)CCC2	Active	0.8	0.8	0.2	reliable

3	Salmonella				
Molecule	Predicted Outcome	Probability	Probability Active	Probability Inactive	Predicted Reliability
BrC1=CC=C(C=C1)C1NC(=S)NC2=C1S(=O)(=O)CCC2	Inactive	1.0	0.0	1.0	reliable

4	Acetylcholinesterase				
Molecule	Predicted Outcome	Probability	Probability Active	Probability Inactive	Predicted Reliability
BrC1=CC=C(C=C1)C1NC(=S)NC2=C1S(=O)(=O)CCC2	Inactive	1.0	0.0	1.0	reliable

5	Hepatite C - Type1				
Molecule	Predicted Outcome	Probability	Probability Active	Probability Inactive	Predicted Reliability
BrC1=CC=C(C=C1)C1NC(=S)NC2=C1S(=O)(=O)CCC2	Inactive	0.8	0.2	0.8	reliable

6	E_coli				
Molecule	Predicted Outcome	Probability	Probability Active	Probability Inactive	Predicted Reliability
BrC1=CC=C(C=C1)C1NC(=S)NC2=C1S(=O)(=O)CCC2	Inactive	1.0	0.0	1.0	reliable

7	Hepatite C - NS3-protease helicase				
**Molecule**	**Predicted Outcome**	**Probability**	**Probability Active**	**Probability Inactive**	**Predicted Reliability**
BrC1=CC=C(C=C1)C1NC(=S)NC2=C1S(=O)(=O)CCC2	Inactive	1.0	0.0	1.0	reliable

8	Hepatite C - RNA dependent				
**Molecule**	**Predicted Outcome**	**Probability**	**Probability Active**	**Probability Inactive**	**Predicted Reliability**
BrC1=CC=C(C=C1)C1NC(=S)NC2=C1S(=O)(=O)CCC2	Inactive	1.0	0.0	1.0	reliable

9	C_albicans				
**Molecule**	**Predicted Outcome**	**Probability**	**Probability Active**	**Probability Inactive**	**Predicted Reliability**
BrC1=CC=C(C=C1)C1NC(=S)NC2=C1S(=O)(=O)CCC2	Active	1.0	1.0	0.0	reliable

10	Hepatite C - Serine protease				
**Molecule**	**Predicted Outcome**	**Probability**	**Probability Active**	**Probability Inactive**	**Predicted Reliability**
BrC1=CC=C(C=C1)C1NC(=S)NC2=C1S(=O)(=O)CCC2	Inactive	0.8	0.2	0.8	unreliable

11	Leishmania amazonensis - Promastigota				
**Molecule**	**Predicted Outcome**	**Probability**	**Probability Active**	**Probability Inactive**	**Predicted Reliability**
BrC1=CC=C(C=C1)C1NC(=S)NC2=C1S(=O)(=O)CCC2	Inactive	1.0	0.0	1.0	reliable

12	Leishmania infantum - Promastigota				
**Molecule**	**Predicted Outcome**	**Probability**	**Probability Active**	**Probability Inactive**	**Predicted Reliability**
BrC1=CC=C(C=C1)C1NC(=S)NC2=C1S(=O)(=O)CCC2	Inactive	0.8	0.2	0.8	reliable

13	Leishmania braziliensis				
**Molecule**	**Predicted Outcome**	**Probability**	**Probability Active**	**Probability Inactive**	**Predicted Reliability**
BrC1=CC=C(C=C1)C1NC(=S)NC2=C1S(=O)(=O)CCC2	Active	0.6	0.6	0.4	reliable

14	Drosophila melanogaster				
**Molecule**	**Predicted Outcome**	**Probability**	**Probability Active**	**Probability Inactive**	**Predicted Reliability**
BrC1=CC=C(C=C1)C1NC(=S)NC2=C1S(=O)(=O)CCC2	Inactive	1.0	0.0	1.0	unreliable

15	Leishmania major				
**Molecule**	**Predicted Outcome**	**Probability**	**Probability Active**	**Probability Inactive**	**Predicted Reliability**
BrC1=CC=C(C=C1)C1NC(=S)NC2=C1S(=O)(=O)CCC2	Inactive	1.0	0.0	1.0	reliable

16	Alphis gossypii				
**Molecule**	**Predicted Outcome**	**Probability**	**Probability Active**	**Probability Inactive**	**Predicted Reliability**
BrC1=CC=C(C=C1)C1NC(=S)NC2=C1S(=O)(=O)CCC2	Active	1.0	1.0	0.0	reliable

17	Alzheimer - iNOS				
**Molecule**	**Predicted Outcome**	**Probability**	**Probability Active**	**Probability Inactive**	**Predicted Reliability**
BrC1=CC=C(C=C1)C1NC(=S)NC2=C1S(=O)(=O)CCC2	Inactive	0.8	0.2	0.8	reliable

18	Alzheimer - COX2				
**Molecule**	**Predicted Outcome**	**Probability**	**Probability Active**	**Probability Inactive**	**Predicted Reliability**
BrC1=CC=C(C=C1)C1NC(=S)NC2=C1S(=O)(=O)CCC2	Inactive	1.0	0.0	1.0	reliable

19	Alzheimer - NADPH				
Molecule	Predicted Outcome	Probability	Probability Active	Probability Inactive	Predicted Reliability
BrC1=CC=C(C=C1)C1NC(=S)NC2=C1S(=O)(=O)CCC2	Inactive	1.0	0.0	1.0	reliable

20	Alzheimer - JNK-3				
Molecule	Predicted Outcome	Probability	Probability Active	Probability Inactive	Predicted Reliability
BrC1=CC=C(C=C1)C1NC(=S)NC2=C1S(=O)(=O)CCC2	Inactive	1.0	0.0	1.0	reliable

21	Alzheimer - PDE5				
Molecule	Predicted Outcome	Probability	Probability Active	Probability Inactive	Predicted Reliability
BrC1=CC=C(C=C1)C1NC(=S)NC2=C1S(=O)(=O)CCC2	Inactive	1.0	0.0	1.0	reliable

22	Amastigote Ldonovani				
Molecule	Predicted Outcome	Probability	Probability Active	Probability Inactive	Predicted Reliability
BrC1=CC=C(C=C1)C1NC(=S)NC2=C1S(=O)(=O)CCC2	Inactive	0.8	0.2	0.8	reliable

23	Amastigote Chagas				
Molecule	Predicted Outcome	Probability	Probability Active	Probability Inactive	Predicted Reliability
BrC1=CC=C(C=C1)C1NC(=S)NC2=C1S(=O)(=O)CCC2	Inactive	1.0	0.0	1.0	reliable

24	Promastigote Ldonovani				
Molecule	Predicted Outcome	Probability	Probability Active	Probability Inactive	Predicted Reliability
BrC1=CC=C(C=C1)C1NC(=S)NC2=C1S(=O)(=O)CCC2	Inactive	0.6	0.4	0.6	reliable

25	Epimastigote Chagas				
Molecule	Predicted Outcome	Probability	Probability Active	Probability Inactive	Predicted Reliability
BrC1=CC=C(C=C1)C1NC(=S)NC2=C1S(=O)(=O)CCC2	Inactive	1.0	0.0	1.0	reliable

26	PTR L major				
Molecule	Predicted Outcome	Probability	Probability Active	Probability Inactive	Predicted Reliability
BrC1=CC=C(C=C1)C1NC(=S)NC2=C1S(=O)(=O)CCC2	Inactive	1.0	0.0	1.0	reliable

27	Lamazonensis_amastigota				
Molecule	Predicted Outcome	Probability	Probability Active	Probability Inactive	Predicted Reliability
BrC1=CC=C(C=C1)C1NC(=S)NC2=C1S(=O)(=O)CCC2	Inactive	0.6	0.4	0.6	reliable

28	Tripomastigote Chagas				
Molecule	Predicted Outcome	Probability	Probability Active	Probability Inactive	Predicted Reliability
BrC1=CC=C(C=C1)C1NC(=S)NC2=C1S(=O)(=O)CCC2	Inactive	0.8	0.2	0.8	reliable

29	Lamazonensis_promastigota				
Molecule	Predicted Outcome	Probability	Probability Active	Probability Inactive	Predicted Reliability
BrC1=CC=C(C=C1)C1NC(=S)NC2=C1S(=O)(=O)CCC2	Inactive	1.0	0.0	1.0	reliable

30	Tcruzi_amastigota				
Molecule	Predicted Outcome	Probability	Probability Active	Probability Inactive	Predicted Reliability
BrC1=CC=C(C=C1)C1NC(=S)NC2=C1S(=O)(=O)CCC2	Active	0.6	0.6	0.4	reliable

31	Tcruzi_epimastigota				
Molecule	Predicted Outcome	Probability	Probability Active	Probability Inactive	Predicted Reliability
BrC1=CC=C(C=C1)C1NC(=S)NC2=C1S(=O)(=O)CCC2	Active	0.8	0.8	0.2	reliable

32	Tcruzi_trypomastigota				
Molecule	Predicted Outcome	Probability	Probability Active	Probability Inactive	Predicted Reliability
BrC1=CC=C(C=C1)C1NC(=S)NC2=C1S(=O)(=O)CCC2	Inactive	0.8	0.2	0.8	reliable

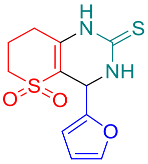

2g O=S1(=O)CCCC2=C1C(NC(=S)N2)C1=CC=CO1

Molecule 1

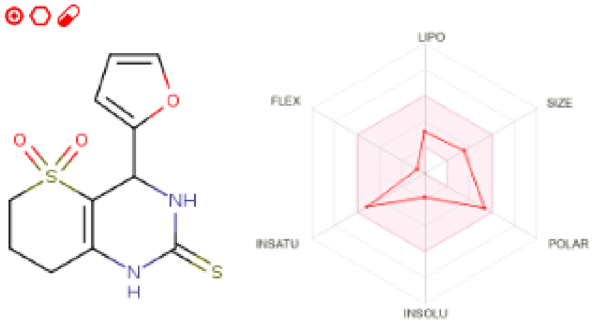

SMILES S=C1NC2=C(C(N1)c1ccco1)S(=O)(=O)CCC2

Physicochemical Properties	
Formula	C11H12N2O3S2
Molecular weight	284.35 g/mol
Num. heavy atoms	18
Num. arom. heavy atoms	5
Fraction Csp3	0.36
Num. rotatable bonds	1
Num. H-bond acceptors	3
Num. H-bond donors	2
Molar Refractivity	78.00
TPSA	111.81 Å²
**Lipophilicity**	
Log $P_{o/w}$ (iLOGP)	1.54
Log $P_{o/w}$ (XLOGP3)	0.13
Log $P_{o/w}$ (WLOGP)	1.21
Log $P_{o/w}$ (MLOGP)	-0.18
Log $P_{o/w}$ (SILICOS-IT)	1.89
Consensus Log $P_{o/w}$	0.92

Water Solubility	
Log *S* (ESOL)	-1.82
Solubility	4.26e+00 mg/ml ; 1.50e-02 mol/l
Class	Very soluble
Log *S* (Ali)	-2.03
Solubility	2.63e+00 mg/ml ; 9.24e-03 mol/l
Class	Soluble
Log *S* (SILICOS-IT)	-3.41
Solubility	1.12e-01 mg/ml ; 3.93e-04 mol/l
Class	Soluble
**Pharmacokinetics**	
GI absorption	High
BBB permeant	No
P-gp substrate	No
CYP1A2 inhibitor	Yes
CYP2C19 inhibitor	Yes
CYP2C9 inhibitor	No
CYP2D6 inhibitor	No
CYP3A4 inhibitor	No
Log K_p (skin permeation)	-7.94 cm/s
**Druglikeness**	
Lipinski	Yes; 0 violation
Ghose	Yes
Veber	Yes
Egan	Yes
Muegge	Yes
Bioavailability Score	0.55
**Medicinal Chemistry**	
PAINS	0 alert
Brenk	1 alert: thiocarbonyl_group
Leadlikeness	Yes
Synthetic accessibility	4.00

Oral toxicity prediction results for input compound

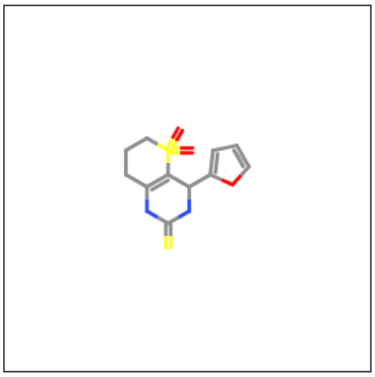

Predicted LD50: 950mg/kg

Predicted Toxicity Class: 4

1 2 3 4 5 6

Average similarity: 31%

Prediction accuracy: 23%

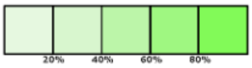

Print Toxicity Report

Name	O=S1(=O)CCCC2=C1C(
Molweight	284.36
Number of hydrogen bond acceptors	5
Number of hydrogen bond donors	2
Number of atoms	18
Number of bonds	20
Number of rotatable bonds	1
Molecular refractivity	78
Topological Polar Surface Area	111.81
octanol/water partition coefficient(logP)	2.96

1	Dengue larvicida				
Molecule	Predicted Outcome	Probability	Probability Active	Probability Inactive	Predicted Reliability
O=S1(=O)CCCC2=C1C(NC(=S)N2)C1=CC=CO1	Active	0.8	0.8	0.2	reliable

2	Salmonella				
Molecule	Predicted Outcome	Probability	Probability Active	Probability Inactive	Predicted Reliability
O=S1(=O)CCCC2=C1C(NC(=S)N2)C1=CC=CO1	Inactive	0.8	0.2	0.8	reliable

3	Sars-Cov				
Molecule	Predicted Outcome	Probability	Probability Active	Probability Inactive	Predicted Reliability
O=S1(=O)CCCC2=C1C(NC(=S)N2)C1=CC=CO1	Inactive	0.6	0.4	0.6	reliable

4	Acetylcholinesterase				
Molecule	Predicted Outcome	Probability	Probability Active	Probability Inactive	Predicted Reliability
O=S1(=O)CCCC2=C1C(NC(=S)N2)C1=CC=CO1	Inactive	1.0	0.0	1.0	reliable

5	E_coli				
Molecule	Predicted Outcome	Probability	Probability Active	Probability Inactive	Predicted Reliability
O=S1(=O)CCCC2=C1C(NC(=S)N2)C1=CC=CO1	Inactive	1.0	0.0	1.0	reliable

6	Hepatite C - Type1				
Molecule	Predicted Outcome	Probability	Probability Active	Probability Inactive	Predicted Reliability
O=S1(=O)CCCC2=C1C(NC(=S)N2)C1=CC=CO1	Inactive	0.8	0.2	0.8	reliable

7	Hepatite C - NS3-protease helicase				
Molecule	Predicted Outcome	Probability	Probability Active	Probability Inactive	Predicted Reliability
O=S1(=O)CCCC2=C1C(NC(=S)N2)C1=CC=CO1	Inactive	1.0	0.0	1.0	reliable

8	C_albicans				
Molecule	Predicted Outcome	Probability	Probability Active	Probability Inactive	Predicted Reliability
O=S1(=O)CCCC2=C1C(NC(=S)N2)C1=CC=CO1	Active	1.0	1.0	0.0	reliable

9	Hepatite C - RNA dependent				
Molecule	Predicted Outcome	Probability	Probability Active	Probability Inactive	Predicted Reliability
O=S1(=O)CCCC2=C1C(NC(=S)N2)C1=CC=CO1	Inactive	1.0	0.0	1.0	reliable

10	Hepatite C - Serine protease				
Molecule	Predicted Outcome	Probability	Probability Active	Probability Inactive	Predicted Reliability
O=S1(=O)CCCC2=C1C(NC(=S)N2)C1=CC=CO1	Inactive	1.0	0.0	1.0	unreliable

11	Leishmania amazonensis - Promastigota				
Molecule	Predicted Outcome	Probability	Probability Active	Probability Inactive	Predicted Reliability
O=S1(=O)CCCC2=C1C(NC(=S)N2)C1=CC=CO1	Inactive	0.8	0.2	0.8	reliable

12	Drosophila melanogaster				
Molecule	Predicted Outcome	Probability	Probability Active	Probability Inactive	Predicted Reliability
O=S1(=O)CCCC2=C1C(NC(=S)N2)C1=CC=CO1	Inactive	1.0	0.0	1.0	unreliable

13	Leishmania infantum - Promastigota				
Molecule	Predicted Outcome	Probability	Probability Active	Probability Inactive	Predicted Reliability
O=S1(=O)CCCC2=C1C(NC(=S)N2)C1=CC=CO1	Inactive	0.8	0.2	0.8	reliable

14	Leishmania braziliensis				
Molecule	Predicted Outcome	Probability	Probability Active	Probability Inactive	Predicted Reliability
O=S1(=O)CCCC2=C1C(NC(=S)N2)C1=CC=CO1	Active	0.6	0.6	0.4	reliable

15	Leishmania major				
Molecule	Predicted Outcome	Probability	Probability Active	Probability Inactive	Predicted Reliability
O=S1(=O)CCCC2=C1C(NC(=S)N2)C1=CC=CO1	Inactive	1.0	0.0	1.0	reliable

16	Alphis gossypii				
Molecule	Predicted Outcome	Probability	Probability Active	Probability Inactive	Predicted Reliability
O=S1(=O)CCCC2=C1C(NC(=S)N2)C1=CC=CO1	Active	1.0	1.0	0.0	reliable

17	Alzheimer - iNOS				
Molecule	Predicted Outcome	Probability	Probability Active	Probability Inactive	Predicted Reliability
O=S1(=O)CCCC2=C1C(NC(=S)N2)C1=CC=CO1	Active	0.8	0.8	0.2	reliable

18	Alzheimer - NADPH				
Molecule	Predicted Outcome	Probability	Probability Active	Probability Inactive	Predicted Reliability
O=S1(=O)CCCC2=C1C(NC(=S)N2)C1=CC=CO1	Inactive	1.0	0.0	1.0	reliable

19	Alzheimer - COX2				
Molecule	Predicted Outcome	Probability	Probability Active	Probability Inactive	Predicted Reliability
O=S1(=O)CCCC2=C1C(NC(=S)N2)C1=CC=CO1	Inactive	1.0	0.0	1.0	reliable

20	Alzheimer - JNK-3				
Molecule	Predicted Outcome	Probability	Probability Active	Probability Inactive	Predicted Reliability
O=S1(=O)CCCC2=C1C(NC(=S)N2)C1=CC=CO1	Inactive	1.0	0.0	1.0	reliable

21	Alzheimer - PDE5				
Molecule	Predicted Outcome	Probability	Probability Active	Probability Inactive	Predicted Reliability
O=S1(=O)CCCC2=C1C(NC(=S)N2)C1=CC=CO1	Inactive	1.0	0.0	1.0	reliable

22	Amastigote Chagas				
Molecule	Predicted Outcome	Probability	Probability Active	Probability Inactive	Predicted Reliability
O=S1(=O)CCCC2=C1C(NC(=S)N2)C1=CC=CO1	Inactive	1.0	0.0	1.0	reliable

23	Promastigote Ldonovani				
Molecule	Predicted Outcome	Probability	Probability Active	Probability Inactive	Predicted Reliability
O=S1(=O)CCCC2=C1C(NC(=S)N2)C1=CC=CO1	Active	0.6	0.6	0.4	reliable

24	Amastigote Chagas				
Molecule	Predicted Outcome	Probability	Probability Active	Probability Inactive	Predicted Reliability
O=S1(=O)CCCC2=C1C(NC(=S)N2)C1=CC=CO1	Inactive	1.0	0.0	1.0	reliable

25	Amastigote Ldonovani				
Molecule	Predicted Outcome	Probability	Probability Active	Probability Inactive	Predicted Reliability
O=S1(=O)CCCC2=C1C(NC(=S)N2)C1=CC=CO1	Inactive	1.0	0.0	1.0	reliable

26	PTR L major				
Molecule	Predicted Outcome	Probability	Probability Active	Probability Inactive	Predicted Reliability
O=S1(=O)CCCC2=C1C(NC(=S)N2)C1=CC=CO1	Inactive	1.0	0.0	1.0	reliable

27	Lamazonensis_amastigota				
Molecule	Predicted Outcome	Probability	Probability Active	Probability Inactive	Predicted Reliability
O=S1(=O)CCCC2=C1C(NC(=S)N2)C1=CC=CO1	Inactive	1.0	0.0	1.0	reliable

28	Tripomastigote Chagas				
Molecule	Predicted Outcome	Probability	Probability Active	Probability Inactive	Predicted Reliability
O=S1(=O)CCCC2=C1C(NC(=S)N2)C1=CC=CO1	Active	0.6	0.6	0.4	reliable

29	Lamazonensis_promastigota				
Molecule	Predicted Outcome	Probability	Probability Active	Probability Inactive	Predicted Reliability
O=S1(=O)CCCC2=C1C(NC(=S)N2)C1=CC=CO1	Inactive	0.6	0.4	0.6	reliable

30	Tcruzi_epimastigota				
Molecule	Predicted Outcome	Probability	Probability Active	Probability Inactive	Predicted Reliability
O=S1(=O)CCCC2=C1C(NC(=S)N2)C1=CC=CO1	Inactive	0.8	0.2	0.8	reliable

31	Tcruzi_trypomastigota				
Molecule	Predicted Outcome	Probability	Probability Active	Probability Inactive	Predicted Reliability
O=S1(=O)CCCC2=C1C(NC(=S)N2)C1=CC=CO1	Inactive	0.8	0.2	0.8	reliable

32	Tcruzi_amastigota				
Molecule	Predicted Outcome	Probability	Probability Active	Probability Inactive	Predicted Reliability
O=S1(=O)CCCC2=C1C(NC(=S)N2)C1=CC=CO1	Active	1.0	1.0	0.0	reliable

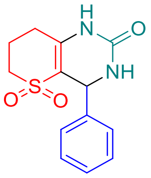

2h O=C1NC(C2=CC=CC=C2)C2=C(CCCS2(=O)=O)N1

Molecule 1

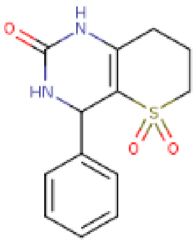

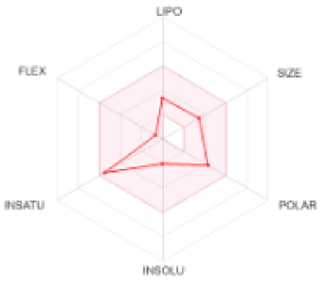

SMILES O=C1NC2=C(C(N1)c1ccccc1)S(=O)(=O)CCC2

Physicochemical Properties	
Formula	C13H14N2O3S
Molecular weight	278.33 g/mol
Num. heavy atoms	19
Num. arom. heavy atoms	6
Fraction Csp3	0.31
Num. rotatable bonds	1
Num. H-bond acceptors	3
Num. H-bond donors	2
Molar Refractivity	78.53
TPSA	83.65 Å²

Lipophilicity	
Log $P_{o/w}$ (iLOGP)	1.36
Log $P_{o/w}$ (XLOGP3)	0.42
Log $P_{o/w}$ (WLOGP)	1.46
Log $P_{o/w}$ (MLOGP)	1.15
Log $P_{o/w}$ (SILICOS-IT)	1.00
Consensus Log $P_{o/w}$	1.08

Water Solubility	
Log *S* (ESOL)	-2.00
Solubility	2.80e+00 mg/ml ; 1.00e-02 mol/l
Class	Very soluble
Log *S* (Ali)	-1.74
Solubility	5.02e+00 mg/ml ; 1.80e-02 mol/l
Class	Very soluble
Log *S* (SILICOS-IT)	-3.99
Solubility	2.84e-02 mg/ml ; 1.02e-04 mol/l
Class	Soluble

Pharmacokinetics	
GI absorption	High
BBB permeant	No
P-gp substrate	Yes
CYP1A2 inhibitor	No
CYP2C19 inhibitor	No
CYP2C9 inhibitor	No
CYP2D6 inhibitor	No
CYP3A4 inhibitor	No
Log K_p (skin permeation)	-7.70 cm/s

Druglikeness	
Lipinski	Yes; 0 violation
Ghose	Yes
Veber	Yes
Egan	Yes
Muegge	Yes
Bioavailability Score	0.55

Medicinal Chemistry	
PAINS	0 alert
Brenk	0 alert
Leadlikeness	Yes
Synthetic accessibility	3.87

Oral toxicity prediction results for input compound

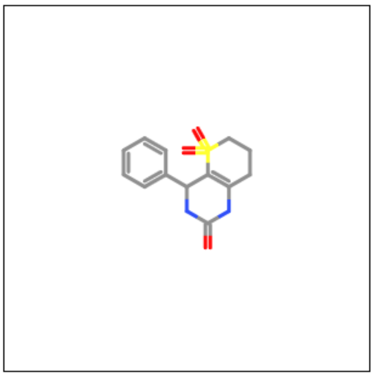

Predicted LD50: 1000mg/kg

Predicted Toxicity Class: 4

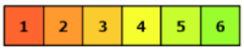

Average similarity: 35.85%

Prediction accuracy: 23%

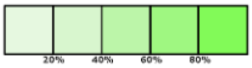

Print Toxicity Report

Name	
Molweight	278.33
Number of hydrogen bond acceptors	5
Number of hydrogen bond donors	2
Number of atoms	19
Number of bonds	21
Number of rotable bonds	1
Molecular refractivity	78.53
Topological Polar Surface Area	83.65
octanol/water partition coefficient(logP)	3.2

1	Sars-Cov				
Molecule	Predicted Outcome	Probability	Probability Active	Probability Inactive	Predicted Reliability
O=C1NC(C2=CC=CC=C2)C2=C(CCCS2(=O)=O)N1	Inactive	0.8	0.2	0.8	reliable

2	Dengue larvicida				
Molecule	Predicted Outcome	Probability	Probability Active	Probability Inactive	Predicted Reliability
O=C1NC(C2=CC=CC=C2)C2=C(CCCS2(=O)=O)N1	Active	1.0	1.0	0.0	reliable

3	Acetylcholinesterase				
Molecule	Predicted Outcome	Probability	Probability Active	Probability Inactive	Predicted Reliability
O=C1NC(C2=CC=CC=C2)C2=C(CCCS2(=O)=O)N1	Inactive	1.0	0.0	1.0	reliable

4	C_albicans				
Molecule	Predicted Outcome	Probability	Probability Active	Probability Inactive	Predicted Reliability
O=C1NC(C2=CC=CC=C2)C2=C(CCCS2(=O)=O)N1	Active	1.0	1.0	0.0	reliable

5	Salmonella				
Molecule	Predicted Outcome	Probability	Probability Active	Probability Inactive	Predicted Reliability
O=C1NC(C2=CC=CC=C2)C2=C(CCCS2(=O)=O)N1	Inactive	1.0	0.0	1.0	reliable

6	E_coli				
Molecule	Predicted Outcome	Probability	Probability Active	Probability Inactive	Predicted Reliability
O=C1NC(C2=CC=CC=C2)C2=C(CCCS2(=O)=O)N1	Inactive	0.8	0.2	0.8	reliable

7	Hepatite C - NS3-protease helicase				
**Molecule**	**Predicted Outcome**	**Probability**	**Probability Active**	**Probability Inactive**	**Predicted Reliability**
O=C1NC(C2=CC=CC=C2)C2=C(CCCS2(=O)=O)N1	Inactive	1.0	0.0	1.0	reliable

8	Hepatite C - Type1				
**Molecule**	**Predicted Outcome**	**Probability**	**Probability Active**	**Probability Inactive**	**Predicted Reliability**
O=C1NC(C2=CC=CC=C2)C2=C(CCCS2(=O)=O)N1	Inactive	0.8	0.2	0.8	reliable

9	Hepatite C - Serine protease				
**Molecule**	**Predicted Outcome**	**Probability**	**Probability Active**	**Probability Inactive**	**Predicted Reliability**
O=C1NC(C2=CC=CC=C2)C2=C(CCCS2(=O)=O)N1	Inactive	0.8	0.2	0.8	unreliable

10	Hepatite C - RNA dependent				
**Molecule**	**Predicted Outcome**	**Probability**	**Probability Active**	**Probability Inactive**	**Predicted Reliability**
O=C1NC(C2=CC=CC=C2)C2=C(CCCS2(=O)=O)N1	Inactive	1.0	0.0	1.0	reliable

11	Leishmania amazonensis - Promastigota				
**Molecule**	**Predicted Outcome**	**Probability**	**Probability Active**	**Probability Inactive**	**Predicted Reliability**
O=C1NC(C2=CC=CC=C2)C2=C(CCCS2(=O)=O)N1	Inactive	0.8	0.2	0.8	reliable

12	Leishmania infantum - Promastigota				
**Molecule**	**Predicted Outcome**	**Probability**	**Probability Active**	**Probability Inactive**	**Predicted Reliability**
O=C1NC(C2=CC=CC=C2)C2=C(CCCS2(=O)=O)N1	Inactive	0.8	0.2	0.8	reliable

13	Drosophila melanogaster				
**Molecule**	**Predicted Outcome**	**Probability**	**Probability Active**	**Probability Inactive**	**Predicted Reliability**
O=C1NC(C2=CC=CC=C2)C2=C(CCCS2(=O)=O)N1	Inactive	1.0	0.0	1.0	reliable

14	Leishmania braziliensis				
**Molecule**	**Predicted Outcome**	**Probability**	**Probability Active**	**Probability Inactive**	**Predicted Reliability**
O=C1NC(C2=CC=CC=C2)C2=C(CCCS2(=O)=O)N1	Inactive	0.8	0.2	0.8	reliable

15	Leishmania major				
**Molecule**	**Predicted Outcome**	**Probability**	**Probability Active**	**Probability Inactive**	**Predicted Reliability**
O=C1NC(C2=CC=CC=C2)C2=C(CCCS2(=O)=O)N1	Inactive	1.0	0.0	1.0	reliable

16	Alphis gossypii				
**Molecule**	**Predicted Outcome**	**Probability**	**Probability Active**	**Probability Inactive**	**Predicted Reliability**
O=C1NC(C2=CC=CC=C2)C2=C(CCCS2(=O)=O)N1	Active	1.0	1.0	0.0	reliable

17	Alzheimer - COX2				
**Molecule**	**Predicted Outcome**	**Probability**	**Probability Active**	**Probability Inactive**	**Predicted Reliability**
O=C1NC(C2=CC=CC=C2)C2=C(CCCS2(=O)=O)N1	Inactive	0.8	0.2	0.8	reliable

18	Alzheimer - iNOS				
**Molecule**	**Predicted Outcome**	**Probability**	**Probability Active**	**Probability Inactive**	**Predicted Reliability**
O=C1NC(C2=CC=CC=C2)C2=C(CCCS2(=O)=O)N1	Inactive	0.6	0.4	0.6	reliable

19	Alzheimer - NADPH				
**Molecule**	**Predicted Outcome**	**Probability**	**Probability Active**	**Probability Inactive**	**Predicted Reliability**
O=C1NC(C2=CC=CC=C2)C2=C(CCCS2(=O)=O)N1	Inactive	0.8	0.2	0.8	reliable

20	Alzheimer - JNK-3				
**Molecule**	**Predicted Outcome**	**Probability**	**Probability Active**	**Probability Inactive**	**Predicted Reliability**
O=C1NC(C2=CC=CC=C2)C2=C(CCCS2(=O)=O)N1	Inactive	1.0	0.0	1.0	reliable

21	Alzheimer - PDE5				
**Molecule**	**Predicted Outcome**	**Probability**	**Probability Active**	**Probability Inactive**	**Predicted Reliability**
O=C1NC(C2=CC=CC=C2)C2=C(CCCS2(=O)=O)N1	Inactive	1.0	0.0	1.0	reliable

22	Epimastigote Chagas				
**Molecule**	**Predicted Outcome**	**Probability**	**Probability Active**	**Probability Inactive**	**Predicted Reliability**
O=C1NC(C2=CC=CC=C2)C2=C(CCCS2(=O)=O)N1	Inactive	1.0	0.0	1.0	reliable

23	Amastigote Chagas				
**Molecule**	**Predicted Outcome**	**Probability**	**Probability Active**	**Probability Inactive**	**Predicted Reliability**
O=C1NC(C2=CC=CC=C2)C2=C(CCCS2(=O)=O)N1	Inactive	1.0	0.0	1.0	reliable

24	Amastigote Ldonovani				
**Molecule**	**Predicted Outcome**	**Probability**	**Probability Active**	**Probability Inactive**	**Predicted Reliability**
O=C1NC(C2=CC=CC=C2)C2=C(CCCS2(=O)=O)N1	Inactive	1.0	0.0	1.0	reliable

25	Promastigote Ldonovani				
**Molecule**	**Predicted Outcome**	**Probability**	**Probability Active**	**Probability Inactive**	**Predicted Reliability**
O=C1NC(C2=CC=CC=C2)C2=C(CCCS2(=O)=O)N1	Inactive	0.8	0.2	0.8	reliable

26	Lamazonensis_amastigota				
**Molecule**	**Predicted Outcome**	**Probability**	**Probability Active**	**Probability Inactive**	**Predicted Reliability**
O=C1NC(C2=CC=CC=C2)C2=C(CCCS2(=O)=O)N1	Inactive	1.0	0.0	1.0	reliable

27	Tripomastigote Chagas				
**Molecule**	**Predicted Outcome**	**Probability**	**Probability Active**	**Probability Inactive**	**Predicted Reliability**
O=C1NC(C2=CC=CC=C2)C2=C(CCCS2(=O)=O)N1	Active	1.0	1.0	0.0	reliable

28	PTR L major				
**Molecule**	**Predicted Outcome**	**Probability**	**Probability Active**	**Probability Inactive**	**Predicted Reliability**
O=C1NC(C2=CC=CC=C2)C2=C(CCCS2(=O)=O)N1	Inactive	0.8	0.2	0.8	reliable

29	Lamazonensis_promastigota				
**Molecule**	**Predicted Outcome**	**Probability**	**Probability Active**	**Probability Inactive**	**Predicted Reliability**
O=C1NC(C2=CC=CC=C2)C2=C(CCCS2(=O)=O)N1	Inactive	1.0	0.0	1.0	reliable

30	Tcruzi_amastigota				
**Molecule**	**Predicted Outcome**	**Probability**	**Probability Active**	**Probability Inactive**	**Predicted Reliability**
O=C1NC(C2=CC=CC=C2)C2=C(CCCS2(=O)=O)N1	Inactive	0.6	0.4	0.6	reliable

31	Tcruzi_epimastigota				
**Molecule**	**Predicted Outcome**	**Probability**	**Probability Active**	**Probability Inactive**	**Predicted Reliability**
O=C1NC(C2=CC=CC=C2)C2=C(CCCS2(=O)=O)N1	Active	1.0	1.0	0.0	reliable

32	Tcruzi_trypomastigota				
**Molecule**	**Predicted Outcome**	**Probability**	**Probability Active**	**Probability Inactive**	**Predicted Reliability**
O=C1NC(C2=CC=CC=C2)C2=C(CCCS2(=O)=O)N1	Inactive	1.0	0.0	1.0	reliable

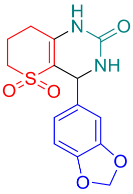

2i O=C1NC(C2=CC=C3OCOC3=C2)C2=C(CCCS2(=O)=O)N1

Molecule 1

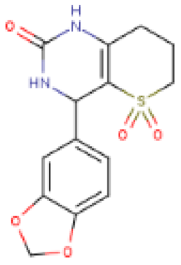

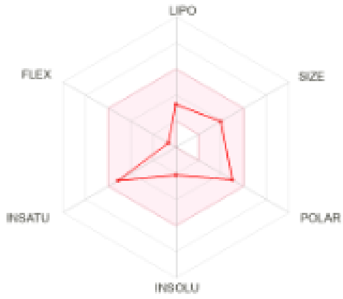

SMILES O=C1NC2=C(C(N1)c1ccc3c(c1)OCO3)S(=O)(=O)CCC2

Physicochemical Properties	
Formula	C14H14N2O5S
Molecular weight	322.34 g/mol
Num. heavy atoms	22
Num. arom. heavy atoms	6
Fraction Csp3	0.36
Num. rotatable bonds	1
Num. H-bond acceptors	5
Num. H-bond donors	2
Molar Refractivity	84.60
TPSA	102.11 Å²

Lipophilicity	
Log $P_{o/w}$ (iLOGP)	1.67
Log $P_{o/w}$ (XLOGP3)	0.24
Log $P_{o/w}$ (WLOGP)	1.18
Log $P_{o/w}$ (MLOGP)	0.74
Log $P_{o/w}$ (SILICOS-IT)	0.86
Consensus Log $P_{o/w}$	0.94

Water Solubility	
Log *S* (ESOL)	-2.13
Solubility	2.41e+00 mg/ml ; 7.49e-03 mol/l
Class	Soluble
Log *S* (Ali)	-1.94
Solubility	3.68e+00 mg/ml ; 1.14e-02 mol/l
Class	Very soluble
Log *S* (SILICOS-IT)	-3.72
Solubility	6.09e-02 mg/ml ; 1.89e-04 mol/l
Class	Soluble

Pharmacokinetics	
GI absorption	High
BBB permeant	No
P-gp substrate	Yes
CYP1A2 inhibitor	No
CYP2C19 inhibitor	No
CYP2C9 inhibitor	No
CYP2D6 inhibitor	No
CYP3A4 inhibitor	No
Log K_p (skin permeation)	-8.10 cm/s

Druglikeness	
Lipinski	Yes; 0 violation
Ghose	Yes
Veber	Yes
Egan	Yes
Muegge	Yes
Bioavailability Score	0.55

Medicinal Chemistry	
PAINS	0 alert
Brenk	0 alert
Leadlikeness	Yes
Synthetic accessibility	3.93

Oral toxicity prediction results for input compound

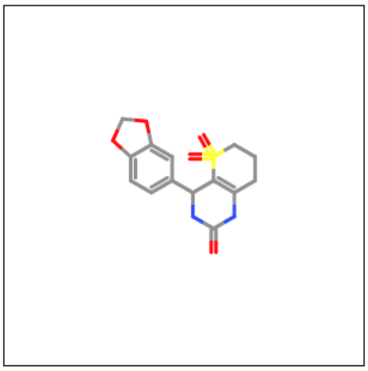

Predicted LD50: 2000mg/kg

Predicted Toxicity Class: 4

1	2	3	4	5	6

Average similarity: 36%

Prediction accuracy: 23%

20% 40% 60% 80%

Print Toxicity Report

Name	
Molweight	322.34
Number of hydrogen bond acceptors	7
Number of hydrogen bond donors	2
Number of atoms	22
Number of bonds	25
Number of rotatable bonds	1
Molecular refractivity	84.6
Topological Polar Surface Area	102.11
octanol/water partition coefficient(logP)	2.93

1	Dengue larvicida				
**Molecule**	**Predicted Outcome**	**Probability**	**Probability Active**	**Probability Inactive**	**Predicted Reliability**
O=C1NC(C2=CC=C3OCOC3=C2)C2=C(CCCS2(=O)=O)N1	Active	1.0	1.0	0.0	reliable

2	Sars-Cov				
**Molecule**	**Predicted Outcome**	**Probability**	**Probability Active**	**Probability Inactive**	**Predicted Reliability**
O=C1NC(C2=CC=C3OCOC3=C2)C2=C(CCCS2(=O)=O)N1	Active	0.8	0.8	0.2	reliable

3	Acetylcholinesterase				
**Molecule**	**Predicted Outcome**	**Probability**	**Probability Active**	**Probability Inactive**	**Predicted Reliability**
O=C1NC(C2=CC=C3OCOC3=C2)C2=C(CCCS2(=O)=O)N1	Inactive	0.8	0.2	0.8	reliable

4	Salmonella				
**Molecule**	**Predicted Outcome**	**Probability**	**Probability Active**	**Probability Inactive**	**Predicted Reliability**
O=C1NC(C2=CC=C3OCOC3=C2)C2=C(CCCS2(=O)=O)N1	Inactive	1.0	0.0	1.0	unreliable

5	C_albicans				
**Molecule**	**Predicted Outcome**	**Probability**	**Probability Active**	**Probability Inactive**	**Predicted Reliability**
O=C1NC(C2=CC=C3OCOC3=C2)C2=C(CCCS2(=O)=O)N1	Active	1.0	1.0	0.0	reliable

6	E_coli				
**Molecule**	**Predicted Outcome**	**Probability**	**Probability Active**	**Probability Inactive**	**Predicted Reliability**
O=C1NC(C2=CC=C3OCOC3=C2)C2=C(CCCS2(=O)=O)N1	Inactive	0.6	0.4	0.6	reliable

7	Hepatite C - NS3-protease helicase				
**Molecule**	**Predicted Outcome**	**Probability**	**Probability Active**	**Probability Inactive**	**Predicted Reliability**
O=C1NC(C2=CC=C3OCOC3=C2)C2=C(CCCS2(=O)=O)N1	Inactive	1.0	0.0	1.0	unreliable

8	Hepatite C - Type1				
**Molecule**	**Predicted Outcome**	**Probability**	**Probability Active**	**Probability Inactive**	**Predicted Reliability**
O=C1NC(C2=CC=C3OCOC3=C2)C2=C(CCCS2(=O)=O)N1	Inactive	0.8	0.2	0.8	unreliable

9	Hepatite C - RNA dependent				
**Molecule**	**Predicted Outcome**	**Probability**	**Probability Active**	**Probability Inactive**	**Predicted Reliability**
O=C1NC(C2=CC=C3OCOC3=C2)C2=C(CCCS2(=O)=O)N1	Inactive	1.0	0.0	1.0	reliable

10	Leishmania amazonensis - Promastigota				
**Molecule**	**Predicted Outcome**	**Probability**	**Probability Active**	**Probability Inactive**	**Predicted Reliability**
O=C1NC(C2=CC=C3OCOC3=C2)C2=C(CCCS2(=O)=O)N1	Active	0.6	0.6	0.4	reliable

11	Hepatite C - Serine protease				
**Molecule**	**Predicted Outcome**	**Probability**	**Probability Active**	**Probability Inactive**	**Predicted Reliability**
O=C1NC(C2=CC=C3OCOC3=C2)C2=C(CCCS2(=O)=O)N1	Inactive	1.0	0.0	1.0	unreliable

12	Leishmania infantum - Promastigota				
**Molecule**	**Predicted Outcome**	**Probability**	**Probability Active**	**Probability Inactive**	**Predicted Reliability**
O=C1NC(C2=CC=C3OCOC3=C2)C2=C(CCCS2(=O)=O)N1	Active	0.8	0.8	0.2	reliable

13	Drosophila melanogaster				
**Molecule**	**Predicted Outcome**	**Probability**	**Probability Active**	**Probability Inactive**	**Predicted Reliability**
O=C1NC(C2=CC=C3OCOC3=C2)C2=C(CCCS2(=O)=O)N1	Inactive	1.0	0.0	1.0	unreliable

14	Leishmania braziliensis				
**Molecule**	**Predicted Outcome**	**Probability**	**Probability Active**	**Probability Inactive**	**Predicted Reliability**
O=C1NC(C2=CC=C3OCOC3=C2)C2=C(CCCS2(=O)=O)N1	Inactive	0.8	0.2	0.8	reliable

15	Leishmania major				
**Molecule**	**Predicted Outcome**	**Probability**	**Probability Active**	**Probability Inactive**	**Predicted Reliability**
O=C1NC(C2=CC=C3OCOC3=C2)C2=C(CCCS2(=O)=O)N1	Active	1.0	1.0	0.0	reliable

16	Alphis gossypii				
**Molecule**	**Predicted Outcome**	**Probability**	**Probability Active**	**Probability Inactive**	**Predicted Reliability**
O=C1NC(C2=CC=C3OCOC3=C2)C2=C(CCCS2(=O)=O)N1	Active	1.0	1.0	0.0	unreliable

17	Alzheimer - NADPH				
**Molecule**	**Predicted Outcome**	**Probability**	**Probability Active**	**Probability Inactive**	**Predicted Reliability**
O=C1NC(C2=CC=C3OCOC3=C2)C2=C(CCCS2(=O)=O)N1	Inactive	0.6	0.4	0.6	reliable

18	Alzheimer - JNK-3				
**Molecule**	**Predicted Outcome**	**Probability**	**Probability Active**	**Probability Inactive**	**Predicted Reliability**
O=C1NC(C2=CC=C3OCOC3=C2)C2=C(CCCS2(=O)=O)N1	Inactive	1.0	0.0	1.0	reliable

19	Alzheimer - iNOS				
**Molecule**	**Predicted Outcome**	**Probability**	**Probability Active**	**Probability Inactive**	**Predicted Reliability**
O=C1NC(C2=CC=C3OCOC3=C2)C2=C(CCCS2(=O)=O)N1	Inactive	0.6	0.4	0.6	reliable

20	Alzheimer - COX2				
**Molecule**	**Predicted Outcome**	**Probability**	**Probability Active**	**Probability Inactive**	**Predicted Reliability**
O=C1NC(C2=CC=C3OCOC3=C2)C2=C(CCCS2(=O)=O)N1	Inactive	1.0	0.0	1.0	reliable

21	Alzheimer - PDE5				
**Molecule**	**Predicted Outcome**	**Probability**	**Probability Active**	**Probability Inactive**	**Predicted Reliability**
O=C1NC(C2=CC=C3OCOC3=C2)C2=C(CCCS2(=O)=O)N1	Inactive	1.0	0.0	1.0	reliable

22	Amastigote Chagas				
Molecule	Predicted Outcome	Probability	Probability Active	Probability Inactive	Predicted Reliability
O=C1NC(C2=CC=C3OCOC3=C2)C2=C(CCCS2(=O)=O)N1	Inactive	1.0	0.0	1.0	reliable

23	Amastigote Ldonovani				
Molecule	Predicted Outcome	Probability	Probability Active	Probability Inactive	Predicted Reliability
O=C1NC(C2=CC=C3OCOC3=C2)C2=C(CCCS2(=O)=O)N1	Inactive	1.0	0.0	1.0	reliable

24	Epimastigote Chagas				
Molecule	Predicted Outcome	Probability	Probability Active	Probability Inactive	Predicted Reliability
O=C1NC(C2=CC=C3OCOC3=C2)C2=C(CCCS2(=O)=O)N1	Inactive	1.0	0.0	1.0	reliable

25	PTR L major				
Molecule	Predicted Outcome	Probability	Probability Active	Probability Inactive	Predicted Reliability
O=C1NC(C2=CC=C3OCOC3=C2)C2=C(CCCS2(=O)=O)N1	Active	1.0	1.0	0.0	reliable

26	Promastigote Ldonovani				
Molecule	Predicted Outcome	Probability	Probability Active	Probability Inactive	Predicted Reliability
O=C1NC(C2=CC=C3OCOC3=C2)C2=C(CCCS2(=O)=O)N1	Inactive	0.8	0.2	0.8	reliable

27	Lamazonensis_amastigota				
Molecule	Predicted Outcome	Probability	Probability Active	Probability Inactive	Predicted Reliability
O=C1NC(C2=CC=C3OCOC3=C2)C2=C(CCCS2(=O)=O)N1	Inactive	1.0	0.0	1.0	reliable

28	Tripomastigote Chagas				
Molecule	Predicted Outcome	Probability	Probability Active	Probability Inactive	Predicted Reliability
O=C1NC(C2=CC=C3OCOC3=C2)C2=C(CCCS2(=O)=O)N1	Inactive	0.8	0.2	0.8	reliable

29	Lamazonensis_promastigota				
Molecule	Predicted Outcome	Probability	Probability Active	Probability Inactive	Predicted Reliability
O=C1NC(C2=CC=C3OCOC3=C2)C2=C(CCCS2(=O)=O)N1	Inactive	0.6	0.4	0.6	reliable

30	Tcruzi_amastigota				
Molecule	Predicted Outcome	Probability	Probability Active	Probability Inactive	Predicted Reliability
O=C1NC(C2=CC=C3OCOC3=C2)C2=C(CCCS2(=O)=O)N1	Inactive	0.8	0.2	0.8	reliable

31	Tcruzi_epimastigota				
**Molecule**	**Predicted Outcome**	**Probability**	**Probability Active**	**Probability Inactive**	**Predicted Reliability**
O=C1NC(C2=CC=C3OCOC3=C2)C2=C(CCCS2(=O)=O)N1	Inactive	0.8	0.2	0.8	reliable

32	Tcruzi_trypomastigota				
**Molecule**	**Predicted Outcome**	**Probability**	**Probability Active**	**Probability Inactive**	**Predicted Reliability**
O=C1NC(C2=CC=C3OCOC3=C2)C2=C(CCCS2(=O)=O)N1	Inactive	0.8	0.2	0.8	reliable

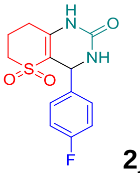

2j FC1=CC=C(C=C1)C1NC(=O)NC2=C1S(=O)(=O)CCC2

Molecule 1

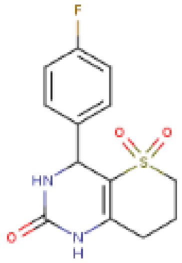

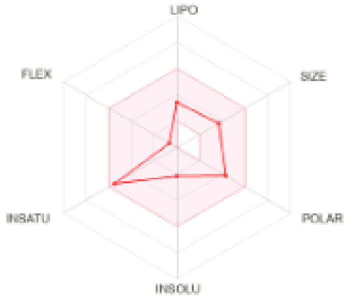

SMILES O=C1NC2=C(C(N1)c1ccc(cc1)F)S(=O)(=O)CCC2

Physicochemical Properties	
Formula	C13H13FN2O3S
Molecular weight	296.32 g/mol
Num. heavy atoms	20
Num. arom. heavy atoms	6
Fraction Csp3	0.31
Num. rotatable bonds	1
Num. H-bond acceptors	4
Num. H-bond donors	2
Molar Refractivity	78.49
TPSA	83.65 Å²

Lipophilicity	
Log $P_{o/w}$ (iLOGP)	1.56
Log $P_{o/w}$ (XLOGP3)	0.52
Log $P_{o/w}$ (WLOGP)	2.01
Log $P_{o/w}$ (MLOGP)	1.55
Log $P_{o/w}$ (SILICOS-IT)	1.41
Consensus Log $P_{o/w}$	1.41

Water Solubility	
Log *S* (ESOL)	-2.16
Solubility	2.05e+00 mg/ml ; 6.91e-03 mol/l
Class	Soluble
Log *S* (Ali)	-1.85
Solubility	4.21e+00 mg/ml ; 1.42e-02 mol/l
Class	Very soluble
Log *S* (SILICOS-IT)	-4.26
Solubility	1.61e-02 mg/ml ; 5.45e-05 mol/l
Class	Moderately soluble

Pharmacokinetics	
GI absorption	High
BBB permeant	No
P-gp substrate	Yes
CYP1A2 inhibitor	No
CYP2C19 inhibitor	No
CYP2C9 inhibitor	No
CYP2D6 inhibitor	No
CYP3A4 inhibitor	No
Log K_p (skin permeation)	-7.74 cm/s

Druglikeness	
Lipinski	Yes; 0 violation
Ghose	Yes
Veber	Yes
Egan	Yes
Muegge	Yes
Bioavailability Score	0.55

Medicinal Chemistry	
PAINS	0 alert
Brenk	0 alert
Leadlikeness	Yes
Synthetic accessibility	3.86

Oral toxicity prediction results for input compound

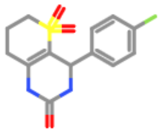

Predicted LD50: 1000mg/kg

Predicted Toxicity Class: 4

1	2	3	4	5	6

Average similarity: 34.87%

Prediction accuracy: 23%

20% 40% 60% 80%

Print Toxicity Report

Name	
Molweight	296.32
Number of hydrogen bond acceptors	5
Number of hydrogen bond donors	2
Number of atoms	20
Number of bonds	22
Number of rotable bonds	1
Molecular refractivity	78.49
Topological Polar Surface Area	83.65
octanol/water partition coefficient(logP)	3.34

1	Sars-Cov				
**Molecule**	**Predicted Outcome**	**Probability**	**Probability Active**	**Probability Inactive**	**Predicted Reliability**
FC1=CC=C(C=C1)C1NC(=O)NC2=C1S(=O)(=O)CCC2	Inactive	1.0	0.0	1.0	reliable

2	Dengue larvicida				
**Molecule**	**Predicted Outcome**	**Probability**	**Probability Active**	**Probability Inactive**	**Predicted Reliability**
FC1=CC=C(C=C1)C1NC(=O)NC2=C1S(=O)(=O)CCC2	Active	0.8	0.8	0.2	reliable

3	Acetylcholinesterase				
**Molecule**	**Predicted Outcome**	**Probability**	**Probability Active**	**Probability Inactive**	**Predicted Reliability**
FC1=CC=C(C=C1)C1NC(=O)NC2=C1S(=O)(=O)CCC2	Inactive	1.0	0.0	1.0	reliable

4	Salmonella				
**Molecule**	**Predicted Outcome**	**Probability**	**Probability Active**	**Probability Inactive**	**Predicted Reliability**
FC1=CC=C(C=C1)C1NC(=O)NC2=C1S(=O)(=O)CCC2	Active	0.6	0.6	0.4	reliable

5	C_albicans				
**Molecule**	**Predicted Outcome**	**Probability**	**Probability Active**	**Probability Inactive**	**Predicted Reliability**
FC1=CC=C(C=C1)C1NC(=O)NC2=C1S(=O)(=O)CCC2	Active	1.0	1.0	0.0	reliable

6	E_coli				
**Molecule**	**Predicted Outcome**	**Probability**	**Probability Active**	**Probability Inactive**	**Predicted Reliability**
FC1=CC=C(C=C1)C1NC(=O)NC2=C1S(=O)(=O)CCC2	Inactive	0.6	0.4	0.6	reliable

7	Hepatite C - NS3-protease helicase				
**Molecule**	**Predicted Outcome**	**Probability**	**Probability Active**	**Probability Inactive**	**Predicted Reliability**
FC1=CC=C(C=C1)C1NC(=O)NC2=C1S(=O)(=O)CCC2	Inactive	1.0	0.0	1.0	reliable

8	Hepatite C - Type1				
**Molecule**	**Predicted Outcome**	**Probability**	**Probability Active**	**Probability Inactive**	**Predicted Reliability**
FC1=CC=C(C=C1)C1NC(=O)NC2=C1S(=O)(=O)CCC2	Inactive	0.8	0.2	0.8	reliable

9	Hepatite C - RNA dependent				
**Molecule**	**Predicted Outcome**	**Probability**	**Probability Active**	**Probability Inactive**	**Predicted Reliability**
FC1=CC=C(C=C1)C1NC(=O)NC2=C1S(=O)(=O)CCC2	Inactive	1.0	0.0	1.0	reliable

10	Leishmania amazonensis - Promastigota				
**Molecule**	**Predicted Outcome**	**Probability**	**Probability Active**	**Probability Inactive**	**Predicted Reliability**
FC1=CC=C(C=C1)C1NC(=O)NC2=C1S(=O)(=O)CCC2	Inactive	0.6	0.4	0.6	reliable

11	Leishmania infantum - Promastigota				
**Molecule**	**Predicted Outcome**	**Probability**	**Probability Active**	**Probability Inactive**	**Predicted Reliability**
FC1=CC=C(C=C1)C1NC(=O)NC2=C1S(=O)(=O)CCC2	Inactive	0.8	0.2	0.8	reliable

12	Hepatite C - Serine protease				
**Molecule**	**Predicted Outcome**	**Probability**	**Probability Active**	**Probability Inactive**	**Predicted Reliability**
FC1=CC=C(C=C1)C1NC(=O)NC2=C1S(=O)(=O)CCC2	Inactive	1.0	0.0	1.0	unreliable

13	Leishmania braziliensis				
**Molecule**	**Predicted Outcome**	**Probability**	**Probability Active**	**Probability Inactive**	**Predicted Reliability**
FC1=CC=C(C=C1)C1NC(=O)NC2=C1S(=O)(=O)CCC2	Active	0.6	0.6	0.4	reliable

14	Drosophila melanogaster				
**Molecule**	**Predicted Outcome**	**Probability**	**Probability Active**	**Probability Inactive**	**Predicted Reliability**
FC1=CC=C(C=C1)C1NC(=O)NC2=C1S(=O)(=O)CCC2	Inactive	1.0	0.0	1.0	reliable

15	Leishmania major				
**Molecule**	**Predicted Outcome**	**Probability**	**Probability Active**	**Probability Inactive**	**Predicted Reliability**
FC1=CC=C(C=C1)C1NC(=O)NC2=C1S(=O)(=O)CCC2	Inactive	1.0	0.0	1.0	reliable

16	Alphis gossypii				
Molecule	Predicted Outcome	Probability	Probability Active	Probability Inactive	Predicted Reliability
FC1=CC=C(C=C1)C1NC(=O)NC2=C1S(=O)(=O)CCC2	Active	1.0	1.0	0.0	reliable

17	Alzheimer - COX2				
Molecule	Predicted Outcome	Probability	Probability Active	Probability Inactive	Predicted Reliability
FC1=CC=C(C=C1)C1NC(=O)NC2=C1S(=O)(=O)CCC2	Inactive	0.6	0.4	0.6	reliable

18	Alzheimer - iNOS				
Molecule	Predicted Outcome	Probability	Probability Active	Probability Inactive	Predicted Reliability
FC1=CC=C(C=C1)C1NC(=O)NC2=C1S(=O)(=O)CCC2	Inactive	1.0	0.0	1.0	reliable

19	Alzheimer - JNK-3				
Molecule	Predicted Outcome	Probability	Probability Active	Probability Inactive	Predicted Reliability
FC1=CC=C(C=C1)C1NC(=O)NC2=C1S(=O)(=O)CCC2	Inactive	1.0	0.0	1.0	reliable

20	Alzheimer - NADPH				
Molecule	Predicted Outcome	Probability	Probability Active	Probability Inactive	Predicted Reliability
FC1=CC=C(C=C1)C1NC(=O)NC2=C1S(=O)(=O)CCC2	Inactive	0.6	0.4	0.6	reliable

21	Alzheimer - PDE5				
Molecule	Predicted Outcome	Probability	Probability Active	Probability Inactive	Predicted Reliability
FC1=CC=C(C=C1)C1NC(=O)NC2=C1S(=O)(=O)CCC2	Inactive	0.8	0.2	0.8	reliable

22	Amastigote Chagas				
Molecule	Predicted Outcome	Probability	Probability Active	Probability Inactive	Predicted Reliability
FC1=CC=C(C=C1)C1NC(=O)NC2=C1S(=O)(=O)CCC2	Inactive	1.0	0.0	1.0	reliable

23	Amastigote Ldonovani				
Molecule	Predicted Outcome	Probability	Probability Active	Probability Inactive	Predicted Reliability
FC1=CC=C(C=C1)C1NC(=O)NC2=C1S(=O)(=O)CCC2	Inactive	1.0	0.0	1.0	reliable

24	Epimastigote Chagas				
Molecule	Predicted Outcome	Probability	Probability Active	Probability Inactive	Predicted Reliability
FC1=CC=C(C=C1)C1NC(=O)NC2=C1S(=O)(=O)CCC2	Inactive	1.0	0.0	1.0	reliable

25	Promastigote Ldonovani				
Molecule	Predicted Outcome	Probability	Probability Active	Probability Inactive	Predicted Reliability
FC1=CC=C(C=C1)C1NC(=O)NC2=C1S(=O)(=O)CCC2	Active	0.6	0.6	0.4	reliable

26	Lamazonensis_amastigota				
Molecule	Predicted Outcome	Probability	Probability Active	Probability Inactive	Predicted Reliability
FC1=CC=C(C=C1)C1NC(=O)NC2=C1S(=O)(=O)CCC2	Inactive	0.8	0.2	0.8	reliable

27	Tripomastigote Chagas				
Molecule	Predicted Outcome	Probability	Probability Active	Probability Inactive	Predicted Reliability
FC1=CC=C(C=C1)C1NC(=O)NC2=C1S(=O)(=O)CCC2	Active	1.0	1.0	0.0	reliable

28	PTR L major				
Molecule	Predicted Outcome	Probability	Probability Active	Probability Inactive	Predicted Reliability
FC1=CC=C(C=C1)C1NC(=O)NC2=C1S(=O)(=O)CCC2	Inactive	0.8	0.2	0.8	reliable

29	Lamazonensis_promastigota				
Molecule	Predicted Outcome	Probability	Probability Active	Probability Inactive	Predicted Reliability
FC1=CC=C(C=C1)C1NC(=O)NC2=C1S(=O)(=O)CCC2	Inactive	1.0	0.0	1.0	reliable

30	Tcruzi_epimastigota				
**Molecule**	**Predicted Outcome**	**Probability**	**Probability Active**	**Probability Inactive**	**Predicted Reliability**
FC1=CC=C(C=C1)C1NC(=O)NC2=C1S(=O)(=O)CCC2	Active	0.8	0.8	0.2	reliable

31	Tcruzi_trypomastigota				
**Molecule**	**Predicted Outcome**	**Probability**	**Probability Active**	**Probability Inactive**	**Predicted Reliability**
FC1=CC=C(C=C1)C1NC(=O)NC2=C1S(=O)(=O)CCC2	Inactive	0.8	0.2	0.8	reliable

32	Tcruzi_amastigota				
**Molecule**	**Predicted Outcome**	**Probability**	**Probability Active**	**Probability Inactive**	**Predicted Reliability**
FC1=CC=C(C=C1)C1NC(=O)NC2=C1S(=O)(=O)CCC2	Inactive	0.6	0.4	0.6	reliable

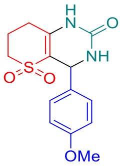

2k COC1=CC=C(C=C1)C1NC(=O)NC2=C1S(=O)(=O)CCC2

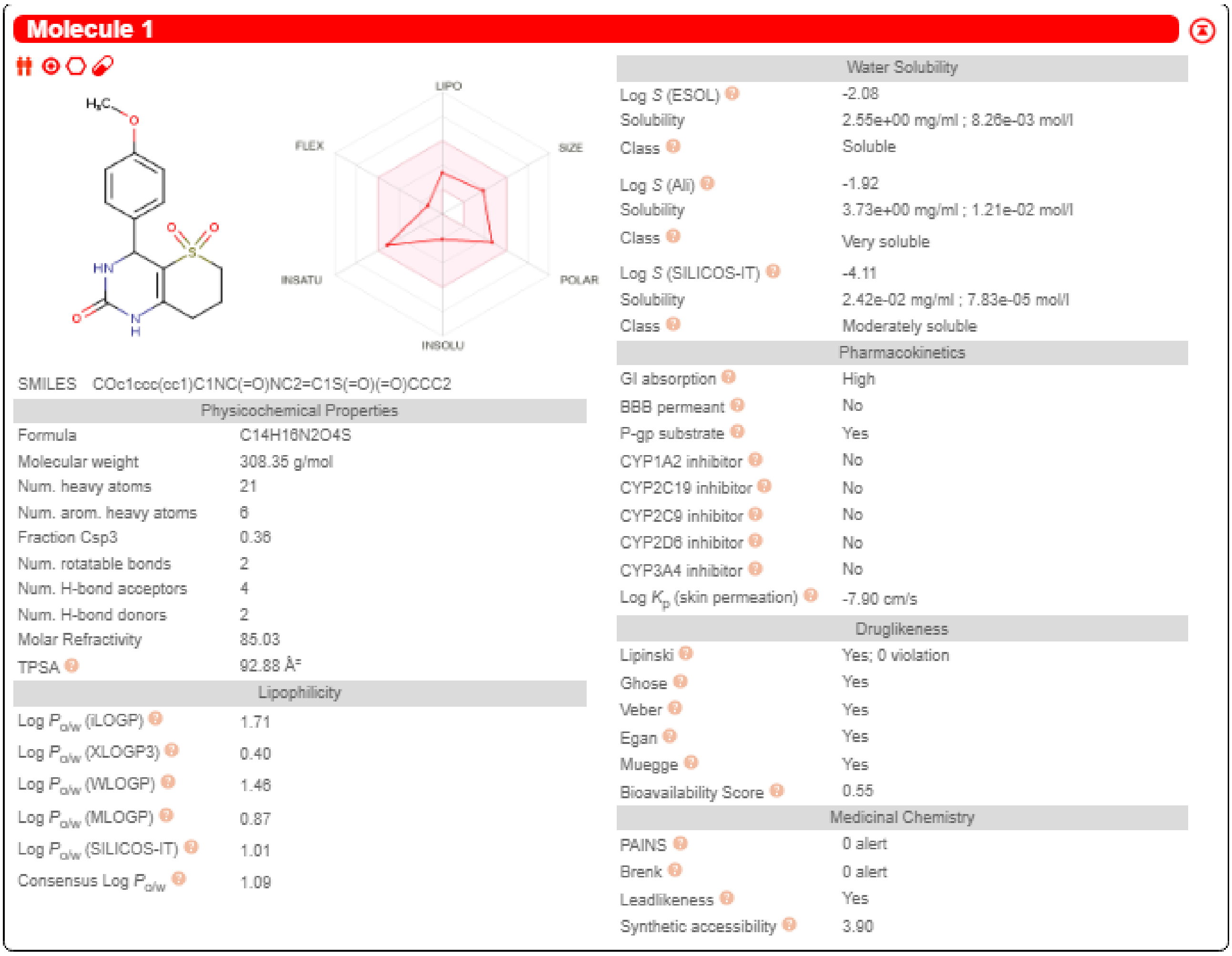

Molecule 1

SMILES COc1ccc(cc1)C1NC(=O)NC2=C1S(=O)(=O)CCC2

Physicochemical Properties	
Formula	C14H16N2O4S
Molecular weight	308.35 g/mol
Num. heavy atoms	21
Num. arom. heavy atoms	6
Fraction Csp3	0.36
Num. rotatable bonds	2
Num. H-bond acceptors	4
Num. H-bond donors	2
Molar Refractivity	85.03
TPSA	92.88 Å²

Lipophilicity	
Log $P_{o/w}$ (iLOGP)	1.71
Log $P_{o/w}$ (XLOGP3)	0.40
Log $P_{o/w}$ (WLOGP)	1.46
Log $P_{o/w}$ (MLOGP)	0.87
Log $P_{o/w}$ (SILICOS-IT)	1.01
Consensus Log $P_{o/w}$	1.09

Water Solubility	
Log *S* (ESOL)	-2.08
Solubility	2.55e+00 mg/ml ; 8.26e-03 mol/l
Class	Soluble
Log *S* (Ali)	-1.92
Solubility	3.73e+00 mg/ml ; 1.21e-02 mol/l
Class	Very soluble
Log *S* (SILICOS-IT)	-4.11
Solubility	2.42e-02 mg/ml ; 7.83e-05 mol/l
Class	Moderately soluble

Pharmacokinetics	
GI absorption	High
BBB permeant	No
P-gp substrate	Yes
CYP1A2 inhibitor	No
CYP2C19 inhibitor	No
CYP2C9 inhibitor	No
CYP2D6 inhibitor	No
CYP3A4 inhibitor	No
Log K_p (skin permeation)	-7.90 cm/s

Druglikeness	
Lipinski	Yes; 0 violation
Ghose	Yes
Veber	Yes
Egan	Yes
Muegge	Yes
Bioavailability Score	0.55

Medicinal Chemistry	
PAINS	0 alert
Brenk	0 alert
Leadlikeness	Yes
Synthetic accessibility	3.90

Oral toxicity prediction results for input compound

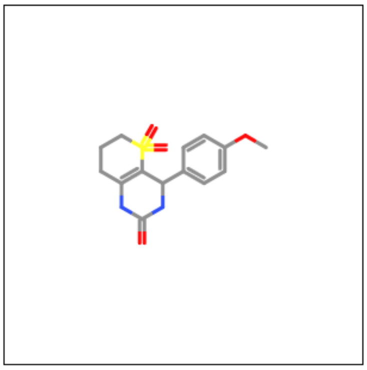

Predicted LD50: 1000mg/kg

Predicted Toxicity Class: 4

1	2	3	4	5	6

Average similarity: 36.31%

Prediction accuracy: 23%

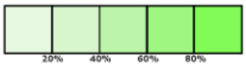

Print Toxicity Report

Name	
Molweight	308.35
Number of hydrogen bond acceptors	6
Number of hydrogen bond donors	2
Number of atoms	21
Number of bonds	23
Number of rotable bonds	2
Molecular refractivity	85.03
Topological Polar Surface Area	92.88
octanol/water partition coefficient(logP)	3.21

1	Sars-Cov				
Molecule	Predicted Outcome	Probability	Probability Active	Probability Inactive	Predicted Reliability
COC1=CC=C(C=C1)C1NC(=O)NC2=C1S(=O)(=O)CCC2	Inactive	0.8	0.2	0.8	reliable

2	Dengue larvicida				
Molecule	Predicted Outcome	Probability	Probability Active	Probability Inactive	Predicted Reliability
COC1=CC=C(C=C1)C1NC(=O)NC2=C1S(=O)(=O)CCC2	Active	1.0	1.0	0.0	reliable

3	Acetylcholinesterase				
Molecule	Predicted Outcome	Probability	Probability Active	Probability Inactive	Predicted Reliability
COC1=CC=C(C=C1)C1NC(=O)NC2=C1S(=O)(=O)CCC2	Inactive	1.0	0.0	1.0	reliable

4	C_albicans				
Molecule	Predicted Outcome	Probability	Probability Active	Probability Inactive	Predicted Reliability
COC1=CC=C(C=C1)C1NC(=O)NC2=C1S(=O)(=O)CCC2	Active	0.8	0.8	0.2	reliable

5	Salmonella				
Molecule	Predicted Outcome	Probability	Probability Active	Probability Inactive	Predicted Reliability
COC1=CC=C(C=C1)C1NC(=O)NC2=C1S(=O)(=O)CCC2	Inactive	1.0	0.0	1.0	reliable

6	Hepatite C - Type1				
Molecule	Predicted Outcome	Probability	Probability Active	Probability Inactive	Predicted Reliability
COC1=CC=C(C=C1)C1NC(=O)NC2=C1S(=O)(=O)CCC2	Inactive	0.8	0.2	0.8	unreliable

7	E_coli				
Molecule	Predicted Outcome	Probability	Probability Active	Probability Inactive	Predicted Reliability
COC1=CC=C(C=C1)C1NC(=O)NC2=C1S(=O)(=O)CCC2	Inactive	0.6	0.4	0.6	reliable

8	Hepatite C - NS3-protease helicase				
Molecule	Predicted Outcome	Probability	Probability Active	Probability Inactive	Predicted Reliability
COC1=CC=C(C=C1)C1NC(=O)NC2=C1S(=O)(=O)CCC2	Inactive	1.0	0.0	1.0	reliable

9	Hepatite C - Serine protease				
Molecule	Predicted Outcome	Probability	Probability Active	Probability Inactive	Predicted Reliability
COC1=CC=C(C=C1)C1NC(=O)NC2=C1S(=O)(=O)CCC2	Inactive	1.0	0.0	1.0	unreliable

10	Hepatite C - RNA dependent				
Molecule	Predicted Outcome	Probability	Probability Active	Probability Inactive	Predicted Reliability
COC1=CC=C(C=C1)C1NC(=O)NC2=C1S(=O)(=O)CCC2	Inactive	1.0	0.0	1.0	reliable

11	Leishmania braziliensis				
Molecule	Predicted Outcome	Probability	Probability Active	Probability Inactive	Predicted Reliability
COC1=CC=C(C=C1)C1NC(=O)NC2=C1S(=O)(=O)CCC2	Inactive	0.8	0.2	0.8	reliable

12	Leishmania infantum - Promastigota				
Molecule	Predicted Outcome	Probability	Probability Active	Probability Inactive	Predicted Reliability
COC1=CC=C(C=C1)C1NC(=O)NC2=C1S(=O)(=O)CCC2	Inactive	0.8	0.2	0.8	reliable

13	Leishmania amazonensis - Promastigota				
Molecule	Predicted Outcome	Probability	Probability Active	Probability Inactive	Predicted Reliability
COC1=CC=C(C=C1)C1NC(=O)NC2=C1S(=O)(=O)CCC2	Inactive	0.6	0.4	0.6	reliable

14	Drosophila melanogaster				
Molecule	Predicted Outcome	Probability	Probability Active	Probability Inactive	Predicted Reliability
COC1=CC=C(C=C1)C1NC(=O)NC2=C1S(=O)(=O)CCC2	Inactive	1.0	0.0	1.0	unreliable

15	Leishmania major				
Molecule	Predicted Outcome	Probability	Probability Active	Probability Inactive	Predicted Reliability
COC1=CC=C(C=C1)C1NC(=O)NC2=C1S(=O)(=O)CCC2	Inactive	1.0	0.0	1.0	reliable

16	Alphis gossypii				
Molecule	Predicted Outcome	Probability	Probability Active	Probability Inactive	Predicted Reliability
COC1=CC=C(C=C1)C1NC(=O)NC2=C1S(=O)(=O)CCC2	Active	1.0	1.0	0.0	reliable

17	Alzheimer - iNOS				
Molecule	Predicted Outcome	Probability	Probability Active	Probability Inactive	Predicted Reliability
COC1=CC=C(C=C1)C1NC(=O)NC2=C1S(=O)(=O)CCC2	Inactive	0.8	0.2	0.8	reliable

18	Alzheimer - COX2				
Molecule	Predicted Outcome	Probability	Probability Active	Probability Inactive	Predicted Reliability
COC1=CC=C(C=C1)C1NC(=O)NC2=C1S(=O)(=O)CCC2	Inactive	0.8	0.2	0.8	reliable

19	Alzheimer - NADPH				
Molecule	Predicted Outcome	Probability	Probability Active	Probability Inactive	Predicted Reliability
COC1=CC=C(C=C1)C1NC(=O)NC2=C1S(=O)(=O)CCC2	Inactive	0.6	0.4	0.6	reliable

20	Alzheimer - JNK-3				
Molecule	Predicted Outcome	Probability	Probability Active	Probability Inactive	Predicted Reliability
COC1=CC=C(C=C1)C1NC(=O)NC2=C1S(=O)(=O)CCC2	Inactive	1.0	0.0	1.0	reliable

21	Alzheimer - PDE5				
Molecule	Predicted Outcome	Probability	Probability Active	Probability Inactive	Predicted Reliability
COC1=CC=C(C=C1)C1NC(=O)NC2=C1S(=O)(=O)CCC2	Inactive	0.8	0.2	0.8	reliable

22	Epimastigote Chagas				
Molecule	Predicted Outcome	Probability	Probability Active	Probability Inactive	Predicted Reliability
COC1=CC=C(C=C1)C1NC(=O)NC2=C1S(=O)(=O)CCC2	Inactive	1.0	0.0	1.0	reliable

23	Amastigote Ldonovani				
Molecule	Predicted Outcome	Probability	Probability Active	Probability Inactive	Predicted Reliability
COC1=CC=C(C=C1)C1NC(=O)NC2=C1S(=O)(=O)CCC2	Inactive	1.0	0.0	1.0	reliable

24	Amastigote Chagas				
Molecule	Predicted Outcome	Probability	Probability Active	Probability Inactive	Predicted Reliability
COC1=CC=C(C=C1)C1NC(=O)NC2=C1S(=O)(=O)CCC2	Inactive	1.0	0.0	1.0	reliable

25	Promastigote Ldonovani				
Molecule	Predicted Outcome	Probability	Probability Active	Probability Inactive	Predicted Reliability
COC1=CC=C(C=C1)C1NC(=O)NC2=C1S(=O)(=O)CCC2	Inactive	1.0	0.0	1.0	reliable

26	PTR L major				
Molecule	Predicted Outcome	Probability	Probability Active	Probability Inactive	Predicted Reliability
COC1=CC=C(C=C1)C1NC(=O)NC2=C1S(=O)(=O)CCC2	Inactive	0.8	0.2	0.8	reliable

27	Lamazonensis_amastigota				
Molecule	Predicted Outcome	Probability	Probability Active	Probability Inactive	Predicted Reliability
COC1=CC=C(C=C1)C1NC(=O)NC2=C1S(=O)(=O)CCC2	Inactive	1.0	0.0	1.0	reliable

28	Lamazonensis_promastigota				
Molecule	Predicted Outcome	Probability	Probability Active	Probability Inactive	Predicted Reliability
COC1=CC=C(C=C1)C1NC(=O)NC2=C1S(=O)(=O)CCC2	Inactive	1.0	0.0	1.0	reliable

29	Tripomastigote Chagas				
Molecule	Predicted Outcome	Probability	Probability Active	Probability Inactive	Predicted Reliability
COC1=CC=C(C=C1)C1NC(=O)NC2=C1S(=O)(=O)CCC2	Active	1.0	1.0	0.0	reliable

30	Tcruzi_trypomastigota				
Molecule	Predicted Outcome	Probability	Probability Active	Probability Inactive	Predicted Reliability
COC1=CC=C(C=C1)C1NC(=O)NC2=C1S(=O)(=O)CCC2	Inactive	0.8	0.2	0.8	reliable

31	Tcruzi_amastigota				
Molecule	Predicted Outcome	Probability	Probability Active	Probability Inactive	Predicted Reliability
COC1=CC=C(C=C1)C1NC(=O)NC2=C1S(=O)(=O)CCC2	Active	0.6	0.6	0.4	reliable

32	Tcruzi_epimastigota				
Molecule	Predicted Outcome	Probability	Probability Active	Probability Inactive	Predicted Reliability
COC1=CC=C(C=C1)C1NC(=O)NC2=C1S(=O)(=O)CCC2	Active	1.0	1.0	0.0	reliable

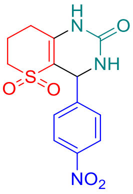

2l O=C1NC(C2=CC=C(C=C2)N(=O)=O)C2=C(CCCS2(=O)=O)N1

Molecule 1

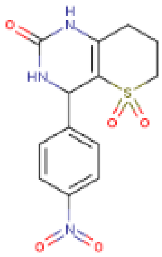

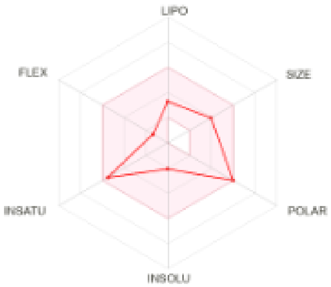

SMILES O=C1NC2=C(C(N1)c1ccc(cc1)N(=O)=O)S(=O)(=O)CCC2

Physicochemical Properties	
Formula	C13H13N3O5S
Molecular weight	323.32 g/mol
Num. heavy atoms	22
Num. arom. heavy atoms	6
Fraction Csp3	0.31
Num. rotatable bonds	2
Num. H-bond acceptors	5
Num. H-bond donors	2
Molar Refractivity	87.36
TPSA	129.47 Å²

Lipophilicity	
Log $P_{o/w}$ (iLOGP)	1.37
Log $P_{o/w}$ (XLOGP3)	0.25
Log $P_{o/w}$ (WLOGP)	1.89
Log $P_{o/w}$ (MLOGP)	0.15
Log $P_{o/w}$ (SILICOS-IT)	-0.79
Consensus Log $P_{o/w}$	0.57

Water Solubility	
Log *S* (ESOL)	-2.07
Solubility	2.74e+00 mg/ml ; 8.47e-03 mol/l
Class	Soluble
Log *S* (Ali)	-2.53
Solubility	9.55e-01 mg/ml ; 2.95e-03 mol/l
Class	Soluble
Log *S* (SILICOS-IT)	-3.83
Solubility	4.78e-02 mg/ml ; 1.48e-04 mol/l
Class	Soluble

Pharmacokinetics	
GI absorption	High
BBB permeant	No
P-gp substrate	No
CYP1A2 inhibitor	No
CYP2C19 inhibitor	No
CYP2C9 inhibitor	No
CYP2D6 inhibitor	No
CYP3A4 inhibitor	No
Log K_p (skin permeation)	-8.09 cm/s

Druglikeness	
Lipinski	Yes; 0 violation
Ghose	Yes
Veber	Yes
Egan	Yes
Muegge	Yes
Bioavailability Score	0.55

Medicinal Chemistry	
PAINS	0 alert
Brenk	1 alert: nitro_group
Leadlikeness	Yes
Synthetic accessibility	3.88

Oral toxicity prediction results for input compound

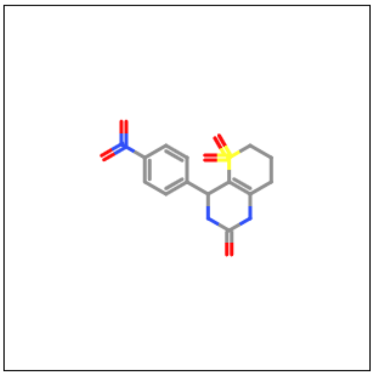

Predicted LD50: 1000mg/kg

Predicted Toxicity Class: 4

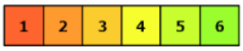

Average similarity: 36.28%

Prediction accuracy: 23%

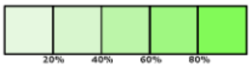

Print Toxicity Report

Name	
Molweight	323.32
Number of hydrogen bond acceptors	7
Number of hydrogen bond donors	2
Number of atoms	22
Number of bonds	24
Number of rotable bonds	2
Molecular refractivity	87.36
Topological Polar Surface Area	129.47
octanol/water partition coefficient(logP)	3.63

1	Sars-Cov				
Molecule	Predicted Outcome	Probability	Probability Active	Probability Inactive	Predicted Reliability
O=C1NC(C2=CC=C(C=C2)N(=O)=O)C2=C(CCCS2(=O)=O)N1	Inactive	1.0	0.0	1.0	reliable

2	Dengue larvicida				
Molecule	Predicted Outcome	Probability	Probability Active	Probability Inactive	Predicted Reliability
O=C1NC(C2=CC=C(C=C2)N(=O)=O)C2=C(CCCS2(=O)=O)N1	Active	1.0	1.0	0.0	reliable

3	Salmonella				
Molecule	Predicted Outcome	Probability	Probability Active	Probability Inactive	Predicted Reliability
O=C1NC(C2=CC=C(C=C2)N(=O)=O)C2=C(CCCS2(=O)=O)N1	Inactive	0.6	0.4	0.6	reliable

4	Acetylcholinesterase				
Molecule	Predicted Outcome	Probability	Probability Active	Probability Inactive	Predicted Reliability
O=C1NC(C2=CC=C(C=C2)N(=O)=O)C2=C(CCCS2(=O)=O)N1	Inactive	1.0	0.0	1.0	reliable

5	Hepatite C - Type1				
Molecule	Predicted Outcome	Probability	Probability Active	Probability Inactive	Predicted Reliability
O=C1NC(C2=CC=C(C=C2)N(=O)=O)C2=C(CCCS2(=O)=O)N1	Inactive	0.6	0.4	0.6	unreliable

6	Hepatite C - NS3-protease helicase				
Molecule	Predicted Outcome	Probability	Probability Active	Probability Inactive	Predicted Reliability
O=C1NC(C2=CC=C(C=C2)N(=O)=O)C2=C(CCCS2(=O)=O)N1	Inactive	1.0	0.0	1.0	reliable

7	E_coli				
Molecule	Predicted Outcome	Probability	Probability Active	Probability Inactive	Predicted Reliability
O=C1NC(C2=CC=C(C=C2)N(=O)=O)C2=C(CCCS2(=O)=O)N1	Inactive	0.6	0.4	0.6	reliable

8	C_albicans				
Molecule	Predicted Outcome	Probability	Probability Active	Probability Inactive	Predicted Reliability
O=C1NC(C2=CC=C(C=C2)N(=O)=O)C2=C(CCCS2(=O)=O)N1	Active	1.0	1.0	0.0	reliable

9	Hepatite C - Serine protease				
Molecule	Predicted Outcome	Probability	Probability Active	Probability Inactive	Predicted Reliability
O=C1NC(C2=CC=C(C=C2)N(=O)=O)C2=C(CCCS2(=O)=O)N1	Inactive	0.8	0.2	0.8	unreliable

10	Leishmania amazonensis - Promastigota				
Molecule	Predicted Outcome	Probability	Probability Active	Probability Inactive	Predicted Reliability
O=C1NC(C2=CC=C(C=C2)N(=O)=O)C2=C(CCCS2(=O)=O)N1	Active	0.8	0.8	0.2	reliable

11	Hepatite C - RNA dependent				
Molecule	Predicted Outcome	Probability	Probability Active	Probability Inactive	Predicted Reliability
O=C1NC(C2=CC=C(C=C2)N(=O)=O)C2=C(CCCS2(=O)=O)N1	Inactive	1.0	0.0	1.0	reliable

12	Leishmania infantum - Promastigota				
Molecule	Predicted Outcome	Probability	Probability Active	Probability Inactive	Predicted Reliability
O=C1NC(C2=CC=C(C=C2)N(=O)=O)C2=C(CCCS2(=O)=O)N1	Inactive	0.6	0.4	0.6	reliable

13	Leishmania braziliensis				
Molecule	Predicted Outcome	Probability	Probability Active	Probability Inactive	Predicted Reliability
O=C1NC(C2=CC=C(C=C2)N(=O)=O)C2=C(CCCS2(=O)=O)N1	Inactive	0.8	0.2	0.8	reliable

14	Drosophila melanogaster				
Molecule	Predicted Outcome	Probability	Probability Active	Probability Inactive	Predicted Reliability
O=C1NC(C2=CC=C(C=C2)N(=O)=O)C2=C(CCCS2(=O)=O)N1	Inactive	1.0	0.0	1.0	unreliable

15	Leishmania major				
Molecule	Predicted Outcome	Probability	Probability Active	Probability Inactive	Predicted Reliability
O=C1NC(C2=CC=C(C=C2)N(=O)=O)C2=C(CCCS2(=O)=O)N1	Inactive	1.0	0.0	1.0	reliable

16	Alphis gossypii				
Molecule	Predicted Outcome	Probability	Probability Active	Probability Inactive	Predicted Reliability
O=C1NC(C2=CC=C(C=C2)N(=O)=O)C2=C(CCCS2(=O)=O)N1	Active	1.0	1.0	0.0	reliable

17	Alzheimer - COX2				
Molecule	Predicted Outcome	Probability	Probability Active	Probability Inactive	Predicted Reliability
O=C1NC(C2=CC=C(C=C2)N(=O)=O)C2=C(CCCS2(=O)=O)N1	Inactive	0.8	0.2	0.8	reliable

18	Alzheimer - NADPH				
Molecule	Predicted Outcome	Probability	Probability Active	Probability Inactive	Predicted Reliability
O=C1NC(C2=CC=C(C=C2)N(=O)=O)C2=C(CCCS2(=O)=O)N1	Inactive	0.6	0.4	0.6	reliable

19	Alzheimer - iNOS				
Molecule	Predicted Outcome	Probability	Probability Active	Probability Inactive	Predicted Reliability
O=C1NC(C2=CC=C(C=C2)N(=O)=O)C2=C(CCCS2(=O)=O)N1	Inactive	0.8	0.2	0.8	reliable

20	Alzheimer - JNK-3				
Molecule	Predicted Outcome	Probability	Probability Active	Probability Inactive	Predicted Reliability
O=C1NC(C2=CC=C(C=C2)N(=O)=O)C2=C(CCCS2(=O)=O)N1	Inactive	1.0	0.0	1.0	reliable

21	Alzheimer - PDE5				
Molecule	Predicted Outcome	Probability	Probability Active	Probability Inactive	Predicted Reliability
O=C1NC(C2=CC=C(C=C2)N(=O)=O)C2=C(CCCS2(=O)=O)N1	Inactive	1.0	0.0	1.0	reliable

22	Amastigote Chagas				
Molecule	Predicted Outcome	Probability	Probability Active	Probability Inactive	Predicted Reliability
O=C1NC(C2=CC=C(C=C2)N(=O)=O)C2=C(CCCS2(=O)=O)N1	Inactive	1.0	0.0	1.0	reliable

23	Amastigote Ldonovani				
Molecule	Predicted Outcome	Probability	Probability Active	Probability Inactive	Predicted Reliability
O=C1NC(C2=CC=C(C=C2)N(=O)=O)C2=C(CCCS2(=O)=O)N1	Inactive	0.8	0.2	0.8	reliable

24	Promastigote Ldonovani				
**Molecule**	**Predicted Outcome**	**Probability**	**Probability Active**	**Probability Inactive**	**Predicted Reliability**
O=C1NC(C2=CC=C(C=C2)N(=O)=O)C2=C(CCCS2(=O)=O)N1	Inactive	0.8	0.2	0.8	reliable

25	Epimastigote Chagas				
**Molecule**	**Predicted Outcome**	**Probability**	**Probability Active**	**Probability Inactive**	**Predicted Reliability**
O=C1NC(C2=CC=C(C=C2)N(=O)=O)C2=C(CCCS2(=O)=O)N1	Inactive	1.0	0.0	1.0	reliable

26	Lamazonensis_amastigota				
**Molecule**	**Predicted Outcome**	**Probability**	**Probability Active**	**Probability Inactive**	**Predicted Reliability**
O=C1NC(C2=CC=C(C=C2)N(=O)=O)C2=C(CCCS2(=O)=O)N1	Inactive	0.8	0.2	0.8	reliable

27	PTR L major				
**Molecule**	**Predicted Outcome**	**Probability**	**Probability Active**	**Probability Inactive**	**Predicted Reliability**
O=C1NC(C2=CC=C(C=C2)N(=O)=O)C2=C(CCCS2(=O)=O)N1	Inactive	0.8	0.2	0.8	reliable

28	Lamazonensis_promastigota				
**Molecule**	**Predicted Outcome**	**Probability**	**Probability Active**	**Probability Inactive**	**Predicted Reliability**
O=C1NC(C2=CC=C(C=C2)N(=O)=O)C2=C(CCCS2(=O)=O)N1	Inactive	1.0	0.0	1.0	reliable

29	Tripomastigote Chagas				
**Molecule**	**Predicted Outcome**	**Probability**	**Probability Active**	**Probability Inactive**	**Predicted Reliability**
O=C1NC(C2=CC=C(C=C2)N(=O)=O)C2=C(CCCS2(=O)=O)N1	Active	1.0	1.0	0.0	reliable

30	Tcruzi_amastigota				
**Molecule**	**Predicted Outcome**	**Probability**	**Probability Active**	**Probability Inactive**	**Predicted Reliability**
O=C1NC(C2=CC=C(C=C2)N(=O)=O)C2=C(CCCS2(=O)=O)N1	Active	0.6	0.6	0.4	reliable

31	Tcruzi_epimastigota				
**Molecule**	**Predicted Outcome**	**Probability**	**Probability Active**	**Probability Inactive**	**Predicted Reliability**
O=C1NC(C2=CC=C(C=C2)N(=O)=O)C2=C(CCCS2(=O)=O)N1	Active	0.6	0.6	0.4	reliable

32	Tcruzi_trypomastigota				
**Molecule**	**Predicted Outcome**	**Probability**	**Probability Active**	**Probability Inactive**	**Predicted Reliability**
O=C1NC(C2=CC=C(C=C2)N(=O)=O)C2=C(CCCS2(=O)=O)N1	Inactive	1.0	0.0	1.0	reliable

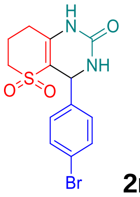

2m BrC1=CC=C(C=C1)C1NC(=O)NC2=C1S(=O)(=O)CCC2

Molecule 1

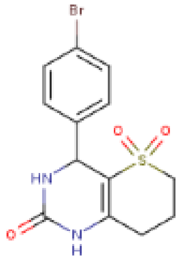

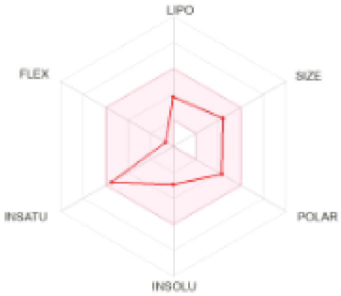

SMILES O=C1NC2=C(C(N1)c1ccc(cc1)Br)S(=O)(=O)CCC2

Physicochemical Properties	
Formula	C13H13BrN2O3S
Molecular weight	357.22 g/mol
Num. heavy atoms	20
Num. arom. heavy atoms	6
Fraction Csp3	0.31
Num. rotatable bonds	1
Num. H-bond acceptors	3
Num. H-bond donors	2
Molar Refractivity	86.23
TPSA	83.65 Å²

Lipophilicity	
Log $P_{o/w}$ (iLOGP)	1.78
Log $P_{o/w}$ (XLOGP3)	1.12
Log $P_{o/w}$ (WLOGP)	2.22
Log $P_{o/w}$ (MLOGP)	1.81
Log $P_{o/w}$ (SILICOS-IT)	1.66
Consensus Log $P_{o/w}$	1.72

Water Solubility	
Log *S* (ESOL)	-2.92
Solubility	4.33e-01 mg/ml ; 1.21e-03 mol/l
Class	Soluble
Log *S* (Ali)	-2.47
Solubility	1.21e+00 mg/ml ; 3.39e-03 mol/l
Class	Soluble
Log *S* (SILICOS-IT)	-4.80
Solubility	5.65e-03 mg/ml ; 1.58e-05 mol/l
Class	Moderately soluble

Pharmacokinetics	
GI absorption	High
BBB permeant	No
P-gp substrate	Yes
CYP1A2 inhibitor	No
CYP2C19 inhibitor	Yes
CYP2C9 inhibitor	No
CYP2D6 inhibitor	No
CYP3A4 inhibitor	No
Log K_p (skin permeation)	-7.68 cm/s

Druglikeness	
Lipinski	Yes; 0 violation
Ghose	Yes
Veber	Yes
Egan	Yes
Muegge	Yes
Bioavailability Score	0.55

Medicinal Chemistry	
PAINS	0 alert
Brenk	0 alert
Leadlikeness	No; 1 violation: MW>350
Synthetic accessibility	3.87

Oral toxicity prediction results for input compound

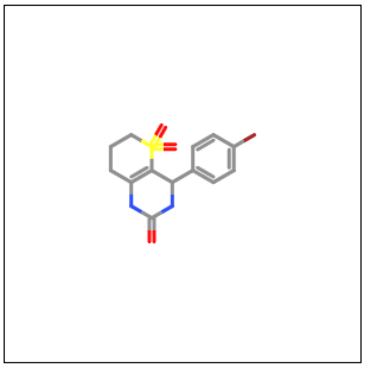

Predicted LD50: 1000mg/kg

Predicted Toxicity Class: 4

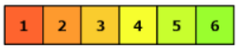

Average similarity: 34.94%

Prediction accuracy: 23%

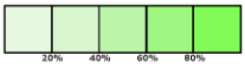

Print Toxicity Report

Name	
Molweight	357.22
Number of hydrogen bond acceptors	5
Number of hydrogen bond donors	2
Number of atoms	20
Number of bonds	22
Number of rotable bonds	1
Molecular refractivity	86.23
Topological Polar Surface Area	83.65
octanol/water partition coefficient(logP)	3.96

1	Dengue larvicida				
**Molecule**	**Predicted Outcome**	**Probability**	**Probability Active**	**Probability Inactive**	**Predicted Reliability**
BrC1=CC=C(C=C1)C1NC(=O)NC2=C1S(=O)(=O)CCC2	Active	1.0	1.0	0.0	reliable

2	Sars-Cov				
**Molecule**	**Predicted Outcome**	**Probability**	**Probability Active**	**Probability Inactive**	**Predicted Reliability**
BrC1=CC=C(C=C1)C1NC(=O)NC2=C1S(=O)(=O)CCC2	Inactive	0.8	0.2	0.8	reliable

3	Acetylcholinesterase				
**Molecule**	**Predicted Outcome**	**Probability**	**Probability Active**	**Probability Inactive**	**Predicted Reliability**
BrC1=CC=C(C=C1)C1NC(=O)NC2=C1S(=O)(=O)CCC2	Inactive	1.0	0.0	1.0	reliable

4	E_coli				
**Molecule**	**Predicted Outcome**	**Probability**	**Probability Active**	**Probability Inactive**	**Predicted Reliability**
BrC1=CC=C(C=C1)C1NC(=O)NC2=C1S(=O)(=O)CCC2	Inactive	0.6	0.4	0.6	reliable

5	C_albicans				
**Molecule**	**Predicted Outcome**	**Probability**	**Probability Active**	**Probability Inactive**	**Predicted Reliability**
BrC1=CC=C(C=C1)C1NC(=O)NC2=C1S(=O)(=O)CCC2	Active	1.0	1.0	0.0	reliable

6	Salmonella				
**Molecule**	**Predicted Outcome**	**Probability**	**Probability Active**	**Probability Inactive**	**Predicted Reliability**
BrC1=CC=C(C=C1)C1NC(=O)NC2=C1S(=O)(=O)CCC2	Inactive	1.0	0.0	1.0	reliable

7	Hepatite C - Type1				
**Molecule**	**Predicted Outcome**	**Probability**	**Probability Active**	**Probability Inactive**	**Predicted Reliability**
BrC1=CC=C(C=C1)C1NC(=O)NC2=C1S(=O)(=O)CCC2	Inactive	0.8	0.2	0.8	reliable

8	Hepatite C - NS3-protease helicase				
**Molecule**	**Predicted Outcome**	**Probability**	**Probability Active**	**Probability Inactive**	**Predicted Reliability**
BrC1=CC=C(C=C1)C1NC(=O)NC2=C1S(=O)(=O)CCC2	Inactive	1.0	0.0	1.0	reliable

9	Hepatite C - Serine protease				
**Molecule**	**Predicted Outcome**	**Probability**	**Probability Active**	**Probability Inactive**	**Predicted Reliability**
BrC1=CC=C(C=C1)C1NC(=O)NC2=C1S(=O)(=O)CCC2	Inactive	0.8	0.2	0.8	unreliable

10	Hepatite C - RNA dependent				
**Molecule**	**Predicted Outcome**	**Probability**	**Probability Active**	**Probability Inactive**	**Predicted Reliability**
BrC1=CC=C(C=C1)C1NC(=O)NC2=C1S(=O)(=O)CCC2	Inactive	1.0	0.0	1.0	reliable

11	Leishmania amazonensis - Promastigota				
**Molecule**	**Predicted Outcome**	**Probability**	**Probability Active**	**Probability Inactive**	**Predicted Reliability**
BrC1=CC=C(C=C1)C1NC(=O)NC2=C1S(=O)(=O)CCC2	Inactive	1.0	0.0	1.0	reliable

12	Leishmania infantum - Promastigota				
**Molecule**	**Predicted Outcome**	**Probability**	**Probability Active**	**Probability Inactive**	**Predicted Reliability**
BrC1=CC=C(C=C1)C1NC(=O)NC2=C1S(=O)(=O)CCC2	Inactive	0.8	0.2	0.8	reliable

13	Leishmania braziliensis				
**Molecule**	**Predicted Outcome**	**Probability**	**Probability Active**	**Probability Inactive**	**Predicted Reliability**
BrC1=CC=C(C=C1)C1NC(=O)NC2=C1S(=O)(=O)CCC2	Inactive	0.8	0.2	0.8	reliable

14	Drosophila melanogaster				
**Molecule**	**Predicted Outcome**	**Probability**	**Probability Active**	**Probability Inactive**	**Predicted Reliability**
BrC1=CC=C(C=C1)C1NC(=O)NC2=C1S(=O)(=O)CCC2	Inactive	1.0	0.0	1.0	unreliable

15	Leishmania major				
**Molecule**	**Predicted Outcome**	**Probability**	**Probability Active**	**Probability Inactive**	**Predicted Reliability**
BrC1=CC=C(C=C1)C1NC(=O)NC2=C1S(=O)(=O)CCC2	Inactive	1.0	0.0	1.0	reliable

16	Alphis gossypii				
**Molecule**	**Predicted Outcome**	**Probability**	**Probability Active**	**Probability Inactive**	**Predicted Reliability**
BrC1=CC=C(C=C1)C1NC(=O)NC2=C1S(=O)(=O)CCC2	Active	1.0	1.0	0.0	reliable

17	Alzheimer - NADPH				
Molecule	Predicted Outcome	Probability	Probability Active	Probability Inactive	Predicted Reliability
BrC1=CC=C(C=C1)C1NC(=O)NC2=C1S(=O)(=O)CCC2	Inactive	0.6	0.4	0.6	reliable

18	Alzheimer - iNOS				
Molecule	Predicted Outcome	Probability	Probability Active	Probability Inactive	Predicted Reliability
BrC1=CC=C(C=C1)C1NC(=O)NC2=C1S(=O)(=O)CCC2	Inactive	0.8	0.2	0.8	reliable

19	Alzheimer - COX2				
Molecule	Predicted Outcome	Probability	Probability Active	Probability Inactive	Predicted Reliability
BrC1=CC=C(C=C1)C1NC(=O)NC2=C1S(=O)(=O)CCC2	Inactive	0.8	0.2	0.8	reliable

20	Alzheimer - JNK-3				
Molecule	Predicted Outcome	Probability	Probability Active	Probability Inactive	Predicted Reliability
BrC1=CC=C(C=C1)C1NC(=O)NC2=C1S(=O)(=O)CCC2	Inactive	1.0	0.0	1.0	reliable

21	Alzheimer - PDE5				
Molecule	Predicted Outcome	Probability	Probability Active	Probability Inactive	Predicted Reliability
BrC1=CC=C(C=C1)C1NC(=O)NC2=C1S(=O)(=O)CCC2	Inactive	1.0	0.0	1.0	reliable

22	Amastigote Ldonovani				
Molecule	Predicted Outcome	Probability	Probability Active	Probability Inactive	Predicted Reliability
BrC1=CC=C(C=C1)C1NC(=O)NC2=C1S(=O)(=O)CCC2	Inactive	1.0	0.0	1.0	reliable

23	Amastigote Chagas				
Molecule	Predicted Outcome	Probability	Probability Active	Probability Inactive	Predicted Reliability
BrC1=CC=C(C=C1)C1NC(=O)NC2=C1S(=O)(=O)CCC2	Inactive	1.0	0.0	1.0	reliable

24	Epimastigote Chagas				
Molecule	Predicted Outcome	Probability	Probability Active	Probability Inactive	Predicted Reliability
BrC1=CC=C(C=C1)C1NC(=O)NC2=C1S(=O)(=O)CCC2	Inactive	1.0	0.0	1.0	reliable

25	Promastigote Ldonovani				
Molecule	Predicted Outcome	Probability	Probability Active	Probability Inactive	Predicted Reliability
BrC1=CC=C(C=C1)C1NC(=O)NC2=C1S(=O)(=O)CCC2	Inactive	0.8	0.2	0.8	reliable

26	PTR L major				
Molecule	Predicted Outcome	Probability	Probability Active	Probability Inactive	Predicted Reliability
BrC1=CC=C(C=C1)C1NC(=O)NC2=C1S(=O)(=O)CCC2	Inactive	0.8	0.2	0.8	reliable

27	Tripomastigote Chagas				
Molecule	Predicted Outcome	Probability	Probability Active	Probability Inactive	Predicted Reliability
BrC1=CC=C(C=C1)C1NC(=O)NC2=C1S(=O)(=O)CCC2	Active	0.8	0.8	0.2	reliable

28	Lamazonensis_amastigota				
Molecule	Predicted Outcome	Probability	Probability Active	Probability Inactive	Predicted Reliability
BrC1=CC=C(C=C1)C1NC(=O)NC2=C1S(=O)(=O)CCC2	Inactive	0.8	0.2	0.8	reliable

29	Lamazonensis_promastigota				
Molecule	Predicted Outcome	Probability	Probability Active	Probability Inactive	Predicted Reliability
BrC1=CC=C(C=C1)C1NC(=O)NC2=C1S(=O)(=O)CCC2	Inactive	1.0	0.0	1.0	reliable

30	Tcruzi_trypomastigota				
Molecule	Predicted Outcome	Probability	Probability Active	Probability Inactive	Predicted Reliability
BrC1=CC=C(C=C1)C1NC(=O)NC2=C1S(=O)(=O)CCC2	Inactive	1.0	0.0	1.0	reliable

31	Tcruzi_amastigota				
Molecule	Predicted Outcome	Probability	Probability Active	Probability Inactive	Predicted Reliability
BrC1=CC=C(C=C1)C1NC(=O)NC2=C1S(=O)(=O)CCC2	Inactive	0.6	0.4	0.6	reliable

32	Tcruzi_epimastigota				
Molecule	Predicted Outcome	Probability	Probability Active	Probability Inactive	Predicted Reliability
BrC1=CC=C(C=C1)C1NC(=O)NC2=C1S(=O)(=O)CCC2	Active	1.0	1.0	0.0	reliable

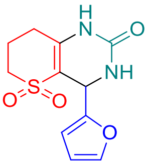

2n O=C1NC(C2=CC=CO2)C2=C(CCCS2(=O)=O)N1

Molecule 1

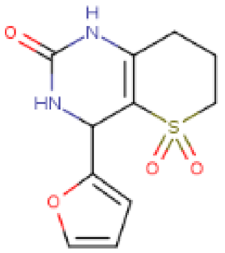

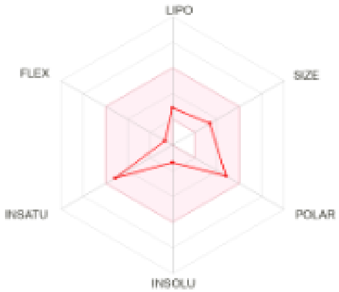

SMILES O=C1NC2=C(C(N1)c1ccco1)S(=O)(=O)CCC2

Physicochemical Properties	
Formula	C11H12N2O4S
Molecular weight	268.29 g/mol
Num. heavy atoms	18
Num. arom. heavy atoms	5
Fraction Csp3	0.36
Num. rotatable bonds	1
Num. H-bond acceptors	4
Num. H-bond donors	2
Molar Refractivity	70.80
TPSA	96.79 Å²

Lipophilicity	
Log $P_{o/w}$ (iLOGP)	1.34
Log $P_{o/w}$ (XLOGP3)	-0.47
Log $P_{o/w}$ (WLOGP)	1.05
Log $P_{o/w}$ (MLOGP)	-0.18
Log $P_{o/w}$ (SILICOS-IT)	0.43
Consensus Log $P_{o/w}$	0.43

Water Solubility	
Log *S* (ESOL)	-1.35
Solubility	1.21e+01 mg/ml ; 4.50e-02 mol/l
Class	Very soluble
Log *S* (Ali)	-1.10
Solubility	2.15e+01 mg/ml ; 8.02e-02 mol/l
Class	Very soluble
Log *S* (SILICOS-IT)	-3.21
Solubility	1.66e-01 mg/ml ; 6.19e-04 mol/l
Class	Soluble

Pharmacokinetics	
GI absorption	High
BBB permeant	No
P-gp substrate	No
CYP1A2 inhibitor	No
CYP2C19 inhibitor	No
CYP2C9 inhibitor	No
CYP2D6 inhibitor	No
CYP3A4 inhibitor	No
Log K_p (skin permeation)	-8.27 cm/s

Druglikeness	
Lipinski	Yes; 0 violation
Ghose	Yes
Veber	Yes
Egan	Yes
Muegge	Yes
Bioavailability Score	0.55

Medicinal Chemistry	
PAINS	0 alert
Brenk	0 alert
Leadlikeness	Yes
Synthetic accessibility	3.98

Oral toxicity prediction results for input compound

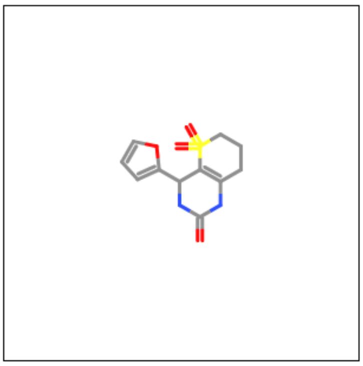

Predicted LD50: 927mg/kg

Predicted Toxicity Class: 4

1	2	3	4	5	6

Average similarity: 31.44%

Prediction accuracy: 23%

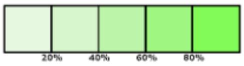

Print Toxicity Report

Name	
Molweight	268.29
Number of hydrogen bond acceptors	5
Number of hydrogen bond donors	2
Number of atoms	18
Number of bonds	20
Number of rotatable bonds	1
Molecular refractivity	70.8
Topological Polar Surface Area	96.79
octanol/water partition coefficient(logP)	2.79

1	Dengue larvicida				
Molecule	Predicted Outcome	Probability	Probability Active	Probability Inactive	Predicted Reliability
O=C1NC(C2=CC=CO2)C2=C(CCCS2(=O)=O)N1	Active	1.0	1.0	0.0	reliable

2	Sars-Cov				
Molecule	Predicted Outcome	Probability	Probability Active	Probability Inactive	Predicted Reliability
O=C1NC(C2=CC=CO2)C2=C(CCCS2(=O)=O)N1	Inactive	0.6	0.4	0.6	reliable

3	Acetylcholinesterase				
Molecule	Predicted Outcome	Probability	Probability Active	Probability Inactive	Predicted Reliability
O=C1NC(C2=CC=CO2)C2=C(CCCS2(=O)=O)N1	Inactive	1.0	0.0	1.0	reliable

4	E_coli				
Molecule	Predicted Outcome	Probability	Probability Active	Probability Inactive	Predicted Reliability
O=C1NC(C2=CC=CO2)C2=C(CCCS2(=O)=O)N1	Active	0.8	0.8	0.2	reliable

5	Salmonella				
Molecule	Predicted Outcome	Probability	Probability Active	Probability Inactive	Predicted Reliability
O=C1NC(C2=CC=CO2)C2=C(CCCS2(=O)=O)N1	Inactive	0.8	0.2	0.8	reliable

6	C_albicans				
Molecule	Predicted Outcome	Probability	Probability Active	Probability Inactive	Predicted Reliability
O=C1NC(C2=CC=CO2)C2=C(CCCS2(=O)=O)N1	Active	1.0	1.0	0.0	reliable

7	Hepatite C - Type1				
Molecule	Predicted Outcome	Probability	Probability Active	Probability Inactive	Predicted Reliability
O=C1NC(C2=CC=CO2)C2=C(CCCS2(=O)=O)N1	Inactive	0.8	0.2	0.8	reliable

8	Hepatite C - NS3-protease helicase				
Molecule	Predicted Outcome	Probability	Probability Active	Probability Inactive	Predicted Reliability
O=C1NC(C2=CC=CO2)C2=C(CCCS2(=O)=O)N1	Inactive	1.0	0.0	1.0	reliable

9	Hepatite C - Serine protease				
Molecule	Predicted Outcome	Probability	Probability Active	Probability Inactive	Predicted Reliability
O=C1NC(C2=CC=CO2)C2=C(CCCS2(=O)=O)N1	Inactive	1.0	0.0	1.0	unreliable

10	Hepatite C - RNA dependent				
Molecule	Predicted Outcome	Probability	Probability Active	Probability Inactive	Predicted Reliability
O=C1NC(C2=CC=CO2)C2=C(CCCS2(=O)=O)N1	Inactive	1.0	0.0	1.0	reliable

11	Leishmania infantum - Promastigota				
Molecule	Predicted Outcome	Probability	Probability Active	Probability Inactive	Predicted Reliability
O=C1NC(C2=CC=CO2)C2=C(CCCS2(=O)=O)N1	Inactive	0.8	0.2	0.8	reliable

12	Leishmania amazonensis - Promastigota				
Molecule	Predicted Outcome	Probability	Probability Active	Probability Inactive	Predicted Reliability
O=C1NC(C2=CC=CO2)C2=C(CCCS2(=O)=O)N1	Inactive	0.8	0.2	0.8	reliable

13	Drosophila melanogaster				
Molecule	Predicted Outcome	Probability	Probability Active	Probability Inactive	Predicted Reliability
O=C1NC(C2=CC=CO2)C2=C(CCCS2(=O)=O)N1	Inactive	1.0	0.0	1.0	unreliable

14	Leishmania braziliensis				
Molecule	Predicted Outcome	Probability	Probability Active	Probability Inactive	Predicted Reliability
O=C1NC(C2=CC=CO2)C2=C(CCCS2(=O)=O)N1	Active	0.8	0.8	0.2	reliable

15	Leishmania major				
Molecule	Predicted Outcome	Probability	Probability Active	Probability Inactive	Predicted Reliability
O=C1NC(C2=CC=CO2)C2=C(CCCS2(=O)=O)N1	Inactive	0.8	0.2	0.8	reliable

16	Alphis gossypii				
Molecule	Predicted Outcome	Probability	Probability Active	Probability Inactive	Predicted Reliability
O=C1NC(C2=CC=CO2)C2=C(CCCS2(=O)=O)N1	Active	1.0	1.0	0.0	reliable

17	Alzheimer - NADPH				
Molecule	Predicted Outcome	Probability	Probability Active	Probability Inactive	Predicted Reliability
O=C1NC(C2=CC=CO2)C2=C(CCCS2(=O)=O)N1	Inactive	0.6	0.4	0.6	reliable

18	Alzheimer - iNOS				
Molecule	Predicted Outcome	Probability	Probability Active	Probability Inactive	Predicted Reliability
O=C1NC(C2=CC=CO2)C2=C(CCCS2(=O)=O)N1	Inactive	0.6	0.4	0.6	reliable

19	Alzheimer - COX2				
Molecule	Predicted Outcome	Probability	Probability Active	Probability Inactive	Predicted Reliability
O=C1NC(C2=CC=CO2)C2=C(CCCS2(=O)=O)N1	Active	0.6	0.6	0.4	reliable

20	Alzheimer - JNK-3				
Molecule	Predicted Outcome	Probability	Probability Active	Probability Inactive	Predicted Reliability
O=C1NC(C2=CC=CO2)C2=C(CCCS2(=O)=O)N1	Inactive	1.0	0.0	1.0	reliable

21	Alzheimer - PDE5				
Molecule	Predicted Outcome	Probability	Probability Active	Probability Inactive	Predicted Reliability
O=C1NC(C2=CC=CO2)C2=C(CCCS2(=O)=O)N1	Inactive	1.0	0.0	1.0	reliable

22	Amastigote Ldonovani				
Molecule	Predicted Outcome	Probability	Probability Active	Probability Inactive	Predicted Reliability
O=C1NC(C2=CC=CO2)C2=C(CCCS2(=O)=O)N1	Inactive	1.0	0.0	1.0	reliable

23	Epimastigote Chagas				
Molecule	Predicted Outcome	Probability	Probability Active	Probability Inactive	Predicted Reliability
O=C1NC(C2=CC=CO2)C2=C(CCCS2(=O)=O)N1	Inactive	0.8	0.2	0.8	reliable

24	Amastigote Chagas				
Molecule	Predicted Outcome	Probability	Probability Active	Probability Inactive	Predicted Reliability
O=C1NC(C2=CC=CO2)C2=C(CCCS2(=O)=O)N1	Inactive	1.0	0.0	1.0	reliable

25	PTR L major				
Molecule	Predicted Outcome	Probability	Probability Active	Probability Inactive	Predicted Reliability
O=C1NC(C2=CC=CO2)C2=C(CCCS2(=O)=O)N1	Inactive	0.8	0.2	0.8	reliable

26	Promastigote Ldonovani				
Molecule	Predicted Outcome	Probability	Probability Active	Probability Inactive	Predicted Reliability
O=C1NC(C2=CC=CO2)C2=C(CCCS2(=O)=O)N1	Active	0.6	0.6	0.4	reliable

27	Tripomastigote Chagas				
Molecule	Predicted Outcome	Probability	Probability Active	Probability Inactive	Predicted Reliability
O=C1NC(C2=CC=CO2)C2=C(CCCS2(=O)=O)N1	Active	0.8	0.8	0.2	reliable

28	Lamazonensis_amastigota				
Molecule	Predicted Outcome	Probability	Probability Active	Probability Inactive	Predicted Reliability
O=C1NC(C2=CC=CO2)C2=C(CCCS2(=O)=O)N1	Inactive	1.0	0.0	1.0	reliable

29	Lamazonensis_promastigota				
Molecule	Predicted Outcome	Probability	Probability Active	Probability Inactive	Predicted Reliability
O=C1NC(C2=CC=CO2)C2=C(CCCS2(=O)=O)N1	Inactive	1.0	0.0	1.0	reliable

30	Tcruzi_amastigota				
Molecule	Predicted Outcome	Probability	Probability Active	Probability Inactive	Predicted Reliability
O=C1NC(C2=CC=CO2)C2=C(CCCS2(=O)=O)N1	Active	1.0	1.0	0.0	reliable

31	Tcruzi_epimastigota				
Molecule	Predicted Outcome	Probability	Probability Active	Probability Inactive	Predicted Reliability
O=C1NC(C2=CC=CO2)C2=C(CCCS2(=O)=O)N1	Inactive	0.8	0.2	0.8	reliable

32	Tcruzi_trypomastigota				
Molecule	Predicted Outcome	Probability	Probability Active	Probability Inactive	Predicted Reliability
O=C1NC(C2=CC=CO2)C2=C(CCCS2(=O)=O)N1	Inactive	1.0	0.0	1.0	reliable

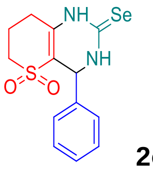

2o O=S1(=O)CCCC2=C1C(NC(=[Se])N2)C1=CC=CC=C1

Molecule 1

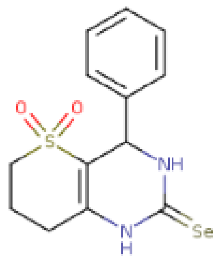

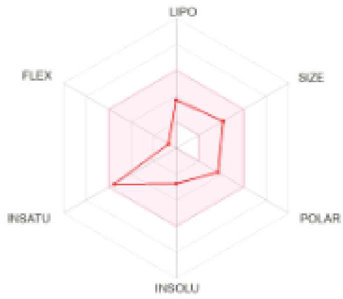

SMILES [Se]=C1NC2=C(C(N1)c1ccccc1)S(=O)(=O)CCC2

Physicochemical Properties	
Formula	C13H14N2O2SSe
Molecular weight	341.29 g/mol
Num. heavy atoms	19
Num. arom. heavy atoms	6
Fraction Csp3	0.31
Num. rotatable bonds	1
Num. H-bond acceptors	2
Num. H-bond donors	2
Molar Refractivity	83.90
TPSA	66.58 Å²

Lipophilicity	
Log $P_{o/w}$ (iLOGP)	0.00
Log $P_{o/w}$ (XLOGP3)	0.96
Log $P_{o/w}$ (WLOGP)	0.59
Log $P_{o/w}$ (MLOGP)	1.16
Log $P_{o/w}$ (SILICOS-IT)	0.30
Consensus Log $P_{o/w}$	0.60

Water Solubility	
Log *S* (ESOL)	-2.73
Solubility	6.38e-01 mg/ml ; 1.87e-03 mol/l
Class	Soluble
Log *S* (Ali)	-1.95
Solubility	3.87e+00 mg/ml ; 1.13e-02 mol/l
Class	Very soluble
Log *S* (SILICOS-IT)	-4.33
Solubility	1.60e-02 mg/ml ; 4.70e-05 mol/l
Class	Moderately soluble

Pharmacokinetics	
GI absorption	High
BBB permeant	No
P-gp substrate	Yes
CYP1A2 inhibitor	No
CYP2C19 inhibitor	No
CYP2C9 inhibitor	No
CYP2D6 inhibitor	No
CYP3A4 inhibitor	No
Log K_p (skin permeation)	-7.70 cm/s

Druglikeness	
Lipinski	Yes; 0 violation
Ghose	Yes
Veber	Yes
Egan	Yes
Muegge	Yes
Bioavailability Score	0.55

Medicinal Chemistry	
PAINS	0 alert
Brenk	1 alert: heavy_metal
Leadlikeness	Yes
Synthetic accessibility	4.04

Oral toxicity prediction results for input compound

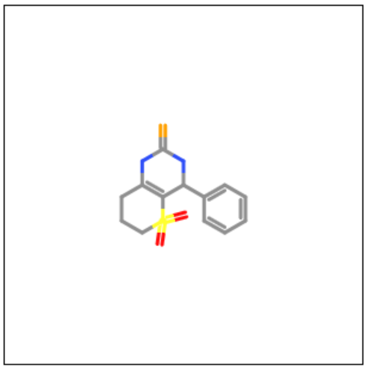

Predicted LD50: 1000mg/kg

Predicted Toxicity Class: 4

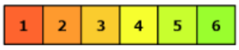

Average similarity: 32.68%

Prediction accuracy: 23%

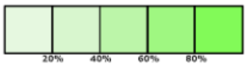

Print Toxicity Report

Name	
Molweight	341.29
Number of hydrogen bond acceptors	5
Number of hydrogen bond donors	2
Number of atoms	19
Number of bonds	21
Number of rotatable bonds	1
Molecular refractivity	83.9
Topological Polar Surface Area	66.58
octanol/water partition coefficient(logP)	2.34

1	Dengue larvicida				
Molecule	Predicted Outcome	Probability	Probability Active	Probability Inactive	Predicted Reliability
O=S1(=O)CCCC2=C1C(NC(=[Se])N2)C1=CC=CC=C1	Active	0.8	0.8	0.2	reliable

2	Sars-Cov				
Molecule	Predicted Outcome	Probability	Probability Active	Probability Inactive	Predicted Reliability
O=S1(=O)CCCC2=C1C(NC(=[Se])N2)C1=CC=CC=C1	Inactive	0.6	0.4	0.6	reliable

3	C_albicans				
Molecule	Predicted Outcome	Probability	Probability Active	Probability Inactive	Predicted Reliability
O=S1(=O)CCCC2=C1C(NC(=[Se])N2)C1=CC=CC=C1	Active	1.0	1.0	0.0	reliable

4	Acetylcholinesterase				
Molecule	Predicted Outcome	Probability	Probability Active	Probability Inactive	Predicted Reliability
O=S1(=O)CCCC2=C1C(NC(=[Se])N2)C1=CC=CC=C1	Inactive	1.0	0.0	1.0	reliable

5	Salmonella				
Molecule	Predicted Outcome	Probability	Probability Active	Probability Inactive	Predicted Reliability
O=S1(=O)CCCC2=C1C(NC(=[Se])N2)C1=CC=CC=C1	Inactive	1.0	0.0	1.0	reliable

6	E_coli				
Molecule	Predicted Outcome	Probability	Probability Active	Probability Inactive	Predicted Reliability
O=S1(=O)CCCC2=C1C(NC(=[Se])N2)C1=CC=CC=C1	Inactive	1.0	0.0	1.0	reliable

7	Hepatite C - Type1				
Molecule	Predicted Outcome	Probability	Probability Active	Probability Inactive	Predicted Reliability
O=S1(=O)CCCC2=C1C(NC(=[Se])N2)C1=CC=CC=C1	Inactive	0.8	0.2	0.8	reliable

8	Hepatite C - NS3-protease helicase				
Molecule	Predicted Outcome	Probability	Probability Active	Probability Inactive	Predicted Reliability
O=S1(=O)CCCC2=C1C(NC(=[Se])N2)C1=CC=CC=C1	Inactive	1.0	0.0	1.0	reliable

9	Hepatite C - RNA dependent				
Molecule	Predicted Outcome	Probability	Probability Active	Probability Inactive	Predicted Reliability
O=S1(=O)CCCC2=C1C(NC(=[Se])N2)C1=CC=CC=C1	Inactive	1.0	0.0	1.0	reliable

10	Hepatite C - Serine protease				
Molecule	Predicted Outcome	Probability	Probability Active	Probability Inactive	Predicted Reliability
O=S1(=O)CCCC2=C1C(NC(=[Se])N2)C1=CC=CC=C1	Inactive	0.8	0.2	0.8	unreliable

11	Leishmania amazonensis - Promastigota				
Molecule	Predicted Outcome	Probability	Probability Active	Probability Inactive	Predicted Reliability
O=S1(=O)CCCC2=C1C(NC(=[Se])N2)C1=CC=CC=C1	Inactive	0.8	0.2	0.8	reliable

12	Leishmania infantum - Promastigota				
Molecule	Predicted Outcome	Probability	Probability Active	Probability Inactive	Predicted Reliability
O=S1(=O)CCCC2=C1C(NC(=[Se])N2)C1=CC=CC=C1	Inactive	0.8	0.2	0.8	reliable

13	Leishmania braziliensis				
Molecule	Predicted Outcome	Probability	Probability Active	Probability Inactive	Predicted Reliability
O=S1(=O)CCCC2=C1C(NC(=[Se])N2)C1=CC=CC=C1	Active	0.8	0.8	0.2	reliable

14	Drosophila melanogaster				
Molecule	Predicted Outcome	Probability	Probability Active	Probability Inactive	Predicted Reliability
O=S1(=O)CCCC2=C1C(NC(=[Se])N2)C1=CC=CC=C1	Inactive	1.0	0.0	1.0	reliable

15	Leishmania major				
Molecule	Predicted Outcome	Probability	Probability Active	Probability Inactive	Predicted Reliability
O=S1(=O)CCCC2=C1C(NC(=[Se])N2)C1=CC=CC=C1	Inactive	1.0	0.0	1.0	reliable

16	Alphis gossypii				
Molecule	Predicted Outcome	Probability	Probability Active	Probability Inactive	Predicted Reliability
O=S1(=O)CCCC2=C1C(NC(=[Se])N2)C1=CC=CC=C1	Active	1.0	1.0	0.0	reliable

17	Alzheimer - iNOS				
Molecule	Predicted Outcome	Probability	Probability Active	Probability Inactive	Predicted Reliability
O=S1(=O)CCCC2=C1C(NC(=[Se])N2)C1=CC=CC=C1	Active	0.6	0.6	0.4	reliable

18	Alzheimer - COX2				
Molecule	Predicted Outcome	Probability	Probability Active	Probability Inactive	Predicted Reliability
O=S1(=O)CCCC2=C1C(NC(=[Se])N2)C1=CC=CC=C1	Inactive	1.0	0.0	1.0	reliable

19	Alzheimer - JNK-3				
Molecule	Predicted Outcome	Probability	Probability Active	Probability Inactive	Predicted Reliability
O=S1(=O)CCCC2=C1C(NC(=[Se])N2)C1=CC=CC=C1	Inactive	1.0	0.0	1.0	reliable

20	Alzheimer - NADPH				
Molecule	Predicted Outcome	Probability	Probability Active	Probability Inactive	Predicted Reliability
O=S1(=O)CCCC2=C1C(NC(=[Se])N2)C1=CC=CC=C1	Inactive	0.8	0.2	0.8	reliable

21	Alzheimer - PDE5				
Molecule	Predicted Outcome	Probability	Probability Active	Probability Inactive	Predicted Reliability
O=S1(=O)CCCC2=C1C(NC(=[Se])N2)C1=CC=CC=C1	Inactive	0.8	0.2	0.8	reliable

22	Amastigote Ldonovani				
Molecule	Predicted Outcome	Probability	Probability Active	Probability Inactive	Predicted Reliability
O=S1(=O)CCCC2=C1C(NC(=[Se])N2)C1=CC=CC=C1	Inactive	1.0	0.0	1.0	reliable

23	Amastigote Chagas				
Molecule	Predicted Outcome	Probability	Probability Active	Probability Inactive	Predicted Reliability
O=S1(=O)CCCC2=C1C(NC(=[Se])N2)C1=CC=CC=C1	Inactive	1.0	0.0	1.0	reliable

24	Epimastigote Chagas				
Molecule	Predicted Outcome	Probability	Probability Active	Probability Inactive	Predicted Reliability
O=S1(=O)CCCC2=C1C(NC(=[Se])N2)C1=CC=CC=C1	Inactive	1.0	0.0	1.0	reliable

25	Promastigote Ldonovani				
Molecule	Predicted Outcome	Probability	Probability Active	Probability Inactive	Predicted Reliability
O=S1(=O)CCCC2=C1C(NC(=[Se])N2)C1=CC=CC=C1	Inactive	0.8	0.2	0.8	reliable

26	PTR L major				
Molecule	Predicted Outcome	Probability	Probability Active	Probability Inactive	Predicted Reliability
O=S1(=O)CCCC2=C1C(NC(=[Se])N2)C1=CC=CC=C1	Inactive	1.0	0.0	1.0	reliable

27	Lamazonensis_promastigota				
Molecule	Predicted Outcome	Probability	Probability Active	Probability Inactive	Predicted Reliability
O=S1(=O)CCCC2=C1C(NC(=[Se])N2)C1=CC=CC=C1	Inactive	1.0	0.0	1.0	reliable

28	Lamazonensis_amastigota				
Molecule	Predicted Outcome	Probability	Probability Active	Probability Inactive	Predicted Reliability
O=S1(=O)CCCC2=C1C(NC(=[Se])N2)C1=CC=CC=C1	Inactive	1.0	0.0	1.0	reliable

29	Tripomastigote Chagas				
Molecule	Predicted Outcome	Probability	Probability Active	Probability Inactive	Predicted Reliability
O=S1(=O)CCCC2=C1C(NC(=[Se])N2)C1=CC=CC=C1	Active	1.0	1.0	0.0	reliable

30	Tcruzi_amastigota				
Molecule	Predicted Outcome	Probability	Probability Active	Probability Inactive	Predicted Reliability
O=S1(=O)CCCC2=C1C(NC(=[Se])N2)C1=CC=CC=C1	Inactive	0.8	0.2	0.8	reliable

31	Tcruzi_epimastigota				
Molecule	Predicted Outcome	Probability	Probability Active	Probability Inactive	Predicted Reliability
O=S1(=O)CCCC2=C1C(NC(=[Se])N2)C1=CC=CC=C1	Active	0.8	0.8	0.2	reliable

32	Tcruzi_trypomastigota				
Molecule	Predicted Outcome	Probability	Probability Active	Probability Inactive	Predicted Reliability
O=S1(=O)CCCC2=C1C(NC(=[Se])N2)C1=CC=CC=C1	Inactive	1.0	0.0	1.0	reliable

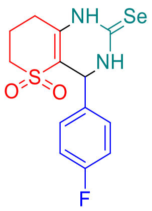

2p FC1=CC=C(C=C1)C1NC(=[Se])NC2=C1S(=O)(=O)CCC2

Molecule 1

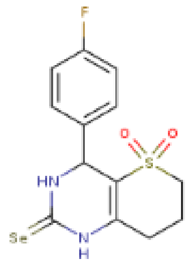

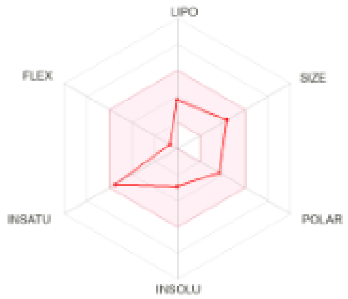

SMILES [Se]=C1NC2=C(C(N1)c1ccc(cc1)F)S(=O)(=O)CCC2

Physicochemical Properties	
Formula	C13H13FN2O2SSe
Molecular weight	359.28 g/mol
Num. heavy atoms	20
Num. arom. heavy atoms	6
Fraction Csp3	0.31
Num. rotatable bonds	1
Num. H-bond acceptors	3
Num. H-bond donors	2
Molar Refractivity	83.86
TPSA	66.58 Å²

Lipophilicity	
Log $P_{o/w}$ (iLOGP)	0.00
Log $P_{o/w}$ (XLOGP3)	1.06
Log $P_{o/w}$ (WLOGP)	1.15
Log $P_{o/w}$ (MLOGP)	1.57
Log $P_{o/w}$ (SILICOS-IT)	0.71
Consensus Log $P_{o/w}$	0.90

Water Solubility	
Log *S* (ESOL)	-2.89
Solubility	4.61e-01 mg/ml ; 1.28e-03 mol/l
Class	Soluble
Log *S* (Ali)	-2.05
Solubility	3.21e+00 mg/ml ; 8.93e-03 mol/l
Class	Soluble
Log *S* (SILICOS-IT)	-4.59
Solubility	9.13e-03 mg/ml ; 2.54e-05 mol/l
Class	Moderately soluble

Pharmacokinetics	
GI absorption	High
BBB permeant	Yes
P-gp substrate	Yes
CYP1A2 inhibitor	No
CYP2C19 inhibitor	No
CYP2C9 inhibitor	No
CYP2D6 inhibitor	No
CYP3A4 inhibitor	No
Log K_p (skin permeation)	-7.74 cm/s

Druglikeness	
Lipinski	Yes; 0 violation
Ghose	Yes
Veber	Yes
Egan	Yes
Muegge	Yes
Bioavailability Score	0.55

Medicinal Chemistry	
PAINS	0 alert
Brenk	1 alert: heavy_metal
Leadlikeness	No; 1 violation: MW>350
Synthetic accessibility	4.02

Oral toxicity prediction results for input compound

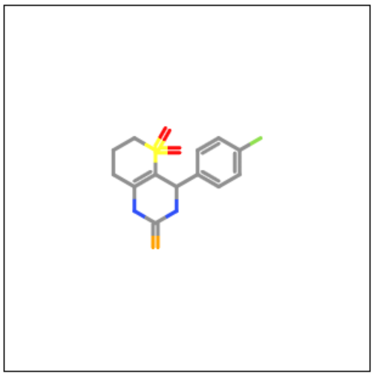

Predicted LD50: 1000mg/kg

Predicted Toxicity Class: 4

1	2	3	4	5	6

Average similarity: 31.66%

Prediction accuracy: 23%

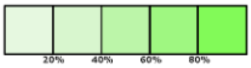

Print Toxicity Report

Name	
Molweight	359.28
Number of hydrogen bond acceptors	5
Number of hydrogen bond donors	2
Number of atoms	20
Number of bonds	22
Number of rotatable bonds	1
Molecular refractivity	83.86
Topological Polar Surface Area	66.58
octanol/water partition coefficient(logP)	2.47

1	Dengue larvicida				
Molecule	Predicted Outcome	Probability	Probability Active	Probability Inactive	Predicted Reliability
FC1=CC=C(C=C1)C1NC(=[Se])NC2=C1S(=O)(=O)CCC2	Active	0.6	0.6	0.4	reliable

2	Sars-Cov				
Molecule	Predicted Outcome	Probability	Probability Active	Probability Inactive	Predicted Reliability
FC1=CC=C(C=C1)C1NC(=[Se])NC2=C1S(=O)(=O)CCC2	Inactive	1.0	0.0	1.0	reliable

3	Acetylcholinesterase				
Molecule	Predicted Outcome	Probability	Probability Active	Probability Inactive	Predicted Reliability
FC1=CC=C(C=C1)C1NC(=[Se])NC2=C1S(=O)(=O)CCC2	Inactive	1.0	0.0	1.0	reliable

4	C_albicans				
Molecule	Predicted Outcome	Probability	Probability Active	Probability Inactive	Predicted Reliability
FC1=CC=C(C=C1)C1NC(=[Se])NC2=C1S(=O)(=O)CCC2	Active	1.0	1.0	0.0	reliable

5	Salmonella				
Molecule	Predicted Outcome	Probability	Probability Active	Probability Inactive	Predicted Reliability
FC1=CC=C(C=C1)C1NC(=[Se])NC2=C1S(=O)(=O)CCC2	Active	0.6	0.6	0.4	reliable

6	Hepatite C - Type1				
Molecule	Predicted Outcome	Probability	Probability Active	Probability Inactive	Predicted Reliability
FC1=CC=C(C=C1)C1NC(=[Se])NC2=C1S(=O)(=O)CCC2	Inactive	0.8	0.2	0.8	reliable

7	E_coli				
Molecule	Predicted Outcome	Probability	Probability Active	Probability Inactive	Predicted Reliability
FC1=CC=C(C=C1)C1NC(=[Se])NC2=C1S(=O)(=O)CCC2	Inactive	1.0	0.0	1.0	reliable

8	Hepatite C - NS3-protease helicase				
Molecule	Predicted Outcome	Probability	Probability Active	Probability Inactive	Predicted Reliability
FC1=CC=C(C=C1)C1NC(=[Se])NC2=C1S(=O)(=O)CCC2	Inactive	1.0	0.0	1.0	reliable

9	Hepatite C - RNA dependent				
Molecule	Predicted Outcome	Probability	Probability Active	Probability Inactive	Predicted Reliability
FC1=CC=C(C=C1)C1NC(=[Se])NC2=C1S(=O)(=O)CCC2	Inactive	1.0	0.0	1.0	reliable

10	Hepatite C - Serine protease				
Molecule	Predicted Outcome	Probability	Probability Active	Probability Inactive	Predicted Reliability
FC1=CC=C(C=C1)C1NC(=[Se])NC2=C1S(=O)(=O)CCC2	Inactive	1.0	0.0	1.0	unreliable

11	Leishmania infantum - Promastigota				
Molecule	Predicted Outcome	Probability	Probability Active	Probability Inactive	Predicted Reliability
FC1=CC=C(C=C1)C1NC(=[Se])NC2=C1S(=O)(=O)CCC2	Inactive	0.8	0.2	0.8	reliable

12	Leishmania amazonensis - Promastigota				
Molecule	Predicted Outcome	Probability	Probability Active	Probability Inactive	Predicted Reliability
FC1=CC=C(C=C1)C1NC(=[Se])NC2=C1S(=O)(=O)CCC2	Inactive	0.6	0.4	0.6	reliable

13	Leishmania braziliensis				
Molecule	Predicted Outcome	Probability	Probability Active	Probability Inactive	Predicted Reliability
FC1=CC=C(C=C1)C1NC(=[Se])NC2=C1S(=O)(=O)CCC2	Active	0.8	0.8	0.2	reliable

14	Drosophila melanogaster				
Molecule	Predicted Outcome	Probability	Probability Active	Probability Inactive	Predicted Reliability
FC1=CC=C(C=C1)C1NC(=[Se])NC2=C1S(=O)(=O)CCC2	Inactive	1.0	0.0	1.0	reliable

15	Leishmania major				
Molecule	Predicted Outcome	Probability	Probability Active	Probability Inactive	Predicted Reliability
FC1=CC=C(C=C1)C1NC(=[Se])NC2=C1S(=O)(=O)CCC2	Inactive	1.0	0.0	1.0	reliable

16	Alphis gossypii				
Molecule	Predicted Outcome	Probability	Probability Active	Probability Inactive	Predicted Reliability
FC1=CC=C(C=C1)C1NC(=[Se])NC2=C1S(=O)(=O)CCC2	Active	1.0	1.0	0.0	reliable

17	Alzheimer - iNOS				
Molecule	Predicted Outcome	Probability	Probability Active	Probability Inactive	Predicted Reliability
FC1=CC=C(C=C1)C1NC(=[Se])NC2=C1S(=O)(=O)CCC2	Inactive	0.8	0.2	0.8	reliable

18	Alzheimer - JNK-3				
Molecule	Predicted Outcome	Probability	Probability Active	Probability Inactive	Predicted Reliability
FC1=CC=C(C=C1)C1NC(=[Se])NC2=C1S(=O)(=O)CCC2	Inactive	1.0	0.0	1.0	reliable

19	Alzheimer - COX2				
Molecule	Predicted Outcome	Probability	Probability Active	Probability Inactive	Predicted Reliability
FC1=CC=C(C=C1)C1NC(=[Se])NC2=C1S(=O)(=O)CCC2	Inactive	1.0	0.0	1.0	reliable

20	Alzheimer - NADPH				
Molecule	Predicted Outcome	Probability	Probability Active	Probability Inactive	Predicted Reliability
FC1=CC=C(C=C1)C1NC(=[Se])NC2=C1S(=O)(=O)CCC2	Inactive	0.6	0.4	0.6	reliable

21	Amastigote Chagas				
Molecule	Predicted Outcome	Probability	Probability Active	Probability Inactive	Predicted Reliability
FC1=CC=C(C=C1)C1NC(=[Se])NC2=C1S(=O)(=O)CCC2	Inactive	1.0	0.0	1.0	reliable

22	Alzheimer - PDE5				
Molecule	Predicted Outcome	Probability	Probability Active	Probability Inactive	Predicted Reliability
FC1=CC=C(C=C1)C1NC(=[Se])NC2=C1S(=O)(=O)CCC2	Inactive	0.8	0.2	0.8	reliable

23	Amastigote Ldonovani				
Molecule	Predicted Outcome	Probability	Probability Active	Probability Inactive	Predicted Reliability
FC1=CC=C(C=C1)C1NC(=[Se])NC2=C1S(=O)(=O)CCC2	Inactive	1.0	0.0	1.0	reliable

24	Epimastigote Chagas				
Molecule	Predicted Outcome	Probability	Probability Active	Probability Inactive	Predicted Reliability
FC1=CC=C(C=C1)C1NC(=[Se])NC2=C1S(=O)(=O)CCC2	Inactive	1.0	0.0	1.0	reliable

25	Promastigote Ldonovani				
Molecule	Predicted Outcome	Probability	Probability Active	Probability Inactive	Predicted Reliability
FC1=CC=C(C=C1)C1NC(=[Se])NC2=C1S(=O)(=O)CCC2	Active	0.6	0.6	0.4	reliable

26	PTR L major				
Molecule	Predicted Outcome	Probability	Probability Active	Probability Inactive	Predicted Reliability
FC1=CC=C(C=C1)C1NC(=[Se])NC2=C1S(=O)(=O)CCC2	Inactive	1.0	0.0	1.0	reliable

27	Tripomastigote Chagas				
Molecule	Predicted Outcome	Probability	Probability Active	Probability Inactive	Predicted Reliability
FC1=CC=C(C=C1)C1NC(=[Se])NC2=C1S(=O)(=O)CCC2	Active	1.0	1.0	0.0	reliable

28	Lamazonensis_amastigota				
Molecule	Predicted Outcome	Probability	Probability Active	Probability Inactive	Predicted Reliability
FC1=CC=C(C=C1)C1NC(=[Se])NC2=C1S(=O)(=O)CCC2	Inactive	1.0	0.0	1.0	reliable

29	Lamazonensis_promastigota				
Molecule	Predicted Outcome	Probability	Probability Active	Probability Inactive	Predicted Reliability
FC1=CC=C(C=C1)C1NC(=[Se])NC2=C1S(=O)(=O)CCC2	Inactive	1.0	0.0	1.0	reliable

30	Tcruzi_amastigota				
Molecule	Predicted Outcome	Probability	Probability Active	Probability Inactive	Predicted Reliability
FC1=CC=C(C=C1)C1NC(=[Se])NC2=C1S(=O)(=O)CCC2	Inactive	0.6	0.4	0.6	reliable

31	Tcruzi_epimastigota				
Molecule	Predicted Outcome	Probability	Probability Active	Probability Inactive	Predicted Reliability
FC1=CC=C(C=C1)C1NC(=[Se])NC2=C1S(=O)(=O)CCC2	Inactive	0.6	0.4	0.6	reliable

32	Tcruzi_trypomastigota				
Molecule	Predicted Outcome	Probability	Probability Active	Probability Inactive	Predicted Reliability
FC1=CC=C(C=C1)C1NC(=[Se])NC2=C1S(=O)(=O)CCC2	Inactive	0.6	0.4	0.6	reliable

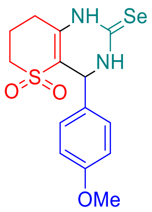

2q COC1=CC=C(C=C1)C1NC(=[Se])NC2=C1S(=O)(=O)CCC2

Molecule 1

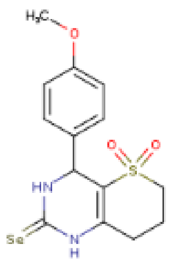

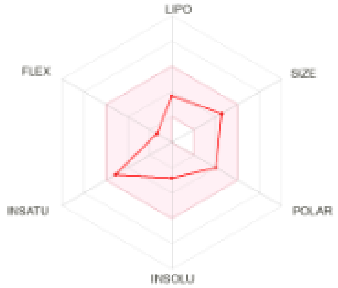

SMILES COc1ccc(cc1)C1NC(=[Se])NC2=C1S(=O)(=O)CCC2

Physicochemical Properties	
Formula	C14H16N2O3SSe
Molecular weight	371.31 g/mol
Num. heavy atoms	21
Num. arom. heavy atoms	6
Fraction Csp3	0.36
Num. rotatable bonds	2
Num. H-bond acceptors	3
Num. H-bond donors	2
Molar Refractivity	90.39
TPSA	75.81 Å²

Lipophilicity	
Log $P_{o/w}$ (iLOGP)	0.00
Log $P_{o/w}$ (XLOGP3)	0.93
Log $P_{o/w}$ (WLOGP)	0.60
Log $P_{o/w}$ (MLOGP)	0.87
Log $P_{o/w}$ (SILICOS-IT)	0.32
Consensus Log $P_{o/w}$	0.54

Water Solubility	
Log *S* (ESOL)	-2.81
Solubility	5.78e-01 mg/ml ; 1.56e-03 mol/l
Class	Soluble
Log *S* (Ali)	-2.11
Solubility	2.89e+00 mg/ml ; 7.79e-03 mol/l
Class	Soluble
Log *S* (SILICOS-IT)	-4.43
Solubility	1.37e-02 mg/ml ; 3.68e-05 mol/l
Class	Moderately soluble

Pharmacokinetics	
GI absorption	High
BBB permeant	No
P-gp substrate	Yes
CYP1A2 inhibitor	No
CYP2C19 inhibitor	No
CYP2C9 inhibitor	No
CYP2D6 inhibitor	No
CYP3A4 inhibitor	No
Log K_p (skin permeation)	-7.90 cm/s

Druglikeness	
Lipinski	Yes; 0 violation
Ghose	Yes
Veber	Yes
Egan	Yes
Muegge	Yes
Bioavailability Score	0.55

Medicinal Chemistry	
PAINS	0 alert
Brenk	1 alert: heavy_metal
Leadlikeness	No; 1 violation: MW>350
Synthetic accessibility	4.06

Oral toxicity prediction results for input compound

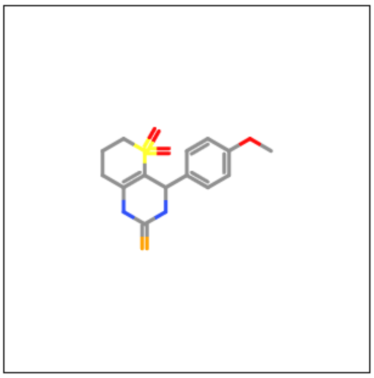

Predicted LD50: 581mg/kg

Predicted Toxicity Class: 4

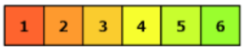

Average similarity: 32.14%

Prediction accuracy: 23%

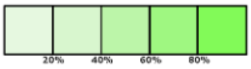

Print Toxicity Report

Name	
Molweight	371.31
Number of hydrogen bond acceptors	6
Number of hydrogen bond donors	2
Number of atoms	21
Number of bonds	23
Number of rotatable bonds	2
Molecular refractivity	90.39
Topological Polar Surface Area	75.81
octanol/water partition coefficient(logP)	2.34

1	Dengue larvicida				
Molecule	Predicted Outcome	Probability	Probability Active	Probability Inactive	Predicted Reliability
COC1=CC=C(C=C1)C1NC(=[Se])NC2=C1S(=O)(=O)CCC2	Active	0.8	0.8	0.2	reliable

2	Sars-Cov				
Molecule	Predicted Outcome	Probability	Probability Active	Probability Inactive	Predicted Reliability
COC1=CC=C(C=C1)C1NC(=[Se])NC2=C1S(=O)(=O)CCC2	Inactive	0.8	0.2	0.8	reliable

3	Acetylcholinesterase				
Molecule	Predicted Outcome	Probability	Probability Active	Probability Inactive	Predicted Reliability
COC1=CC=C(C=C1)C1NC(=[Se])NC2=C1S(=O)(=O)CCC2	Inactive	1.0	0.0	1.0	reliable

4	Salmonella				
Molecule	Predicted Outcome	Probability	Probability Active	Probability Inactive	Predicted Reliability
COC1=CC=C(C=C1)C1NC(=[Se])NC2=C1S(=O)(=O)CCC2	Inactive	1.0	0.0	1.0	reliable

5	C_albicans				
Molecule	Predicted Outcome	Probability	Probability Active	Probability Inactive	Predicted Reliability
COC1=CC=C(C=C1)C1NC(=[Se])NC2=C1S(=O)(=O)CCC2	Active	1.0	1.0	0.0	reliable

6	Hepatite C - Type1				
Molecule	Predicted Outcome	Probability	Probability Active	Probability Inactive	Predicted Reliability
COC1=CC=C(C=C1)C1NC(=[Se])NC2=C1S(=O)(=O)CCC2	Inactive	0.8	0.2	0.8	unreliable

7	E_coli				
Molecule	Predicted Outcome	Probability	Probability Active	Probability Inactive	Predicted Reliability
COC1=CC=C(C=C1)C1NC(=[Se])NC2=C1S(=O)(=O)CCC2	Inactive	0.8	0.2	0.8	reliable

8	Hepatite C - NS3-protease helicase				
Molecule	Predicted Outcome	Probability	Probability Active	Probability Inactive	Predicted Reliability
COC1=CC=C(C=C1)C1NC(=[Se])NC2=C1S(=O)(=O)CCC2	Inactive	1.0	0.0	1.0	reliable

9	Hepatite C - Serine protease				
Molecule	Predicted Outcome	Probability	Probability Active	Probability Inactive	Predicted Reliability
COC1=CC=C(C=C1)C1NC(=[Se])NC2=C1S(=O)(=O)CCC2	Inactive	1.0	0.0	1.0	unreliable

10	Hepatite C - RNA dependent				
Molecule	Predicted Outcome	Probability	Probability Active	Probability Inactive	Predicted Reliability
COC1=CC=C(C=C1)C1NC(=[Se])NC2=C1S(=O)(=O)CCC2	Inactive	1.0	0.0	1.0	reliable

11	Leishmania amazonensis - Promastigota				
Molecule	Predicted Outcome	Probability	Probability Active	Probability Inactive	Predicted Reliability
COC1=CC=C(C=C1)C1NC(=[Se])NC2=C1S(=O)(=O)CCC2	Inactive	0.6	0.4	0.6	reliable

12	Leishmania infantum - Promastigota				
Molecule	Predicted Outcome	Probability	Probability Active	Probability Inactive	Predicted Reliability
COC1=CC=C(C=C1)C1NC(=[Se])NC2=C1S(=O)(=O)CCC2	Inactive	0.8	0.2	0.8	reliable

13	Leishmania braziliensis				
Molecule	Predicted Outcome	Probability	Probability Active	Probability Inactive	Predicted Reliability
COC1=CC=C(C=C1)C1NC(=[Se])NC2=C1S(=O)(=O)CCC2	Active	0.8	0.8	0.2	reliable

14	Leishmania major				
Molecule	Predicted Outcome	Probability	Probability Active	Probability Inactive	Predicted Reliability
COC1=CC=C(C=C1)C1NC(=[Se])NC2=C1S(=O)(=O)CCC2	Inactive	1.0	0.0	1.0	reliable

15	Drosophila melanogaster				
Molecule	Predicted Outcome	Probability	Probability Active	Probability Inactive	Predicted Reliability
COC1=CC=C(C=C1)C1NC(=[Se])NC2=C1S(=O)(=O)CCC2	Inactive	1.0	0.0	1.0	unreliable

16	Alphis gossypii				
Molecule	Predicted Outcome	Probability	Probability Active	Probability Inactive	Predicted Reliability
COC1=CC=C(C=C1)C1NC(=[Se])NC2=C1S(=O)(=O)CCC2	Active	1.0	1.0	0.0	reliable

17	Alzheimer - COX2				
Molecule	Predicted Outcome	Probability	Probability Active	Probability Inactive	Predicted Reliability
COC1=CC=C(C=C1)C1NC(=[Se])NC2=C1S(=O)(=O)CCC2	Inactive	1.0	0.0	1.0	reliable

18	Alzheimer - NADPH				
Molecule	Predicted Outcome	Probability	Probability Active	Probability Inactive	Predicted Reliability
COC1=CC=C(C=C1)C1NC(=[Se])NC2=C1S(=O)(=O)CCC2	Inactive	0.6	0.4	0.6	reliable

19	Alzheimer - iNOS				
Molecule	Predicted Outcome	Probability	Probability Active	Probability Inactive	Predicted Reliability
COC1=CC=C(C=C1)C1NC(=[Se])NC2=C1S(=O)(=O)CCC2	Inactive	0.8	0.2	0.8	reliable

20	Alzheimer - JNK-3				
Molecule	Predicted Outcome	Probability	Probability Active	Probability Inactive	Predicted Reliability
COC1=CC=C(C=C1)C1NC(=[Se])NC2=C1S(=O)(=O)CCC2	Inactive	1.0	0.0	1.0	reliable

21	Alzheimer - PDE5				
Molecule	Predicted Outcome	Probability	Probability Active	Probability Inactive	Predicted Reliability
COC1=CC=C(C=C1)C1NC(=[Se])NC2=C1S(=O)(=O)CCC2	Inactive	0.8	0.2	0.8	reliable

22	Amastigote Chagas				
Molecule	Predicted Outcome	Probability	Probability Active	Probability Inactive	Predicted Reliability
COC1=CC=C(C=C1)C1NC(=[Se])NC2=C1S(=O)(=O)CCC2	Inactive	1.0	0.0	1.0	reliable

23	Amastigote Ldonovani				
Molecule	Predicted Outcome	Probability	Probability Active	Probability Inactive	Predicted Reliability
COC1=CC=C(C=C1)C1NC(=[Se])NC2=C1S(=O)(=O)CCC2	Inactive	1.0	0.0	1.0	reliable

24	Epimastigote Chagas				
Molecule	Predicted Outcome	Probability	Probability Active	Probability Inactive	Predicted Reliability
COC1=CC=C(C=C1)C1NC(=[Se])NC2=C1S(=O)(=O)CCC2	Inactive	1.0	0.0	1.0	reliable

25	Promastigote Ldonovani				
Molecule	Predicted Outcome	Probability	Probability Active	Probability Inactive	Predicted Reliability
COC1=CC=C(C=C1)C1NC(=[Se])NC2=C1S(=O)(=O)CCC2	Inactive	1.0	0.0	1.0	reliable

26	Lamazonensis_amastigota				
Molecule	Predicted Outcome	Probability	Probability Active	Probability Inactive	Predicted Reliability
COC1=CC=C(C=C1)C1NC(=[Se])NC2=C1S(=O)(=O)CCC2	Inactive	1.0	0.0	1.0	reliable

27	PTR L major				
Molecule	Predicted Outcome	Probability	Probability Active	Probability Inactive	Predicted Reliability
COC1=CC=C(C=C1)C1NC(=[Se])NC2=C1S(=O)(=O)CCC2	Inactive	1.0	0.0	1.0	reliable

28	Tripomastigote Chagas				
Molecule	Predicted Outcome	Probability	Probability Active	Probability Inactive	Predicted Reliability
COC1=CC=C(C=C1)C1NC(=[Se])NC2=C1S(=O)(=O)CCC2	Active	1.0	1.0	0.0	reliable

29	Lamazonensis_promastigota				
**Molecule**	**Predicted Outcome**	**Probability**	**Probability Active**	**Probability Inactive**	**Predicted Reliability**
COC1=CC=C(C=C1)C1NC(=[Se])NC2=C1S(=O)(=O)CCC2	Inactive	1.0	0.0	1.0	reliable

30	Tcruzi_epimastigota				
**Molecule**	**Predicted Outcome**	**Probability**	**Probability Active**	**Probability Inactive**	**Predicted Reliability**
COC1=CC=C(C=C1)C1NC(=[Se])NC2=C1S(=O)(=O)CCC2	Inactive	0.6	0.4	0.6	reliable

31	Tcruzi_amastigota				
**Molecule**	**Predicted Outcome**	**Probability**	**Probability Active**	**Probability Inactive**	**Predicted Reliability**
COC1=CC=C(C=C1)C1NC(=[Se])NC2=C1S(=O)(=O)CCC2	Inactive	0.6	0.4	0.6	reliable

32	Tcruzi_trypomastigota				
**Molecule**	**Predicted Outcome**	**Probability**	**Probability Active**	**Probability Inactive**	**Predicted Reliability**
COC1=CC=C(C=C1)C1NC(=[Se])NC2=C1S(=O)(=O)CCC2	Inactive	0.8	0.2	0.8	reliable

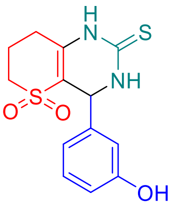

2r OC1=CC(=CC=C1)C1NC(=S)NC2=C1S(=O)(=O)CCC2

Molecule 1

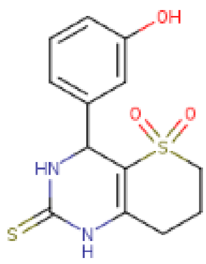

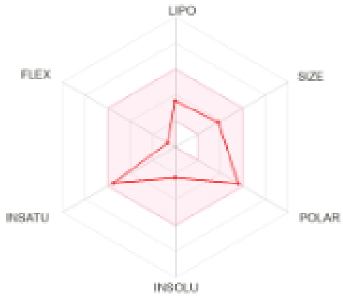

SMILES S=C1NC2=C(C(N1)c1cccc(c1)O)S(=O)(=O)CCC2

Physicochemical Properties	
Formula	C13H14N2O3S2
Molecular weight	310.39 g/mol
Num. heavy atoms	20
Num. arom. heavy atoms	6
Fraction Csp3	0.31
Num. rotatable bonds	1
Num. H-bond acceptors	3
Num. H-bond donors	3
Molar Refractivity	87.76
TPSA	118.90 Å²

Lipophilicity	
Log $P_{o/w}$ (iLOGP)	1.42
Log $P_{o/w}$ (XLOGP3)	0.67
Log $P_{o/w}$ (WLOGP)	1.33
Log $P_{o/w}$ (MLOGP)	0.60
Log $P_{o/w}$ (SILICOS-IT)	1.96
Consensus Log $P_{o/w}$	1.19

Water Solubility	
Log *S* (ESOL)	-2.34
Solubility	1.41e+00 mg/ml ; 4.54e-03 mol/l
Class	Soluble
Log *S* (Ali)	-2.74
Solubility	5.60e-01 mg/ml ; 1.81e-03 mol/l
Class	Soluble
Log *S* (SILICOS-IT)	-3.61
Solubility	7.69e-02 mg/ml ; 2.48e-04 mol/l
Class	Soluble

Pharmacokinetics	
GI absorption	High
BBB permeant	No
P-gp substrate	Yes
CYP1A2 inhibitor	No
CYP2C19 inhibitor	No
CYP2C9 inhibitor	No
CYP2D6 inhibitor	No
CYP3A4 inhibitor	No
Log K_p (skin permeation)	-7.72 cm/s

Druglikeness	
Lipinski	Yes; 0 violation
Ghose	Yes
Veber	Yes
Egan	Yes
Muegge	Yes
Bioavailability Score	0.55

Medicinal Chemistry	
PAINS	0 alert
Brenk	1 alert: thiocarbonyl_group
Leadlikeness	Yes
Synthetic accessibility	3.88

Oral toxicity prediction results for input compound

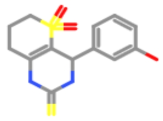

Predicted LD50: 927mg/kg

Predicted Toxicity Class: 4

1	2	3	4	5	6

Average similarity: 33.34%

Prediction accuracy: 23%

20% 40% 60% 80%

Print Toxicity Report

Name	
Molweight	310.39
Number of hydrogen bond acceptors	6
Number of hydrogen bond donors	3
Number of atoms	20
Number of bonds	22
Number of rotable bonds	1
Molecular refractivity	87.76
Topological Polar Surface Area	118.9
octanol/water partition coefficient(logP)	3.07

1	Sars-Cov				
Molecule	Predicted Outcome	Probability	Probability Active	Probability Inactive	Predicted Reliability
OC1=CC(=CC=C1)C1NC(=S)NC2=C1S(=O)(=O)CCC2	Inactive	0.6	0.4	0.6	unreliable

2	Dengue larvicida				
Molecule	Predicted Outcome	Probability	Probability Active	Probability Inactive	Predicted Reliability
OC1=CC(=CC=C1)C1NC(=S)NC2=C1S(=O)(=O)CCC2	Active	0.8	0.8	0.2	reliable

3	Salmonella				
Molecule	Predicted Outcome	Probability	Probability Active	Probability Inactive	Predicted Reliability
OC1=CC(=CC=C1)C1NC(=S)NC2=C1S(=O)(=O)CCC2	Inactive	1.0	0.0	1.0	reliable

4	Acetylcholinesterase				
Molecule	Predicted Outcome	Probability	Probability Active	Probability Inactive	Predicted Reliability
OC1=CC(=CC=C1)C1NC(=S)NC2=C1S(=O)(=O)CCC2	Inactive	1.0	0.0	1.0	reliable

5	E_coli				
Molecule	Predicted Outcome	Probability	Probability Active	Probability Inactive	Predicted Reliability
OC1=CC(=CC=C1)C1NC(=S)NC2=C1S(=O)(=O)CCC2	Inactive	1.0	0.0	1.0	reliable

6	C_albicans				
Molecule	Predicted Outcome	Probability	Probability Active	Probability Inactive	Predicted Reliability
OC1=CC(=CC=C1)C1NC(=S)NC2=C1S(=O)(=O)CCC2	Active	1.0	1.0	0.0	reliable

7	Hepatite C - Type1				
Molecule	Predicted Outcome	Probability	Probability Active	Probability Inactive	Predicted Reliability
OC1=CC(=CC=C1)C1NC(=S)NC2=C1S(=O)(=O)CCC2	Inactive	0.8	0.2	0.8	reliable

8	Hepatite C - NS3-protease helicase				
Molecule	Predicted Outcome	Probability	Probability Active	Probability Inactive	Predicted Reliability
OC1=CC(=CC=C1)C1NC(=S)NC2=C1S(=O)(=O)CCC2	Inactive	1.0	0.0	1.0	reliable

9	Hepatite C - Serine protease				
Molecule	Predicted Outcome	Probability	Probability Active	Probability Inactive	Predicted Reliability
OC1=CC(=CC=C1)C1NC(=S)NC2=C1S(=O)(=O)CCC2	Inactive	1.0	0.0	1.0	unreliable

10	Leishmania amazonensis - Promastigota				
Molecule	Predicted Outcome	Probability	Probability Active	Probability Inactive	Predicted Reliability
OC1=CC(=CC=C1)C1NC(=S)NC2=C1S(=O)(=O)CCC2	Inactive	0.8	0.2	0.8	reliable

11	Hepatite C - RNA dependent				
Molecule	Predicted Outcome	Probability	Probability Active	Probability Inactive	Predicted Reliability
OC1=CC(=CC=C1)C1NC(=S)NC2=C1S(=O)(=O)CCC2	Inactive	1.0	0.0	1.0	reliable

12	Leishmania infantum - Promastigota				
Molecule	Predicted Outcome	Probability	Probability Active	Probability Inactive	Predicted Reliability
OC1=CC(=CC=C1)C1NC(=S)NC2=C1S(=O)(=O)CCC2	Active	1.0	1.0	0.0	reliable

13	Leishmania braziliensis				
Molecule	Predicted Outcome	Probability	Probability Active	Probability Inactive	Predicted Reliability
OC1=CC(=CC=C1)C1NC(=S)NC2=C1S(=O)(=O)CCC2	Active	0.6	0.6	0.4	reliable

14	Drosophila melanogaster				
Molecule	Predicted Outcome	Probability	Probability Active	Probability Inactive	Predicted Reliability
OC1=CC(=CC=C1)C1NC(=S)NC2=C1S(=O)(=O)CCC2	Inactive	1.0	0.0	1.0	unreliable

15	Leishmania major				
Molecule	Predicted Outcome	Probability	Probability Active	Probability Inactive	Predicted Reliability
OC1=CC(=CC=C1)C1NC(=S)NC2=C1S(=O)(=O)CCC2	Inactive	1.0	0.0	1.0	reliable

16	Alphis gossypii				
Molecule	Predicted Outcome	Probability	Probability Active	Probability Inactive	Predicted Reliability
OC1=CC(=CC=C1)C1NC(=S)NC2=C1S(=O)(=O)CCC2	Active	1.0	1.0	0.0	unreliable

17	Alzheimer - COX2				
Molecule	Predicted Outcome	Probability	Probability Active	Probability Inactive	Predicted Reliability
OC1=CC(=CC=C1)C1NC(=S)NC2=C1S(=O)(=O)CCC2	Inactive	1.0	0.0	1.0	reliable

18	Alzheimer - iNOS				
Molecule	Predicted Outcome	Probability	Probability Active	Probability Inactive	Predicted Reliability
OC1=CC(=CC=C1)C1NC(=S)NC2=C1S(=O)(=O)CCC2	Active	0.6	0.6	0.4	reliable

19	Alzheimer - NADPH				
Molecule	Predicted Outcome	Probability	Probability Active	Probability Inactive	Predicted Reliability
OC1=CC(=CC=C1)C1NC(=S)NC2=C1S(=O)(=O)CCC2	Inactive	1.0	0.0	1.0	reliable

20	Alzheimer - JNK-3				
Molecule	Predicted Outcome	Probability	Probability Active	Probability Inactive	Predicted Reliability
OC1=CC(=CC=C1)C1NC(=S)NC2=C1S(=O)(=O)CCC2	Inactive	1.0	0.0	1.0	reliable

21	Epimastigote Chagas				
Molecule	Predicted Outcome	Probability	Probability Active	Probability Inactive	Predicted Reliability
OC1=CC(=CC=C1)C1NC(=S)NC2=C1S(=O)(=O)CCC2	Inactive	1.0	0.0	1.0	reliable

22	Alzheimer - PDE5				
Molecule	Predicted Outcome	Probability	Probability Active	Probability Inactive	Predicted Reliability
OC1=CC(=CC=C1)C1NC(=S)NC2=C1S(=O)(=O)CCC2	Inactive	1.0	0.0	1.0	reliable

23	Amastigote Ldonovani				
Molecule	Predicted Outcome	Probability	Probability Active	Probability Inactive	Predicted Reliability
OC1=CC(=CC=C1)C1NC(=S)NC2=C1S(=O)(=O)CCC2	Inactive	1.0	0.0	1.0	reliable

24	Amastigote Chagas				
Molecule	Predicted Outcome	Probability	Probability Active	Probability Inactive	Predicted Reliability
OC1=CC(=CC=C1)C1NC(=S)NC2=C1S(=O)(=O)CCC2	Inactive	0.6	0.4	0.6	reliable

25	Promastigote Ldonovani				
Molecule	Predicted Outcome	Probability	Probability Active	Probability Inactive	Predicted Reliability
OC1=CC(=CC=C1)C1NC(=S)NC2=C1S(=O)(=O)CCC2	Inactive	0.6	0.4	0.6	reliable

26	PTR L major				
Molecule	Predicted Outcome	Probability	Probability Active	Probability Inactive	Predicted Reliability
OC1=CC(=CC=C1)C1NC(=S)NC2=C1S(=O)(=O)CCC2	Inactive	1.0	0.0	1.0	reliable

27	Tripomastigote Chagas				
Molecule	Predicted Outcome	Probability	Probability Active	Probability Inactive	Predicted Reliability
OC1=CC(=CC=C1)C1NC(=S)NC2=C1S(=O)(=O)CCC2	Inactive	1.0	0.0	1.0	reliable

28	Lamazonensis_amastigota				
Molecule	Predicted Outcome	Probability	Probability Active	Probability Inactive	Predicted Reliability
OC1=CC(=CC=C1)C1NC(=S)NC2=C1S(=O)(=O)CCC2	Inactive	1.0	0.0	1.0	reliable

29	Lamazonensis_promastigota				
**Molecule**	**Predicted Outcome**	**Probability**	**Probability Active**	**Probability Inactive**	**Predicted Reliability**
OC1=CC(=CC=C1)C1NC(=S)NC2=C1S(=O)(=O)CCC2	Inactive	1.0	0.0	1.0	reliable

30	Tcruzi_amastigota				
**Molecule**	**Predicted Outcome**	**Probability**	**Probability Active**	**Probability Inactive**	**Predicted Reliability**
OC1=CC(=CC=C1)C1NC(=S)NC2=C1S(=O)(=O)CCC2	Inactive	0.6	0.4	0.6	reliable

31	Tcruzi_trypomastigota				
**Molecule**	**Predicted Outcome**	**Probability**	**Probability Active**	**Probability Inactive**	**Predicted Reliability**
OC1=CC(=CC=C1)C1NC(=S)NC2=C1S(=O)(=O)CCC2	Inactive	1.0	0.0	1.0	reliable

32	Tcruzi_epimastigota				
**Molecule**	**Predicted Outcome**	**Probability**	**Probability Active**	**Probability Inactive**	**Predicted Reliability**
OC1=CC(=CC=C1)C1NC(=S)NC2=C1S(=O)(=O)CCC2	Active	0.8	0.8	0.2	reliable

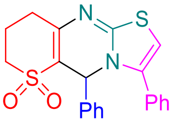

3 O=S1(=O)CCCC2=C1C(N1C(SC=C1C1=CC=CC=C1)=N2)C1=CC=CC=C1

Molecule 1

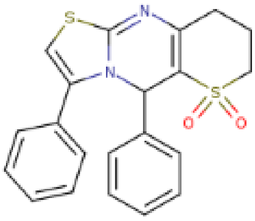

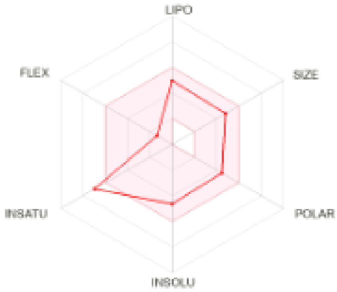

SMILES O=S1(=O)CCCC2=C1C(c1ccccc1)n1c(=N2)scc1c1ccccc1

Physicochemical Properties	
Formula	C21H18N2O2S2
Molecular weight	394.51 g/mol
Num. heavy atoms	27
Num. arom. heavy atoms	17
Fraction Csp3	0.19
Num. rotatable bonds	2
Num. H-bond acceptors	3
Num. H-bond donors	0
Molar Refractivity	111.74
TPSA	88.05 Å²

Lipophilicity	
Log $P_{o/w}$ (iLOGP)	2.90
Log $P_{o/w}$ (XLOGP3)	3.21
Log $P_{o/w}$ (WLOGP)	4.84
Log $P_{o/w}$ (MLOGP)	3.10
Log $P_{o/w}$ (SILICOS-IT)	4.73
Consensus Log $P_{o/w}$	3.76

Water Solubility	
Log *S* (ESOL)	-4.64
Solubility	8.99e-03 mg/ml ; 2.28e-05 mol/l
Class	Moderately soluble
Log *S* (Ali)	-4.73
Solubility	7.32e-03 mg/ml ; 1.86e-05 mol/l
Class	Moderately soluble
Log *S* (SILICOS-IT)	-6.81
Solubility	6.17e-05 mg/ml ; 1.56e-07 mol/l
Class	Poorly soluble

Pharmacokinetics	
GI absorption	High
BBB permeant	No
P-gp substrate	No
CYP1A2 inhibitor	Yes
CYP2C19 inhibitor	Yes
CYP2C9 inhibitor	Yes
CYP2D6 inhibitor	No
CYP3A4 inhibitor	Yes
Log K_p (skin permeation)	-6.43 cm/s

Druglikeness	
Lipinski	Yes; 0 violation
Ghose	Yes
Veber	Yes
Egan	Yes
Muegge	Yes
Bioavailability Score	0.55

Medicinal Chemistry	
PAINS	0 alert
Brenk	0 alert
Leadlikeness	No; 1 violation: MW>350
Synthetic accessibility	4.57

Oral toxicity prediction results for input compound

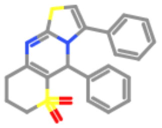

Predicted LD50: 1000mg/kg

Predicted Toxicity Class: 4

1	2	3	4	5	6

Average similarity: 44.18%

Prediction accuracy: 54.26%

20% 40% 60% 80%

Print Toxicity Report

Name	O=S1(=O)CCCC2=C1C(
Molweight	394.51
Number of hydrogen bond acceptors	5
Number of hydrogen bond donors	0
Number of atoms	27
Number of bonds	31
Number of rotable bonds	2
Molecular refractivity	118.23
Topological Polar Surface Area	83.42
octanol/water partition coefficient(logP)	5.02

1	Hepatite C - Serine protease				
**Molecule**	**Predicted Outcome**	**Probability**	**Probability Active**	**Probability Inactive**	**Predicted Reliability**
O=S1(=O)CCCC2=C1C(N1C(SC=C1C1=CC=CC=C1)=N2)C1=CC=CC=C1	Inactive	1.0	0.0	1.0	unreliable

2	Hepatite C - NS3-protease helicase				
**Molecule**	**Predicted Outcome**	**Probability**	**Probability Active**	**Probability Inactive**	**Predicted Reliability**
O=S1(=O)CCCC2=C1C(N1C(SC=C1C1=CC=CC=C1)=N2)C1=CC=CC=C1	Inactive	1.0	0.0	1.0	unreliable

3	Hepatite C - Type1				
**Molecule**	**Predicted Outcome**	**Probability**	**Probability Active**	**Probability Inactive**	**Predicted Reliability**
O=S1(=O)CCCC2=C1C(N1C(SC=C1C1=CC=CC=C1)=N2)C1=CC=CC=C1	Inactive	0.6	0.4	0.6	reliable

4	Hepatite C - RNA dependent				
**Molecule**	**Predicted Outcome**	**Probability**	**Probability Active**	**Probability Inactive**	**Predicted Reliability**
O=S1(=O)CCCC2=C1C(N1C(SC=C1C1=CC=CC=C1)=N2)C1=CC=CC=C1	Inactive	1.0	0.0	1.0	reliable

5	Leishmania amazonensis - Promastigota				
**Molecule**	**Predicted Outcome**	**Probability**	**Probability Active**	**Probability Inactive**	**Predicted Reliability**
O=S1(=O)CCCC2=C1C(N1C(SC=C1C1=CC=CC=C1)=N2)C1=CC=CC=C1	Active	0.6	0.6	0.4	reliable

6	Drosophila melanogaster				
**Molecule**	**Predicted Outcome**	**Probability**	**Probability Active**	**Probability Inactive**	**Predicted Reliability**
O=S1(=O)CCCC2=C1C(N1C(SC=C1C1=CC=CC=C1)=N2)C1=CC=CC=C1	Inactive	1.0	0.0	1.0	unreliable

7	Leishmania major				
Molecule	Predicted Outcome	Probability	Probability Active	Probability Inactive	Predicted Reliability
O=S1(=O)CCCC2=C1C(N1C(SC=C1C1=CC=CC=C1)=N2)C1=CC=CC=C1	Inactive	1.0	0.0	1.0	reliable

8	Alphis gossypii				
Molecule	Predicted Outcome	Probability	Probability Active	Probability Inactive	Predicted Reliability
O=S1(=O)CCCC2=C1C(N1C(SC=C1C1=CC=CC=C1)=N2)C1=CC=CC=C1	Active	1.0	1.0	0.0	unreliable

9	C_albicans				
Molecule	Predicted Outcome	Probability	Probability Active	Probability Inactive	Predicted Reliability
O=S1(=O)CCCC2=C1C(N1C(SC=C1C1=CC=CC=C1)=N2)C1=CC=CC=C1	Active	0.8	0.8	0.2	reliable

10	Salmonella				
Molecule	Predicted Outcome	Probability	Probability Active	Probability Inactive	Predicted Reliability
O=S1(=O)CCCC2=C1C(N1C(SC=C1C1=CC=CC=C1)=N2)C1=CC=CC=C1	Inactive	1.0	0.0	1.0	unreliable

11	Leishmania infantum - Promastigota				
Molecule	Predicted Outcome	Probability	Probability Active	Probability Inactive	Predicted Reliability
O=S1(=O)CCCC2=C1C(N1C(SC=C1C1=CC=CC=C1)=N2)C1=CC=CC=C1	Inactive	1.0	0.0	1.0	reliable

12	E_coli				
Molecule	Predicted Outcome	Probability	Probability Active	Probability Inactive	Predicted Reliability
O=S1(=O)CCCC2=C1C(N1C(SC=C1C1=CC=CC=C1)=N2)C1=CC=CC=C1	Inactive	1.0	0.0	1.0	reliable

13	Leishmania braziliensis				
Molecule	Predicted Outcome	Probability	Probability Active	Probability Inactive	Predicted Reliability
O=S1(=O)CCCC2=C1C(N1C(SC=C1C1=CC=CC=C1)=N2)C1=CC=CC=C1	Active	0.6	0.6	0.4	reliable

14	Tcruzi_amastigota				
Molecule	Predicted Outcome	Probability	Probability Active	Probability Inactive	Predicted Reliability
O=S1(=O)CCCC2=C1C(N1C(SC=C1C1=CC=CC=C1)=N2)C1=CC=CC=C1	Inactive	0.8	0.2	0.8	reliable

15	Tcruzi_epimastigota				
Molecule	Predicted Outcome	Probability	Probability Active	Probability Inactive	Predicted Reliability
O=S1(=O)CCCC2=C1C(N1C(SC=C1C1=CC=CC=C1)=N2)C1=CC=CC=C1	Active	1.0	1.0	0.0	reliable

16	Tcruzi_trypomastigota				
Molecule	Predicted Outcome	Probability	Probability Active	Probability Inactive	Predicted Reliability
O=S1(=O)CCCC2=C1C(N1C(SC=C1C1=CC=CC=C1)=N2)C1=CC=CC=C1	Inactive	1.0	0.0	1.0	reliable

17	Sars-Cov				
Molecule	Predicted Outcome	Probability	Probability Active	Probability Inactive	Predicted Reliability
O=S1(=O)CCCC2=C1C(N1C(SC=C1C1=CC=CC=C1)=N2)C1=CC=CC=C1	Inactive	0.6	0.4	0.6	unreliable

18	Dengue larvicida				
Molecule	Predicted Outcome	Probability	Probability Active	Probability Inactive	Predicted Reliability
O=S1(=O)CCCC2=C1C(N1C(SC=C1C1=CC=CC=C1)=N2)C1=CC=CC=C1	Inactive	0.6	0.4	0.6	reliable

19	Acetylcholinesterase				
Molecule	Predicted Outcome	Probability	Probability Active	Probability Inactive	Predicted Reliability
O=S1(=O)CCCC2=C1C(N1C(SC=C1C1=CC=CC=C1)=N2)C1=CC=CC=C1	Inactive	1.0	0.0	1.0	reliable

20	Epimastigote Chagas				
Molecule	Predicted Outcome	Probability	Probability Active	Probability Inactive	Predicted Reliability
O=S1(=O)CCCC2=C1C(N1C(SC=C1C1=CC=CC=C1)=N2)C1=CC=CC=C1	Inactive	1.0	0.0	1.0	reliable

21	Alzheimer - COX2				
Molecule	Predicted Outcome	Probability	Probability Active	Probability Inactive	Predicted Reliability
O=S1(=O)CCCC2=C1C(N1C(SC=C1C1=CC=CC=C1)=N2)C1=CC=CC=C1	Inactive	0.8	0.2	0.8	reliable

22	PTR L major				
Molecule	Predicted Outcome	Probability	Probability Active	Probability Inactive	Predicted Reliability
O=S1(=O)CCCC2=C1C(N1C(SC=C1C1=CC=CC=C1)=N2)C1=CC=CC=C1	Inactive	1.0	0.0	1.0	reliable

23	Promastigote Ldonovani				
Molecule	Predicted Outcome	Probability	Probability Active	Probability Inactive	Predicted Reliability
O=S1(=O)CCCC2=C1C(N1C(SC=C1C1=CC=CC=C1)=N2)C1=CC=CC=C1	Inactive	0.6	0.4	0.6	reliable

24	Tripomastigote Chagas				
Molecule	Predicted Outcome	Probability	Probability Active	Probability Inactive	Predicted Reliability
O=S1(=O)CCCC2=C1C(N1C(SC=C1C1=CC=CC=C1)=N2)C1=CC=CC=C1	Active	0.6	0.6	0.4	reliable

25	Lamazonensis_amastigota				
Molecule	Predicted Outcome	Probability	Probability Active	Probability Inactive	Predicted Reliability
O=S1(=O)CCCC2=C1C(N1C(SC=C1C1=CC=CC=C1)=N2)C1=CC=CC=C1	Inactive	1.0	0.0	1.0	unreliable

26	Lamazonensis_promastigota				
Molecule	Predicted Outcome	Probability	Probability Active	Probability Inactive	Predicted Reliability
O=S1(=O)CCCC2=C1C(N1C(SC=C1C1=CC=CC=C1)=N2)C1=CC=CC=C1	Inactive	0.8	0.2	0.8	reliable

27	Alzheimer - iNOS				
Molecule	Predicted Outcome	Probability	Probability Active	Probability Inactive	Predicted Reliability
O=S1(=O)CCCC2=C1C(N1C(SC=C1C1=CC=CC=C1)=N2)C1=CC=CC=C1	Inactive	0.8	0.2	0.8	reliable

28	Alzheimer - NADPH				
Molecule	Predicted Outcome	Probability	Probability Active	Probability Inactive	Predicted Reliability
O=S1(=O)CCCC2=C1C(N1C(SC=C1C1=CC=CC=C1)=N2)C1=CC=CC=C1	Inactive	1.0	0.0	1.0	reliable

29	Alzheimer - JNK-3				
Molecule	Predicted Outcome	Probability	Probability Active	Probability Inactive	Predicted Reliability
O=S1(=O)CCCC2=C1C(N1C(SC=C1C1=CC=CC=C1)=N2)C1=CC=CC=C1	Inactive	1.0	0.0	1.0	reliable

30	Tcruzi_amastigota				
Molecule	Predicted Outcome	Probability	Probability Active	Probability Inactive	Predicted Reliability
O=S1(=O)CCCC2=C1C(N1C(SC=C1C1=CC=CC=C1)=N2)C1=CC=CC=C1	Inactive	0.8	0.2	0.8	reliable

31	Tcruzi_epimastigota				
Molecule	Predicted Outcome	Probability	Probability Active	Probability Inactive	Predicted Reliability
O=S1(=O)CCCC2=C1C(N1C(SC=C1C1=CC=CC=C1)=N2)C1=CC=CC=C1	Active	1.0	1.0	0.0	reliable

32	Tcruzi_trypomastigota				
Molecule	Predicted Outcome	Probability	Probability Active	Probability Inactive	Predicted Reliability
O=S1(=O)CCCC2=C1C(N1C(SC=C1C1=CC=CC=C1)=N2)C1=CC=CC=C1	Inactive	1.0	0.0	1.0	reliable

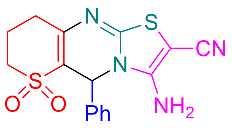

4

NC1=C(SC2=NC3=C(C(N12)C1=CC=CC=C1)S(=O)(=O)CCC3)C#N

Molecule 1

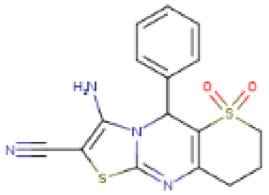

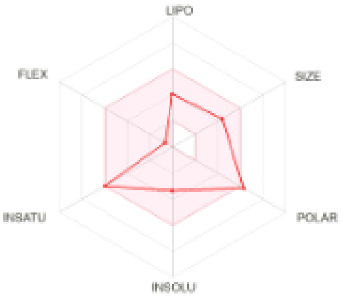

SMILES N#Cc1sc2=NC3=C(C(n2c1N)c1ccccc1)S(=O)(=O)CCC3

Physicochemical Properties	
Formula	C16H14N4O2S2
Molecular weight	358.44 g/mol
Num. heavy atoms	24
Num. arom. heavy atoms	11
Fraction Csp3	0.25
Num. rotatable bonds	1
Num. H-bond acceptors	4
Num. H-bond donors	1
Molar Refractivity	95.42
TPSA	137.86 Å²

Lipophilicity	
Log $P_{o/w}$ (iLOGP)	2.09
Log $P_{o/w}$ (XLOGP3)	1.58
Log $P_{o/w}$ (WLOGP)	2.64
Log $P_{o/w}$ (MLOGP)	1.11
Log $P_{o/w}$ (SILICOS-IT)	2.50
Consensus Log $P_{o/w}$	1.98

Water Solubility	
Log *S* (ESOL)	-3.33
Solubility	1.67e-01 mg/ml ; 4.67e-04 mol/l
Class	Soluble
Log *S* (Ali)	-4.09
Solubility	2.94e-02 mg/ml ; 8.21e-05 mol/l
Class	Moderately soluble
Log *S* (SILICOS-IT)	-4.04
Solubility	3.24e-02 mg/ml ; 9.04e-05 mol/l
Class	Moderately soluble

Pharmacokinetics	
GI absorption	Low
BBB permeant	No
P-gp substrate	No
CYP1A2 inhibitor	No
CYP2C19 inhibitor	Yes
CYP2C9 inhibitor	No
CYP2D6 inhibitor	No
CYP3A4 inhibitor	Yes
Log K_p (skin permeation)	-7.36 cm/s

Druglikeness	
Lipinski	Yes; 0 violation
Ghose	Yes
Veber	Yes
Egan	No; 1 violation: TPSA>131.6
Muegge	Yes
Bioavailability Score	0.55

Medicinal Chemistry	
PAINS	0 alert
Brenk	0 alert
Leadlikeness	No; 1 violation: MW>350
Synthetic accessibility	4.32

Oral toxicity prediction results for input compound

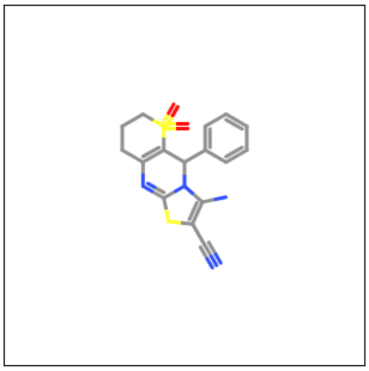

Predicted LD50: 1000mg/kg

Predicted Toxicity Class: 4

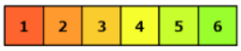

Average similarity: 38.2%

Prediction accuracy: 23%

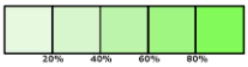

Print Toxicity Report

Name	
Molweight	358.44
Number of hydrogen bond acceptors	7
Number of hydrogen bond donors	1
Number of atoms	24
Number of bonds	27
Number of rotatable bonds	1
Molecular refractivity	100.21
Topological Polar Surface Area	133.23
octanol/water partition coefficient(logP)	3.37

1	Sars-Cov				
**Molecule**	**Predicted Outcome**	**Probability**	**Probability Active**	**Probability Inactive**	**Predicted Reliability**
NC1=C(SC2=NC3=C(C(N12)C1=CC=CC=C1)S(=O)(=O)CCC3)C#N	Inactive	0.6	0.4	0.6	unreliable

2	Dengue larvicida				
**Molecule**	**Predicted Outcome**	**Probability**	**Probability Active**	**Probability Inactive**	**Predicted Reliability**
NC1=C(SC2=NC3=C(C(N12)C1=CC=CC=C1)S(=O)(=O)CCC3)C#N	Inactive	0.6	0.4	0.6	reliable

3	Acetylcholinesterase				
**Molecule**	**Predicted Outcome**	**Probability**	**Probability Active**	**Probability Inactive**	**Predicted Reliability**
NC1=C(SC2=NC3=C(C(N12)C1=CC=CC=C1)S(=O)(=O)CCC3)C#N	Inactive	1.0	0.0	1.0	reliable

4	Salmonella				
**Molecule**	**Predicted Outcome**	**Probability**	**Probability Active**	**Probability Inactive**	**Predicted Reliability**
NC1=C(SC2=NC3=C(C(N12)C1=CC=CC=C1)S(=O)(=O)CCC3)C#N	Active	0.6	0.6	0.4	unreliable

5	C_albicans				
**Molecule**	**Predicted Outcome**	**Probability**	**Probability Active**	**Probability Inactive**	**Predicted Reliability**
NC1=C(SC2=NC3=C(C(N12)C1=CC=CC=C1)S(=O)(=O)CCC3)C#N	Active	0.8	0.8	0.2	reliable

6	E_coli				
**Molecule**	**Predicted Outcome**	**Probability**	**Probability Active**	**Probability Inactive**	**Predicted Reliability**
NC1=C(SC2=NC3=C(C(N12)C1=CC=CC=C1)S(=O)(=O)CCC3)C#N	Inactive	1.0	0.0	1.0	unreliable

7	Hepatite C - Type1				
**Molecule**	**Predicted Outcome**	**Probability**	**Probability Active**	**Probability Inactive**	**Predicted Reliability**
NC1=C(SC2=NC3=C(C(N12)C1=CC=CC=C1)S(=O)(=O)CCC3)C#N	Inactive	0.6	0.4	0.6	unreliable

8	Hepatite C - NS3-protease helicase				
**Molecule**	**Predicted Outcome**	**Probability**	**Probability Active**	**Probability Inactive**	**Predicted Reliability**
NC1=C(SC2=NC3=C(C(N12)C1=CC=CC=C1)S(=O)(=O)CCC3)C#N	Inactive	1.0	0.0	1.0	unreliable

9	Hepatite C - RNA dependent				
**Molecule**	**Predicted Outcome**	**Probability**	**Probability Active**	**Probability Inactive**	**Predicted Reliability**
NC1=C(SC2=NC3=C(C(N12)C1=CC=CC=C1)S(=O)(=O)CCC3)C#N	Inactive	1.0	0.0	1.0	unreliable

10	Hepatite C - Serine protease				
**Molecule**	**Predicted Outcome**	**Probability**	**Probability Active**	**Probability Inactive**	**Predicted Reliability**
NC1=C(SC2=NC3=C(C(N12)C1=CC=CC=C1)S(=O)(=O)CCC3)C#N	Inactive	1.0	0.0	1.0	unreliable

11	Leishmania infantum - Promastigota				
**Molecule**	**Predicted Outcome**	**Probability**	**Probability Active**	**Probability Inactive**	**Predicted Reliability**
NC1=C(SC2=NC3=C(C(N12)C1=CC=CC=C1)S(=O)(=O)CCC3)C#N	Inactive	1.0	0.0	1.0	reliable

12	Leishmania amazonensis - Promastigota				
**Molecule**	**Predicted Outcome**	**Probability**	**Probability Active**	**Probability Inactive**	**Predicted Reliability**
NC1=C(SC2=NC3=C(C(N12)C1=CC=CC=C1)S(=O)(=O)CCC3)C#N	Inactive	0.8	0.2	0.8	reliable

13	Leishmania braziliensis				
Molecule	Predicted Outcome	Probability	Probability Active	Probability Inactive	Predicted Reliability
NC1=C(SC2=NC3=C(C(N12)C1=CC=CC=C1)S(=O)(=O)CCC3)C#N	Active	0.6	0.6	0.4	unreliable

14	Drosophila melanogaster				
Molecule	Predicted Outcome	Probability	Probability Active	Probability Inactive	Predicted Reliability
NC1=C(SC2=NC3=C(C(N12)C1=CC=CC=C1)S(=O)(=O)CCC3)C#N	Inactive	1.0	0.0	1.0	unreliable

15	Alphis gossypii				
Molecule	Predicted Outcome	Probability	Probability Active	Probability Inactive	Predicted Reliability
NC1=C(SC2=NC3=C(C(N12)C1=CC=CC=C1)S(=O)(=O)CCC3)C#N	Active	1.0	1.0	0.0	unreliable

16	Leishmania major				
Molecule	Predicted Outcome	Probability	Probability Active	Probability Inactive	Predicted Reliability
NC1=C(SC2=NC3=C(C(N12)C1=CC=CC=C1)S(=O)(=O)CCC3)C#N	Inactive	1.0	0.0	1.0	unreliable

17	Alzheimer - iNOS				
Molecule	Predicted Outcome	Probability	Probability Active	Probability Inactive	Predicted Reliability
NC1=C(SC2=NC3=C(C(N12)C1=CC=CC=C1)S(=O)(=O)CCC3)C#N	Active	0.6	0.6	0.4	reliable

18	Alzheimer - COX2				
Molecule	Predicted Outcome	Probability	Probability Active	Probability Inactive	Predicted Reliability
NC1=C(SC2=NC3=C(C(N12)C1=CC=CC=C1)S(=O)(=O)CCC3)C#N	Inactive	1.0	0.0	1.0	reliable

19	Alzheimer - JNK-3				
Molecule	Predicted Outcome	Probability	Probability Active	Probability Inactive	Predicted Reliability
NC1=C(SC2=NC3=C(C(N12)C1=CC=CC=C1)S(=O)(=O)CCC3)C#N	Inactive	1.0	0.0	1.0	unreliable

20	Alzheimer - NADPH				
Molecule	Predicted Outcome	Probability	Probability Active	Probability Inactive	Predicted Reliability
NC1=C(SC2=NC3=C(C(N12)C1=CC=CC=C1)S(=O)(=O)CCC3)C#N	Inactive	0.8	0.2	0.8	reliable

21	Alzheimer - PDE5				
Molecule	Predicted Outcome	Probability	Probability Active	Probability Inactive	Predicted Reliability
NC1=C(SC2=NC3=C(C(N12)C1=CC=CC=C1)S(=O)(=O)CCC3)C#N	Inactive	1.0	0.0	1.0	unreliable

22	Amastigote Chagas				
Molecule	Predicted Outcome	Probability	Probability Active	Probability Inactive	Predicted Reliability
NC1=C(SC2=NC3=C(C(N12)C1=CC=CC=C1)S(=O)(=O)CCC3)C#N	Inactive	1.0	0.0	1.0	reliable

23	Promastigote Ldonovani				
Molecule	Predicted Outcome	Probability	Probability Active	Probability Inactive	Predicted Reliability
NC1=C(SC2=NC3=C(C(N12)C1=CC=CC=C1)S(=O)(=O)CCC3)C#N	Inactive	0.8	0.2	0.8	reliable

24	Amastigote Ldonovani				
Molecule	Predicted Outcome	Probability	Probability Active	Probability Inactive	Predicted Reliability
NC1=C(SC2=NC3=C(C(N12)C1=CC=CC=C1)S(=O)(=O)CCC3)C#N	Inactive	1.0	0.0	1.0	reliable

25	Epimastigote Chagas				
Molecule	Predicted Outcome	Probability	Probability Active	Probability Inactive	Predicted Reliability
NC1=C(SC2=NC3=C(C(N12)C1=CC=CC=C1)S(=O)(=O)CCC3)C#N	Inactive	0.8	0.2	0.8	reliable

26	PTR L major				
Molecule	Predicted Outcome	Probability	Probability Active	Probability Inactive	Predicted Reliability
NC1=C(SC2=NC3=C(C(N12)C1=CC=CC=C1)S(=O)(=O)CCC3)C#N	Inactive	1.0	0.0	1.0	unreliable

27	Lamazonensis_amastigota				
Molecule	Predicted Outcome	Probability	Probability Active	Probability Inactive	Predicted Reliability
NC1=C(SC2=NC3=C(C(N12)C1=CC=CC=C1)S(=O)(=O)CCC3)C#N	Inactive	1.0	0.0	1.0	unreliable

28	Tripomastigote Chagas				
Molecule	Predicted Outcome	Probability	Probability Active	Probability Inactive	Predicted Reliability
NC1=C(SC2=NC3=C(C(N12)C1=CC=CC=C1)S(=O)(=O)CCC3)C#N	Inactive	0.6	0.4	0.6	unreliable

29	Lamazonensis_promastigota				
**Molecule**	**Predicted Outcome**	**Probability**	**Probability Active**	**Probability Inactive**	**Predicted Reliability**
NC1=C(SC2=NC3=C(C(N12)C1=CC=CC=C1)S(=O)(=O)CCC3)C#N	Inactive	0.8	0.2	0.8	reliable

30	Tcruzi_trypomastigota				
**Molecule**	**Predicted Outcome**	**Probability**	**Probability Active**	**Probability Inactive**	**Predicted Reliability**
NC1=C(SC2=NC3=C(C(N12)C1=CC=CC=C1)S(=O)(=O)CCC3)C#N	Active	1.0	1.0	0.0	unreliable

31	Tcruzi_epimastigota				
**Molecule**	**Predicted Outcome**	**Probability**	**Probability Active**	**Probability Inactive**	**Predicted Reliability**
NC1=C(SC2=NC3=C(C(N12)C1=CC=CC=C1)S(=O)(=O)CCC3)C#N	Active	1.0	1.0	0.0	reliable

32	Tcruzi_amastigota				
**Molecule**	**Predicted Outcome**	**Probability**	**Probability Active**	**Probability Inactive**	**Predicted Reliability**
NC1=C(SC2=NC3=C(C(N12)C1=CC=CC=C1)S(=O)(=O)CCC3)C#N	Inactive	1.0	0.0	1.0	reliable

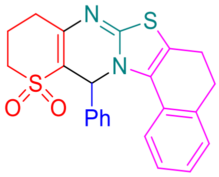

5

O=S1(=O)CCCC2=C1C(N1C(SC3=C1C1=C(CC3)C=CC=C1)=N2)C1=CC=CC=C1

Molecule 1

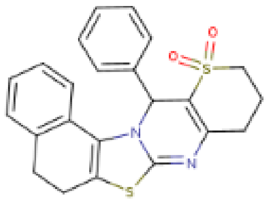

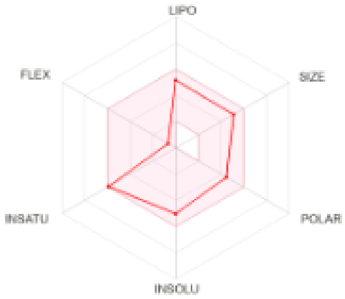

SMILES O=S1(=O)CCCC2=C1C(c1ccccc1)n1c(=N2)sc2c1c1ccccc1CC2

Physicochemical Properties	
Formula	C23H20N2O2S2
Molecular weight	420.55 g/mol
Num. heavy atoms	29
Num. arom. heavy atoms	17
Fraction Csp3	0.26
Num. rotatable bonds	1
Num. H-bond acceptors	3
Num. H-bond donors	0
Molar Refractivity	119.55
TPSA	88.05 Å²

Lipophilicity	
Log $P_{o/w}$ (iLOGP)	3.20
Log $P_{o/w}$ (XLOGP3)	3.51
Log $P_{o/w}$ (WLOGP)	4.94
Log $P_{o/w}$ (MLOGP)	3.53
Log $P_{o/w}$ (SILICOS-IT)	5.36
Consensus Log $P_{o/w}$	4.11

Water Solubility	
Log *S* (ESOL)	-5.03
Solubility	3.96e-03 mg/ml ; 9.41e-06 mol/l
Class	Moderately soluble
Log *S* (Ali)	-5.04
Solubility	3.81e-03 mg/ml ; 9.07e-06 mol/l
Class	Moderately soluble
Log *S* (SILICOS-IT)	-7.33
Solubility	1.95e-05 mg/ml ; 4.63e-08 mol/l
Class	Poorly soluble

Pharmacokinetics	
GI absorption	High
BBB permeant	No
P-gp substrate	No
CYP1A2 inhibitor	Yes
CYP2C19 inhibitor	Yes
CYP2C9 inhibitor	Yes
CYP2D6 inhibitor	No
CYP3A4 inhibitor	Yes
Log K_p (skin permeation)	-6.37 cm/s

Druglikeness	
Lipinski	Yes; 0 violation
Ghose	Yes
Veber	Yes
Egan	Yes
Muegge	Yes
Bioavailability Score	0.55

Medicinal Chemistry	
PAINS	0 alert
Brenk	0 alert
Leadlikeness	No; 2 violations: MW>350, XLOGP3>3.5
Synthetic accessibility	4.73

Oral toxicity prediction results for input compound

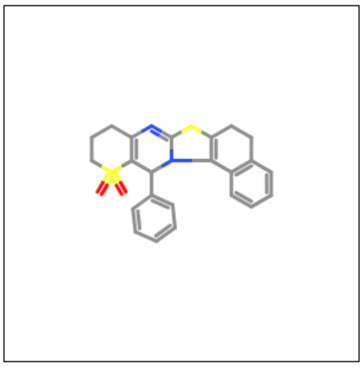

Predicted LD50: 1000mg/kg

Predicted Toxicity Class: 4

1	2	3	4	5	6

Average similarity: 41.53%

Prediction accuracy: 54.26%

20% 40% 60% 80%

Print Toxicity Report

Name	O=S1(=O)CCCC2=C1C(
Molweight	420.55
Number of hydrogen bond acceptors	5
Number of hydrogen bond donors	0
Number of atoms	29
Number of bonds	34
Number of rotable bonds	1
Molecular refractivity	125.89
Topological Polar Surface Area	83.42
octanol/water partition coefficient(logP)	5.34

1	Sars-Cov				
Molecule	Predicted Outcome	Probability	Probability Active	Probability Inactive	Predicted Reliability
O=S1(=O)CCCC2=C1C(N1C(SC3=C1C1=C(CC3)C=CC=C1)=N2)C1=CC=CC=C1	Inactive	0.6	0.4	0.6	unreliable

2	Dengue larvicida				
Molecule	Predicted Outcome	Probability	Probability Active	Probability Inactive	Predicted Reliability
O=S1(=O)CCCC2=C1C(N1C(SC3=C1C1=C(CC3)C=CC=C1)=N2)C1=CC=CC=C1	Active	0.6	0.6	0.4	reliable

3	Salmonella				
Molecule	Predicted Outcome	Probability	Probability Active	Probability Inactive	Predicted Reliability
O=S1(=O)CCCC2=C1C(N1C(SC3=C1C1=C(CC3)C=CC=C1)=N2)C1=CC=CC=C1	Inactive	1.0	0.0	1.0	unreliable

4	E_coli				
Molecule	Predicted Outcome	Probability	Probability Active	Probability Inactive	Predicted Reliability
O=S1(=O)CCCC2=C1C(N1C(SC3=C1C1=C(CC3)C=CC=C1)=N2)C1=CC=CC=C1	Inactive	1.0	0.0	1.0	reliable

5	Acetylcholinesterase				
Molecule	Predicted Outcome	Probability	Probability Active	Probability Inactive	Predicted Reliability
O=S1(=O)CCCC2=C1C(N1C(SC3=C1C1=C(CC3)C=CC=C1)=N2)C1=CC=CC=C1	Inactive	1.0	0.0	1.0	reliable

6	Hepatite C - Type1				
Molecule	Predicted Outcome	Probability	Probability Active	Probability Inactive	Predicted Reliability
O=S1(=O)CCCC2=C1C(N1C(SC3=C1C1=C(CC3)C=CC=C1)=N2)C1=CC=CC=C1	Active	0.6	0.6	0.4	unreliable

7	Hepatite C - NS3-protease helicase				
Molecule	Predicted Outcome	Probability	Probability Active	Probability Inactive	Predicted Reliability
O=S1(=O)CCCC2=C1C(N1C(SC3=C1C1=C(CC3)C=CC=C1)=N2)C1=CC=CC=C1	Inactive	1.0	0.0	1.0	unreliable

8	Hepatite C - Serine protease				
Molecule	Predicted Outcome	Probability	Probability Active	Probability Inactive	Predicted Reliability
O=S1(=O)CCCC2=C1C(N1C(SC3=C1C1=C(CC3)C=CC=C1)=N2)C1=CC=CC=C1	Inactive	1.0	0.0	1.0	unreliable

9	C_albicans				
Molecule	Predicted Outcome	Probability	Probability Active	Probability Inactive	Predicted Reliability
O=S1(=O)CCCC2=C1C(N1C(SC3=C1C1=C(CC3)C=CC=C1)=N2)C1=CC=CC=C1	Active	0.8	0.8	0.2	reliable

10	Hepatite C - RNA dependent				
Molecule	Predicted Outcome	Probability	Probability Active	Probability Inactive	Predicted Reliability
O=S1(=O)CCCC2=C1C(N1C(SC3=C1C1=C(CC3)C=CC=C1)=N2)C1=CC=CC=C1	Inactive	1.0	0.0	1.0	unreliable

11	Leishmania amazonensis - Promastigota				
Molecule	Predicted Outcome	Probability	Probability Active	Probability Inactive	Predicted Reliability
O=S1(=O)CCCC2=C1C(N1C(SC3=C1C1=C(CC3)C=CC=C1)=N2)C1=CC=CC=C1	Inactive	0.8	0.2	0.8	reliable

12	Leishmania infantum - Promastigota				
Molecule	Predicted Outcome	Probability	Probability Active	Probability Inactive	Predicted Reliability
O=S1(=O)CCCC2=C1C(N1C(SC3=C1C1=C(CC3)C=CC=C1)=N2)C1=CC=CC=C1	Inactive	1.0	0.0	1.0	unreliable

13	Leishmania braziliensis				
Molecule	Predicted Outcome	Probability	Probability Active	Probability Inactive	Predicted Reliability
O=S1(=O)CCCC2=C1C(N1C(SC3=C1C1=C(CC3)C=CC=C1)=N2)C1=CC=CC=C1	Active	0.6	0.6	0.4	unreliable

14	Drosophila melanogaster				
Molecule	Predicted Outcome	Probability	Probability Active	Probability Inactive	Predicted Reliability
O=S1(=O)CCCC2=C1C(N1C(SC3=C1C1=C(CC3)C=CC=C1)=N2)C1=CC=CC=C1	Inactive	1.0	0.0	1.0	unreliable

15	Leishmania major				
Molecule	Predicted Outcome	Probability	Probability Active	Probability Inactive	Predicted Reliability
O=S1(=O)CCCC2=C1C(N1C(SC3=C1C1=C(CC3)C=CC=C1)=N2)C1=CC=CC=C1	Inactive	0.6	0.4	0.6	reliable

16	Alphis gossypii				
Molecule	Predicted Outcome	Probability	Probability Active	Probability Inactive	Predicted Reliability
O=S1(=O)CCCC2=C1C(N1C(SC3=C1C1=C(CC3)C=CC=C1)=N2)C1=CC=CC=C1	Active	1.0	1.0	0.0	unreliable

17	Alzheimer - iNOS				
Molecule	Predicted Outcome	Probability	Probability Active	Probability Inactive	Predicted Reliability
O=S1(=O)CCCC2=C1C(N1C(SC3=C1C1=C(CC3)C=CC=C1)=N2)C1=CC=CC=C1	Inactive	0.6	0.4	0.6	reliable

18	Alzheimer - JNK-3				
Molecule	Predicted Outcome	Probability	Probability Active	Probability Inactive	Predicted Reliability
O=S1(=O)CCCC2=C1C(N1C(SC3=C1C1=C(CC3)C=CC=C1)=N2)C1=CC=CC=C1	Inactive	1.0	0.0	1.0	reliable

19	Alzheimer - COX2				
Molecule	Predicted Outcome	Probability	Probability Active	Probability Inactive	Predicted Reliability
O=S1(=O)CCCC2=C1C(N1C(SC3=C1C1=C(CC3)C=CC=C1)=N2)C1=CC=CC=C1	Inactive	0.8	0.2	0.8	reliable

20	Alzheimer - NADPH				
Molecule	Predicted Outcome	Probability	Probability Active	Probability Inactive	Predicted Reliability
O=S1(=O)CCCC2=C1C(N1C(SC3=C1C1=C(CC3)C=CC=C1)=N2)C1=CC=CC=C1	Inactive	0.8	0.2	0.8	reliable

21	Alzheimer - PDE5				
Molecule	Predicted Outcome	Probability	Probability Active	Probability Inactive	Predicted Reliability
O=S1(=O)CCCC2=C1C(N1C(SC3=C1C1=C(CC3)C=CC=C1)=N2)C1=CC=CC=C1	Inactive	1.0	0.0	1.0	unreliable

22	Amastigote Chagas				
Molecule	Predicted Outcome	Probability	Probability Active	Probability Inactive	Predicted Reliability
O=S1(=O)CCCC2=C1C(N1C(SC3=C1C1=C(CC3)C=CC=C1)=N2)C1=CC=CC=C1	Inactive	1.0	0.0	1.0	reliable

23	Epimastigote Chagas				
Molecule	Predicted Outcome	Probability	Probability Active	Probability Inactive	Predicted Reliability
O=S1(=O)CCCC2=C1C(N1C(SC3=C1C1=C(CC3)C=CC=C1)=N2)C1=CC=CC=C1	Inactive	1.0	0.0	1.0	reliable

24	Promastigote Ldonovani				
Molecule	Predicted Outcome	Probability	Probability Active	Probability Inactive	Predicted Reliability
O=S1(=O)CCCC2=C1C(N1C(SC3=C1C1=C(CC3)C=CC=C1)=N2)C1=CC=CC=C1	Inactive	0.8	0.2	0.8	reliable

25	Amastigote Ldonovani				
Molecule	Predicted Outcome	Probability	Probability Active	Probability Inactive	Predicted Reliability
O=S1(=O)CCCC2=C1C(N1C(SC3=C1C1=C(CC3)C=CC=C1)=N2)C1=CC=CC=C1	Inactive	1.0	0.0	1.0	reliable

26	PTR L major				
Molecule	Predicted Outcome	Probability	Probability Active	Probability Inactive	Predicted Reliability
O=S1(=O)CCCC2=C1C(N1C(SC3=C1C1=C(CC3)C=CC=C1)=N2)C1=CC=CC=C1	Active	0.6	0.6	0.4	reliable

27	Lamazonensis_amastigota				
Molecule	Predicted Outcome	Probability	Probability Active	Probability Inactive	Predicted Reliability
O=S1(=O)CCCC2=C1C(N1C(SC3=C1C1=C(CC3)C=CC=C1)=N2)C1=CC=CC=C1	Inactive	1.0	0.0	1.0	unreliable

28	Tripomastigote Chagas				
Molecule	Predicted Outcome	Probability	Probability Active	Probability Inactive	Predicted Reliability
O=S1(=O)CCCC2=C1C(N1C(SC3=C1C1=C(CC3)C=CC=C1)=N2)C1=CC=CC=C1	Active	0.8	0.8	0.2	unreliable

29	Lamazonensis_promastigota				
**Molecule**	**Predicted Outcome**	**Probability**	**Probability Active**	**Probability Inactive**	**Predicted Reliability**
O=S1(=O)CCCC2=C1C(N1C(SC3=C1C1=C(CC3)C=CC=C1)=N2)C1=CC=CC=C1	Inactive	0.8	0.2	0.8	reliable

30	Tcruzi_epimastigota				
**Molecule**	**Predicted Outcome**	**Probability**	**Probability Active**	**Probability Inactive**	**Predicted Reliability**
O=S1(=O)CCCC2=C1C(N1C(SC3=C1C1=C(CC3)C=CC=C1)=N2)C1=CC=CC=C1	Active	1.0	1.0	0.0	reliable

31	Tcruzi_amastigota				
**Molecule**	**Predicted Outcome**	**Probability**	**Probability Active**	**Probability Inactive**	**Predicted Reliability**
O=S1(=O)CCCC2=C1C(N1C(SC3=C1C1=C(CC3)C=CC=C1)=N2)C1=CC=CC=C1	Inactive	0.6	0.4	0.6	reliable

32	Tcruzi_trypomastigota				
**Molecule**	**Predicted Outcome**	**Probability**	**Probability Active**	**Probability Inactive**	**Predicted Reliability**
O=S1(=O)CCCC2=C1C(N1C(SC3=C1C1=C(CC3)C=CC=C1)=N2)C1=CC=CC=C1	Inactive	1.0	0.0	1.0	unreliable

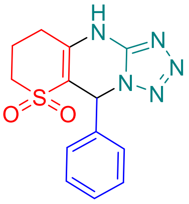

6

O=S1(=O)CCCC2=C1C(N1N=NN=C1N2)C1=CC=CC=C1

Molecule 1

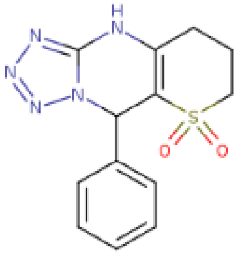

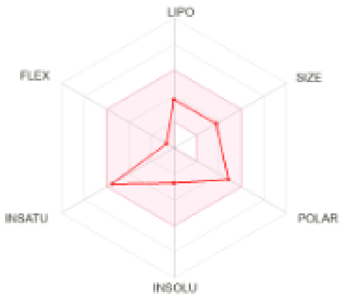

SMILES O=S1(=O)CCCC2=C1C(c1ccccc1)n1c(N2)nnn1

Physicochemical Properties	
Formula	C13H13N5O2S
Molecular weight	303.34 g/mol
Num. heavy atoms	21
Num. arom. heavy atoms	11
Fraction Csp3	0.31
Num. rotatable bonds	1
Num. H-bond acceptors	5
Num. H-bond donors	1
Molar Refractivity	79.30
TPSA	98.15 Å²

Lipophilicity	
Log $P_{o/w}$ (iLOGP)	1.54
Log $P_{o/w}$ (XLOGP3)	1.04
Log $P_{o/w}$ (WLOGP)	1.62
Log $P_{o/w}$ (MLOGP)	1.72
Log $P_{o/w}$ (SILICOS-IT)	-0.07
Consensus Log $P_{o/w}$	1.17

Water Solubility	
Log *S* (ESOL)	-2.70
Solubility	6.09e-01 mg/ml ; 2.01e-03 mol/l
Class	Soluble
Log *S* (Ali)	-2.69
Solubility	6.17e-01 mg/ml ; 2.03e-03 mol/l
Class	Soluble
Log *S* (SILICOS-IT)	-3.55
Solubility	8.63e-02 mg/ml ; 2.84e-04 mol/l
Class	Soluble

Pharmacokinetics	
GI absorption	High
BBB permeant	No
P-gp substrate	Yes
CYP1A2 inhibitor	No
CYP2C19 inhibitor	No
CYP2C9 inhibitor	No
CYP2D6 inhibitor	No
CYP3A4 inhibitor	No
Log K_p (skin permeation)	-7.41 cm/s

Druglikeness	
Lipinski	Yes; 0 violation
Ghose	Yes
Veber	Yes
Egan	Yes
Muegge	Yes
Bioavailability Score	0.55

Medicinal Chemistry	
PAINS	0 alert
Brenk	0 alert
Leadlikeness	Yes
Synthetic accessibility	4.14

Oral toxicity prediction results for input compound

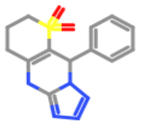

Predicted LD50: 2025mg/kg

Predicted Toxicity Class: 5

1	2	3	4	5	6

Average similarity: 38.3%

Prediction accuracy: 23%

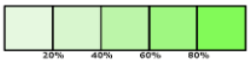

Name	O=S1(=O)CCCC2=C1C(
Molweight	303.34
Number of hydrogen bond acceptors	6
Number of hydrogen bond donors	1
Number of atoms	21
Number of bonds	24
Number of rotable bonds	1
Molecular refractivity	79.3
Topological Polar Surface Area	98.15
octanol/water partition coefficient(logP)	2.33

Print Toxicity Report

1	Dengue larvicida				
**Molecule**	**Predicted Outcome**	**Probability**	**Probability Active**	**Probability Inactive**	**Predicted Reliability**
O=S1(=O)CCCC2=C1C(N1N=NN=C1N2)C1=CC=CC=C1	Inactive	1.0	0.0	1.0	reliable

2	Salmonella				
**Molecule**	**Predicted Outcome**	**Probability**	**Probability Active**	**Probability Inactive**	**Predicted Reliability**
O=S1(=O)CCCC2=C1C(N1N=NN=C1N2)C1=CC=CC=C1	Inactive	0.8	0.2	0.8	reliable

3	Acetylcholinesterase				
**Molecule**	**Predicted Outcome**	**Probability**	**Probability Active**	**Probability Inactive**	**Predicted Reliability**
O=S1(=O)CCCC2=C1C(N1N=NN=C1N2)C1=CC=CC=C1	Inactive	1.0	0.0	1.0	reliable

4	C_albicans				
**Molecule**	**Predicted Outcome**	**Probability**	**Probability Active**	**Probability Inactive**	**Predicted Reliability**
O=S1(=O)CCCC2=C1C(N1N=NN=C1N2)C1=CC=CC=C1	Active	1.0	1.0	0.0	reliable

5	Sars-Cov				
**Molecule**	**Predicted Outcome**	**Probability**	**Probability Active**	**Probability Inactive**	**Predicted Reliability**
O=S1(=O)CCCC2=C1C(N1N=NN=C1N2)C1=CC=CC=C1	Inactive	0.6	0.4	0.6	reliable

6	Hepatite C - Type1				
**Molecule**	**Predicted Outcome**	**Probability**	**Probability Active**	**Probability Inactive**	**Predicted Reliability**
O=S1(=O)CCCC2=C1C(N1N=NN=C1N2)C1=CC=CC=C1	Inactive	0.8	0.2	0.8	reliable

7	E_coli				
**Molecule**	**Predicted Outcome**	**Probability**	**Probability Active**	**Probability Inactive**	**Predicted Reliability**
O=S1(=O)CCCC2=C1C(N1N=NN=C1N2)C1=CC=CC=C1	Inactive	1.0	0.0	1.0	reliable

8	Hepatite C - NS3-protease helicase				
**Molecule**	**Predicted Outcome**	**Probability**	**Probability Active**	**Probability Inactive**	**Predicted Reliability**
O=S1(=O)CCCC2=C1C(N1N=NN=C1N2)C1=CC=CC=C1	Inactive	1.0	0.0	1.0	reliable

9	Hepatite C - Serine protease				
**Molecule**	**Predicted Outcome**	**Probability**	**Probability Active**	**Probability Inactive**	**Predicted Reliability**
O=S1(=O)CCCC2=C1C(N1N=NN=C1N2)C1=CC=CC=C1	Inactive	1.0	0.0	1.0	unreliable

10	Leishmania amazonensis - Promastigota				
**Molecule**	**Predicted Outcome**	**Probability**	**Probability Active**	**Probability Inactive**	**Predicted Reliability**
O=S1(=O)CCCC2=C1C(N1N=NN=C1N2)C1=CC=CC=C1	Inactive	1.0	0.0	1.0	reliable

11	Hepatite C - RNA dependent				
**Molecule**	**Predicted Outcome**	**Probability**	**Probability Active**	**Probability Inactive**	**Predicted Reliability**
O=S1(=O)CCCC2=C1C(N1N=NN=C1N2)C1=CC=CC=C1	Inactive	1.0	0.0	1.0	reliable

12	Leishmania infantum - Promastigota				
**Molecule**	**Predicted Outcome**	**Probability**	**Probability Active**	**Probability Inactive**	**Predicted Reliability**
O=S1(=O)CCCC2=C1C(N1N=NN=C1N2)C1=CC=CC=C1	Inactive	1.0	0.0	1.0	reliable

13	Leishmania braziliensis				
Molecule	Predicted Outcome	Probability	Probability Active	Probability Inactive	Predicted Reliability
O=S1(=O)CCCC2=C1C(N1N=NN=C1N2)C1=CC=CC=C1	Inactive	0.6	0.4	0.6	reliable

14	Drosophila melanogaster				
Molecule	Predicted Outcome	Probability	Probability Active	Probability Inactive	Predicted Reliability
O=S1(=O)CCCC2=C1C(N1N=NN=C1N2)C1=CC=CC=C1	Inactive	0.8	0.2	0.8	reliable

15	Leishmania major				
Molecule	Predicted Outcome	Probability	Probability Active	Probability Inactive	Predicted Reliability
O=S1(=O)CCCC2=C1C(N1N=NN=C1N2)C1=CC=CC=C1	Inactive	1.0	0.0	1.0	reliable

16	Alphis gossypii				
Molecule	Predicted Outcome	Probability	Probability Active	Probability Inactive	Predicted Reliability
O=S1(=O)CCCC2=C1C(N1N=NN=C1N2)C1=CC=CC=C1	Active	1.0	1.0	0.0	reliable

17	Alzheimer - COX2				
Molecule	Predicted Outcome	Probability	Probability Active	Probability Inactive	Predicted Reliability
O=S1(=O)CCCC2=C1C(N1N=NN=C1N2)C1=CC=CC=C1	Inactive	1.0	0.0	1.0	reliable

18	Alzheimer - JNK-3				
Molecule	Predicted Outcome	Probability	Probability Active	Probability Inactive	Predicted Reliability
O=S1(=O)CCCC2=C1C(N1N=NN=C1N2)C1=CC=CC=C1	Inactive	1.0	0.0	1.0	reliable

19	Alzheimer - NADPH				
Molecule	Predicted Outcome	Probability	Probability Active	Probability Inactive	Predicted Reliability
O=S1(=O)CCCC2=C1C(N1N=NN=C1N2)C1=CC=CC=C1	Inactive	1.0	0.0	1.0	reliable

20	Alzheimer - iNOS				
Molecule	Predicted Outcome	Probability	Probability Active	Probability Inactive	Predicted Reliability
O=S1(=O)CCCC2=C1C(N1N=NN=C1N2)C1=CC=CC=C1	Inactive	0.6	0.4	0.6	reliable

21	Alzheimer - PDE5				
Molecule	Predicted Outcome	Probability	Probability Active	Probability Inactive	Predicted Reliability
O=S1(=O)CCCC2=C1C(N1N=NN=C1N2)C1=CC=CC=C1	Inactive	1.0	0.0	1.0	reliable

22	Amastigote Chagas				
Molecule	Predicted Outcome	Probability	Probability Active	Probability Inactive	Predicted Reliability
O=S1(=O)CCCC2=C1C(N1N=NN=C1N2)C1=CC=CC=C1	Inactive	1.0	0.0	1.0	reliable

23	Amastigote Ldonovani				
Molecule	Predicted Outcome	Probability	Probability Active	Probability Inactive	Predicted Reliability
O=S1(=O)CCCC2=C1C(N1N=NN=C1N2)C1=CC=CC=C1	Inactive	1.0	0.0	1.0	reliable

24	Promastigote Ldonovani				
Molecule	Predicted Outcome	Probability	Probability Active	Probability Inactive	Predicted Reliability
O=S1(=O)CCCC2=C1C(N1N=NN=C1N2)C1=CC=CC=C1	Inactive	0.6	0.4	0.6	reliable

25	Epimastigote Chagas				
Molecule	Predicted Outcome	Probability	Probability Active	Probability Inactive	Predicted Reliability
O=S1(=O)CCCC2=C1C(N1N=NN=C1N2)C1=CC=CC=C1	Inactive	1.0	0.0	1.0	reliable

26	PTR L major				
Molecule	Predicted Outcome	Probability	Probability Active	Probability Inactive	Predicted Reliability
O=S1(=O)CCCC2=C1C(N1N=NN=C1N2)C1=CC=CC=C1	Inactive	0.6	0.4	0.6	reliable

27	Lamazonensis_amastigota				
Molecule	Predicted Outcome	Probability	Probability Active	Probability Inactive	Predicted Reliability
O=S1(=O)CCCC2=C1C(N1N=NN=C1N2)C1=CC=CC=C1	Inactive	1.0	0.0	1.0	unreliable

28	Tripomastigote Chagas				
Molecule	Predicted Outcome	Probability	Probability Active	Probability Inactive	Predicted Reliability
O=S1(=O)CCCC2=C1C(N1N=NN=C1N2)C1=CC=CC=C1	Active	0.6	0.6	0.4	reliable

29	Lamazonensis_promastigota				
**Molecule**	**Predicted Outcome**	**Probability**	**Probability Active**	**Probability Inactive**	**Predicted Reliability**
O=S1(=O)CCCC2=C1C(N1N=NN=C1N2)C1=CC=CC=C1	Inactive	1.0	0.0	1.0	reliable

30	Tcruzi_amastigota				
**Molecule**	**Predicted Outcome**	**Probability**	**Probability Active**	**Probability Inactive**	**Predicted Reliability**
O=S1(=O)CCCC2=C1C(N1N=NN=C1N2)C1=CC=CC=C1	Active	0.8	0.8	0.2	reliable

31	Tcruzi_epimastigota				
**Molecule**	**Predicted Outcome**	**Probability**	**Probability Active**	**Probability Inactive**	**Predicted Reliability**
O=S1(=O)CCCC2=C1C(N1N=NN=C1N2)C1=CC=CC=C1	Active	0.6	0.6	0.4	reliable

32	Tcruzi_trypomastigota				
**Molecule**	**Predicted Outcome**	**Probability**	**Probability Active**	**Probability Inactive**	**Predicted Reliability**
O=S1(=O)CCCC2=C1C(N1N=NN=C1N2)C1=CC=CC=C1	Inactive	0.8	0.2	0.8	reliable

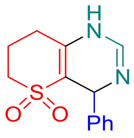

7

O=S1(=O)CCCC2=C1C(N=CN2)C1=CC=CC=C1

Molecule 1

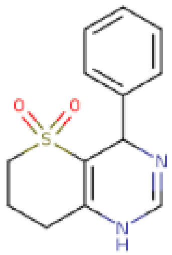

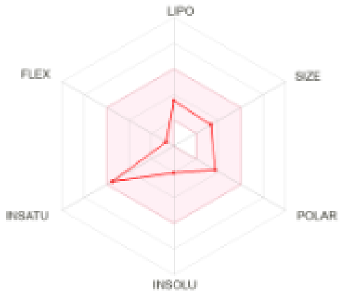

SMILES O=S1(=O)CCCC2=C1C(N=CN2)c1ccccc1

Physicochemical Properties	
Formula	C13H14N2O2S
Molecular weight	262.33 g/mol
Num. heavy atoms	18
Num. arom. heavy atoms	6
Fraction Csp3	0.31
Num. rotatable bonds	1
Num. H-bond acceptors	3
Num. H-bond donors	1
Molar Refractivity	78.24
TPSA	66.91 Å²

Lipophilicity	
Log $P_{o/w}$ (iLOGP)	1.29
Log $P_{o/w}$ (XLOGP3)	0.69
Log $P_{o/w}$ (WLOGP)	1.77
Log $P_{o/w}$ (MLOGP)	1.57
Log $P_{o/w}$ (SILICOS-IT)	2.00
Consensus Log $P_{o/w}$	1.46

Water Solubility	
Log *S* (ESOL)	-2.08
Solubility	2.17e+00 mg/ml ; 8.28e-03 mol/l
Class	Soluble
Log *S* (Ali)	-1.67
Solubility	5.58e+00 mg/ml ; 2.13e-02 mol/l
Class	Very soluble
Log *S* (SILICOS-IT)	-3.87
Solubility	3.54e-02 mg/ml ; 1.35e-04 mol/l
Class	Soluble

Pharmacokinetics	
GI absorption	High
BBB permeant	Yes
P-gp substrate	No
CYP1A2 inhibitor	No
CYP2C19 inhibitor	No
CYP2C9 inhibitor	No
CYP2D6 inhibitor	No
CYP3A4 inhibitor	No
Log K_p (skin permeation)	-7.41 cm/s

Druglikeness	
Lipinski	Yes; 0 violation
Ghose	Yes
Veber	Yes
Egan	Yes
Muegge	Yes
Bioavailability Score	0.55

Medicinal Chemistry	
PAINS	0 alert
Brenk	0 alert
Leadlikeness	Yes
Synthetic accessibility	4.11

Oral toxicity prediction results for input compound

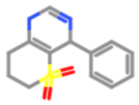

Predicted LD50: 1000mg/kg

Predicted Toxicity Class: 4

1	2	3	4	5	6

Average similarity: 31.56%

Prediction accuracy: 23%

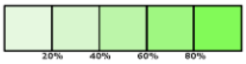

Name	O=S1(=O)CCCC2=C1C(
Molweight	262.33
Number of hydrogen bond acceptors	4
Number of hydrogen bond donors	1
Number of atoms	18
Number of bonds	20
Number of rotable bonds	1
Molecular refractivity	78.24
Topological Polar Surface Area	66.91
octanol/water partition coefficient(logP)	2.62

1	Dengue larvicida				
Molecule	Predicted Outcome	Probability	Probability Active	Probability Inactive	Predicted Reliability
O=S1(=O)CCCC2=C1C(N=CN2)C1=CC=CC=C1	Inactive	0.6	0.4	0.6	reliable

2	Sars-Cov				
Molecule	Predicted Outcome	Probability	Probability Active	Probability Inactive	Predicted Reliability
O=S1(=O)CCCC2=C1C(N=CN2)C1=CC=CC=C1	Active	0.6	0.6	0.4	reliable

3	Acetylcholinesterase				
Molecule	Predicted Outcome	Probability	Probability Active	Probability Inactive	Predicted Reliability
O=S1(=O)CCCC2=C1C(N=CN2)C1=CC=CC=C1	Inactive	1.0	0.0	1.0	reliable

4	C_albicans				
Molecule	Predicted Outcome	Probability	Probability Active	Probability Inactive	Predicted Reliability
O=S1(=O)CCCC2=C1C(N=CN2)C1=CC=CC=C1	Active	1.0	1.0	0.0	reliable

5	Salmonella				
Molecule	Predicted Outcome	Probability	Probability Active	Probability Inactive	Predicted Reliability
O=S1(=O)CCCC2=C1C(N=CN2)C1=CC=CC=C1	Inactive	1.0	0.0	1.0	reliable

6	E_coli				
Molecule	Predicted Outcome	Probability	Probability Active	Probability Inactive	Predicted Reliability
O=S1(=O)CCCC2=C1C(N=CN2)C1=CC=CC=C1	Active	0.8	0.8	0.2	reliable

7	Hepatite C - Type1				
Molecule	Predicted Outcome	Probability	Probability Active	Probability Inactive	Predicted Reliability
O=S1(=O)CCCC2=C1C(N=CN2)C1=CC=CC=C1	Inactive	0.8	0.2	0.8	reliable

8	Hepatite C - NS3-protease helicase				
Molecule	Predicted Outcome	Probability	Probability Active	Probability Inactive	Predicted Reliability
O=S1(=O)CCCC2=C1C(N=CN2)C1=CC=CC=C1	Inactive	1.0	0.0	1.0	reliable

9	Hepatite C - Serine protease				
Molecule	Predicted Outcome	Probability	Probability Active	Probability Inactive	Predicted Reliability
O=S1(=O)CCCC2=C1C(N=CN2)C1=CC=CC=C1	Inactive	1.0	0.0	1.0	unreliable

10	Hepatite C - RNA dependent				
Molecule	Predicted Outcome	Probability	Probability Active	Probability Inactive	Predicted Reliability
O=S1(=O)CCCC2=C1C(N=CN2)C1=CC=CC=C1	Inactive	1.0	0.0	1.0	reliable

11	Leishmania infantum - Promastigota				
Molecule	Predicted Outcome	Probability	Probability Active	Probability Inactive	Predicted Reliability
O=S1(=O)CCCC2=C1C(N=CN2)C1=CC=CC=C1	Inactive	1.0	0.0	1.0	reliable

12	Leishmania amazonensis - Promastigota				
Molecule	Predicted Outcome	Probability	Probability Active	Probability Inactive	Predicted Reliability
O=S1(=O)CCCC2=C1C(N=CN2)C1=CC=CC=C1	Inactive	0.6	0.4	0.6	reliable

13	Leishmania braziliensis				
**Molecule**	**Predicted Outcome**	**Probability**	**Probability Active**	**Probability Inactive**	**Predicted Reliability**
O=S1(=O)CCCC2=C1C(N=CN2)C1=CC=CC=C1	Inactive	0.6	0.4	0.6	reliable

14	Drosophila melanogaster				
**Molecule**	**Predicted Outcome**	**Probability**	**Probability Active**	**Probability Inactive**	**Predicted Reliability**
O=S1(=O)CCCC2=C1C(N=CN2)C1=CC=CC=C1	Inactive	0.6	0.4	0.6	reliable

15	Leishmania major				
**Molecule**	**Predicted Outcome**	**Probability**	**Probability Active**	**Probability Inactive**	**Predicted Reliability**
O=S1(=O)CCCC2=C1C(N=CN2)C1=CC=CC=C1	Inactive	1.0	0.0	1.0	reliable

16	Alphis gossypii				
**Molecule**	**Predicted Outcome**	**Probability**	**Probability Active**	**Probability Inactive**	**Predicted Reliability**
O=S1(=O)CCCC2=C1C(N=CN2)C1=CC=CC=C1	Active	1.0	1.0	0.0	reliable

17	Alzheimer - iNOS				
**Molecule**	**Predicted Outcome**	**Probability**	**Probability Active**	**Probability Inactive**	**Predicted Reliability**
O=S1(=O)CCCC2=C1C(N=CN2)C1=CC=CC=C1	Inactive	1.0	0.0	1.0	reliable

18	Alzheimer - NADPH				
**Molecule**	**Predicted Outcome**	**Probability**	**Probability Active**	**Probability Inactive**	**Predicted Reliability**
O=S1(=O)CCCC2=C1C(N=CN2)C1=CC=CC=C1	Inactive	0.8	0.2	0.8	reliable

19	Alzheimer - COX2				
**Molecule**	**Predicted Outcome**	**Probability**	**Probability Active**	**Probability Inactive**	**Predicted Reliability**
O=S1(=O)CCCC2=C1C(N=CN2)C1=CC=CC=C1	Inactive	1.0	0.0	1.0	reliable

20	Alzheimer - JNK-3				
**Molecule**	**Predicted Outcome**	**Probability**	**Probability Active**	**Probability Inactive**	**Predicted Reliability**
O=S1(=O)CCCC2=C1C(N=CN2)C1=CC=CC=C1	Inactive	1.0	0.0	1.0	reliable

21	Alzheimer - PDE5				
**Molecule**	**Predicted Outcome**	**Probability**	**Probability Active**	**Probability Inactive**	**Predicted Reliability**
O=S1(=O)CCCC2=C1C(N=CN2)C1=CC=CC=C1	Inactive	1.0	0.0	1.0	reliable

22	Amastigote Ldonovani				
**Molecule**	**Predicted Outcome**	**Probability**	**Probability Active**	**Probability Inactive**	**Predicted Reliability**
O=S1(=O)CCCC2=C1C(N=CN2)C1=CC=CC=C1	Inactive	1.0	0.0	1.0	reliable

23	Amastigote Chagas				
**Molecule**	**Predicted Outcome**	**Probability**	**Probability Active**	**Probability Inactive**	**Predicted Reliability**
O=S1(=O)CCCC2=C1C(N=CN2)C1=CC=CC=C1	Inactive	1.0	0.0	1.0	reliable

24	Epimastigote Chagas				
**Molecule**	**Predicted Outcome**	**Probability**	**Probability Active**	**Probability Inactive**	**Predicted Reliability**
O=S1(=O)CCCC2=C1C(N=CN2)C1=CC=CC=C1	Inactive	1.0	0.0	1.0	reliable

25	Promastigote Ldonovani				
**Molecule**	**Predicted Outcome**	**Probability**	**Probability Active**	**Probability Inactive**	**Predicted Reliability**
O=S1(=O)CCCC2=C1C(N=CN2)C1=CC=CC=C1	Inactive	0.8	0.2	0.8	reliable

26	PTR L major				
**Molecule**	**Predicted Outcome**	**Probability**	**Probability Active**	**Probability Inactive**	**Predicted Reliability**
O=S1(=O)CCCC2=C1C(N=CN2)C1=CC=CC=C1	Inactive	1.0	0.0	1.0	reliable

27	Lamazonensis_promastigota				
**Molecule**	**Predicted Outcome**	**Probability**	**Probability Active**	**Probability Inactive**	**Predicted Reliability**
O=S1(=O)CCCC2=C1C(N=CN2)C1=CC=CC=C1	Inactive	1.0	0.0	1.0	reliable

28	Lamazonensis_amastigota				
**Molecule**	**Predicted Outcome**	**Probability**	**Probability Active**	**Probability Inactive**	**Predicted Reliability**
O=S1(=O)CCCC2=C1C(N=CN2)C1=CC=CC=C1	Inactive	1.0	0.0	1.0	reliable

29	Tripomastigote Chagas				
**Molecule**	**Predicted Outcome**	**Probability**	**Probability Active**	**Probability Inactive**	**Predicted Reliability**
O=S1(=O)CCCC2=C1C(N=CN2)C1=CC=CC=C1	Active	1.0	1.0	0.0	reliable

30	Tcruzi_amastigota				
**Molecule**	**Predicted Outcome**	**Probability**	**Probability Active**	**Probability Inactive**	**Predicted Reliability**
O=S1(=O)CCCC2=C1C(N=CN2)C1=CC=CC=C1	Inactive	1.0	0.0	1.0	reliable

31	Tcruzi_epimastigota				
**Molecule**	**Predicted Outcome**	**Probability**	**Probability Active**	**Probability Inactive**	**Predicted Reliability**
O=S1(=O)CCCC2=C1C(N=CN2)C1=CC=CC=C1	Active	0.6	0.6	0.4	reliable

32	Tcruzi_trypomastigota				
**Molecule**	**Predicted Outcome**	**Probability**	**Probability Active**	**Probability Inactive**	**Predicted Reliability**
O=S1(=O)CCCC2=C1C(N=CN2)C1=CC=CC=C1	Inactive	0.8	0.2	0.8	reliable

Plausible mechanisms of desulfurization for compound 2a

Due to the strong thiophilic affinity of Hg^{2+}, mercury(II) acetate was employed to form HgS. We utilized a strategy where the azido group was generated by the desulfurization of compound **2a** with Hg^{2+} ions. The introduction of Hg^{2+} ions triggered the N_3^- to attack the 2-C atom of the pyrimidine ring, leading to the removal of HgS and the formation of an intramolecular guanylation product **6**, through an irreversible desulfurization reaction. When using mild oxidant such as hydrogen peroxide under vanadyl(IV) sulfate catalysis, the thione group of **2a** was oxidized to the cyclic sulfinate **A** which loses sulfur dioxide to form transient N-heterocyclic carbene species **B**, and its spontaneous rearrangement provides the dihydropyrimidine **7** (Scheme). For further reading please see [J. Chem. Sci., 2018, 130, 46, https://doi.org/10.1007/s12039-018-1453-0; Synlett, 2009, 4, 599-602; DOI: 10.1055/s-0028-1087920]

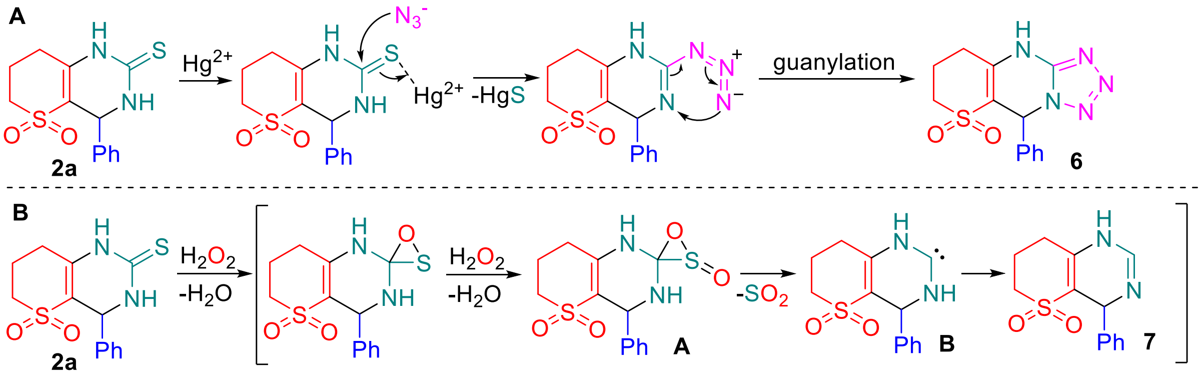

Scheme: Plausible mechanisms for desulfurization and oxidation chemistry **2a** for the formation of products **6** (**A**) and **7** (**B**).
